# Six RNA Viruses and Forty-One Hosts: Viral Small RNAs and Modulation of Small RNA Repertoires in Vertebrate and Invertebrate Systems

**DOI:** 10.1371/journal.ppat.1000764

**Published:** 2010-02-12

**Authors:** Poornima Parameswaran, Ella Sklan, Courtney Wilkins, Trever Burgon, Melanie A. Samuel, Rui Lu, K. Mark Ansel, Vigo Heissmeyer, Shirit Einav, William Jackson, Tammy Doukas, Suman Paranjape, Charlotta Polacek, Flavia Barreto dos Santos, Roxana Jalili, Farbod Babrzadeh, Baback Gharizadeh, Dirk Grimm, Mark Kay, Satoshi Koike, Peter Sarnow, Mostafa Ronaghi, Shou-Wei Ding, Eva Harris, Marie Chow, Michael S. Diamond, Karla Kirkegaard, Jeffrey S. Glenn, Andrew Z. Fire

**Affiliations:** 1 Department of Microbiology & Immunology, Stanford University School of Medicine, Stanford, California, United States of America; 2 Department of Gastroenterology & Hepatology, Stanford University School of Medicine, Stanford, California, United States of America; 3 Department of Microbiology & Immunology, University of Arkansas for Medical Sciences, Little Rock, Arkansas, United States of America; 4 Department of Molecular Microbiology, Washington University School of Medicine, St. Louis, Missouri, United States of America; 5 Department of Plant Pathology & Microbiology, University of California at Riverside, Riverside, California, United States of America; 6 Strategic Asthma Basic Research Center and the Department of Microbiology & Immunology, University of California at San Francisco, San Francisco, California, United States of America; 7 Institute of Molecular Immunology, Helmholtz Center Munich, German Research Center for Environmental Health, Munich, Germany; 8 Division of Infectious Diseases and Vaccinology, School of Public Health, University of California at Berkeley, Berkeley, California, United States of America; 9 Stanford Genome Technology Center, Stanford University School of Medicine, Stanford, California, United States of America; 10 Departments of Pediatrics & Genetics, Stanford University School of Medicine, Stanford, California, United States of America; 11 Tokyo Metropolitan Organization for Medical Research, Tokyo Metropolitan Institute of Medical Science, Tokyo, Japan; 12 Departments of Medicine, Molecular Microbiology, Pathology & Immunology, Washington University School of Medicine, St. Louis, Missouri, United States of America; 13 Departments of Pathology & Genetics, Stanford University School of Medicine, Stanford, California, United States of America; The Rockefeller University, United States of America

## Abstract

We have used multiplexed high-throughput sequencing to characterize changes in small RNA populations that occur during viral infection in animal cells. Small RNA-based mechanisms such as RNA interference (RNAi) have been shown in plant and invertebrate systems to play a key role in host responses to viral infection. Although homologs of the key RNAi effector pathways are present in mammalian cells, and can launch an RNAi-mediated degradation of experimentally targeted mRNAs, any role for such responses in mammalian host-virus interactions remains to be characterized. Six different viruses were examined in 41 experimentally susceptible and resistant host systems. We identified virus-derived small RNAs (vsRNAs) from all six viruses, with total abundance varying from “vanishingly rare” (less than 0.1% of cellular small RNA) to highly abundant (comparable to abundant micro-RNAs “miRNAs”). In addition to the appearance of vsRNAs during infection, we saw a number of specific changes in host miRNA profiles. For several infection models investigated in more detail, the RNAi and Interferon pathways modulated the abundance of vsRNAs. We also found evidence for populations of vsRNAs that exist as duplexed siRNAs with zero to three nucleotide 3′ overhangs. Using populations of cells carrying a Hepatitis C replicon, we observed strand-selective loading of siRNAs onto Argonaute complexes. These experiments define vsRNAs as one possible component of the interplay between animal viruses and their hosts.

## Author Summary

Short RNAs derived from invading viruses with RNA genomes are important components of antiviral immunity in plants, worms and flies. The regulated generation of these short RNAs, and their engagement by the immune apparatus, is essential for inhibiting viral growth in these organisms. Mammals have the necessary protein components to generate these viral-derived short RNAs (“vsRNAs”), raising the question of whether vsRNAs in mammals are a general feature of infections with RNA viruses. Our work with Hepatitis C, Polio, Dengue, Vesicular Stomatitis, and West Nile viruses in a broad host repertoire demonstrates the generality of RNA virus-derived vsRNA production, and the ability of the cellular short RNA apparatus to engage these vsRNAs in mammalian cells. Detailed analyses of vsRNA and host-derived short RNA populations demonstrate both common and virus-specific features of the interplay between viral infection and short RNA populations. The vsRNA populations described in this work represent a novel dimension in both viral pathogenesis and host response.

## Introduction

Biological systems are protected by innate immune mechanisms initiated by host sensors called pattern recognition receptors (‘PRRs’) that recognize specific “foreign” features of invading pathogens to initiate multiple downstream anti-pathogen cascades. PRRs that detect nucleic acid structures characteristic of viral infection (such as single- or double-stranded RNA or DNA) are among the innate responders that protect diverse cell types from viral pathogenesis (for review, see [1],[2]). How the cell handles viral double-stranded RNA (dsRNA) is of special interest because dsRNA is a necessary intermediate in the replication of RNA viruses. In addition to dsRNA that forms during replication of the virus genome, RNA duplexes can form due to self-complementarity in the virus genome, and in some instances, from sense-antisense transcription of overlapping genes.

Four of the most studied families of PRRs for dsRNA are: (a) cytoplasmic RNA helicases like Retinoic acid-inducible gene I & Melanoma differentiation-associated gene-5 (“RIG-I” & “Mda-5,” which trigger mitochondrial-localized antiviral pathways); (b) Protein Kinase R (“PKR,” which induces a translational arrest state in cells after sensing dsRNA); (c) 2′–5′ oligoadenylate synthetase (“OAS,” which stimulates the ssRNase activity of RNase L in response to dsRNA); and (d) Toll-like receptors (“TLRs,” which bind various forms of RNA or DNA). All of these PRRs trigger the Interferon (IFN) responses, and activate IFN-stimulated genes (ISGs) that establish an antiviral state in the infected cell (for review, see [3]). The IFN signaling pathway is central to the detection of, and response to, virus infections in cells. Type I IFNs (IFN-α and IFN-β) make up one of the first lines of defense in the innate immune response to viruses by inducing antiviral ISGs, modulating the levels of specific host-encoded miRNAs [4], and in a feedback loop, that of PKR and OAS. Many viruses are also susceptible to treatment with Type I IFNs, and conversely, cells that have higher basal activity of ISGs seem to mount a more successful antiviral response, and are not targeted by viruses [5].

Dicer is another PRR that recognizes dsRNA, chopping it into smaller duplexes called siRNAs that are 19–27 nucleotides (nt) long [6],[7]. These siRNAs have a terminal 5′ mono-phosphate and a terminal 3′ hydroxyl on both strands, generally have 2 nt 3′ overhangs, and are fed into an RNA-induced silencing complex “RISC” (for review on Dicer and Argonautes, see [8],[9]). siRNA duplexes are unwound, and only one strand remains associated with RISC (the mechanism of unwinding and choice of strand is poorly understood; for review, see [10]). One of the key components of RISC is a protein called Argonaute-2 (Ago-2), which belongs to the Argonaute family of proteins. Ago-2 is the only member of the family that has cleavage activity, and is the designated ‘slicer’ protein in RISC that mediates cleavage of mRNA in a sequence-directed manner by a process termed RNA interference, or ‘RNAi’ [11],[12],[13],[14].

There is strong evidence for an antiviral role for RNAi in plant and invertebrate systems (for review, see [15],[16],[17]). Viruses replicate most effectively in these systems in the absence of key elements of the RNAi pathway: either in cells lacking components of the RNAi machinery, or in the presence of virus-encoded suppressors of the silencing pathway (for review, see [18],[19]). As expected, virus-derived siRNAs (vsRNAs) can be detected in some plant and invertebrate systems that are capable of mounting a successful/partially successful RNAi response [15],[16],[17]. A population of vsRNAs would be an expected component of any viral defense pathway that acted through an RNAi mechanism.

In mammalian cells, short duplex RNAs can effectively enter the RNAi pathway and function in sequence-specific silencing, while duplexes longer than 30 nt generally produce a more complex response including the induction of multiple non-specific pathways including the IFN response (for review, see [20],[21]). Indeed, RNA and DNA viruses have evolved a host of defense mechanisms to counteract the nonspecific signaling effects of dsRNA. For example, Adenovirus VA RNA sequesters PKR [22], while proteins from Vaccinia virus (E3L), Porcine Rotaviruses (NSP3), and Influenza A virus (NS1) sequester dsRNA and prevent stimulation of the IFN response [23],[24],[25],[26]. Viral proteins can also inhibit signaling downstream of dsRNA binding, as in the case of the HCV protease NS3/4A, which cleaves IPS-1 (the RIG-I/MDA-5 signaling partner) to consequently disrupt induction of IFN responses [27]. Several of these dsRNA-binding proteins may also facilitate viral evasion of host immune responses by inhibiting RNAi [28]. Additionally, some viruses make their genomes inaccessible to PRRs of various types including IFN effectors and the siRNA-programmed RISC complex (e.g. [29]).

Viruses may also perturb another class of effectors involved in RNAi called micro-RNAs (miRNAs), which are a class of cellular small RNAs generated by Dicer from hairpin structures. Cellular miRNA profiles are frequently modulated upon infection by viruses, and this may contribute in some cases to infectivity and pathogenesis [30]. Conversely, some viruses usurp the host miRNA machinery for processing miRNA-like structures encoded in the viral genome, potentially using these molecules for regulation of virus/host gene expression [31].

With so much potential for RNA-mediated cross talk between the IFN response, the RNAi pathway, and the virus itself, it has been difficult to demonstrate a precise role for the RNAi pathway in vertebrate antiviral defense. The difficulties in segregating IFN and RNAi functions have given rise to speculations that the antiviral role of RNAi may have been lost during evolution, or alternatively, that RNAi-based defense may only be harnessed by triggers such as short hairpins and siRNAs that do not stimulate the IFN pathway. There has been some attempt at demonstrating recognition of viral RNA by the RNAi machinery. For instance, in Vero cells (which lack IFNα/β), inhibition of RNAi by Dicer knockdown increases replication of an RNA virus, the Influenza A Virus [32]. Additionally, there are cases where short virus-derived RNAs can be detected in vertebrate systems (e.g. from HDV [33] by high-throughput sequencing, and the HCV replicon [34], by bulk analysis methods). However, it is still not clear how general the presence of such RNAs is, and whether these RNAs can participate in host defense mechanisms. To complicate this issue, many of the classically-studied virus-host systems have been chosen based on the ability of the virus to rapidly replicate and kill host cells; these experimental infection systems may artificially under-represent the capacity of vertebrate cells to protect themselves, hence biasing against systems where RNAi might have a significant role in host-virus interactions.

Here, we sought a broader survey of potential RNA-derived defenses in viral infection systems. Given no knowledge of which virus type might engage the RNAi machinery, and which cell types might efficiently use this machinery in defense, we cast a wide net in terms of both virus families and host cells. In this study, we describe small RNA populations from six different RNA viral pathogens, each in a variety of animal cell infection systems (including both immune-competent and immune-compromised hosts). Upon examining small RNA populations from ∼150 samples with sample-specific DNA barcodes, we found viral-derived small RNAs (vsRNAs) from each virus, with vsRNA populations sensitive to both viral and host characteristics. A more detailed analysis of vsRNAs in two viral infection models (Hepatitis C Virus and Poliovirus) in various host types revealed that multiple distinct pools of vsRNAs may co-exist during infection: as single strands, as part of duplexes, and in complexes that may contain Argonautes. We also observed specific changes in cell-derived miRNA populations, providing a clear indication of host perturbation by the virus. The characterization of small RNA populations during RNA virus infections provides both an experimental entry point, and an indication of the complexity that will need to be addressed in understanding roles for small RNAs in host and viral processes.

## Results

### Detection and analysis of small RNA populations during viral infection

**Figure 1:**
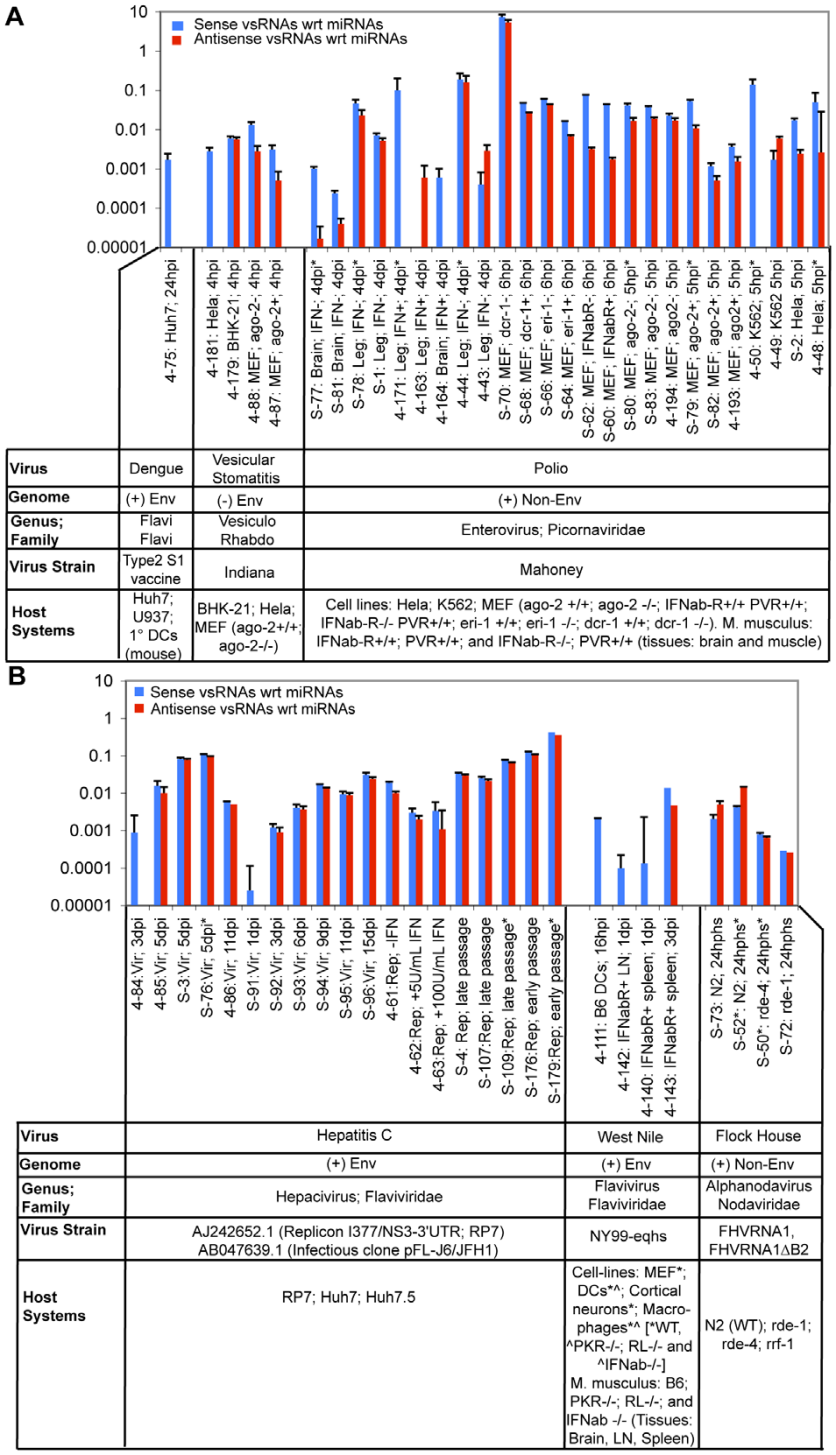
Virus-derived vsRNA abundance varies as a function of virus type & strain, host type & genotype, time post-infection, and cloning method used. Abundance of vsRNAs in various host systems infected with (**1A**) Dengue Virus, Vesicular Stomatitis Virus, or Polio Virus, and (**1B**) Hepatitis C Virus, West Nile Virus, or Flock House Virus. Samples sequenced on the Solexa platform are prefixed with ‘S-,’ while samples sequenced on the GS-20/GS-FLX are pre-fixed with ‘4-.’ The asterisks indicate vsRNAs from RNA pools captured using the 5′-P-INDependent cloning protocol. Samples that had no detectable vsRNAs were not plotted. Levels of vsRNAs in these samples (sense ‘BLUE’ or antisense ‘RED’ relative to the mRNA of the virus) are represented as a ratio relative to the count of all miRNAs (i.e. v/miR). miRNA sequences are defined in species-specific miRNA databases obtained from miRBase *ver*9.2. **Note:** v/miR values are represented on a logarithmic scale.

In the following sections, we will describe small RNA populations present during infection of animal cells with six different viruses. In each case, we have taken infected cells, extracted small RNA populations, and characterized these populations using high-throughput sequencing methods. Two high-throughput sequencing platforms were used: Roche/454 pyrosequencing (http://www.454.com/), to obtain several hundred thousand sequences from pools of appropriately linkered amplicon templates; and Solexa/Illumina technology, which yields larger datasets of shorter reads (http://www.illumina.com/). Due to the large number of samples to be analyzed, we used DNA barcodes to ‘tag’ RNA samples from individual experiments, which facilitated sequencing in parallel from multiple samples. This allowed us to work with samples from different viral systems and diverse experimental conditions in a cost-effective manner, with a small number of instrument runs. Viral-derived sequences were identified in sequence datasets through pattern matching using standard software (BLAT [35] and BLAST [36]). We use the term ‘vsRNA’ to refer to small RNA segments whose sequences show perfect complementarity to the infecting viral genome at every base position (reference genomes listed in **Table S1**). vsRNAs are distinct from host-derived miRNAs that may show partial complementarity to sites in the viral genome (e.g. [37],[38],[39], **Fig. S1**). We detected 77,609 vsRNAs out of 19,425,777 sequences from 151 datasets (**Fig. 1** **, Figure S2: length distributions**). The most abundant vsRNAs from each virus are listed in **Table S2**.

For a small number of vsRNAs (0.033%) we observed a perfect match to both the host and viral genomes (**Table S3, Table S4**). The fractions of vsRNAs that matched host genomes were approximately as expected by random sequence coincidence (for example: the human genome, with a unique genome complexity of 2×10^9^ bp, would match approximatly 1 in 4000 arbitrary 22-mer sequences). The perfect nature of the homology makes it difficult to determine whether this minor class of sRNAs was derived from the host or from the virus.

Furthermore, to validate the specificity of the barcoding and sequencing assays, we carried out sequence comparisons to the full set of viruses for each experimental sample. We identified 13 vsRNAs that were ‘rogue’ hits i.e. mapped to one of the other 5 viruses not used in that particular experiment. In no sample were the ‘rogue’ matches present at more than 0.008% of all parsed sequences (**Table S3**).

For certain purposes, it will be of interest to compare vsRNA incidences in different samples. Such comparisons require some normalization for total depth of RNA sequencing. In **Table S3**, we provide two distinct normalizations for each sample: normalization to total small RNAs recovered and sequenced (v/sRNA), and normalization to the population of cellular miRNAs that are expected to represent a large proportion of bona-fide small RNA effectors (v/miR; miRNAs are defined as documented in miRBase *ver*9.2 [40],[41],[42]). There is a substantial challenge in choosing and interpreting appropriate normalization schemes: any change in sample character that results in increased levels of non-specific degradation of RNA will increase the levels of non-specific decay products (which may include decay products of both cellular and viral long RNAs), and impact both v/miR and v/sRNA ratios. Another important consideration is whether the difference in v/miR (or v/sRNA) ratios between two samples is above the variance in ratios observed between technical replicates. For all relevant technical replicates in our analysis, the variances in v/miR and v/sRNA ratios were <3-fold and <1.5-fold, respectively. These notes provide caution in interpreting small differences in normalization values between samples.

In describing the results of this work, we have taken care to avoid any *a-priori* assumption that small RNAs identified by sequencing play a functional role in gene silencing, viral pathogenesis, or host response. In the *Discussion* section, we will summarize arguments pertaining to this question.

### vsRNAs in an invertebrate infection model *(C. elegans)*

Components of the worm RNAi machinery such as the argonaute, *rde-1* [43], the dsRNA binding protein, *rde-4* [44],[45], and the RNA-dependent RNA Polymerase or RdRP, *rrf-1* [46] are essential for protection against Vesicular Stomatitis Virus ‘VSV’ [47], and Flock House Virus ‘FHV’ replication [48]. To characterize small RNA populations in an animal system known to utilize the RNAi machinery in antiviral defense, we used *C. elegans* experimentally infected with FHV RNA1ΔB2 (FHV RNA1 that expresses a mutant version of the RNAi suppressor protein, B2; [48]).

Two different vsRNA capture and library production schemes were used to enrich for Dicer products or for RdRP products, both of which have structures distinct from those of RNA fragments generated by alkali-induced degradation. The first (5′-phosphate-dependent cloning) requires a single phosphate at the 5′ end of the RNA, and allows for the capture of Dicer products (which have a mono-Phosphate and a hydroxyl moiety at their 5′ and 3′ termini). The second (5′-phosphate-independent cloning; [49]) is designed to capture RNA populations with any number of 5′ phosphates (zero, mono, di, tri), including both RdRP products (which have a tri-Phosphate and a hydroxyl moiety at their 5′ and 3′ termini) and Dicer products. Both procedures require a 3′ end that can ligate to a pre-adenylated linker, and allow for the capture of 3′-OH and 2′-O-Methyl structures but not 3′ phosphate termini, thus minimizing the extent of capture of degradation products (many of which have 3′ mono-phosphate termini).

**Figure 2:**
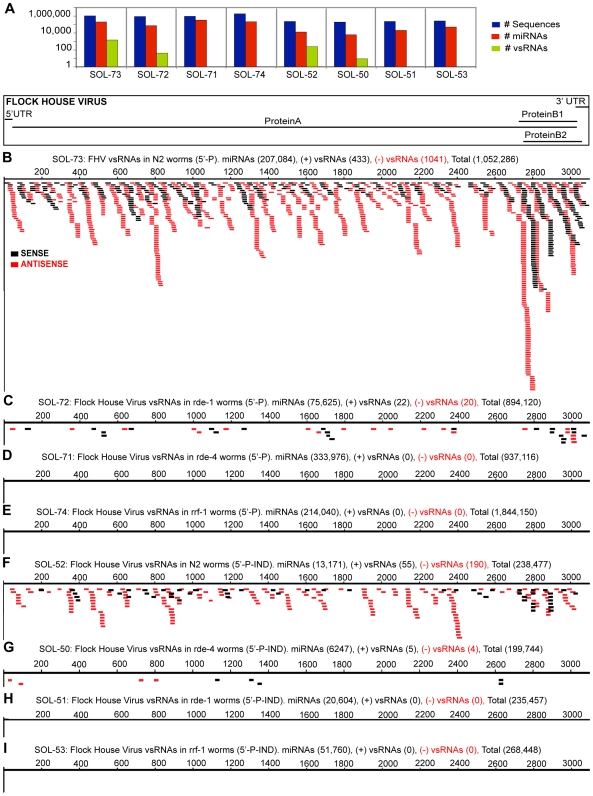
Flock House Virus-derived vsRNAs are more abundant in RNAi-competent worms, and exist as both 5′-monophosphorylated, and 5′-triphosphorylated species. The incidence, strandedness and lengths of vsRNAs are drawn as a function of their position along the viral genome. Each filled box represents one instance of a captured vsRNA, with the lengths of the boxes proportional to the lengths of the vsRNAs. vsRNAs from the positive and negative strands are shaded black and red respectively. All samples were sequenced on Illumina's platform. (**2A**) Sequence counts for all small RNAs, miRNAs, vsRNAs (Y-axis: log scale). 5′-P vsRNAs from wild-type *Bristol N2* (**2B**; Sol-73), *rde-1* (**2C**; Sol-72), *rde-4* (**2D**; Sol-71), and *rrf-1* (**2E**; Sol-74) worms, 24 hours post-heat-shock. 5′-xP vsRNAs from wild-type *Bristol N2* (**2F**; Sol-52), *rde-4* (**2G**; Sol-50), *rde-1* (**2H**; Sol-51), and *rrf-1* (**2I**; Sol-53) worms, 24 hours post-heat-shock.

5′ mono-phosphorylated (5′-P) vsRNAs were present during abortive FHV RNA1ΔB2 replication in wild-type animals (v/miR = 0.007; **Fig. 2B**). vsRNAs were absent in two RNAi-defective mutants, *rrf-1(pk1417)I* and *rde-4(ne299)III* (**Table S3**), while as predicted, genomic viral RNA replicated to high levels in these mutants (**Parameswaran P, unpublished**). Similarly, vsRNAs were much reduced (19-fold; *P*-value = 2.3E-227) in *rde-1(ne300)V mutants* (**Fig. 2C**). We also observed a difference in strand ratios of vsRNAs (Positive∶Negative) between strains: 1∶2.4 in wild-type, versus 1∶1.1 in the mutant, *rde-1* (*P*-value = 0.0016).

The population of RNAs captured with no requirement for a 5′-P terminus (i.e. 5′-xP RNAs) yielded a stronger signature for vsRNAs in wild-type worms with replicating RNA1ΔB2 (v/miR = 0.019; **Fig. 2F**). Fewer vsRNAs mapped to the positive strand of FHV than to the negative strand, with a Positive∶Negative vsRNA strand ratio of 1∶3.5 (*P*-value = 1.1E-48). *rde-4−/−* was the only RNAi-defective mutant that yielded a detectable signature for 5′-xP vsRNAs (v/miR = 0.0014), with a strand ratio (Positive∶Negative) of 1.3∶1 (**Fig. 2G**). Interestingly, in wild-type worms, both 5′-P and 5′-xP vsRNAs were distributed throughout the length of the genome, with increased frequencies of positive-strand vsRNAs detected in the 3′ region that also encodes the subgenomic RNA species RNA3 (**Fig. 2B, 2F**).

### vsRNAs in various mammalian host-virus infection models

**Figure 3:**
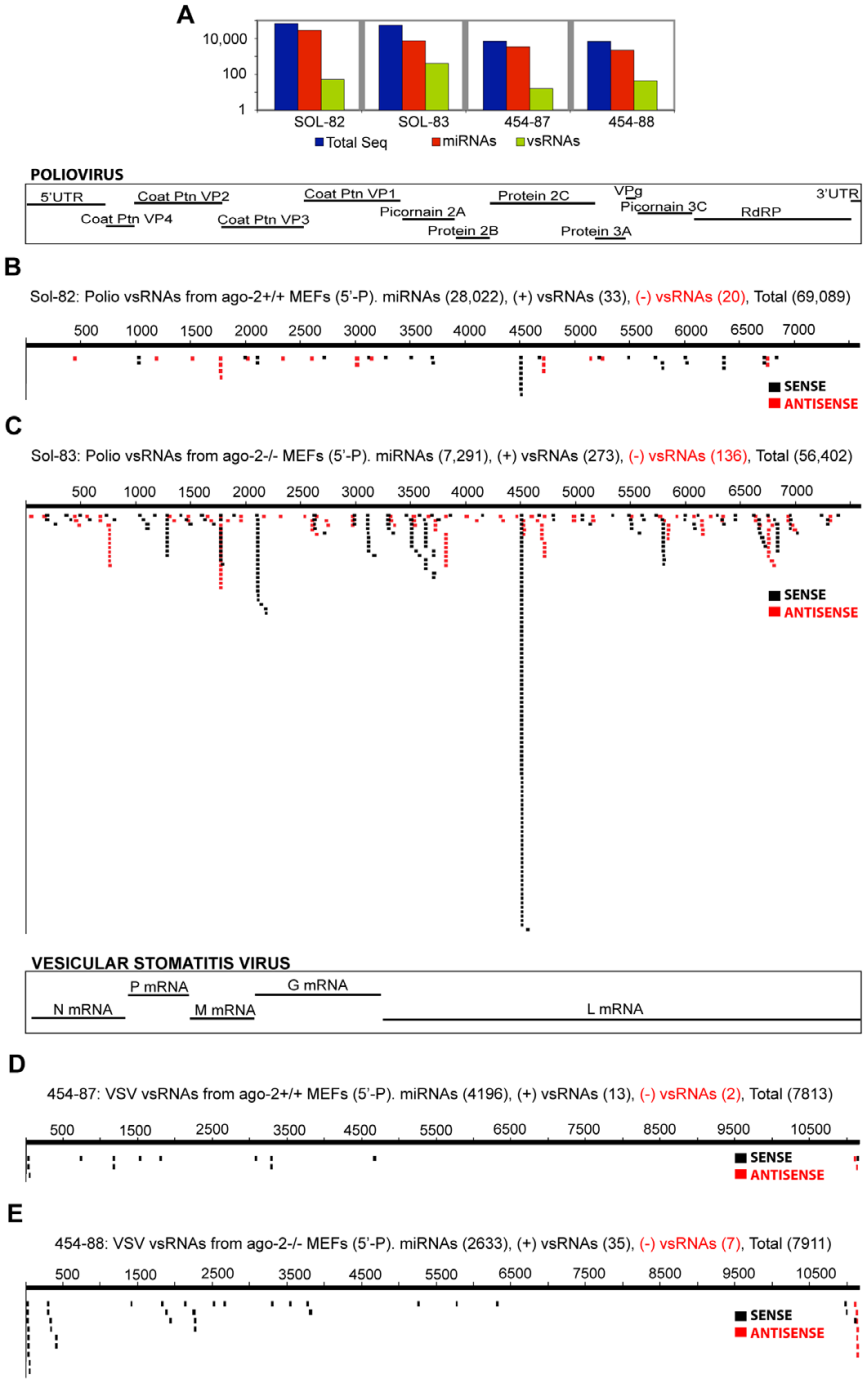
Poliovirus- and Vesicular Stomatitis Virus-derived vsRNAs are more abundant in MEFs deficient in Argonaute-2. Samples sequenced on the Solexa platform are prefixed with ‘Sol-,’ while samples sequenced on the GS-20/GS-FLX are pre-fixed with ‘454-.’ (**3A**) Sequence count: all RNAs, miRNAs, vsRNAs (Y-axis: log scale). vsRNAs with a 5′-monophosphate moiety from *ago-2+/+* MEFs (**3B**; Sample: Sol-82) and *ago-2−/−* MEFs (**3C**; Sample: Sol-83), transfected with a plasmid encoding for full-length, self-replicating Poliovirus RNA. vsRNAs with a 5′-monophosphate moiety from *ago-2+/+* MEFs (**3D**; Sample: 454-87) and *ago-2−/−* MEFs (**3E**; Sample: 454-88) infected with Vesicular Stomatitis Virus.

**Figure 4:**
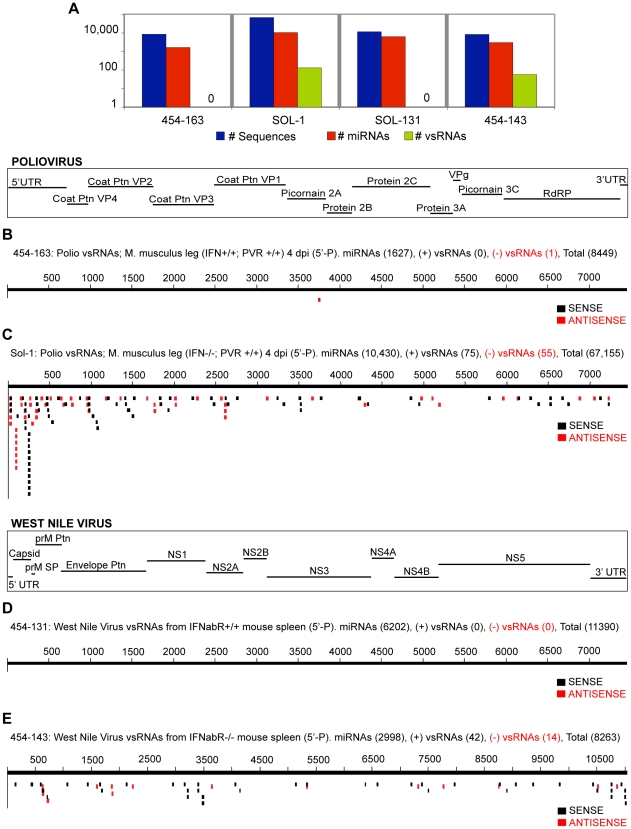
vsRNAs are abundant in infected hosts that do not have a functional Interferon-αβ Receptor. (**4A**) Sequence count: all RNAs, miRNAs, vsRNAs (Y-axis: log scale). vsRNAs from leg muscle of an *IFNαβR+/+; PVR+/+* (**4B**; 454-163), or *IFNαβR−/−; PVR+/+* (**4C**; Sol-1) mouse infected with poliovirus (4 d.p.i; 5′-Phosphate-dependent capture). vsRNAs with a 5′ monophosphate from the spleen of an *IFNαβR+/+* (**4D**; 454-131), or *IFNαβR−/−* (**4E**; 454-143) mouse infected with West Nile Virus (3 d.p.i).

To identify virus-host systems in which RNAi might participate as an antiviral defense mechanism, we sequenced small RNAs from diverse populations of cells (of human or mouse origin) infected with one of five viruses: Vesicular Stomatitis Virus (VSV), Poliovirus, West Nile Virus (WNV), Dengue Virus, or Hepatitis C Virus (HCV). These viruses were purposefully chosen as token members of diverse families (**Fig. 1**), and are mostly positive-stranded (except for Vesicular Stomatitis Virus, which is negative-stranded). We identified vsRNAs from all six surveyed viruses (**Fig. 1** **; Table S3**), albeit in only a fraction of all infected samples investigated. From this initial survey, we made a choice of a single host-virus system in which to further investigate vsRNA biogenesis. The viral system chosen for this purpose was HCV infection of human Hepatoma cells. While the remainder of the *Results* section will focus primarily on HCV, we will briefly summarize our observations in the four other virus systems. For the Polio, VSV, West Nile and Dengue (**Fig. S3**) systems (**Table S3**), the abundance and molecular features of vsRNAs were dependent on the nature of the host and/or the virus, with some notable trends:

- vsRNA abundance was generally low (for samples in which v/miR was greater than 0, median v/miR = 0.012; **Table S3**).
- vsRNA strand ratios (Positive∶Negative) were divergent from the strand ratios observed for full-length viral RNAs. For Polio, VSV, and West Nile, experimentally-determined strand ratios of full-length viral RNAs in infected cells range from 10∶1 to >100∶1 (Positive∶Negative; [50],[51],[52],[53],[54],[55],[56]). Each of these viruses showed a more equivalent vsRNA strand ratio, particularly seen in 5′P-dependent capture. Two VSV-infected, one WNV-infected and ten Poliovirus-infected samples each demonstrated a Positive∶Negative ratio of <5∶1 (**Table S3**). The substantially less skewed +/− vsRNA strand balance argues against a major fraction of vsRNAs deriving from simple random degradation of viral long RNA pools.
- In the absence of Dicer, the observed vsRNA abundance in MEFs only dropped about 2.1-fold (relative to all sequences; *P*-value = 1.3E-106; **Table S3**), while the miRNA abundance dropped by over 100-fold. Relative to miRNA counts, the vsRNA abundance increased 175-fold in the *dcr-1−/−* MEFs (*P*-value = 0), indicating that unlike miRNAs, there were substantial populations of vsRNAs that did not require Dcr-1 for their biogenesis.
- In the absence of host Argonaute-2 (tested for VSV and Polio in MEFs; **Fig. 3** **, Fig. S4, Fig. S5;** cell lines described in [11]), the population of vsRNAs may have increased relative to miRNAs [*P*-values: 1.7E-06 (VSV; 4.4-fold increase), 8.7E-89 (Polio; >8-fold increase)]. This cannot be attributed to increased viral load, as there is no significant change in the levels of Poliovirus (**Fig. S6**), or in VSV full-length RNAs (**Courtney Wilkins, Marie Chow, personal communication**) between *ago-2−/−* and *ago-2+/+* cells. One intriguing possibility is that the increased vsRNA abundance could reflect a consequence of enhanced vsRNA duplex stability in the absence of unwinding or “recycling” by Argonaute-2.
- In the absence of a functional IFN-α/β receptor in the host (tested for WNV and Polio; **Fig. 4**), vsRNAs were more abundant relative to miRNAs [*P*-values: 5.8E-25 (WNV; >30-fold), 0.021 (Polio; 1.7- to 5.5-fold)].
- In addition to the production of vsRNAs, viral infection may be expected to lead to perturbations in levels of endogenous small RNAs (e.g. miRNAs). Although a much more extensive experimental dataset will be required for definitive assessment of individual miRNA changes, several changes in miRNA patterns that were consistently observed in diverse infection conditions illustrate the potential for host miRNA influences during viral infection (**Fig. S7**). One example of this comes in examining *miR-21* during WNV infection. *miR-21* increases significantly after WNV infection in spleen and macrophages from WT mice, in spleen, macrophages and dendritic cells from *IFNαβR−/−* mice, and in macrophages and dendritic cells from PKR*−/−RNaseL−/−* mice (**Fig. S7**).

A more detailed description of small RNA profiles from West Nile Virus, Dengue, Vesicular Stomatitis Virus, and Poliovirus is provided in the supporting document **(Text S1)**, and in **Supplementary Tables & Figures**.

### Infectious and replicon models of HCV infection yield a signature for vsRNAs

HCV is an enveloped, positive-stranded RNA virus that is a member of the *Flaviviridae* family. Its genome is flanked by short stretches of structured RNA in the 5′ and 3′ UTRs, is uncapped, and lacks a 3′ poly-A tail. A previous study with HCV-1b-infected Huh7.5 cells failed to identify HCV-derived vsRNAs using standard sequencing protocols [57]. We expanded on this work by choosing two cell-culture-based systems that are used for studying HCV replication: an HCC cell line (Huh7) harboring a subgenomic replicon of genotype 1b [55], and an infectious virion system (Huh7.5 cells infected with tissue-culture-produced virions of genotype 2a [58]).

v/miR levels of 5′-P vsRNAs from replicon cells varied between 0.03 and 0.14 (**Fig. S8, Table S3**), with an estimated 7300 +/− 2200 vsRNA molecules per cell (based on the approximation that the most abundant miRNA, miR-122a, is present at 15,000 copies per hepatoma cell in culture [59]). In virus-infected Huh7.5 cells, we detected a very low incidence of vsRNAs at early time points, with an increase over time (**Fig. S9, S10**). vsRNAs were found starting at 1 day post-infection (dpi) in Huh7.5 cells (v/miR = 0.000025), and steadily increased (excluding a possible dip at 11dpi), reaching a v/miR value of 0.056 at 15 dpi.

**Figure 5:**
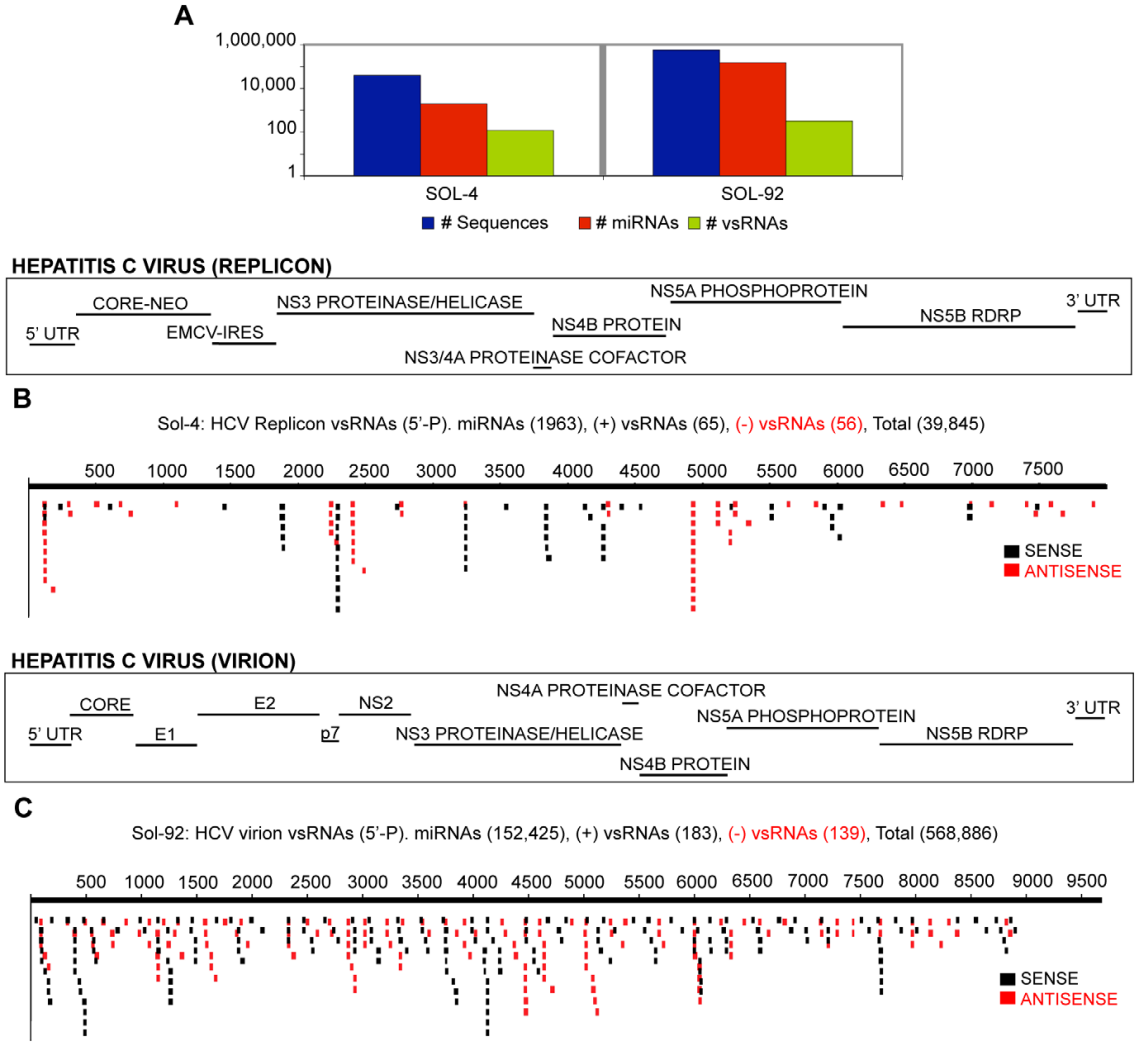
vsRNAs are detectable in different models of Hepatitis C Virus infection. (**5A**) Sequence count: all RNAs, miRNAs, vsRNAs (Y-axis: log scale). vsRNAs from: (**5B**) Huh7 cells with HCV replicon (Sample: Sol-4); (**5C**) Huh7.5 cells infected with HCV virions, harvested 3 d.p.i (Sample: Sol-92).

vsRNAs from the sense (positive) and antisense (negative) viral strands were roughly equally abundant in both the replicon and the infectious virion systems (sense-to-antisense ratios of 1.07 to 1.9; **Fig. 5** **, Table S3**). This contrasts with the observed ratios of genome-length viral RNAs, where the sense strand is 5- to 10-fold more abundant in replicon-harboring cells and in infected hepatocytes [53],[54],[55]. The near-equivalent abundance of vsRNA strands is consistent with vsRNAs deriving from cleavage of a double-stranded replication intermediate, which has an equimolar ratio of positive and negative strands.

HCV vsRNAs from both the 1b and 2a genotypes were distributed throughout the length of the genome, with several ‘hotspots,’ where many vsRNAs were found clustered in specific regions of the genome (**Fig. 5**). Direct comparison of vsRNA distributions, and of individual vsRNA hotspot species between the replicates demonstrated that both were reproducible properties of HCV infection (**Fig. S11A–D**). Additionally, the ability of structured sequences such as those found in the HCV IRES and EMCV IRES to produce specific small RNA populations is of considerable interest. A comparison of vsRNA localization and published secondary structures of the HCV IRES and EMCV IRES is shown in **Fig. S12 & S13**.

We also found some evidence for nucleotide bias among HCV replicon (HCVrep)-derived positive-strand and negative-strand vsRNA populations, including a bias toward strings of Cs and Gs at the 5′ and 3′ termini respectively (**Fig. S14**). This potentially creates favorable conditions either for intramolecular base pairing within a vsRNA, or for base pairing between overlapping sense-antisense vsRNA pairs at their termini.

**Figure 6:**
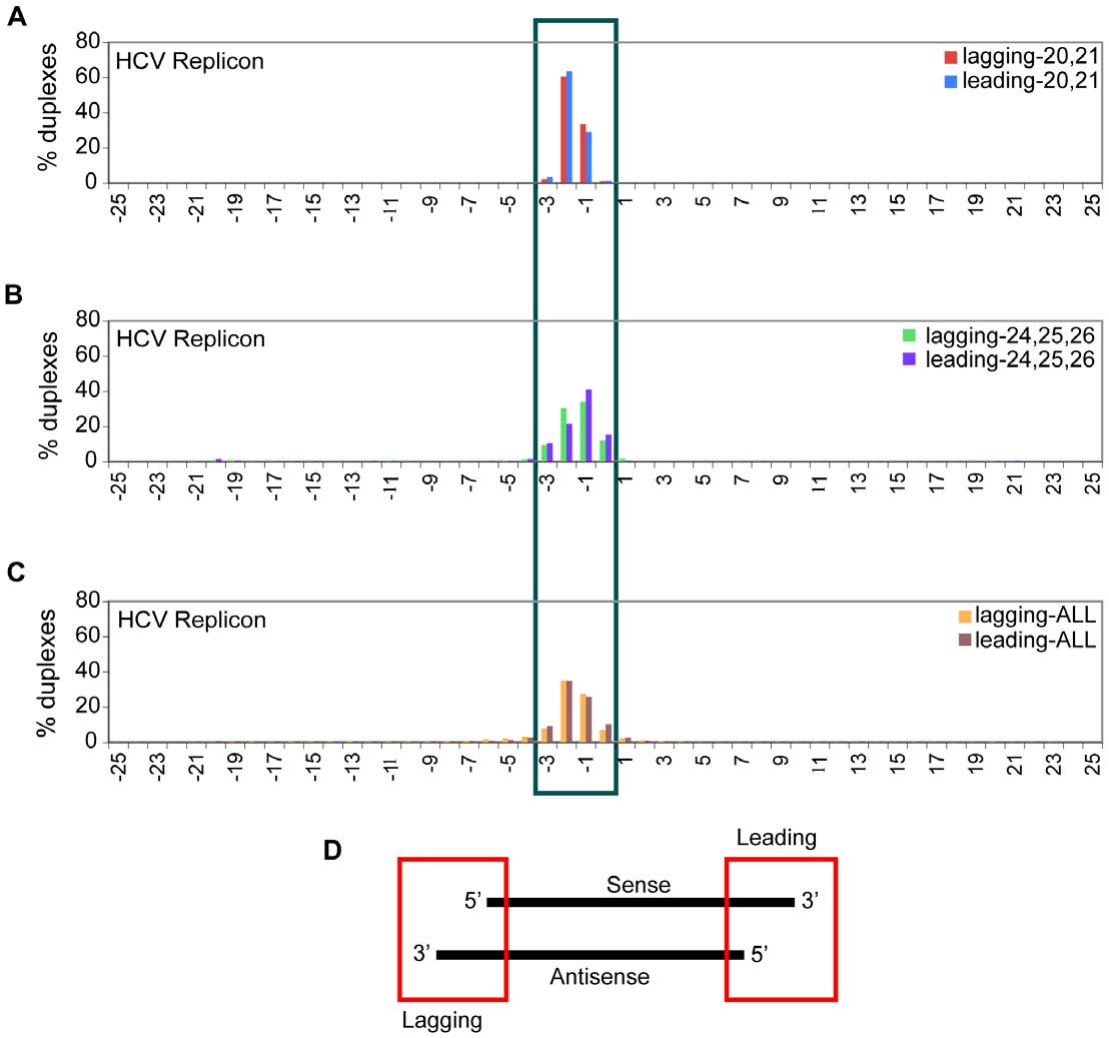
Sub-populations of sense and antisense vsRNAs exist in duplexes with canonical 1–2 nt 3′ overhangs. The assumption inherent in this analysis is that both passenger and guide strands of an siRNA duplex are accessible for capture. All sense (positive strand) and antisense (negative strand) vsRNAs were considered potential partners for this analysis. *X-axis*: range of overhangs (+24 to −24); *Y-axis*: percent of duplexes that fall into each overhang category. Overhangs formed from overlapping sets of HCVrep-derived vsRNAs (Sol-176) after size segregation, represented as a percent of total number of overlapping instances in the +24 to −24 bp window: (**6A**) 20,21-mer vsRNAs; (**6B**) 24,25,26-mers; (**6C**) all size-classes of vsRNAs. (**6D**) Lagging overhangs are computed as: End position of antisense vsRNA – Start position of sense vsRNA; Leading overhangs are computed as: Start position of antisense vsRNA – End position of sense vsRNA. Thus, a 2 base 3′ overhang will have a value of −2, while a two base 5′ overhang will have a value of +2.

We further investigated the potential for duplexed structures of sense and antisense vsRNAs from HCVrep, by comparing sequence placement for sense and antisense vsRNAs within the viral genome. There are examples of independently captured sense and antisense HCVrep-derived vsRNAs that could derive from a dsRNA duplex with a 0–3 base 3′ overhang (**Fig. 6A–C**). These are similar to canonical overhangs in Dicer-generated siRNAs. If we first separated HCVrep-derived vsRNAs into different size ranges and then calculated the distribution of overhangs, duplexes formed by overlapping sets of 20–21 nt sense and antisense vsRNAs had a strong bias for one or two nt 3′ overhangs (**Fig. 6A**). On the other hand, duplexes formed by vsRNAs that are 24–26 nt long have a wider overhang range of zero to three nucleotides (**Fig. 6B**; False Discovery Rate is less than 0.01%).

### vsRNAs associate with Argonaute proteins

**Figure 7:**
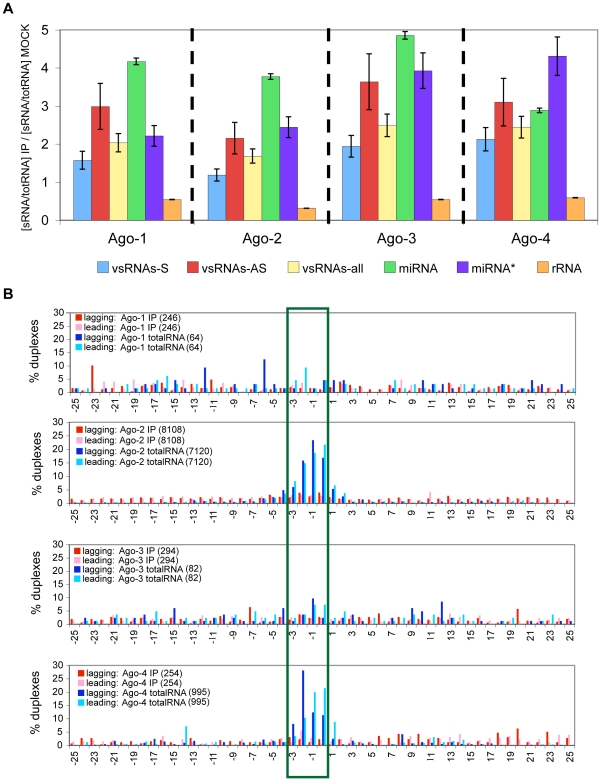
Only one strand of the vsRNA duplex is incorporated into Argonaute complexes. (**7A**) Percent enrichment for various RNAs in Ago-IPs, compared to Mock-IPs, computed as: [(xRNA/totSeq)_IP_/(xRNA/totSeq)_MockIP_]; xRNA = vsRNA, miRNA, miRNA*, or rRNA; totSeq = total number of sequences. The number of vsRNAs varied from 86 to 2472, and the number of total sequences varied from 126,022 to 2,147,467 in these samples. Fractionation of any specific RNA with Argonaute-bound complexes is evidenced in this analysis by retention of representation (compared to miRNAs) and enrichment (beyond that observed for rRNA-derived segments) in the immunoprecipitated pool. (**7B**) Comparison between leading and lagging overhangs formed by HCVrep-derived vsRNAs that either associate with an Argonaute (IP), or are present in cell lysates (totalRNA). All detected sense (positive strand) and antisense (negative strand) vsRNAs were considered potential partners for this analysis.

To explore the possibility that vsRNAs may associate with core components of the RISC machinery (the Argonaute, or “Ago” proteins) despite our inability to detect a role for vsRNAs in silencing pathways, we used transient transfection to express FLAG/HA-tagged Ago-1, Ago-2, Ago-3 or Ago-4 [12] in HCVrep cell lines. We note a limitation of the Argonaute immunoprecipitation (IP) assays in that a large fraction of small RNAs from the cell may be capable of associating with Argonautes in a specific or non-specific manner; nonetheless, the expectation of such experiments is that immunoprecipitation will lead to enrichment for small RNAs that specifically associate with the tagged Argonaute. We compared RNA populations from each of the four Argonaute IPs to RNAs from the Mock-IP (i.e. IP with FLAG Ab, using lysates from mock-transfected cells), to give us an indication of the specificity of the IPs, and conversely, of the degree of non-specificity due to “stickiness” of the α-FLAG-M2 Antibody (**Fig. 7A**). Specifically, we compared the enrichment for vsRNAs in the Ago IPs (relative to Mock IPs), first to the enrichment for RNAs previously known to be Ago-associated (miRNAs, some miRNA*s) [12],[60],[61],[62], and second to the de-enrichment for RNAs which have less (or no) specific association with Argonaute (ribosomal RNAs). In the Ago IPs, we observed a several-fold enrichment for vsRNAs (similar to that observed for miRNAs), accompanied by a marked de-enrichment for rRNA fragments (**Fig. 7A**; for raw data, see **Fig. S15B–C**). This indicates that at least a subpopulation of vsRNAs associates specifically with all four Argonautes.

A striking feature of Ago association in general is the rapid reduction of the initial dsRNA duplex to a single-stranded guide RNA [63]. We compared the duplex properties of vsRNA populations in total cell lysates to those of vsRNAs that are specifically associated with the Argonautes (**Fig. 7B**). The IP datasets for Ago-2 and Ago-4 showed a notable feature: a striking de-enrichment for duplexes with 0–3 nt 3′ overhangs, compared to their respective total RNA samples (*P*-values of 0 and 2.1E-49 respectively). Ago-1 IP and Ago-3 IP showed a de-enrichment for such duplexes, but total RNA samples for Ago-1 and Ago-3 did not have sufficient sequence coverage to allow for a comparison. These data suggest that HCVrep cell lysates have populations of duplexed (and some single-stranded) vsRNAs, with only a single strand of each duplex reproducibly incorporated into an Ago complex.

## Discussion

We were interested in understanding the role played by the RNAi machinery in shaping the course of viral pathogenesis in vertebrate and invertebrate host systems. Our work builds on prior observations that effective replication by certain RNA viruses in plants, *C. elegans* and in *D. melanogaster* requires suppression of the antiviral RNAi response [15],[16],[64]. In each of these invertebrate systems, there is strong evidence for an antiviral mechanism that is directed by small RNAs derived from the virus genome (‘vsRNAs’). Similar questions of great interest in mammals remain unresolved. Using a sequencing approach to investigate the involvement of small RNA-based responses in viral infection, we detected vsRNAs from several mammalian host-viral systems.

### vsRNAs have specific characteristics that distinguish them from RNAs generated by non-specific degradation of viral full-length RNA

We consider two possible sources for the vsRNA populations that were observed during infection: (i) the vsRNAs could be participants in a specific pathway (or pathways) in which small RNAs are generated from the viral genome for host or viral functions; and (ii) the vsRNAs could be products of non-specific degradation of longer (e.g. full-length or subgenomic) viral RNAs mediated by ssRNA nucleases, chemicals, pH, mechanical shear etc.

We note that the small RNA populations characterized by sequencing may be a mixture of (i) biologically relevant small RNAs, and (ii) degradation products with limited significance. In particular, any population of larger RNAs, on extraction and experimental manipulation, can yield a sub-population of RNAs in every size range, including the miRNA and siRNA size range of 19–30 nt. Since viral genomic RNAs and mRNAs are abundant in infected cells, we would certainly expect degraded derivatives to contribute to sequenced pools.

Despite the likely capture of some degradation products, there are several strong indications of vsRNA populations that are not simply the result of degradative mechanisms.

#### Strand ratio

For all five vertebrate viruses used in this study, the ratios of full-length genomic RNAs during infection are highly skewed towards the positive strand (**Fig. S6**; [50],[51],[52],[53],[54],[55],[56]). In contrast, we observed conditions for HCV, Polio, VSV, and West Nile Virus in which the Positive∶Negative strand ratio among vsRNAs was not too different from 1∶1 (**Fig. 1** **, Table S3**). These comparable levels of positive strand and negative strand vsRNAs are not consistent with simple random degradation of longer viral RNAs; rather the observed ratios are consistent with a mechanism that involves processing of dsRNA products by a dsRNA-specific endonuclease. Alternatively, the skew in +/− ratios may be due to (hypothetical) differential accessibility of the positive and negative full-length strands to nuclease digestion, with the negative strand being more accessible despite being less abundant. For HCV and Polio, other results, including our ability to detect specific strand pairing in the vsRNA population (see below) argues against the latter hypothesis. For VSV, the known fact that the both strands are near-equivalently inaccessible due to association with the Nucleocapsid protein (for review, see [65],[66]) argues against the latter hypothesis.

#### Strand pairing

A prominent feature of siRNA duplexes generated by Dicer (an RNaseIII family member) is the presence of approximately two unpaired nucleotides at the 3′ termini of either strand [67],[68],[69]. We see evidence for such duplexes from the total pool of vsRNAs in HCV and polio infections (**Fig. 6** **; Fig. S16I–S16T**), suggesting that a fraction of the detected vsRNAs may be generated by Dicer-like nucleases. Interestingly, a similar anatomy is also required for association of siRNAs with ‘RISC,’ the RNA-induced silencing complex [63],[70],[71],[72]. In contrast, for VSV, we see very distinct hotspots for positive-strand and negative-strand vsRNAs (**Fig. 3D–E** **,** **S17**), suggesting that these vsRNAs may be derived from structures in individual full-length viral RNAs, and not from a dsRNA replication intermediate (similar to what was observed by Molnar et. al. in plants infected with a Tombusvirus [73]). This is supported by the observation that for negative-strand viruses like VSV, full-length viral strands of either orientation are rapidly encased into an RNP structure by association with the Nucleocapsid protein, thus substantially inhibiting strand-pairing of positive and negative full-length viral RNAs (for review, see [65]).

#### Argonaute association

We have evidence that vsRNAs (similar to miRNAs; **Fig. 7A**) specifically associate with four members of a family of proteins called Argonautes, which are the core components of RISC [11],[12]. Compared to total vsRNA populations, we see evidence for de-enrichment of populations in putative double-stranded siRNA structures (i.e. a de-enrichment for overlapping sets of sense and antisense vsRNAs) in the Argonaute complexes (**Fig. 7B**). This is consistent with a model (for review, see [74]) wherein populations of vsRNAs diced from dsRNA substrates persist as duplexes in cellular compartments, with only one strand of the duplex stably maintained by Argonaute complexes.

### Is engagement of the antiviral arm of RNAi in mammals conditional?

The above characteristics of vsRNA populations strongly argue that mammalian cells retain the ability (present in lower organisms; for review, see [16],[17]) to utilize vsRNAs as RNAi effectors. As for any host-virus interaction, the expectation would be that the utilization of small RNA-based mechanisms would be highly dependent on the biology of the virus and the host. This is evident from observed differences in the strandedness of vsRNAs in different systems infected with the same virus, and may be accounted for by several factors such as (a) substantial contributions from potential degradation mechanisms; (b) nuclease activity on ssRNA templates, especially if the secondary structures in one strand are targeted preferentially; (c) strand accessibility; (d) Dicer processivity and activity, and (e) selective RISC loading. Some infectious systems (e.g. Poliovirus or VSV in HeLa cells; **Fig. S18B, S17C**) yield a vsRNA profile with a skew towards positive strand vsRNAs. By contrast, productive infections with the same viruses in other host environments (e.g. Poliovirus in mouse muscle (**Fig. 4C** **,** **S18H**) or VSV in MEF/BHK cells (**Fig. 3D–E** **,** **S17B**) can show strong signatures from both strands, consistent with potential generation by dsRNA-related mechanisms.

Host-virus interactions may also determine bulk abundance of vsRNA populations. In the systems we surveyed, vsRNA abundance varied between 0 and 12.8 (v/miR; relative to miRNAs), or between 0 and 0.02 (v/sRNA; relative to all small RNAs; **Table S3**). Even within a single host, tissue specific factors seem to govern the efficacies of small RNA-related mechanisms. This was observed in *IFNαβR−/−* mice infected with West Nile Virus: even though levels of full-length viral RNA in the brain were comparable to levels in spleen & lymph nodes [5],[50], vsRNAs in the brain were undetectable (454-141, 454-144; **Table S3**).

Despite the complex nature of the factors that govern vsRNA abundance, a number of trends are suggested by comparison of vsRNA levels in paired “wild-type” and “mutant” hosts infected with various viruses. In each of the six sets of experiments where we compared vsRNA levels in parallel infections in *IFNαβR*(+/−), or *ago-2*(+/−) hosts, we observed an increase (ranging from 1.7-fold to >30-fold) in vsRNA levels in the infected “mutant” host. For five out of these six comparisons, the variation in vsRNA abundance between “wild-type” and “mutant” hosts is higher than the (maximum) 3-fold difference we observed in technical replicates.

### Features of vsRNAs

#### 5′-P in mammals versus 5′-xP in *C. elegans*

In *C. elegans*, FHV-derived vsRNAs exist as both primary (5′-P) and secondary (5′-xP) populations, with 5′-xP vsRNAs exhibiting more of a bias toward the antisense (or negative) orientation. The overall picture of small RNA-based surveillance that emerges from Flock House Virus infection in *C. elegans* is consistent with vsRNAs and the RNAi machinery participating substantially in cellular response to the challenge posed by FHV replication. In *C. elegans* undergoing triggered RNAi, a small number of ‘primary’ siRNAs with 5′-P structures are formed by initial cleavage of the dsRNA inoculum, with a much larger population of 5′-triphosphosphorylated antisense small RNAs formed by recruitment of cellular RNA-directed RNA polymerases ‘RdRPs’ to targeted mRNAs [49],[75]. The latter population presumably forms the bulk of effector RNAs, consistent with a requirement for the cellular RdRP *rrf-1* both in the production of a substantial ‘secondary’ pool of 5′-xP vsRNAs [46],[49],[75], and in functional immunity [47],[48]. Also consistent with this model is the shift observed in comparing strand ratios from *rde-1−/−* and wild type hosts (**Fig. 2**): *rde-1* is not required for Dicer-mediated production of sense and antisense primary siRNAs, but is required for the production of more abundant antisense secondary siRNAs [44],[49],[75].

In *Drosophila* and mammals, the primary RNAi pathway has been definitively identified as having 5′-monophosphorylated RNAi effectors that are generated by Dicer, and that subsequently associate with RISC [76],[77],[78]. Though RdRPs have been reported in these systems [79],[80],[81], we do not know their contributions to the biogenesis of vsRNAs, or to the amplification of the antiviral RNAi response. Interestingly, we observed a modest increase in vsRNA/miRNA ratio, using a cloning protocol that allows any number of 5′ phosphates (5′-P-IND protocol). This was observed from multiple mammalian samples infected with either HCV or Poliovirus (**Table S3; Fig. S19** for vsRNA strand ratios), and indicates a potential 5′ chemical diversity among vsRNAs.

#### Non-random distribution

Certain positions on the viral genome also reproducibly serve as ‘hotspots’ for vsRNA production. The relatively abundant ‘hotspot’ vsRNAs may be more stable, favored in biosynthesis by sequence-specificity of Dicer, reflective of regional or structural specificity in susceptibility of the replication intermediate or ssRNA structures to the dicing complex, or coincident with pause sites during viral replication. Additionally, in infections with viruses that generate subgenomic mRNAs during infection (Vesicular Stomatitis virus and Flock House Virus), these hotspots may also reflect the molecular ratios of the various mRNA populations that are ‘transcribed’ from full-length genomic viral RNAs. For instance, we observed an increased abundance of vsRNAs from the region of overlap between the full-length RNA1 and the subgenomic RNA3 of Flock House Virus (**Fig. 2**). We also detected fewer VSV-derived vsRNAs (in MEFs infected with VSV) from the less abundant subgenomic transcripts (L and G mRNAs), than from the more abundant mRNAs encoding the N, P and M proteins (**Fig. 3C, 3D**). We note here that the extent of individual contributions from the various factors pertaining to molarity and/or specificity to vsRNA abundance is unknown.

#### Abundant vsRNAs are not derived from miRNA-like precursors

Some DNA viruses, particularly those from the Herpes family co-opt the host's RNAi machinery to process viral hairpin RNAs into miRNAs [31]. Unlike the Herpes family of viruses, the more abundant vsRNAs from the various RNA viruses we investigated do not appear (based on predicted secondary structure) to be derived from miRNA-like precursors. It is conceivable, however, that some of the rare vsRNAs may be derived from miRNA-like precursors, or that miRNA precursors from RNA viruses have non-canonical structures.

### Origins and functionalities of vsRNAs

#### Are vsRNAs derived from the virus or the host?

While majority of vsRNAs (99.97%) shared perfect homology only with the genome of the infecting virus, we detected a small percent of sRNAs (0.033%) that mapped to both the viral and the host genomes, confounding the source of their origin, and making their classification as ‘vsRNAs’ difficult (**Table S3, S4**). This observation raises the intriguing possibility of some vsRNAs with perfect homology to the host genome (or host sRNAs with perfect homology to the viral genome) participating in potential RNAi-based cross-regulation between host and virus.

#### Are vsRNAs abundant enough for biological relevance?

The miRNA population is an aggregate of hundreds of different biological effector RNAs. A diverse range of concentrations has been reported for functional miRNAs. If we compare bulk vsRNA populations to individual miRNAs, vsRNA populations are more abundant than some functional miRNAs, and less abundant than others. By extrapolation, low abundance of vsRNAs does not imply lack of functionality. However, we do stress that we do not yet know what fraction of the vsRNA pool is functional.

#### Can vsRNAs access full-length viral RNA?

The action of Dicer alone (in producing vsRNAs) is not sufficient to halt virus replication, as has been shown in *Drosophila* [82],[83],[84]. For RNAi to be effective in antiviral defense, it is essential that vsRNAs get incorporated into RISC, and that the vsRNA-programmed RISCs find and cleave their targets. From the HCV replicon system, we have evidence for vsRNA-primed Ago complexes (**Fig. 7**). However, we do not know the extent to which vsRNAs complexed with Ago mediate silencing of full-length replicating/translating/quiescent viral RNAs, as viruses may use counter-defenses to protect themselves from being seen by RISC effectors [29].

#### Other roles for vsRNAs?

vsRNAs have been postulated to mediate roles other than viral gene silencing. One report suggests that vsRNAs from Hepatitis Delta Virus ‘HDV’ may be involved in RNA synthesis [33]. From our studies, vsRNAs from VSV overlay tightly with Leader RNAs that are thought to play a role in regulating viral transcription/replication [85],[86],[87]. We have identified multiple populations of vsRNAs in several systems, both of the 5′-P, and the 5′-xP type (**Table S3**), and it is plausible that while some of them may direct the associated Ago complexes to silence host transcripts in a vsRNA-mediated manner, others may participate in pathways distinct from silencing, such as stimulating/regulating the balance of viral transcription and replication.

### Viral modulation of the small RNA machinery

Viruses can hijack the RNAi machinery at various levels: at the level of Dicer, Argonaute, RISC-mediated silencing, or a combination of the above (for review, see [19]). Downregulation of Dicer has an attenuating effect on Hepatitis C Virus replication [88]. Several studies have shown that HCV proteins inhibit Dicer and Argonaute [34],[89],[90]. In these experiments, inhibition is not complete (∼60–70%; [89]), concordant with our ability to detect vsRNA populations with structures consistent with synthesis by Dicer. We also see evidence for loading of vsRNAs onto Ago complexes, indicating that some downstream steps are not entirely impaired.

### Is there cross talk between other antiviral pathways and RNAi?

Key players in the piRNA pathway, Piwi and Aubergine, are required for protection against Drosophila X Virus infections [84]. The PIWI-piRNA pathway produces 26–31 nt RNAs in a Dicer-independent manner, and is mostly active in the germline [91], and in adjacent somatic tissues [92],[93]. We note that animal systems with PIWI proteins (vertebrates), we observe a wide size range for vsRNAs (**Fig. S2**). These diverse size classes, together with our observation that vsRNAs are still present in systems that lack Dicer (Poliovirus infections in *dcr−/−* MEFs), suggest that the Dicer pathway (which is thought to primarily produce RNAs shorter than 27 nt) may not be the only source for these vsRNAs, and that there may some contribution from baseline levels of PIWI proteins (or other novel proteins) in the various systems. We also identified a population of Polio-derived vsRNAs in *dcr-1−/−* and in *eri-1+/+* MEFs that can form duplexes with 9 or 10 bp overhangs (**Fig. S16I, S16L**), which are hallmarks of piRNAs that are formed by a ping-pong mechanism [94],[95]. This observation brings up the question of how the piRNA pathway interfaces with the RNAi pathway during viral infections, and whether in the absence of the RNAi (and perhaps the IFN) machineries, we could uncover a role for the piRNA pathway in antiviral immunity.

Long dsRNAs, such as those found in the viral replication intermediate, primarily induce the non-specific interferon response in mammalian cells. In the absence of the robust IFN pathway, long dsRNA becomes a trigger for the sequence-directed RNAi pathway [96],[97],[98],[99]. Accordingly, we detected a more abundant signature for dsRNA-derived vsRNAs in *IFNαβR−/−* mice (Poliovirus and West Nile Virus; **Table S3**). Conversely, when we stimulated the Interferon pathway by providing an exogenous supply of IFN-α to a culture of HCVrep-harboring cells, we found that concurrent with reduction in full-length genomic RNA (as reported in: [55],[100]), HCV-derived vsRNAs also dropped several fold (**Fig. S20**). Thus vsRNA abundance seems to associate with the strength of the IFN response: the more robust the IFN response, the fewer the number of vsRNAs. The observed boost in vsRNA abundance in IFN knockout conditions could conceivably reflect a number of distinct effects including augmented viral replication in the absence of IFN, and/or specific interactions between IFN stimulation and the RNAi machineries.

### miRNA modulations

We observed consistent effects of viral infection on miRNA profiles in distinct experimental systems (**Fig. S7**). For example:

- *miR-17-5p* levels significantly dropped in Polio, VSV, HCVrep, Dengue and WNV infected systems (with some exceptions). Other members of the *miR-17-92* cluster were also diminished across cell types.
- Abundance of *miR-125b* decreased in all infected models, except in brain and leg muscle of Poliovirus-infected *IFNαβR−/−* mice.
- *miR-21* levels increased in infected immune cells in culture, and in spleens and lymph nodes of infected mice.

Downregulation of the *miR-17-92* cluster (i) in virus-infected cells may be a pro-apoptotic indicator, since an increase in expression of the *miR-17-92* cluster is associated with an inhibition of apoptosis [101],[102],[103]. A decrease in *miR-125b* (ii) is observed post-LPS-stimulation of macrophages, and causes de-repression of *TNF-α* in a sequence-specific manner [104]. We speculate that *miR-125b* may be one of the regulators of the *TNF-α* response during viral infection, and that regulation of *miR-125b* may require an intact IFN response. Increased levels of the anti-apoptotic *miR-21* (iii) were found in memory and effector T cells, compared to naïve T cells [105]. It is tempting to speculate that this upregulation of *miR-21* may be indicative of proliferating immune cells post-recognition of viral antigens.

### Therapeutic potential of vsRNAs

The identification of multiple vsRNAs, some of which are derived from ‘hotspot’ locations in diverse viral genomes may be useful for designing cocktails of siRNAs for therapeutic purposes, and for mapping areas of the viral genome that are more susceptible to RNAi machineries. Much work still remains to be done in designing siRNA duplexes such that the most accessible strand of the virus may be successfully targeted by siRNAs. A major concern is whether RNAi would be effective in combating viruses with fast replication kinetics. Poliovirus [106] and Semliki Forest Virus [107] replication in cultured cells have been effectively attenuated by an exogenous supply of siRNA triggers against the virus. Whether this holds true for clearing infections in whole organisms remains to be tested.

## Materials and Methods

### Ethics statement

Mice used for experimental infections with West Nile Virus were genotyped and bred in the animal facilities of the Washington University School of Medicine, and experiments were performed with approval from, and according to the guidelines of, the Washington University Animal Studies Committee (which is IACUC approved). For infections with Poliovirus, mice that express the human Poliovirus Receptor gene were maintained in BSL-2 animal facilities at Stanford University. The methods for mouse use and care were approved by the Stanford University Administrative Panel on Laboratory Animal Care (APLAC), and are in accordance with the USDA Animal Welfare Act and the Public Health Service Policy on Human Care and Use of Laboratory Animals.

### Host systems and infection conditions

#### Invertebrates

***C. elegans*** **:** Worms of various genotypes (N2, *rde-1*, *rde-4*, *rrf-1*; [43],[46],[108]) with extra-chromosomal copies of hs::FHVRNA1ΔB2 [48] were heat-shocked for 3 hours at 33°C as L4s/young adults and were harvested and frozen in liquid Nitrogen 24 hours post-heat-shock. RNA1 encodes a functional FHV RNA replicase (FHV Protein A), and a second protein (FHV Protein B2) that contributes to viral infectivity by inhibiting the RNAi pathway (by binding dsRNA in bulk [109],[110]). The replicon RNA1ΔB2 lacks the ability to encode B2 protein, and can effectively replicate only in RNAi-deficient genetic backgrounds [48].

#### Mammals

**Dengue Virus:** Dengue virus-2 (DENV-2) 16681 (Accession# M19197.1) was used for all infections. Huh7 (human hepatoma) cells were infected at an MOI = 1, and were harvested at 0, 2.5, 12, and 24 h.p.i. For infection of U937 cells (a human monocytic cell line), virus (MOI = 5) was complexed with the anti-DENV antibody 3H5, before addition to cells. After 2 hours, the medium was replaced, and cells were harvested 2, 15, and 35 h.p.i. Primary monocyte-derived dendritic cells (MDDCs) were infected at an MOI of 2 and harvested 4 and 24 h.p.i [111]. **Hepatitis C Virus:** HCV subgenomic Replicon-harboring cells (RP7 cells) were established by electroporation of viral RNA from a modified I377/NS3-3′UTR replicon of genotype 1b ([55]; for sequence, see accession# AJ242652.1) into Huh7 cell lines, and maintained under G418 selection. For determining the effect of IFN-α on the production of vsRNAs, RP7 cells were treated with low (5U/mL) or high (100 U/mL) IFN-α concentrations for 72 hours pre-harvest. Virions for infections of Huh7.5 cells (a derivative of Huh7) were obtained by transfection of infectious pFL-J6/JFH1 RNA (Accession# AB047639.1) into Huh7.5 cells [58]. These virions were subsequently used to infect Huh7.5 cells, and infected cells were harvested at 1, 3, 5, 6, 9, 11 and 15 d.p.i. Infected cells were split at the 3, 5, 6, 9 and 11 day time-points, with one half of the cells propagated for subsequent time-points, and the other half used for harvesting RNA. **Poliovirus:** The Mahoney strain (Accession# NC_002058.3) was used for all infections. For infection of HeLa cells, an MOI = 5 (harvest points: 2 and 5.5 h.p.i) was used. *PVR+/+; IFNαβR−/−* and *PVR+/+; IFNαβR+/+* mouse embryonic fibroblasts ‘MEFs’ were infected at an MOI = 1. K562 cells in which persistent infection was established and confirmed were thawed and passaged a couple of times before harvest. Infection in *PVR−/−* MEFs (*ago-2+/+*, *ago-2−/−*, *eri-1+/+*, *eri-1−/−*, dcr-1+/+**, dcr-1−/−**) was established by co-transfection of cells in 10-cm dishes with 100ug of DNA plasmid encoding for the full-length Poliovirus 1 genome under the control of a T7 promoter (pGEM-PV1), and 10 ug of a plasmid encoding for T7 promoter protein. *ago(+/−)* MEFs were harvested 5 hours post-transfection, while the other cell lines were harvested 6 hours post-transfection. For infections in mice, 10^6^ PFU of virus was injected into one hind leg of 6-week old male mice (intramuscular inoculation; *PVR+/+; IFNαβR−/−* and *PVR+/+; IFNαβR+/+*). At 4 d.p.i, the brain and the inoculated leg muscle were dissected from both paralyzed and non-paralyzed individuals. **Wild type and Dicer fl/fl mouse embryonic fibroblasts were generated and immortalized through infection with a retrovirus that expresses SV40 large T as previously described *[112]*. *Cells were then re-infected with a retrovirus that expresses Cre recombinase and confers puromycin resistance. Cells were treated with puromycin (2 ug/ml) for 5 days, after which clones were selected by limiting dilution*. **Vesicular Stomatitis Virus:** A replication-competent, GFP-expressing recombinant virus (VSV-GFP) derived from the Indiana strain (Accession#: NC_001560.1) was used for all infections [47]. For infection in MEFs (*ago-2+/+* and *ago-2−/−*), BHK-21, and HeLa cells, an MOI of 5 PFU/cell was used, and all cells were harvested 4 h.p.i. **West Nile Virus:** The NY99-eqhs strain (Accession# AF260967.1) was used for all infections. Primary cultures of Macrophages and Dendritic cells were established from WT C57*BL/6* mice and congenic *PKR−/−; RNaseL−/−*, and *IFNαβR−/−* mice. MEFs and cortical neurons were established from WT C57*BL/6* mice [113],[114]. These were infected with virus at an MOI = 0.01, and harvested 4 and 16 h.p.i. For infections in mice (WT *B6*, *PKR−/−; RNaseL−/−*, and *IFNαβR−/−*), 100 PFU of virus was injected subcutaneously into the footpad, and mice were sacrificed 1 and 3 d.p.i. Spleen, brain and lymph nodes were dissected from these mice [113],[114].

### Sample processing and library preparation

Worms and mouse tissues were flash frozen in liquid nitrogen, powdered and lysed. Cell lines were trypsinized and washed in PBS pre-lysis. The mirVana kit (Ambion) was used for isolation of RNAs shorter than 200 nt from all samples. Different protocols were used to prepare libraries of RNAs with mono-phosphorylated, or with modified 5′ termini. Briefly, in the 5′-P-Dep protocol, RNA was linkered at the 3′ terminus, size-selected, linkered at the 5′ terminus, reverse-transcribed, amplified using ten-nucleotide barcoded PCR primers [115], and sequenced on the Roche/454 GS-20 or the GS-FLX platforms. In one version of the 5′-P-IND protocol (used for all samples sequenced on the GS-20/FLX), the RNA was linkered on the 3′ end, size-selected and reverse-transcribed before addition of the second linker [49]. In the 5′-P-IND protocol for preparing Solexa libraries, RNA was linkered on the 3′ end, dephosphorylated and re-phosphorylated (to replace any multi-phosphate moieties with a monophosphate), linkered on the 5′ end, reverse-transcribed and amplified (**method courtesy of Guoping Gu**). All 5′-P-Dep and 5′-P-IND libraries for Solexa were prepared by introducing four-nucleotide barcodes as part of the 5′ linker, rather than during PCR amplification (**Lui WO, Parameswaran P; unpublished**). Shorter barcodes allowed for the use of non-barcoded primers for amplification, and most importantly, for greater allocation of sequence space to the sequence of interest. After preparation of barcoded libraries, the libraries were pooled together in molar ratios that were proportional to the sequencing depth required from each sample. The presence of a large number of libraries required multiple sequencing runs on the Roche/454 and the Illumina sequencers.

### Data analysis

Sequences obtained from the Roche/454 platform were handled differently from those obtained on the Solexa platform due to different amplicon structures. Sequences from the Roche/454 platform were binned based on their barcodes using Barsort [115], trimmed using perl scripts **(PP)** and aligned using a local copy of the multiple alignment program Blast (word size = 11). Sequences from Illumina's platform were segregated into individual datasets based on perfect barcode match, trimmed to ensure removal of the flanking adapter sequences before analysis, and aligned using Blat (tile size = 11; step size = 5, run on Mac OS X). Alignments were performed to databases of species-specific miRNAs, and of the various viral genomes. The alignments were subsequently parsed to yield unique hits with the highest homology for each matching read. We filtered for matches of >16 nt for Roche/454 sequencing, and of >19 nt for Solexa sequencing.

For comparing incidence of miRNAs across samples, the frequency of miRNAs and standard error were computed as per the following formulae [116]:

- Relative frequency of miRNAx (p^∼^) = 100 * (Count of miRNAx +2)/(Total miRNA count +4)
- Standard Error of p^∼^ for a 95% confidence interval (Std.Error) = Sqrt[((p^∼^ * (100−p^∼^))/(Total miRNA count +4))]
- 95% Confidence Interval = 1.96 * Std.Error

The distribution, length and strandedness of vsRNAs (i.e. RNAs that mapped to the genome of the infecting virus) were plotted as a function of the length of the viral genome. Starts and ends of positive-strand and negative-strand vsRNAs were then compared to generate a matrix of the percent incidence of different types of overhangs. For generating the nucleotide bias, the vsRNAs were aligned at the 5′ termini (or at the 3′ termini), and the nucleotide frequencies were counted at each position ten nucleotides upstream and ten nucleotides from the 5′ end of the vsRNA population (or ten nucleotides downstream and ten nucleotides from the 3′ end of the vsRNA population). These numbers were then fed into Pictogram (**Chris Burge, MIT**; http://genes.mit.edu/pictogram.html), and were normalized to the total numbers of A, C, G and T in the population of cloned vsRNAs, thus circumventing any bias that arose from a skewed ratio of nucleotides inherent to either the cloning process, or to the genome of the virus.

Calculation of *P*-values: For establishing statistical significance, two-tailed *P*-values were calculated using either the *Z*-test for two proportions, or Fisher's Exact test. Fisher's Exact test was used in test cases with small sample or population sizes, while the *Z*-test was used if sample or population sizes were large.

Calculation of False Discovery Rate (FDR): Populations of 20,21-mers and 24,25,26-mer HCV vsRNAs were pooled, and positive strand and negative strand vsRNAs were randomly selected from the pool. 10,000 datasets that mirrored positive strand and negative strand vsRNA abundances from the original 20,21-mer population, and another 10,000 datasets that mirrored vsRNA abundances (of both polarities) from the original 24,25,26-mer population were generated. The percent of randomly generated datasets wherein 1–2 nt 3′overhang duplexes were as abundant as in the original 20,21-mer population, or as depleted as in the 24,25,26-mer population was calculated (for example, FDR of 0.01% indicates that we were unable to detect percent incidence of duplexes similar to what was observed, in at least 10,000 randomly-generated datasets).

### Argonaute immunoprecipitations (adapted from [117])

Cultures of HCVrep cells were grown to ∼80% confluency in 10-cm. plates, and transfected with 20ug each of FLAG/HA-tagged Ago-1, Ago-2, Ago-3 or Ago-4 (codon-optimized), and harvested 24 hours post-transfection. Cells were washed twice with 4mL PBS, and 0.75mL of ISOB/NP40 (10mM Tris pH 7.9, 0.15M NaCl, 1.5mM MgCl_2_, 0.8% NP40, proteinase inhibitor at 1 tablet per 13mL solution) was added to each plate. Cell lysates were vortexed, incubated on ice for 20 minutes, spun at 4°C for 10 min at 13,000 rpm, and the supernatants were transferred to new tubes. 10 uL ‘Fake’ (uncoated) Sepharose 4B beads were washed twice with 1.5ml NET-1 buffer (1×TBS-0.2% Tween), and incubated with supernatants at 4°C for 2 hrs. The supernatants post-‘fake-bead’ IP were subsequently incubated with 10uL of washed Anti-Flag M2 beads [Sigma] at 4°C for 4 hrs. Finally, beads were washed 3× with 500uL NET-1 buffer, and RNA was extracted with lysis buffer (mirVana kit, Ambion). Transfections were performed in duplicate: 1 plate was used as input for IPs, while the other was used for cloning of total small RNA populations. For Mock-IPs, lysates from mock-transfected cells were used as described above.

### Northern analysis

The loading control was a 5.7 kb region of the pGEM plasmid carrying the Poliovirus 1 genome (pGEM-PV1), obtained by digestion of the plasmid with HindIII and AgeI. Equivalent amounts of RNA (as measured using a NanoDrop) from each sample were run on a 1% agarose-formaldehyde gel, and transferred under basic conditions to a Hybond+ membrane. The loading control was visualized by staining the blot with methylene blue. 60-mer DNA probes to specifically detect either the positive strand (AF-PP-350: GTCACCGCTTGTAGAATTGTCATTGCCCTGTTGATGTTCCTTTCTGTTTGAACCTGGCTG & AF-PP-351: TCATCTATGGTTTGCCGATACGTGGTGTTGCTAATCCATGGCACT ACCATAGTACATGAG), or the negative strand (AF-PP-84: TTCACGGGTACGTTC ACTCCTGACAACAACCAGACATCACCTGCCCGCAGGTTCTGCCCG, AF-PP-86: ATTC GGACACCAAAACAAAGCGGTGTACACTGCAGGTTACAAAATTTGCAACTACCACTT) were end-labeled with ^32^P, and were successively used on the same blot (the blot was stripped in between the two hybridizations). The blots were hybridized and washed at 50°C, and exposed using a phosphorimager screen.

## Supporting Information

Text S1 Supporting text containing supplementary results, methods and references
(0.18 MB PDF)

Figure S1 Host-encoded miRNAs with partial homology to HCV (summarized from published data). Human miRNAs that demonstrate a sequence-directed effect on HCV RNA levels are indicated [19],[20],[21]. These miRNAs are encoded in the host genome, and are hence distinct from HCVrep-derived vsRNAs that are viral-encoded.
(0.19 MB PDF)

Figure S2 Frequency-Length profiles for vsRNAs. X-axis: vsRNA lengths; Y-axis: number of vsRNA instances.
(2.09 MB PDF)

Figure S3 Rare Dengue-derived vsRNAs are detectable in certain host backgrounds. (S3A) Sequence count: all RNAs, miRNAs, vsRNAs (Y-axis: log scale). (S3B) vsRNAs with a 5′ monophosphate moiety from Huh7 cells infected with DENV-2, 25 h.p.i. (Sample: 454-75).
(0.23 MB PDF)

Figure S4 RNase-protection confirms that VSV-specific vsRNAs are more abundant in *ago2−/−* MEFs. RNA was isolated from mock-infected and VSV-infected *ago2+/+* or *ago2−/−* MEFs at 4 h.p.i. vsRNAs derived from positive strand viral RNAs were detected by RNase protection using a radiolabeled probe specific to the 5′ end of the VSV-N gene.
(0.69 MB PDF)

Figure S5 (Profiles from GS-20/FLX-sequenced libraries) Poliovirus vsRNAs are more abundant in MEFs deficient in Argonaute-2. (S5A) Sequence count: all RNAs, miRNAs, vsRNAs (Y-axis: log scale). vsRNAs with a 5′-monophosphate moiety from (S5B) *ago-2+/+* MEFs (Sample: 454-193) and (S5C) *ago-2−/−* MEFs (Sample: 454-194) transfected with a plasmid encoding for self-replicating full-length Poliovirus RNA.
(0.29 MB PDF)

Figure S6 Abundance of the two polarities of Poliovirus full-length RNAs varies across systems, with full-length positive strand generally present in excess over full-length negative strand. Visualization of full-length Poliovirus in various samples by Northern analysis: (S6A) Positive strand; (S6B) Negative strand. Normalization control: fragment of plasmid with homology to Poliovirus (see Materials and Methods).
(0.59 MB PDF)

Figure S7 Only a handful of miRNAs are modulated during viral infection. (7A) Log_2_-transformed ratios of specific miRNA abundances between infected and uninfected samples (or between early and late time-points) were represented as a heat map. Only those miRNAs that exhibited a > = 2-fold, and a statistically significant upregulation or downregulation (i.e. no overlap of 95% confidence intervals) were considered. Fold changes were log_2_-transformed. No row-wise or column-wise normalizations were performed. The yellow squares represent miRNAs that are upregulated in infected cells/late time-points compared to uninfected/early time-points. The blue squares represent miRNAs that are downregulated in infected cells/late time-points compared to uninfected/early time-points. Higher intensities correlate with a greater fold change. *Note:* The baseline frequency for each miRNA was set to 0.0001 (rather than 0), to enable computation of ratios. (7B) Clustering of miRNA profiles across various infected and uninfected mouse tissues/cell lines reveals that majority of samples cluster according to tissue type/cell line origin. miRNA frequencies were first normalized against the total number of miRNAs for each sample. Cube-root values of the frequencies were subsequently used in uncentered clustering of the data set, both row-wise and column-wise. These plots were generated using GenePattern [http://www.broad.mit.edu/cancer/software/genepattern/].
(7.91 MB TIF)

Figure S8 Abundance and distribution of HCVrep-derived 5′-P vsRNAs varies between early and late passage cells, but 5′-xP vsRNAs are enriched (relative to 5′-P) in both early and late passages. (S8A) Sequence count: all RNAs, miRNAs, vsRNAs (Y-axis: log scale). vsRNAs from a late passage (later than passage 15) of cells: (S8B) captured using the 5′-P-dependent protocol (Sol-107); (S8C) captured using the 5′-P-INDependent protocol (Sol-109). vsRNAs from an early passage (passage 3) of cells: (S8D) captured using the 5′-P-dependent protocol (Sol-176); (S8E) captured using the 5′-P-INDependent protocol (Sol-179).
(0.34 MB PDF)

Figure S9 Increase in the abundance of HCVvir-derived vsRNAs (relative to miRNAs) at later time-points in infection (Solexa-sequenced libraries). Positive strand vsRNAs are shown as blue bars, and negative strand vsRNAs as red bars. (S9A) Sequence count: all RNAs, miRNAs, vsRNAs (Y-axis: log scale). vsRNAs with 5′ monophosphates from Huh7.5 cells infected with HCV virions: (S9B) 1 d.p.i (Sample: Sol-91); (S9C) 3 d.p.i (Sample Sol-92); (S9D) 6 d.p.i (Sample Sol-93); (S9E) 9 d.p.i (Sample Sol-94); (S9F) 11 d.p.i (Sample Sol-95); (S9G) 15 d.p.i (Sample Sol-96).
(0.37 MB PDF)

Figure S10 Temporal changes in the abundance of HCVvir-derived vsRNAs (GS-20/FLX-sequenced libraries). (S10A) Sequence count: all RNAs, miRNAs, vsRNAs (Y-axis: log scale). vsRNAs with 5′ monophosphates from Huh7.5 cells infected with HCV virions: (S10B) 3 d.p.i (Sample: 454-84); (S10C) 5 d.p.i (Sample: 454-85); (S10D) 11 d.p.i (Sample 454-86).
(0.27 MB PDF)

Figure S11 vsRNA coincidence plots show high degree of similarity between independent amplicon libraries from the same starting sample, and reproducible detection of hotspots. (S11A) Coincidences are defined by vsRNAs of the same orientation, with the same Start and End positions. Percent coincidence between ‘Sample-1’ and ‘Sample-2’ was calculated as follows: (# of coincidences between Sample-1 and Sample-2)/[(# of vsRNAs in Sample-1) + (# of vsRNAs in Sample-2)] * 100. These values are plotted as a heat map, with dark-to-light blue representing low-to-high values. (S11B-D) Pairwise comparison of the relative abundance of individual vsRNAs between independent amplicon libraries of early-passage HCV replicon cells. Each point represents a unique vsRNA, and the X- and Y-axes represent relative abundance (with respect to all vsRNAs) in the indicated amplicon libraries. (S11E) Sequence count: all RNAs, miRNAs, vsRNAs (Y-axis: log scale). vsRNAs with 5′ monophosphates from HCVrep cells: (S11F) Sample: Sol-176; (S11G) Sample Sol-201; (S11H) Sample Sol-203; (S11I) Sample Sol-205. Positive strand vsRNAs are shown as blue bars, and negative strand vsRNAs as red bars.
(0.70 MB PDF)

Figure S12 A map of vsRNA start and end positions, superimposed on the secondary structure of the HCV IRES (see [19] for source of IRES structure). Only positions that had an incidence of > = 2 Starts/Ends were mapped.
(0.32 MB PDF)

Figure S13 A map of vsRNA start and end positions, superimposed on the secondary structure of the EMCV IRES (which is part of the HCV Replicon genome; see [20] for source of IRES structure). Only positions that had an incidence of > = 2 starts/ends were mapped.
(0.42 MB PDF)

Figure S14 vsRNAs from cells with the HCV Replicon (Sol-4) exhibit a bias for Cytidines at their 5′ termini, and a bias for Guanines at their 3′ termini. vsRNAs were first aligned at their 5′ ends (or at their 3′ ends), and the nucleotide frequencies for 10 upstream (or 10 downstream) positions, plus for the first 10 (or last 10) vsRNA base positions were determined separately for positive strand and negative strand vsRNAs. This analysis was performed on all vsRNA instances, or post-collapse of the vsRNA dataset (‘unique instances’), such that multiple instances of vsRNAs were represented only once. Arrows indicate Start/End positions of vsRNAs. Nucleotide frequencies have been normalized to the overall nucleotide composition within each dataset. (S14A–S14D) Pictogram of 10 bases upstream+10 bases (from 5′ termini) of vsRNAs derived from (S14A) Positive strand: unique instances; (S14B) Negative strand: unique instances; (S14C) Positive strand: all instances; (S14D) Negative strand: all instances. (S14E–S14H) Pictogram of 10 bases downstream+10 bases (from 3′ termini) of vsRNAs derived from (S14E) Positive strand: unique instances; (S14F) Negative strand: unique instances; (S14G) Positive strand: all instances; (S14H) Negative strand: all instances.
(0.67 MB PDF)

Figure S15 Mock-IP enriches for rRNAs. (S15A) [(xRNA/totSeq)_MockIP_/(xRNA/totSeq)_totalRNA_] was computed for the various IPs; xRNA: vsRNA, miRNA, miRNA*, or rRNA; totSeq = total number of sequences from each experiment. The number of vsRNAs varied from 86 to 1,857, and the number of total sequences varied from 126,022 to 891,858 in these samples. (S15B–C) Raw data: [(xRNA/totSeq)] for the various IPs (including the mock IP), and for the totalRNA populations; xRNA: vsRNA, miRNA, miRNA*, or rRNA; totSeq: total number of sequences from each experiment.
(0.25 MB PDF)

Figure S16 Start-to-Start, Start-to-End, and duplex overhang plots for positive strand and negative strand vsRNAs show differences between Poliovirus-infected hosts with wild-type or mutant copies of dcr-1 and eri-1. Y-axis: percent of vsRNA pairs with specified Start-to-Start or Start-to-End distances on a scale of +120 to −120. (S16A, S16B, S16E, S16F) Start-to-End distances for positive strand and negative strand vsRNAs cloned from *dcr-1+/+*, *dcr-1−/−*, *eri-1+/+*, *eri-1−/−* MEFs; (S16C, S16D, S16G, S16H) Start-to-Start distances for positive strand and negative strand vsRNAs cloned from *dcr-1+/+*, *dcr-1−/−*, *eri-1+/+*, *eri-1−/−* MEFs. Predicted overhangs formed by overlapping sets of sense and antisense 20 and 21 nt vsRNAs sequenced from: (S16I) *eri-1+/+* MEFs +Poliovirus; (S16J) *eri-1−/−* MEFs +Poliovirus; (S16K) *dcr-1+/+* MEFs +Poliovirus; (S16L) *dcr-1−/−* MEFs +Poliovirus. (S16M-P) Overhangs formed by 24,25,26-mers from systems listed above. (S16Q-T) Overhangs formed by all size-classes of vsRNAs from systems listed above. All captured vsRNAs of either polarity were considered to be potential partners for this analysis.
(0.50 MB PDF)

Figure S17 Abundance, distribution and orientation biases of VSV-derived vsRNAs are a function of cell-type. (S17A) Sequence count: all RNAs, miRNAs, vsRNAs (Y-axis: log scale). vsRNAs with 5′ monophosphates from Vesicular Stomatitis virus infections in: (S17B) BHK-21 cells, harvested 4 h.p.i (Sample: 454-179); (S17C) HeLa cells, harvested 4 h.p.i (Sample: 454-181).
(0.28 MB PDF)

Figure S18 vsRNAs are present in both lytic and persistent models of Poliovirus infection, and can be captured using both the 5′-P-dependent, and the 5′-P-INDependent cloning protocols. (S18A) Sequence count: all RNAs, miRNAs, vsRNAs (Y-axis: log scale). vsRNAs with a 5′ monophosphate from: (S18B) HeLa cells, that are prone to lysis upon infection with the Mahoney strain of poliovirus (MOI = 5, 5.5 h.p.i, Sample: Sol-1); (S18C) K562 cells, in which Poliovirus establishes a persistent infection (Sample: 454-49); (S18D) brain of 6wk-old paralyzed Poliovirus-infected *IFNαβR−/−; PVR+/+* male mouse, 4 d.p.i. (Sol-81). vsRNAs captured using the 5′-Phosphate-INDependent cloning protocol, from: (S18E) K562 cells infected with Poliovirus (Sample: 454-50); (S18F) *ago-2+/+* MEFs +Poliovirus (Sample: Sol-79)*; (S18G) *ago-2−/−* MEFs +Poliovirus (Sample: Sol-80)*; leg muscle (S18H) and brain (S18I) of a 6wk-old male mouse (genotype: *IFNαβR−/−; PVR+/+*) infected with Poliovirus, 4 d.p.i. *These MEFs were transfected with a plasmid encoding for self-replicating Poliovirus RNA, and harvested 5 hours post-transfection.
(0.35 MB PDF)

Figure S19 The 5′-P-INDependent cloning protocol subtly enriches for antisense vsRNAs. Comparison of antisense vsRNAs (as a % of all vsRNAs; S19A) and of sense vsRNAs (as a % of all vsRNAs; S19B), between the 5′-P-dependent and the 5′-P-INDependent cloning protocols.
(0.23 MB PDF)

Figure S20 IFN responsiveness reduces the incidence of HCVrep-derived vsRNAs. (S20A) Sequence count: all RNAs, miRNAs, vsRNAs (Y-axis: log scale). vsRNA profiles from: (S20B) untreated HCVrep cells; (S20C) HCVrep cells treated with 5 U/mL IFN, and harvested 72 hours post-treatment; (S20D) HCVrep cells treated with 100 U/mL IFN, and harvested 72 hours post-treatment.
(0.27 MB PDF)

Table S1 Reference genomes of all viruses used.
(0.04 MB PDF)

Table S2 Most abundant vsRNAs. The five most abundant vsRNAs captured in infections with FHV, VSV, Polio, HCVrep and HCVvir are listed. Start position, length, orientation and sequence of these vsRNAs are indicated, as are the number of datasets in which they were identified, and their respective total and normalized counts. vsRNAs were first defined by start position, length and orientation. All defined vsRNAs were then ranked based on (a) number of samples in which they were identified, and (b) their total count across all samples, normalized to the total vsRNA count (or to the total miRNA count) for those samples. For West Nile and Dengue Viruses, because of poor coverage, only four and two vsRNAs (respectively) are shown.
(0.07 MB PDF)

Table S3 Comprehensive list of virus-host systems that were surveyed, with sample descriptions, bulk frequencies and ratios of various classes of small RNAs, and frequencies of ‘rogue’ viral and host genomic matches to vsRNAs. Samples are ordered first by sequencing platform, second by type of virus used in the infection, third by genotype, fourth by cell/tissue type, and fifth by time-point.
(0.41 MB PDF)

Table S4 List of vsRNAs that map perfectly to *both* host and viral genomes. Due to the complete nature of sRNA homology to both viral and host genomes, the origin of the listed vsRNAs cannot be determined. These numbers are also indicated in Table S3.
(0.06 MB PDF)
