# Supplementary material for: Six RNA Viruses and Forty-One Hosts: Viral Small RNAs and Modulation of Small RNA Repertoires in Vertebrate and Invertebrate Systems
Source: PLoS Pathog. 2010 Feb 12;6(2):e1000764. doi: 10.1371/journal.ppat.1000764 (PMC2820531; doi:10.1371/journal.ppat.1000764)
Supplement: Text S1 — Supporting text containing supplementary results, methods and references (0.18 MB PDF) [file ppat.1000764.s001.pdf]

vsRNAs are present in infections with a negative-stranded virus, Vesicular Stomatitis Virus (VSV):

VSV is the prototypical negative-stranded, enveloped virus of the *Vesiculovirus* genus in the *Rhabdoviridae* family. We chose to study VSV-GFP infections in three different host cells: HeLa, MEFs, and BHK-21. Although VSV productively infects each of these host cells, virus growth kinetics and titers vary between cell types. Virus growth is slowest (with lowest titers) in HeLa cells, intermediate for growth (and titers) in MEFs, and most rapid (with highest titers) in BHK-21 cells. In addition we studied the effects of RNAi on VSV-derived vsRNA profiles by comparing virus infections in RNAi-sufficient (*ago-2+/+*) and RNAi-deficient MEFs (*ago-2-/-;* MEFs described in [1]).

VSV-derived vsRNAs were detected in all cell types. HeLa cells had the lowest abundance of vsRNAs (v/miR = 0.003; **Fig. S17C**), while BHK-21 cells had 4.5-fold more vsRNAs (**Fig. S17B**; v/miR = 0.01). The *ago-2-/-* RNAi-deficient MEFs had the highest abundance (**Fig. 3E**; v/miR = 0.02), but vsRNA levels were much reduced in the *ago-2+/+* background (**Fig. 3D**; v/miR = 0.004). This differential vsRNA abundance in MEFs observed by sequencing is also supported by RNase protection assays that show a specific increase in the abundance of N gene-derived positive-strand vsRNAs in *ago-2-/-* MEFs **(Fig. S4)**, compared to *ago-2+/+* MEFs. The increased abundance of vsRNAs in *ago-2-/-* MEFs is not merely due to an increase in copy number of longer viral RNAs, as the viral RNA load does not significantly change between the *ago+/+* and the *ago-2-/-* cells at that time-point (**Courtney Wilkins, Marie Chow, personal communication**). We speculate that in the absence of Argonaute-2, the vsRNAs (generated by nucleases such as Dicer, which is still functional in these cells) are not unwound or "recycled," leading to an effective increase in their stability, and consequently, their

abundance. This trend in vsRNA abundance (in *ago-2-/-* vs. *ago-2+/+* MEFs), together with the commonality in hotspots observed in RNAi-competent and RNAi-deficient cells (**Fig. 3**), bear resemblance to what we observed in Poliovirus infections. Strand ratios also varied between host cell-types. vsRNA strand ratios (Positive:Negative) were close to 1:1 in BHK-21, and > 5:1 in the MEFs (**Table S3**). Notably, all the captured vsRNAs were from the positive strand in HeLa cells. vsRNAs of both positive and negative polarities appear to be preferentially localized to discrete regions distributed along the viral genome in infected MEF (**Fig. 3D-E**) and BHK-21 (**Fig. S17B**), but not in HeLa cells (**Fig. S17C**). In MEFs and BHK-21 cells, positive strand vsRNAs aggregate towards the 5' end of the positive strand, while the negative strand vsRNAs are derived primarily from the 5' end of the negative strand. The terminally positioned vsRNAs originate from regions encoding previously characterized Leader RNAs that have been proposed to play a role in VSV replication [2,3,4].

Host genotype and infection-type affect profiles of Poliovirus-derived vsRNAs:

Lytic versus Persistent infections: Poliovirus is a positive-stranded non-enveloped *Enterovirus*, belonging to the family *Picornaviridae*. Poliovirus establishes a lytic infection in most differentiated cell lines, including the human cervical carcinoma line HeLa, but establishes a persistent infection in K562 cells, which is a less differentiated cell type [5]. We compared 5'-P vsRNA profiles from poliovirus-established persistent and lytic infections, to look for signatures unique to either outcome. From a pilot experiment with 18,848 sequences from multiplexed Roche/454 sequencing, polio vsRNAs were undetectable (2 h.p.i) or barely detectable (5.5 h.p.i.) in HeLa cells (**Table S3**). With a larger sequence set (Illumina), 5'-P vsRNAs were detected at 5.5 h.p.i (v/miR = 0.006; **Fig. S18B**). vsRNA frequencies were marginally lower in infected

K562 cells, compared to HeLa cells (v/miR = 0.002; **Fig. S18C**). Thus, the presence of Poliovirus-derived vsRNAs (relative to miRNA levels) does not seem to be dependent on a lytic outcome of the infection. Strand ratios were distinct in the two cell types. In HeLa cells, the +/- vsRNA strand ratio was 7.13:1, while the ratio in K562 cells was 3:1 (**Table S3**). These differences may be reflective of differences in the biology of viral replication in the two infection models. Even with an excess of sense vsRNAs in these cell types, these ratios are still quite divergent from the full-length +/- genomic ratios of 10:1 to 70:1 (**Fig. S6**; [6,7]); thus the vsRNA population is not a simple degradative by-product of longer viral RNA populations.

Roles of Argonaute-2, Dicer-1 and Eri-1: Dicer-1 (*dcr-1*) is an RNase Type III endonuclease that 'dices' dsRNA structures into smaller 19-27 nt RNA duplexes called siRNAs (or miRNAs, if the substrate is a host-encoded hairpin RNA). Argonaute-2 (*ago-2*) is the 'slicer' in RISC that mediates cleavage of mRNA targets in a sequence-directed manner (for review on *dcr* and *ago*, see [8]). Enhancer of RNAi-1 (*eri-1*) is a 3'-to-5' exonuclease that associates with Dcr-1 in *C. elegans* [9]. *eri-1* mutant worms show enhanced RNAi responses due to increased half-life of siRNAs [10]. To determine if any or all of these three RNAi pathway components play a role in the biogenesis of Poliovirus-derived vsRNAs, we compared vsRNA profiles in cells with and without *dcr-1*, *ago-2* or *eri-1*.

As expected, the host-derived miRNA populations were much reduced in the *dcr-1-/-* MEFs (11 out of 13,984 sequences, or 64 out of 114,504 sequences; i.e. 0.06-0.08% of all sequences), compared to the *dcr-1+/+* MEFs (7-21% of all sequences; **Table S3**). However, the overall vsRNA abundance only dropped about 2.1-fold (v/sRNA; relative to all sequences) in the *dcr-1-/-* MEFs, compared to the *dcr-1+/+* MEFs (*P*-value = 1.3E-106; **Table S3**). Relative to miRNA counts, the vsRNA abundance increased 174.8-fold in the *dcr-1-/-* MEFs (*P*-value = 0),

indicating that there were substantial populations of vsRNAs that, unlike miRNAs, did not require Dcr-1 for their biogenesis. These Dcr-independent vsRNAs are presumably generated by other cellular nucleases, or by viral or cellular RdRP activity on full-length viral RNA. The +/- strand ratio did not change significantly between the genotypes, and was close to 1.5:1 (**Table S3**).

There was an 8-fold to 30-fold enrichment for 5'-P vsRNAs from the *ago-2-/-* MEFs (**Fig. 3C**; v/miR = 0.06**, Fig. S5C**; v/miR = 0.04), compared to the *ago-2+/+* MEFs (**Fig. 3B)** v/miR = 0.002; *P*-value = 0; (**Fig. S5B**) v/miR = 0.005; *P*-value = 8.7E-89). We note that similar to the VSV system, no increase in Poliovirus full-length (genomic) RNA load was evident in the *ago-2-/-* MEFs (**Fig. S6**), even though there was a substantial increase in the abundance of Poliovirus-derived vsRNAs. Also, Ago-2 had little (if any) effect on strand ratio or hotspot positions for Poliovirus-derived vsRNAs from MEFs. vsRNAs were 4.5-fold more abundant in the *eri-1-/-* cells (**Table S3**; v/miR = 0.1, compared to v/miR of 0.02 in *eri-1+/+* cells; *P*-value = 1.9E-221).

To analyze spatial differences in the vsRNA landscape, we prepared auto-correlation charts, i.e. frequency charts of differences in Start positions (Start-to-Start), and of differences between the Ends and the Starts (Start-to-End) of all vsRNA populations. These plots seem to suggest that both the *eri-1* (**Fig. S16E-H**) and the *dcr-1* (**Fig. S16A-D**) pathways may affect the global spatial distributions of vsRNAs, by determining/restricting Start and/or End positions of some vsRNA subpopulations.

Effects of the IFN pathway: We looked at poliovirus infections in cell culture and in *in-vivo* (mouse) systems that lack the IFN-α/β Receptor (*IFN-$\alpha/\beta R$*) genes. Poliovirus replication and associated pathologies in wild-type "WT" mice (transgenic for the Poliovirus Receptor gene;

PVR+/+) are confined primarily to the CNS and some peripheral tissues (for review, see [11]). In *PVR+/+; IFN-α/βR-/-* mice, there is an expansion in cell types that are targeted by Poliovirus, and the virus replicates robustly in additional organs such as lung, spleen etc. [12]. When we infected three *IFN-α/βR*+/+; *PVR*+/+ and three *IFN-α/βR-/-*; *PVR*+/+ mice, at 4 dpi all the *IFN-α/βR-/-; PVR*+/+ mice were paralyzed, but only some of the WT (*PVR*+/+) mice exhibited paralysis, recapitulating previous observations [12] that poliovirus is more virulent in the *IFN-α/βR-/-* background. We detected vsRNAs in significant abundance only in tissues from *IFN-α/βR-/-* mice (**Table S3**). vsRNAs in leg muscle (**Fig. 4C**) were present at v/miR levels of 0.01, with much lower levels (44.6-fold, v/miR = 0.0003) in the brain (*P*-value = 4.5E-120; **Fig. S18D**). The difference in vsRNA profiles between the two tissues is further highlighted by dissimilarities in the +/- vsRNA strand ratios (1.36:1 for muscle, and 6+:1- for brain; *P*-value = 0.0004; **Table S3**). These ratios are very different from the ratio of full-length positive-stranded to negative-stranded viral RNA, which is between 10:1 and 100:1 (**Fig. S6**). There was a slightly skewed distribution of poliovirus-derived vsRNAs across the poliovirus genome. 41% of positive strand vsRNAs and 47% of negative strand vsRNAs mapped to the 10% of the genome that is the 5' UTR (**Fig. 4C**), suggesting that the replication intermediate formed during positive strand synthesis from the negative strand might be a good candidate for Dicer, or for other nucleases in these systems.

One of the drawbacks of the mouse experiments is that they do not indicate whether the increased abundance of vsRNAs in *IFN-αβR*-/- mice was due to the accelerated kinetics of Poliovirus infection in this background. To control for this parameter, we infected *IFN-αβR*+/+; *PVR*+/+ and *IFN-αβR-/-*; *PVR*+/+ MEFs with Poliovirus (MOI = 1), and allowed for 1 infection cycle to proceed before harvesting the cells (~6 h.p.i). The *IFN-αβR-/-* cells had a higher

abundance of vsRNAs (**Table S3**; v/miR = 0.08, versus 0.05 in *IFN-αβR+/+* cells; *P*-value = 2.7E-78). These levels were several-fold higher than observed in muscle from mice. The Positive:Negative strand ratios were similar between the *IFN-αβR+/+* and *IFN-αβR-/-* MEFs (~24:1; **Table S3**), but were different from those in muscle and brain of infected mice, where there was less of a bias against the antisense strand.

West Nile Virus and Dengue Virus- derived vsRNAs

West Nile Virus (WNV), and Dengue Virus are positive-stranded, enveloped viruses belonging to the Flaviviridae family and thus their genome structures and replication strategies are similar to their more distant Flavivirus relative, HCV. However, in contrast to HCV, WNV and Dengue are arthropod-transmitted viruses and thus must be able to replicate in their respective mosquito vectors as well, where they must evade the host RNAi response [13,14].

West Nile Virus-derived vsRNAs: From studies utilizing mice to model WNV infection, it has become clear that WNV interacts extensively with host response mechanisms (for review, see [15]). In initial bulk assays for vsRNAs in African green monkey kidney cells, Chotkowski et al. [16] failed to detect WNV vsRNAs using northern analysis. We likewise failed to detect vsRNAs during infection in cells and tissues of an immuno-competent host (B6 mice; **Fig. 4D**, **Table S3**). These assays were carried out with relatively small sequencing sets (5,204 - 32,254 small RNAs instances from each dataset), and we hence cannot rule out the presence of relatively rare populations of vsRNAs.

To examine the WNV-host interaction in conditions that may be more amenable to functioning of the RNAi apparatus, we characterized small RNA populations in the presence of several host mutations that are known to specifically alter host responses to viral infection. In

infections with WNV, *IFN-α/βR-/-* (*IFN-α/β receptor-/-*) mice and *PKR-/-; RNaseL-/-* mice show expanded tissue tropism and a more severe disease outcome than wild type mice, with the highest virus burden and mortality consistently observed in the *IFN-α/βR-/-* background [17,18].

From all tissue types (brain, spleen, and lymph nodes) and primary cell types (Myeloid dendritic cells, macrophages, Embryonic fibroblasts or "MEFs", and cortical neurons) surveyed (**Table S3,** with relatively low-throughput sequence runs of 3,696 to 32,427 small RNAs), the only sample that yielded a population count (rather than a singular count) for vsRNAs was spleen from *IFN-α/βR-/-* mice at the three-day time-point (v/miR = 0.02; **Fig. 4E**). The vsRNA +/- strand ratio (3.0:1; **Table S3**) was more balanced than the ratio of genomic RNA (which is approximately 100:1 or greater; **Samuel M., unpublished data**), suggesting that these vsRNAs may be derived from precursors that are double-stranded.

Dengue virus-derived vsRNAs: From the various infection systems surveyed, small RNAs were characterized in relatively small datasets as with WNV above (5,129 to 15,508 sequences per sample). vsRNAs were only detectable at 24 h.p.i in the infected Huh7 cells (**Fig. S3**; v/miR = 0.002; +/- strand ratio of 6:0), and remained undetectable at the terminal time-points in U937 cells (35 h.p.i), and in Monocyte-derived dendritic cells (24 h.p.i; **Table S3**).

SUPPLEMENTARY METHODS

RNase protection

ago2+/+ or *ago2-/-* MEFs were infected with VSV-GFP at an MOI of 1 PFU/cell. RNA was harvested from 10^6 cells at 4 hours post-infection using the mirVana kit (Ambion), and analyzed by RNase protection using a T7-transcribed, α-^{32}P(CTP)-radiolabeled probe that was complementary to the first 700 bp of the VSV-N gene coding sequence. Probe was hybridized to 2µg of small RNAs (<200 bp) for 6 hours at room temperature followed by a 30-minute RNase A/T1 digestion at 37 °C (Ambion mirVana miRNA detection kit). Samples were purified according to manufacturer's instructions and analyzed by 15% denaturing PAGE.

REFERENCES

1. Liu J, Carmell MA, Rivas FV, Marsden CG, Thomson JM, et al. (2004) Argonaute2 is the catalytic engine of mammalian RNAi. Science 305: 1437-1441.
2. Kurilla MG, Keene JD (1983) The leader RNA of vesicular stomatitis virus is bound by a cellular protein reactive with anti-La lupus antibodies. Cell 34: 837-845.
3. Leppert M, Rittenhouse L, Perrault J, Summers DF, Kolakofsky D (1979) Plus and minus strand leader RNAs in negative strand virus-infected cells. Cell 18: 735-747.
4. Wilusz J, Kurilla MG, Keene JD (1983) A host protein (La) binds to a unique species of minus-sense leader RNA during replication of vesicular stomatitis virus. Proc Natl Acad Sci U S A 80: 5827-5831.
5. Lloyd RE, Bovee M (1993) Persistent infection of human erythroblastoid cells by poliovirus. Virology 194: 200-209.
6. Novak JE, Kirkegaard K (1991) Improved method for detecting poliovirus negative strands used to demonstrate specificity of positive-strand encapsidation and the ratio of positive to negative strands in infected cells. J Virol 65: 3384-3387.
7. Benton PA, Murphy JW, Lloyd RE (1995) K562 cell strains differ in their response to poliovirus infection. Virology 213: 7-18.
8. Hammond SM (2005) Dicing and slicing: the core machinery of the RNA interference pathway. FEBS Lett 579: 5822-5829.
9. Duchaine TF, Wohlschlegel JA, Kennedy S, Bei Y, Conte D, Jr., et al. (2006) Functional proteomics reveals the biochemical niche of C. elegans DCR-1 in multiple small-RNA-mediated pathways. Cell 124: 343-354.
10. Kennedy S, Wang D, Ruvkun G (2004) A conserved siRNA-degrading RNase negatively regulates RNA interference in C. elegans. Nature 427: 645-649.
11. Nomoto A, Koike S, Aoki J (1994) Tissue tropism and species specificity of poliovirus infection. Trends Microbiol 2: 47-51.
12. Ida-Hosonuma M, Iwasaki T, Yoshikawa T, Nagata N, Sato Y, et al. (2005) The alpha/beta interferon response controls tissue tropism and pathogenicity of poliovirus. J Virol 79: 4460-4469.
13. Sanchez-Vargas I, Scott JC, Poole-Smith BK, Franz AW, Barbosa-Solomieu V, et al. (2009) Dengue virus type 2 infections of Aedes aegypti are modulated by the mosquito's RNA interference pathway. PLoS Pathog 5: e1000299.
14. Brackney DE, Beane JE, Ebel GD (2009) RNAi targeting of West Nile virus in mosquito midguts promotes virus diversification. PLoS Pathog 5: e1000502.
15. Diamond MS, Mehlhop E, Oliphant T, Samuel MA (2009) The host immunologic response to West Nile encephalitis virus. Front Biosci 14: 3024-3034.
16. Chotkowski HL, Ciota AT, Jia Y, Puig-Basagoiti F, Kramer LD, et al. (2008) West Nile virus infection of Drosophila melanogaster induces a protective RNAi response. Virology 377: 197-206.
17. Samuel MA, Diamond MS (2005) Alpha/beta interferon protects against lethal West Nile virus infection by restricting cellular tropism and enhancing neuronal survival. J Virol 79: 13350-13361.

18. Samuel MA, Whitby K, Keller BC, Marri A, Barchet W, et al. (2006) PKR and RNase L contribute to protection against lethal West Nile Virus infection by controlling early viral spread in the periphery and replication in neurons. J Virol 80: 7009-7019.
19. Van Der Velden A, Kaminski A, Jackson RJ, Belsham GJ (1995) Defective point mutants of the encephalomyocarditis virus internal ribosome entry site can be complemented in trans. Virology 214: 82-90.
20. Kieft JS, Zhou K, Jubin R, Murray MG, Lau JY, et al. (1999) The hepatitis C virus internal ribosome entry site adopts an ion-dependent tertiary fold. J Mol Biol 292: 513-529.
