# Supplementary figures and images for: Six RNA Viruses and Forty-One Hosts: Viral Small RNAs and Modulation of Small RNA Repertoires in Vertebrate and Invertebrate Systems
Source: PLoS Pathog. 2010 Feb 12;6(2):e1000764. doi: 10.1371/journal.ppat.1000764 (PMC2820531; doi:10.1371/journal.ppat.1000764)

**S1.**

HEPATITIS C VIRUS

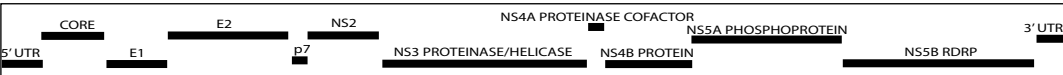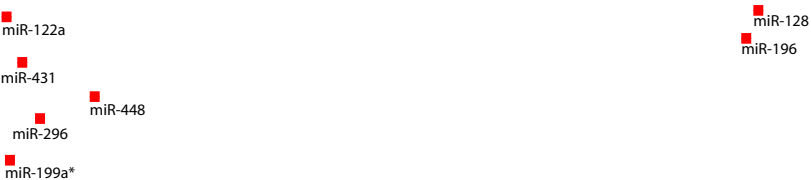

Supplement: Figure S1 — Host-encoded miRNAs with partial homology to HCV (summarized from published data). Human miRNAs that demonstrate a sequence-directed effect on HCV RNA levels are indicated [19],[20],[21]. These miRNAs are encoded in the host genome, and are hence distinct from HCVrep-derived vsRNAs that are viral-encoded. (0.19 MB PDF) [file ppat.1000764.s002.pdf]

**S4.**

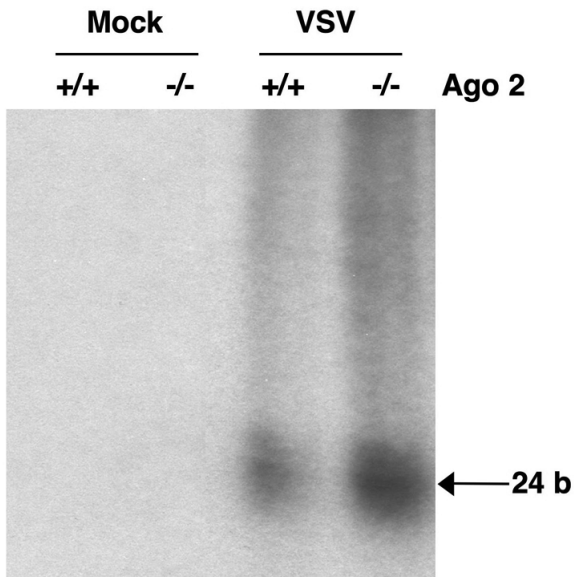

Supplement: Figure S4 — RNase-protection confirms that VSV-specific vsRNAs are more abundant in ago2−/− MEFs. RNA was isolated from mock-infected and VSV-infected ago2+/+ or ago2−/− MEFs at 4 h.p.i. vsRNAs derived from positive strand viral RNAs were detected by RNase protection using a radiolabeled probe specific to the 5′ end of the VSV-N gene. (0.69 MB PDF) [file ppat.1000764.s005.pdf]

**S6A.**

**(+) strand of Poliovirus**

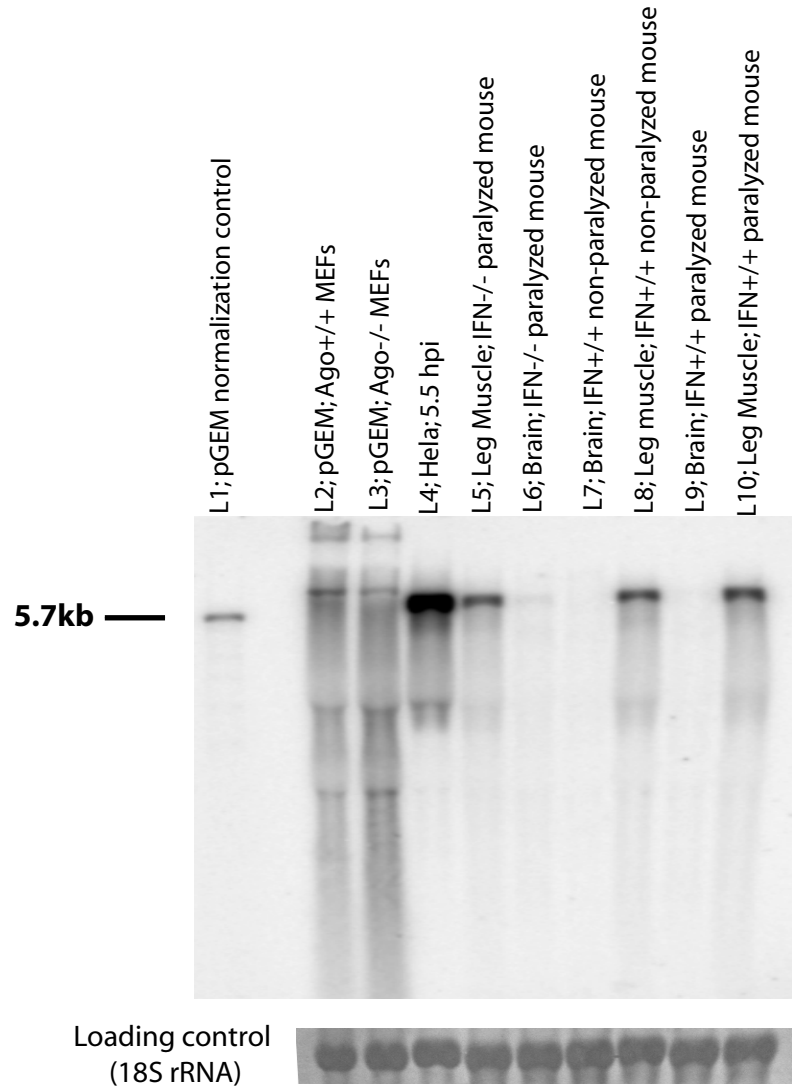

**S6B.**

**(-) strand of Poliovirus**

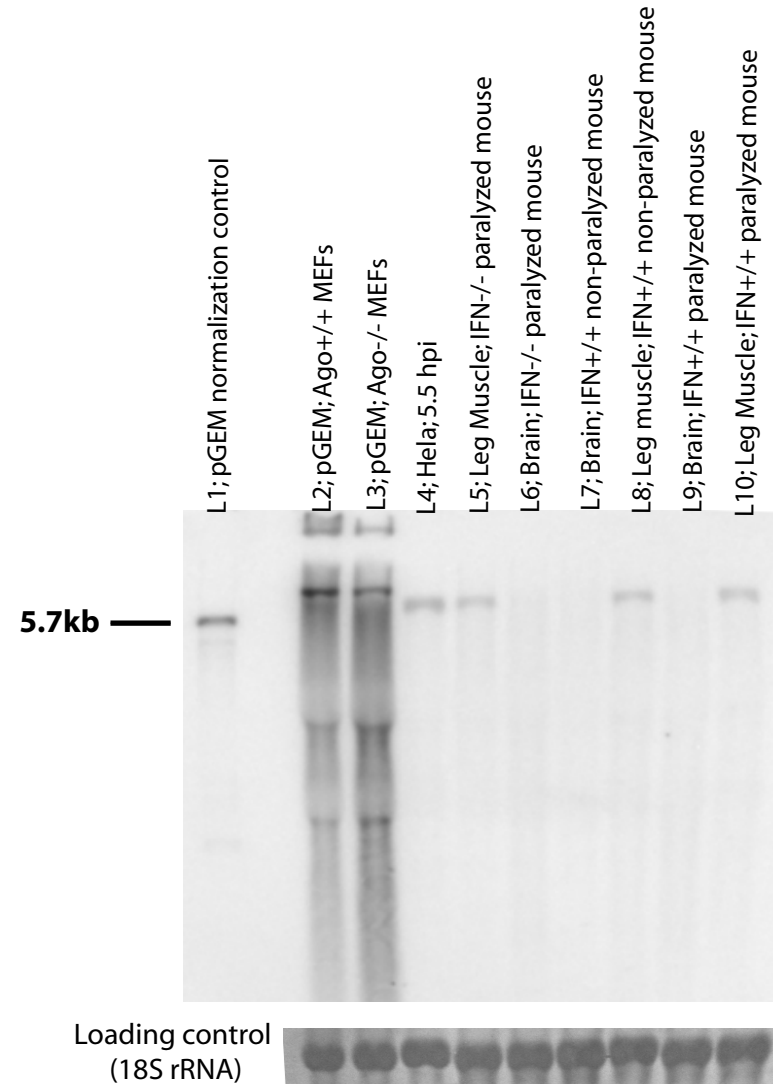

Supplement: Figure S6 — Abundance of the two polarities of Poliovirus full-length RNAs varies across systems, with full-length positive strand generally present in excess over full-length negative strand. Visualization of full-length Poliovirus in various samples by Northern analysis: (S6A) Positive strand; (S6B) Negative strand. Normalization control: fragment of plasmid with homology to Poliovirus (see Materials and Methods). (0.59 MB PDF) [file ppat.1000764.s007.pdf]

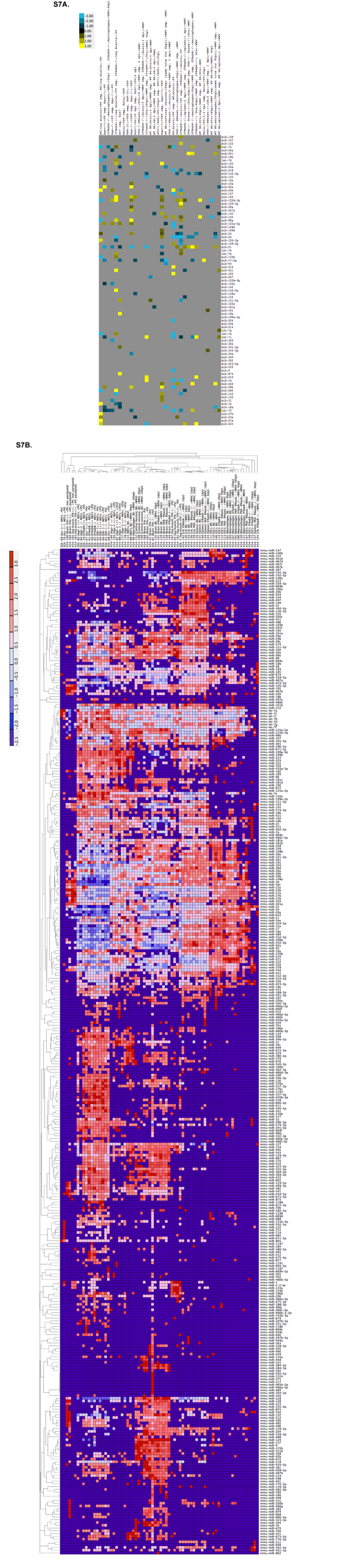

Supplement: Figure S7 — Only a handful of miRNAs are modulated during viral infection. (7A) Log2-transformed ratios of specific miRNA abundances between infected and uninfected samples (or between early and late time-points) were represented as a heat map. Only those miRNAs that exhibited a > = 2-fold, and a statistically significant upregulation or downregulation (i.e. no overlap of 95% confidence intervals) were considered. Fold changes were log2-transformed. No row-wise or column-wise normalizations were performed. The yellow squares represent miRNAs that are upregulated in infected cells/late time-points compared to uninfected/early time-points. The blue squares represent miRNAs that are downregulated in infected cells/late time-points compared to uninfected/early time-points. Higher intensities correlate with a greater fold change. Note: The baseline frequency for each miRNA was set to 0.0001 (rather than 0), to enable computation of ratios. (7B) Clustering of miRNA profiles across various infected and uninfected mouse tissues/cell lines reveals that majority of samples cluster according to tissue type/cell line origin. miRNA frequencies were first normalized against the total number of miRNAs for each sample. Cube-root values of the frequencies were subsequently used in uncentered clustering of the data set, both row-wise and column-wise. These plots were generated using GenePattern [http://www.broad.mit.edu/cancer/software/genepattern/]. (7.91 MB TIF) [file ppat.1000764.s008.tif]

**S12.**

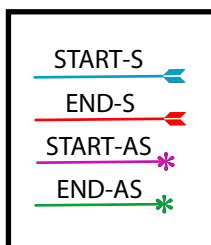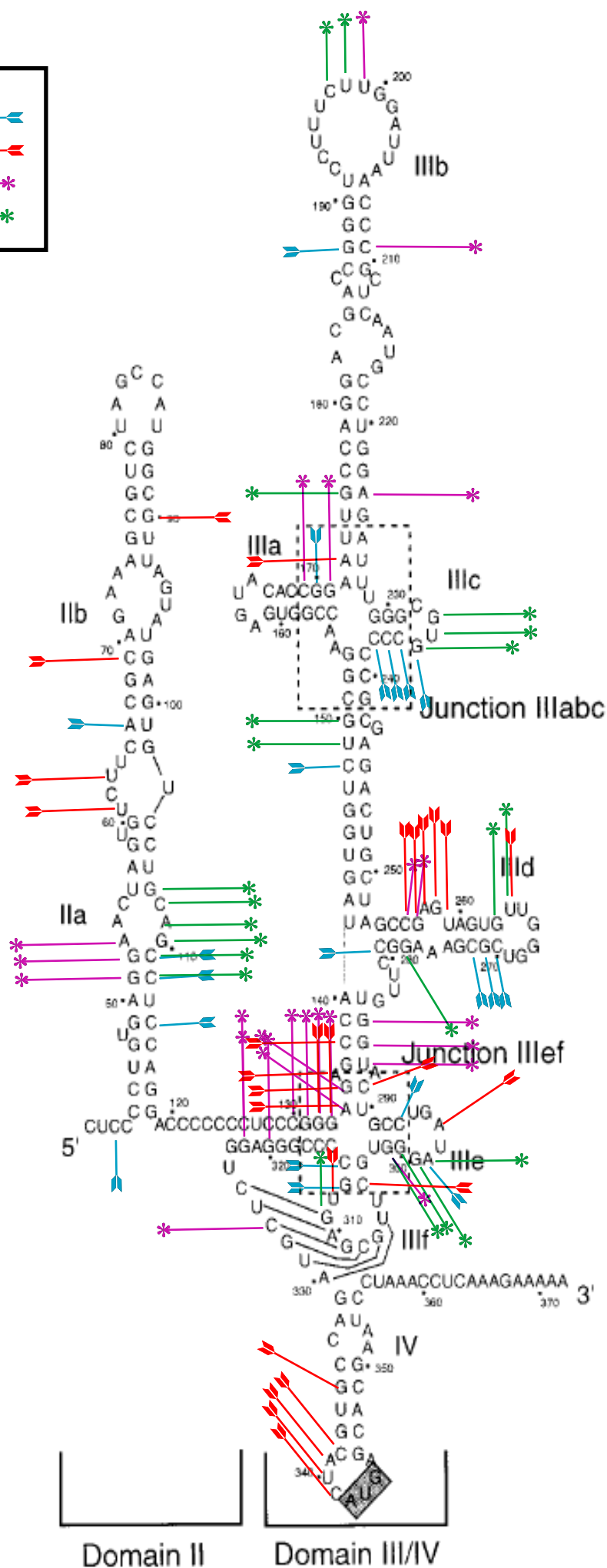

Supplement: Figure S12 — A map of vsRNA start and end positions, superimposed on the secondary structure of the HCV IRES (see [19] for source of IRES structure). Only positions that had an incidence of > = 2 Starts/Ends were mapped. (0.32 MB PDF) [file ppat.1000764.s013.pdf]

S13.

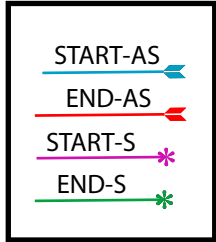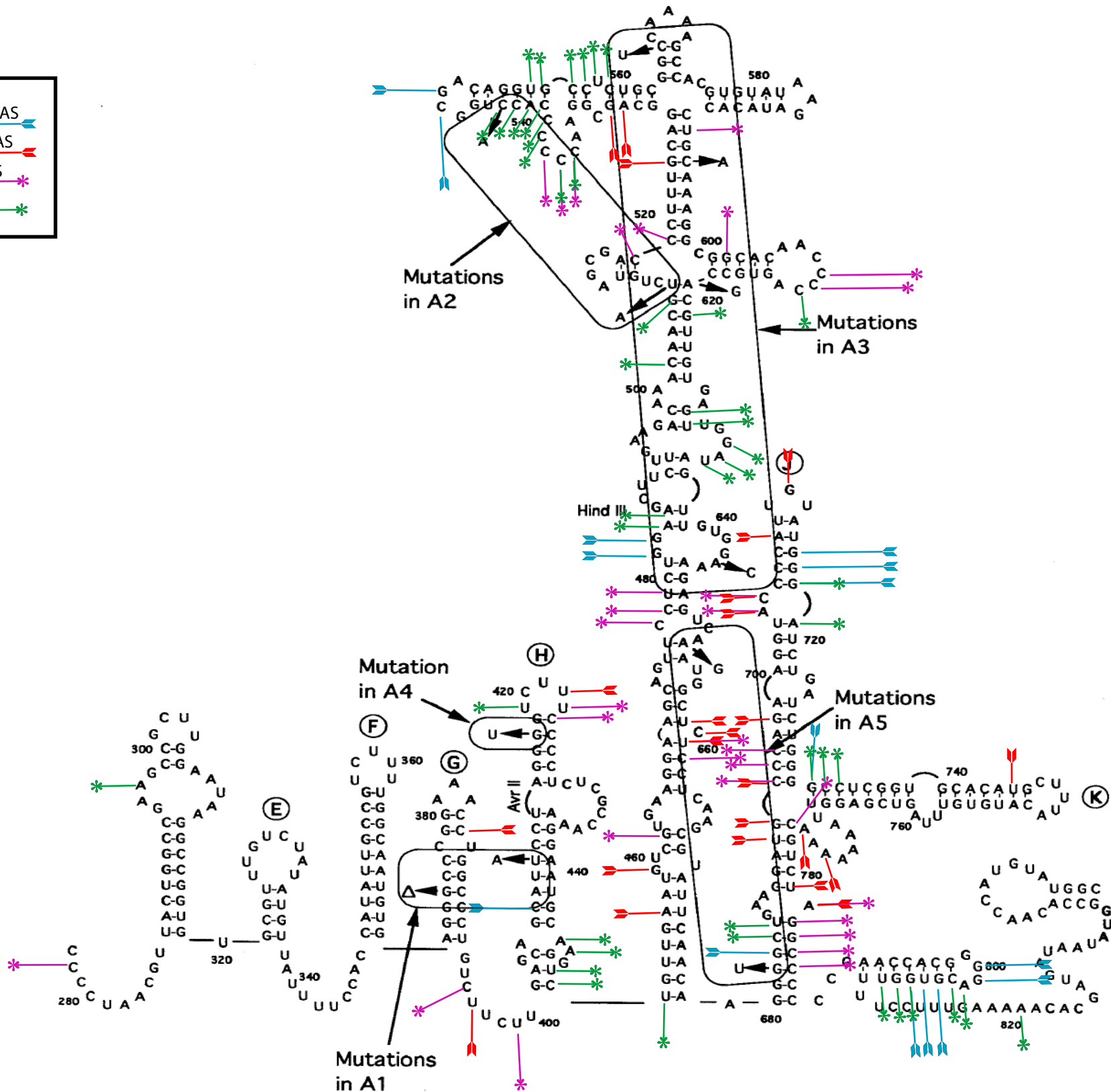

Supplement: Figure S13 — A map of vsRNA start and end positions, superimposed on the secondary structure of the EMCV IRES (which is part of the HCV Replicon genome; see [20] for source of IRES structure). Only positions that had an incidence of > = 2 starts/ends were mapped. (0.42 MB PDF) [file ppat.1000764.s014.pdf]

**S15A.**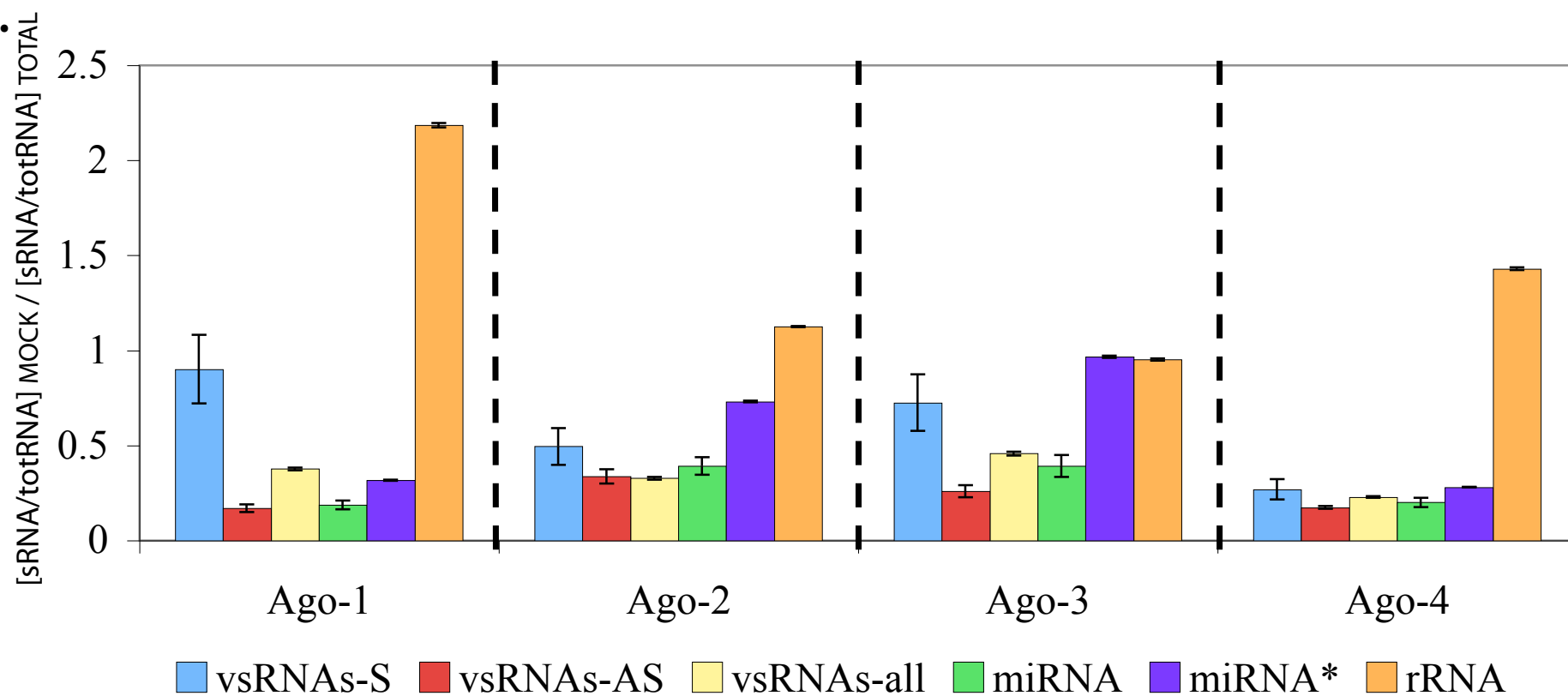**S15B.**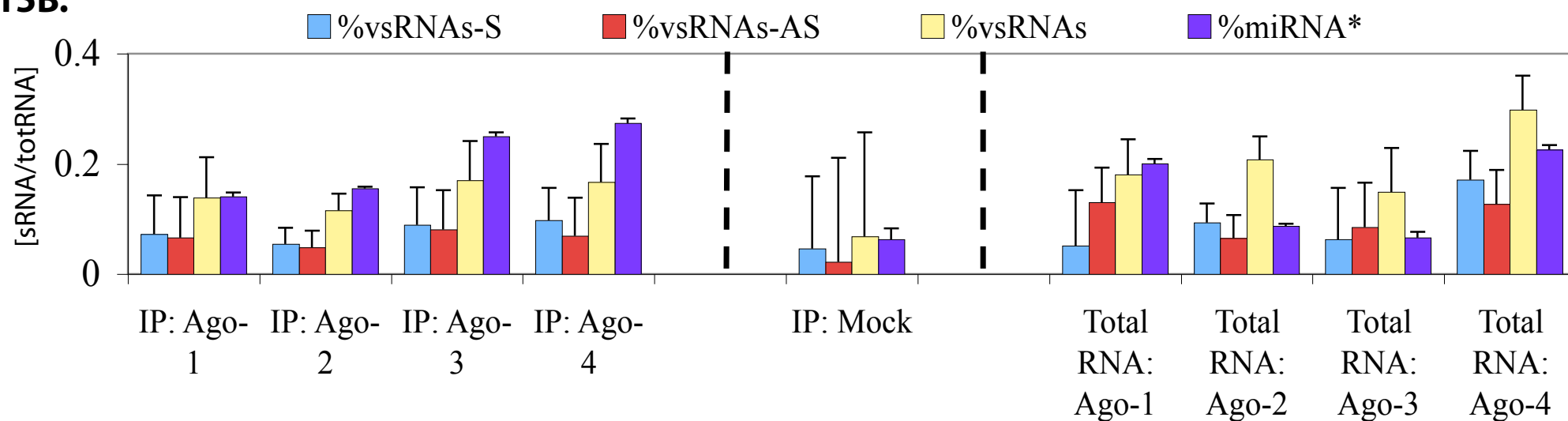**S15C.**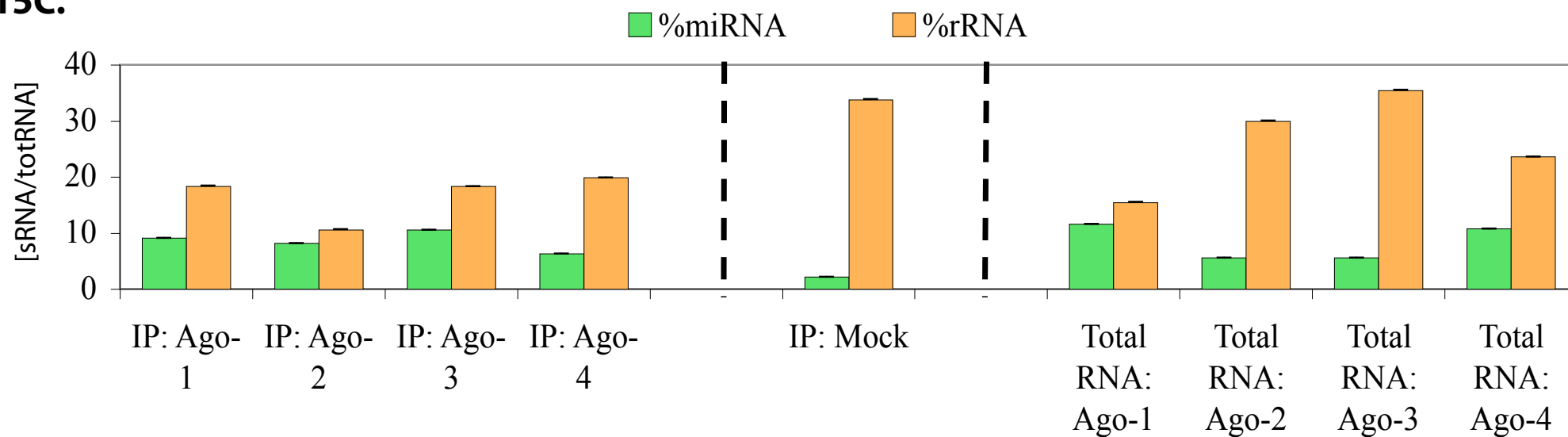

Supplement: Figure S15 — Mock-IP enriches for rRNAs. (S15A) [(xRNA/totSeq)MockIP/(xRNA/totSeq)totalRNA] was computed for the various IPs; xRNA: vsRNA, miRNA, miRNA*, or rRNA; totSeq = total number of sequences from each experiment. The number of vsRNAs varied from 86 to 1,857, and the number of total sequences varied from 126,022 to 891,858 in these samples. (S15B–C) Raw data: [(xRNA/totSeq)] for the various IPs (including the mock IP), and for the totalRNA populations; xRNA: vsRNA, miRNA, miRNA*, or rRNA; totSeq: total number of sequences from each experiment. (0.25 MB PDF) [file ppat.1000764.s016.pdf]

% sequences in +/-120 window

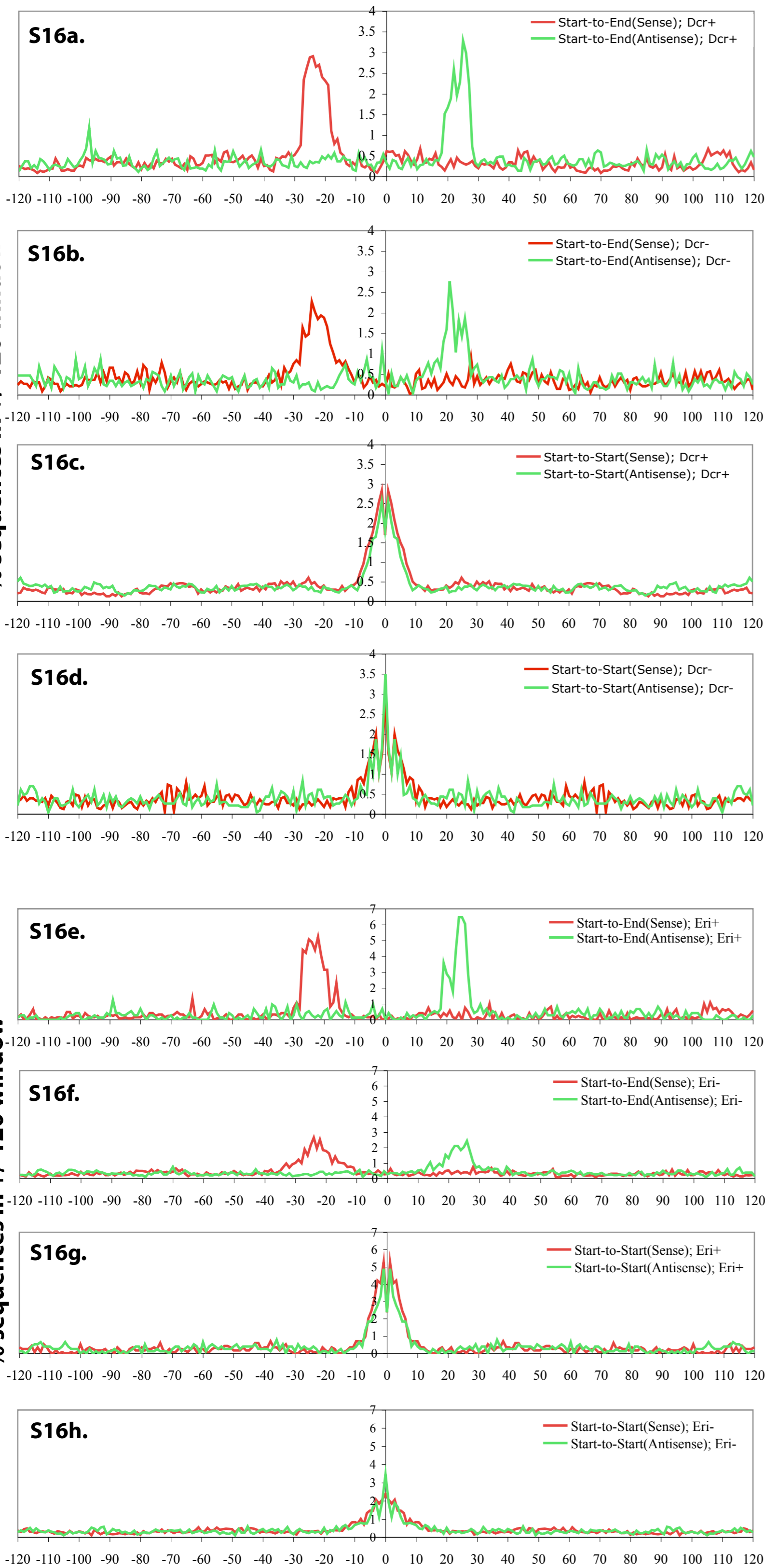

% sequences in +/-120 window

%duplexes in +/-25 window

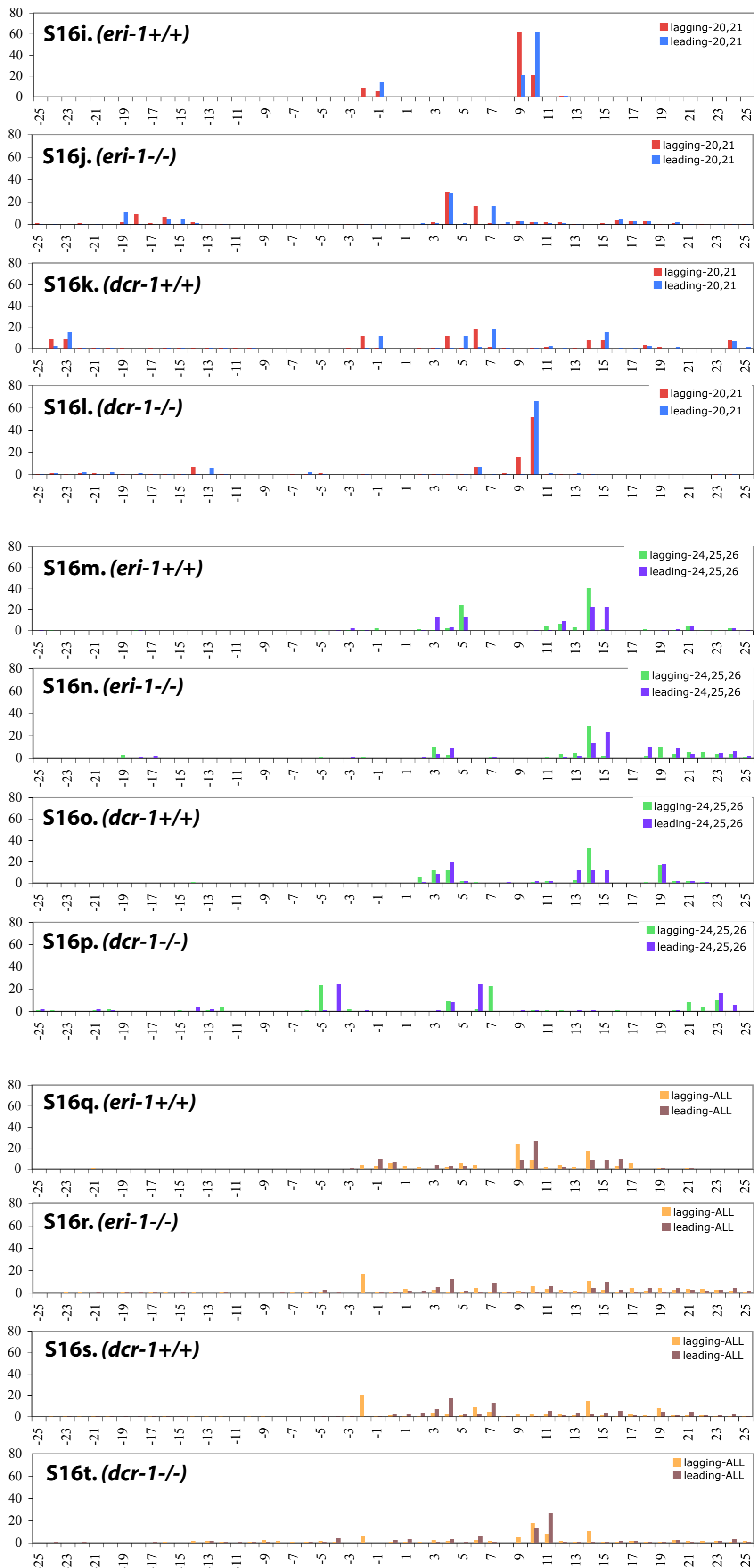

%duplexes in +/-25 window

Supplement: Figure S16 — Start-to-Start, Start-to-End, and duplex overhang plots for positive strand and negative strand vsRNAs show differences between Poliovirus-infected hosts with wild-type or mutant copies of dcr-1 and eri-1. Y-axis: percent of vsRNA pairs with specified Start-to-Start or Start-to-End distances on a scale of +120 to −120. (S16A, S16B, S16E, S16F) Start-to-End distances for positive strand and negative strand vsRNAs cloned from dcr-1+/+, dcr-1−/−, eri-1+/+, eri-1−/− MEFs; (S16C, S16D, S16G, S16H) Start-to-Start distances for positive strand and negative strand vsRNAs cloned from dcr-1+/+, dcr-1−/−, eri-1+/+, eri-1−/− MEFs. Predicted overhangs formed by overlapping sets of sense and antisense 20 and 21 nt vsRNAs sequenced from: (S16I) eri-1+/+ MEFs +Poliovirus; (S16J) eri-1−/− MEFs +Poliovirus; (S16K) dcr-1+/+ MEFs +Poliovirus; (S16L) dcr-1−/− MEFs +Poliovirus. (S16M-P) Overhangs formed by 24,25,26-mers from systems listed above. (S16Q-T) Overhangs formed by all size-classes of vsRNAs from systems listed above. All captured vsRNAs of either polarity were considered to be potential partners for this analysis. (0.50 MB PDF) [file ppat.1000764.s017.pdf]

S18A.

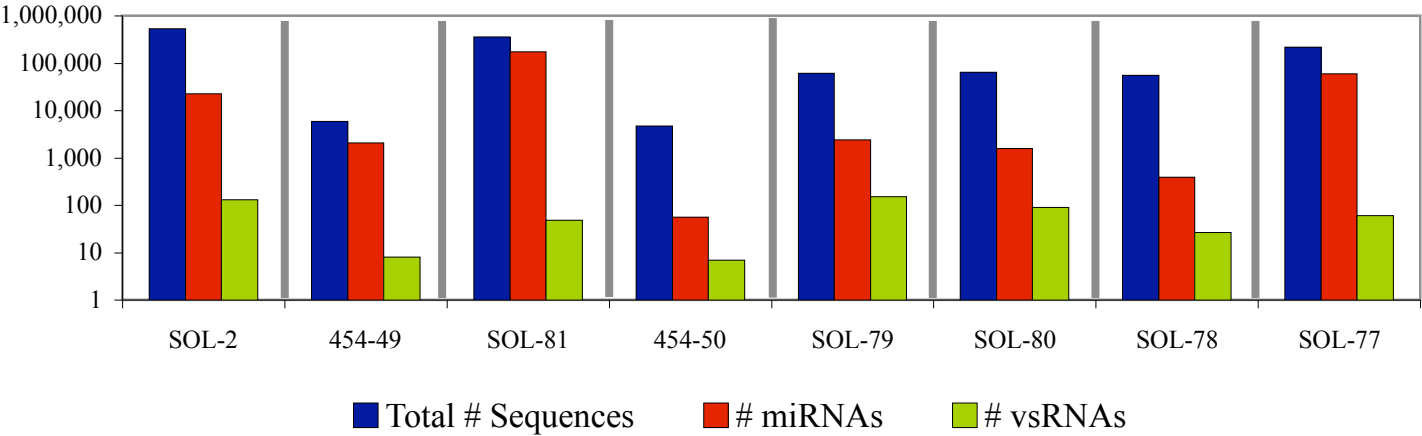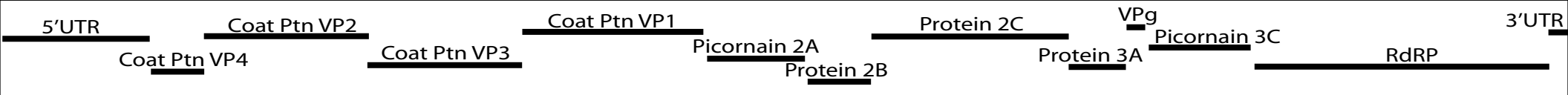

S18B.

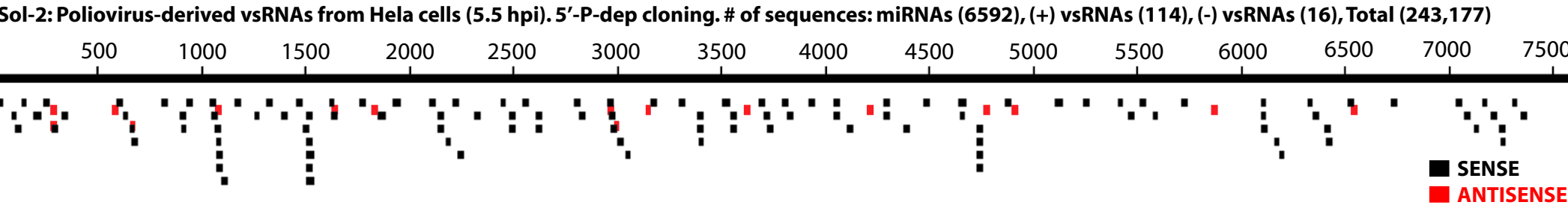

S18C.

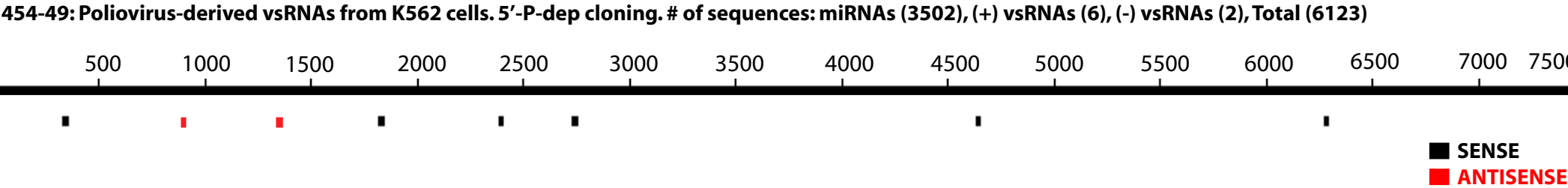

S18D.

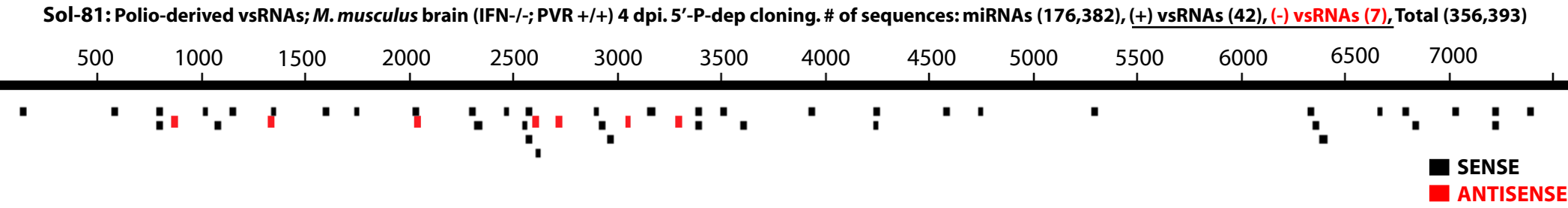

S18E.

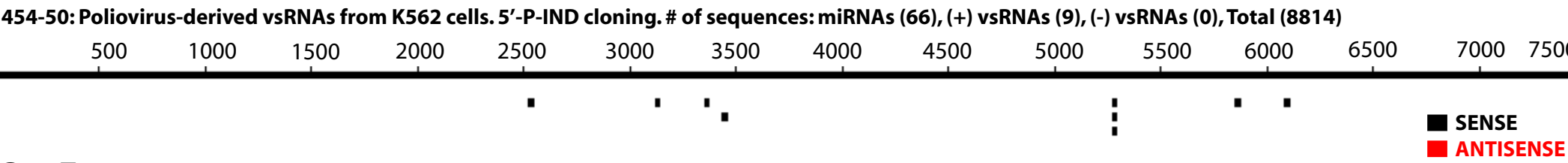

S18F.

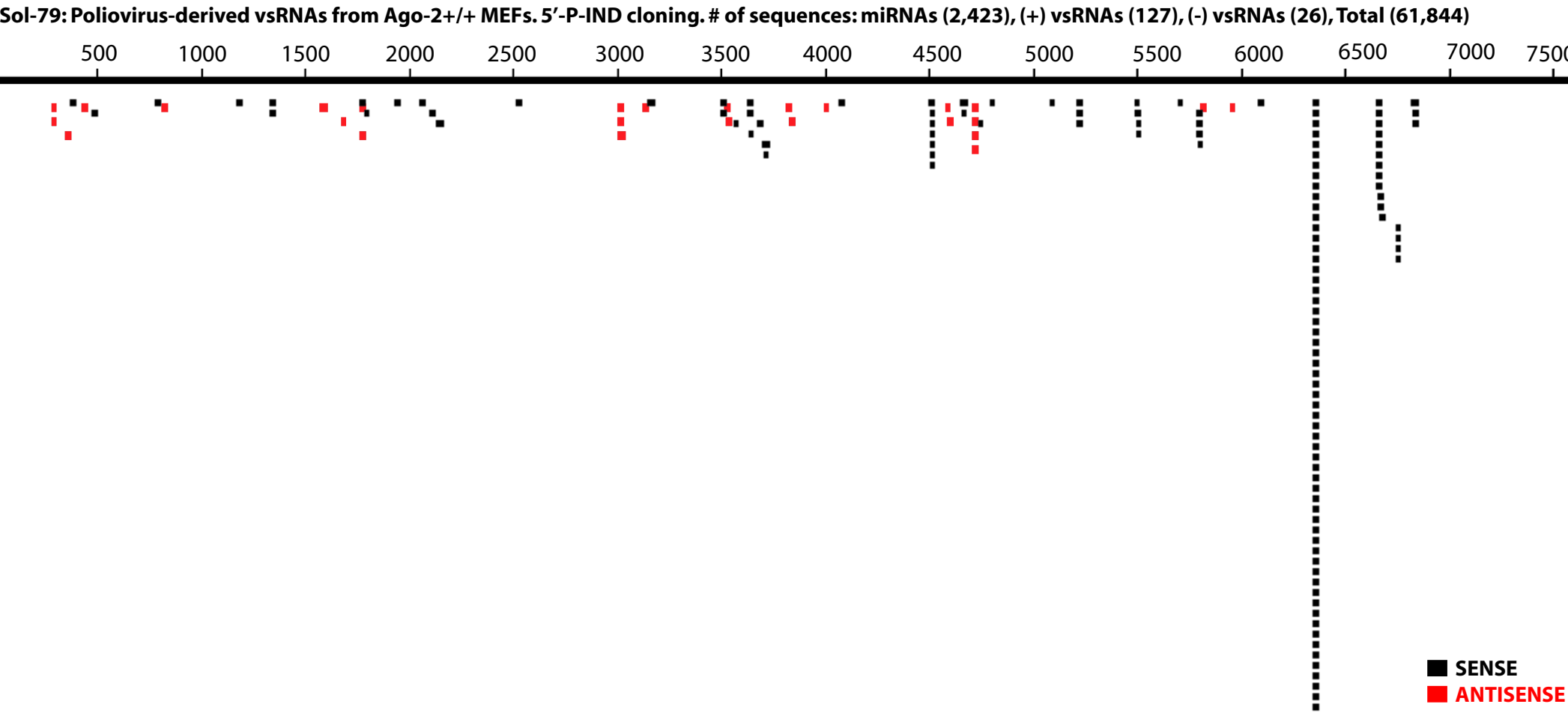

S18G.

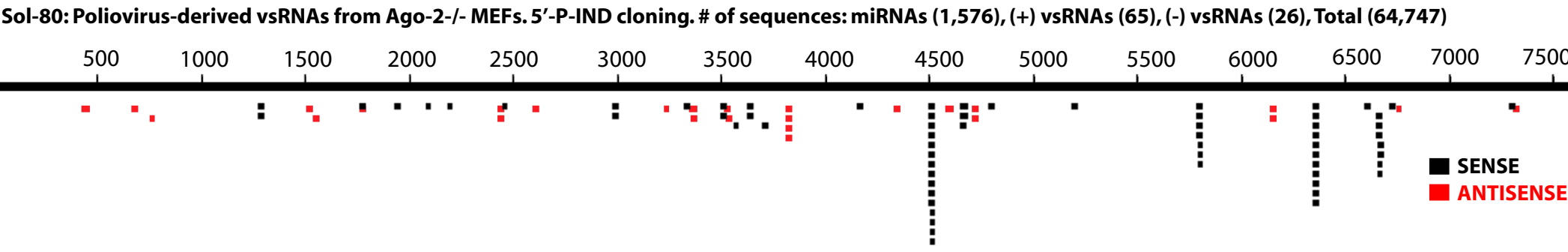

S18H.

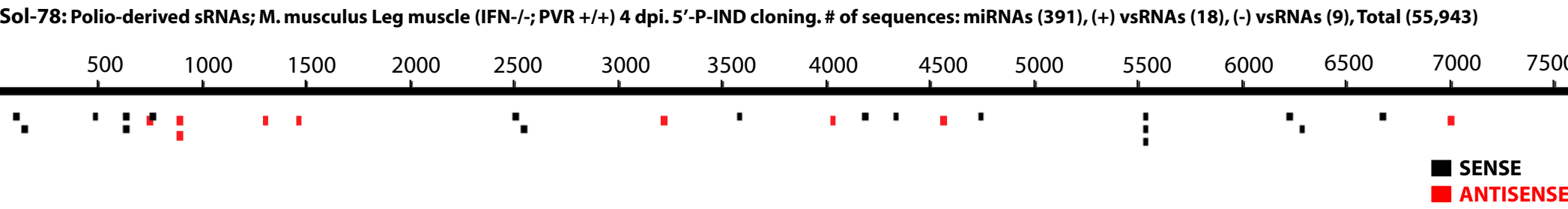

S18I.

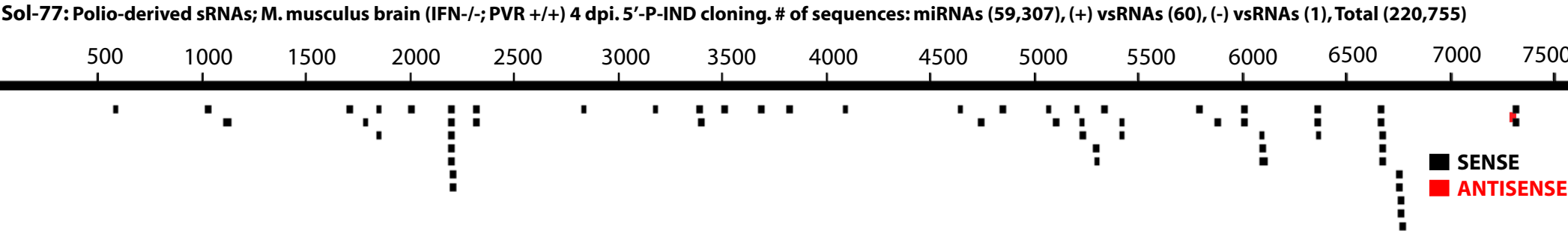

Supplement: Figure S18 — vsRNAs are present in both lytic and persistent models of Poliovirus infection, and can be captured using both the 5′-P-dependent, and the 5′-P-INDependent cloning protocols. (S18A) Sequence count: all RNAs, miRNAs, vsRNAs (Y-axis: log scale). vsRNAs with a 5′ monophosphate from: (S18B) HeLa cells, that are prone to lysis upon infection with the Mahoney strain of poliovirus (MOI = 5, 5.5 h.p.i, Sample: Sol-1); (S18C) K562 cells, in which Poliovirus establishes a persistent infection (Sample: 454-49); (S18D) brain of 6wk-old paralyzed Poliovirus-infected IFNαβR−/−; PVR+/+ male mouse, 4 d.p.i. (Sol-81). vsRNAs captured using the 5′-Phosphate-INDependent cloning protocol, from: (S18E) K562 cells infected with Poliovirus (Sample: 454-50); (S18F) ago-2+/+ MEFs +Poliovirus (Sample: Sol-79)*; (S18G) ago-2−/− MEFs +Poliovirus (Sample: Sol-80)*; leg muscle (S18H) and brain (S18I) of a 6wk-old male mouse (genotype: IFNαβR−/−; PVR+/+) infected with Poliovirus, 4 d.p.i. *These MEFs were transfected with a plasmid encoding for self-replicating Poliovirus RNA, and harvested 5 hours post-transfection. (0.35 MB PDF) [file ppat.1000764.s019.pdf]

**S19A.**

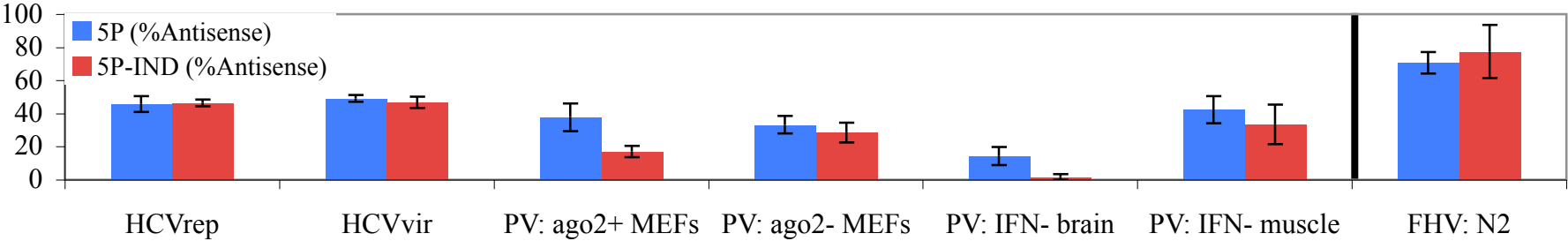

**S19B.**

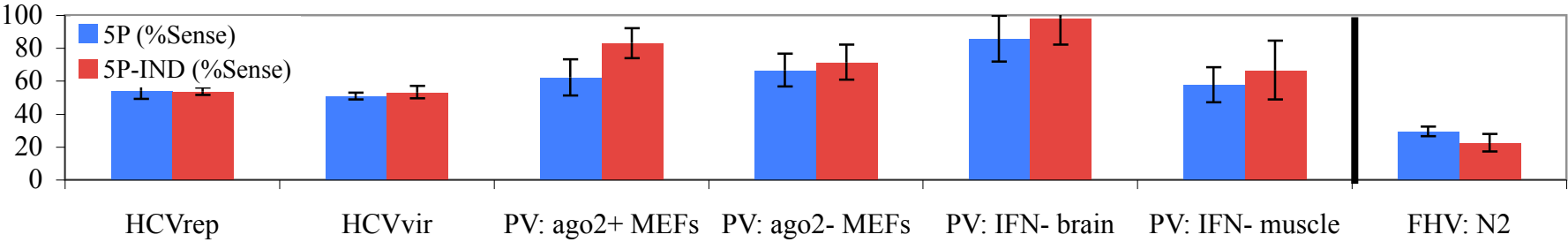

Supplement: Figure S19 — The 5′-P-INDependent cloning protocol subtly enriches for antisense vsRNAs. Comparison of antisense vsRNAs (as a % of all vsRNAs; S19A) and of sense vsRNAs (as a % of all vsRNAs; S19B), between the 5′-P-dependent and the 5′-P-INDependent cloning protocols. (0.23 MB PDF) [file ppat.1000764.s020.pdf]
