# Supplementary material for: Six RNA Viruses and Forty-One Hosts: Viral Small RNAs and Modulation of Small RNA Repertoires in Vertebrate and Invertebrate Systems
Source: PLoS Pathog. 2010 Feb 12;6(2):e1000764. doi: 10.1371/journal.ppat.1000764 (PMC2820531; doi:10.1371/journal.ppat.1000764)

S2.a: Flock House Virus vsRNAs from *N2* worms: Solexa (5'-P-independent cloning; Sol-52)

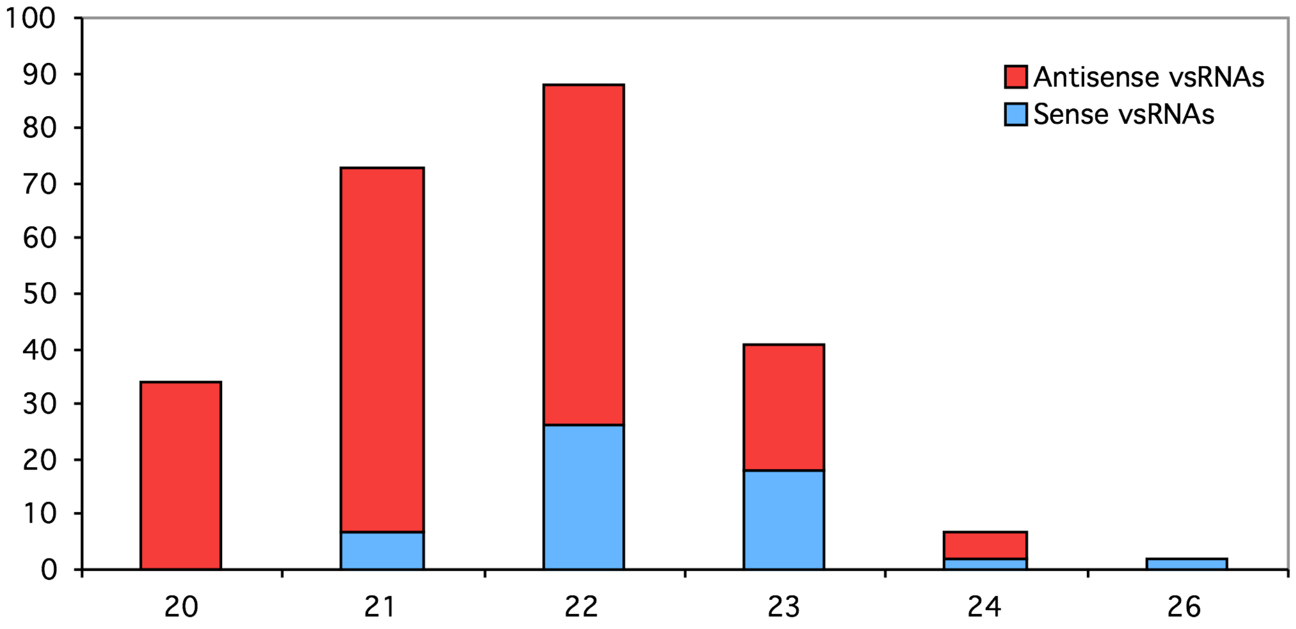

S2.b: Flock House Virus vsRNAs from *rde-4* worms: Solexa (5'-P-independent cloning; SOL-50)

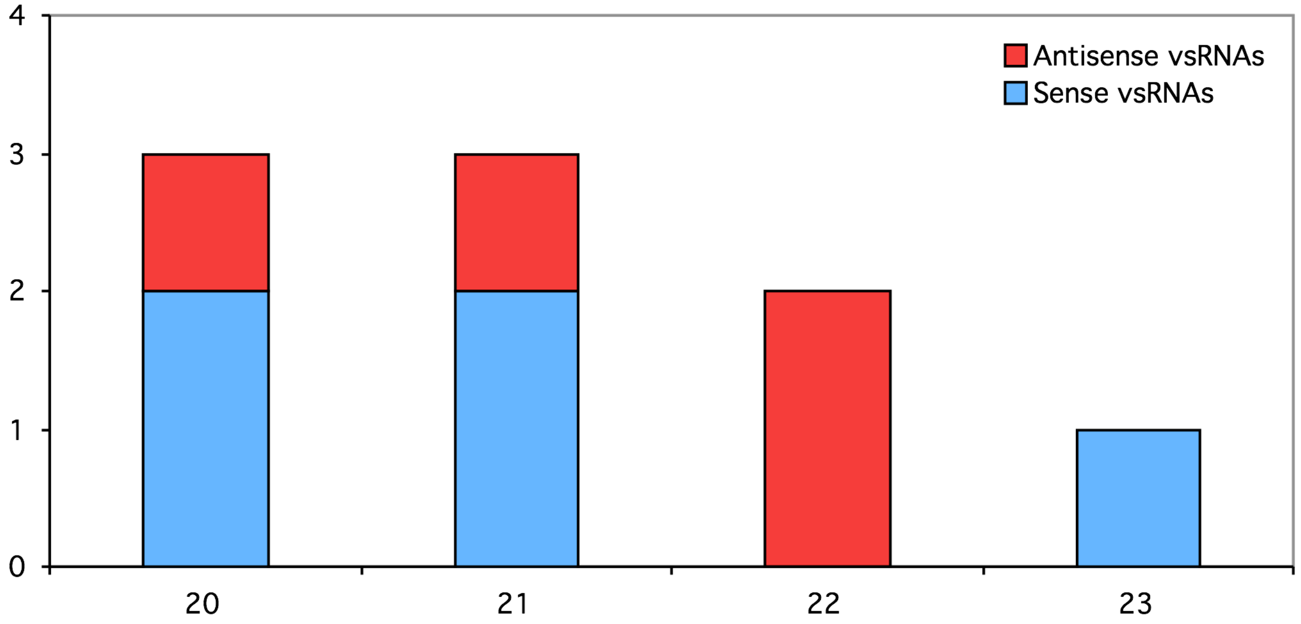

S2.c: Flock House Virus-derived vsRNAs from *C. elegans (rde-1)* 24 hpi: Solexa data (5' P-dependent cloning; Sol-72)

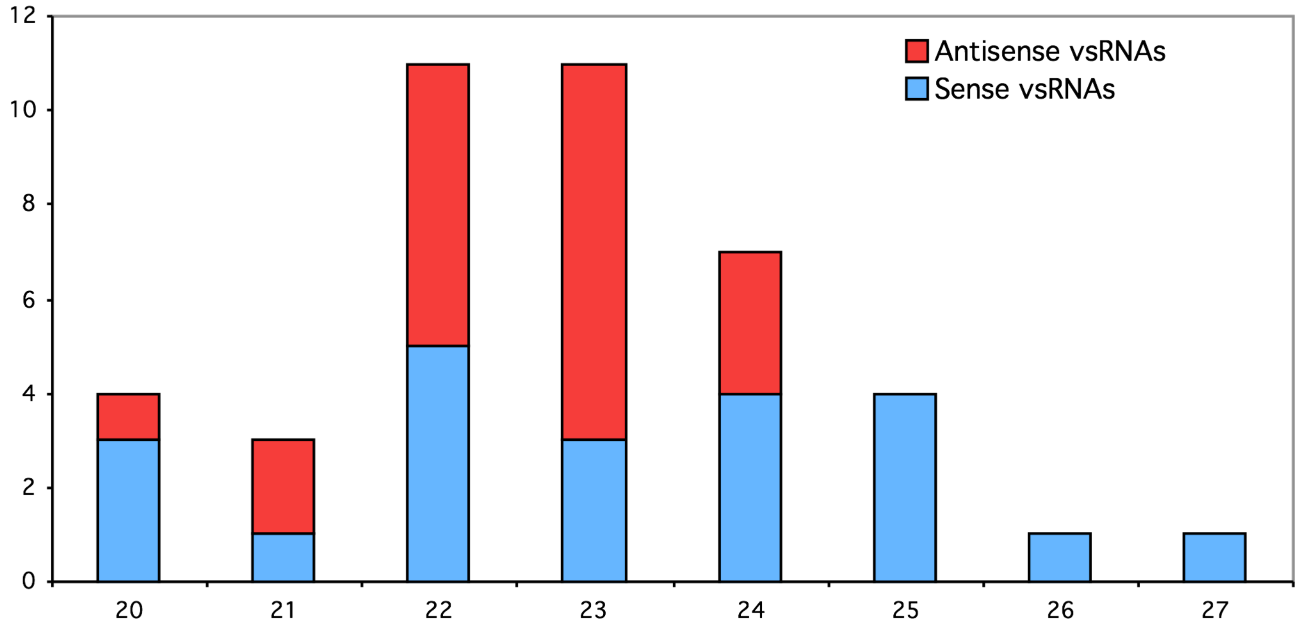

S2.d: Flock House Virus-derived vsRNAs from *C. elegans (N2)* 24 hpi: Solexa data (5' P-dependent cloning; Sol-73)

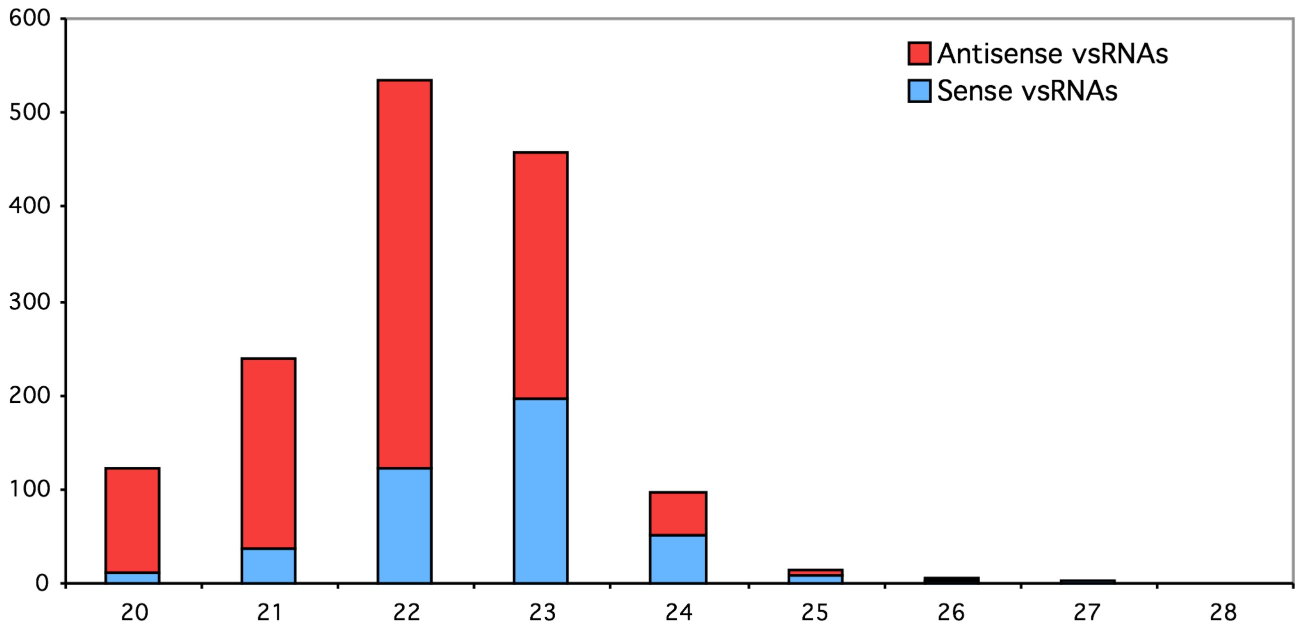

S2.e: Hepatitis C Virus (Replicon) vsRNAs: Cumulative 454 data (5'-P-dependent cloning)

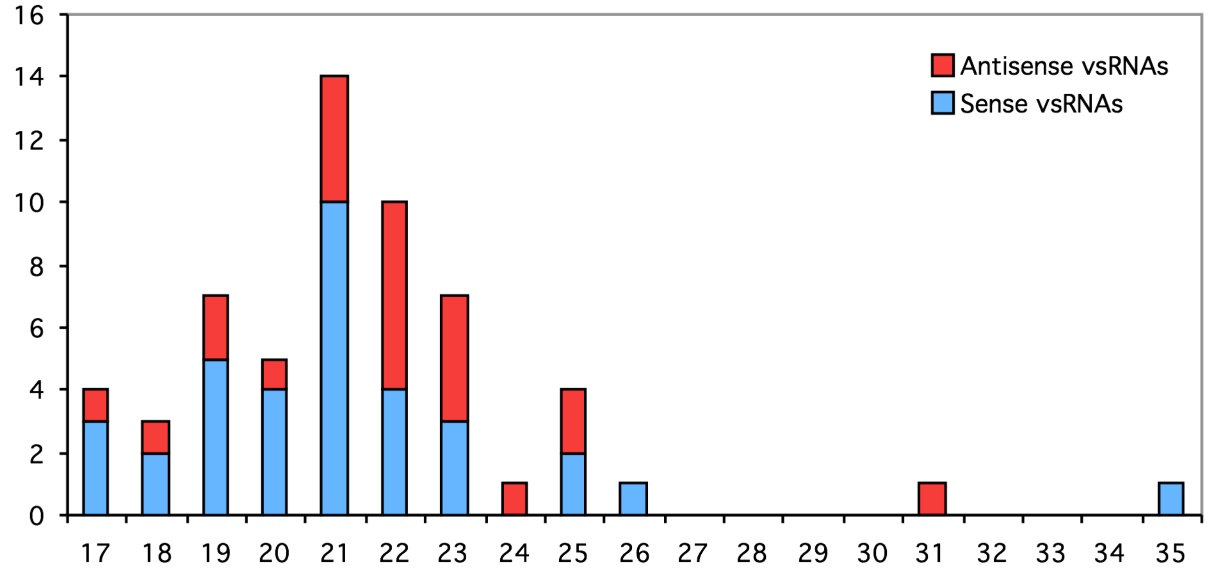

S2.f: Hepatitis C Replicon-derived vsRNAs from RP7; late passage: Solexa data (5' P-dependent cloning; Sol-4)

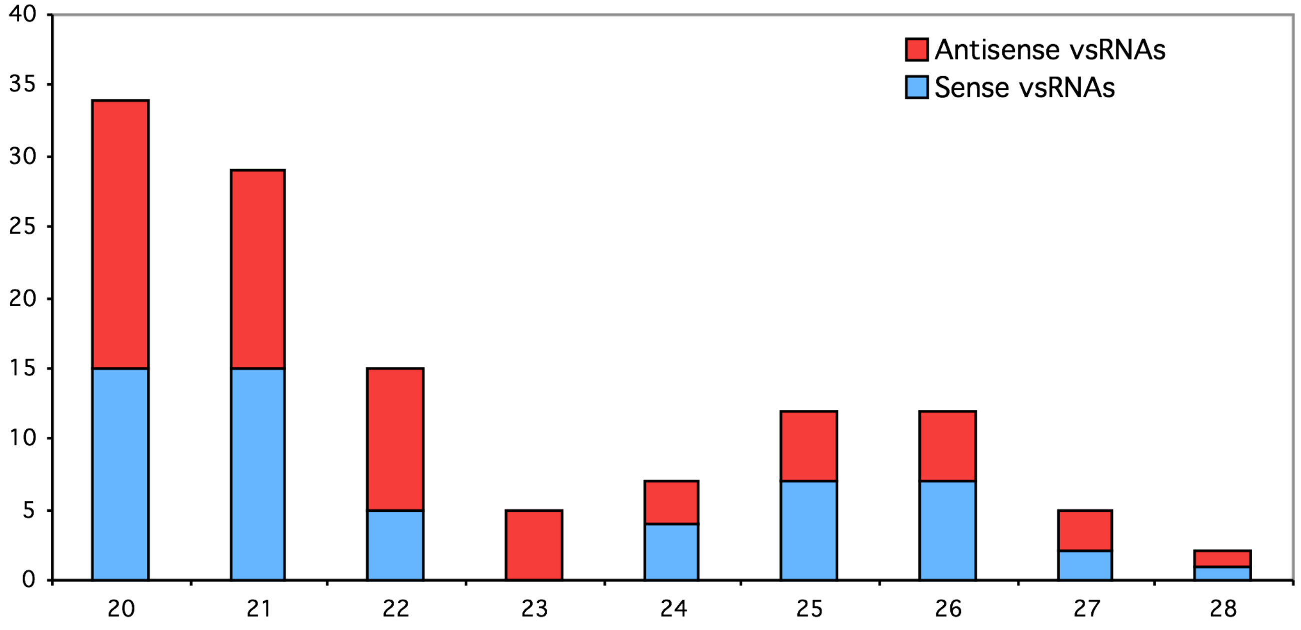

S2.g: Hepatitis C Replicon-derived vsRNAs from RP7; late passage: Solexa data (5' P-dependent cloning; Sol-107)

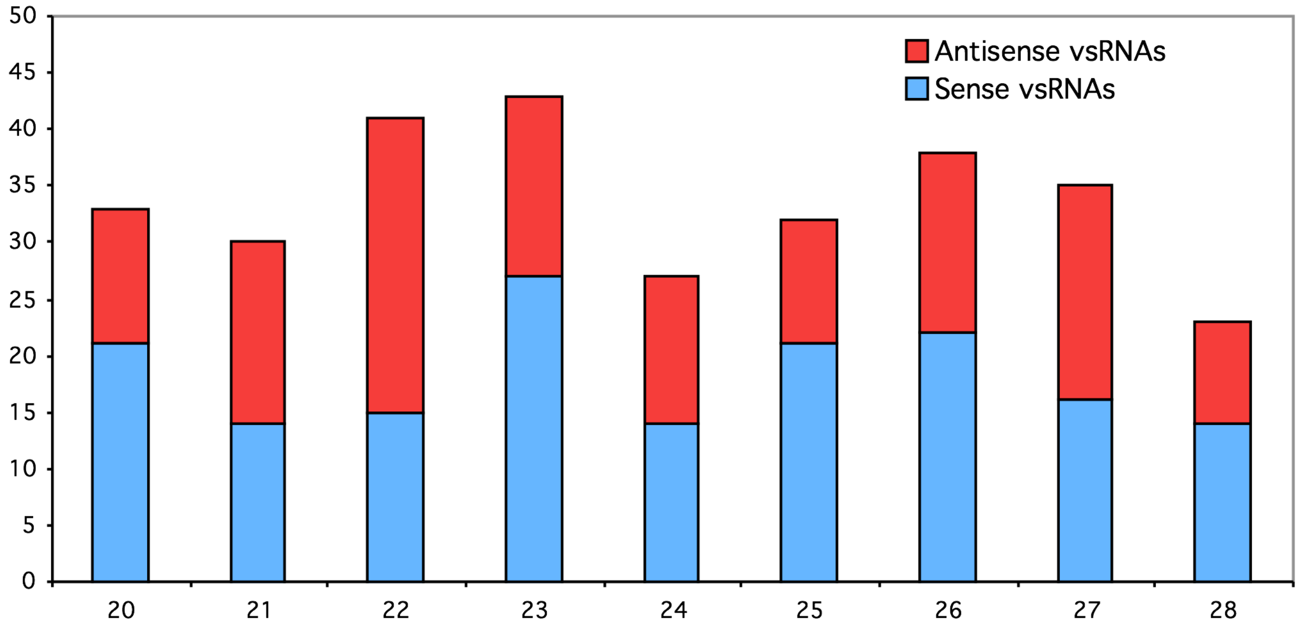

S2.h: Hepatitis C Replicon-derived vsRNAs from RP7; late passage: Solexa data (5' P-INDependent cloning; Sol-109)

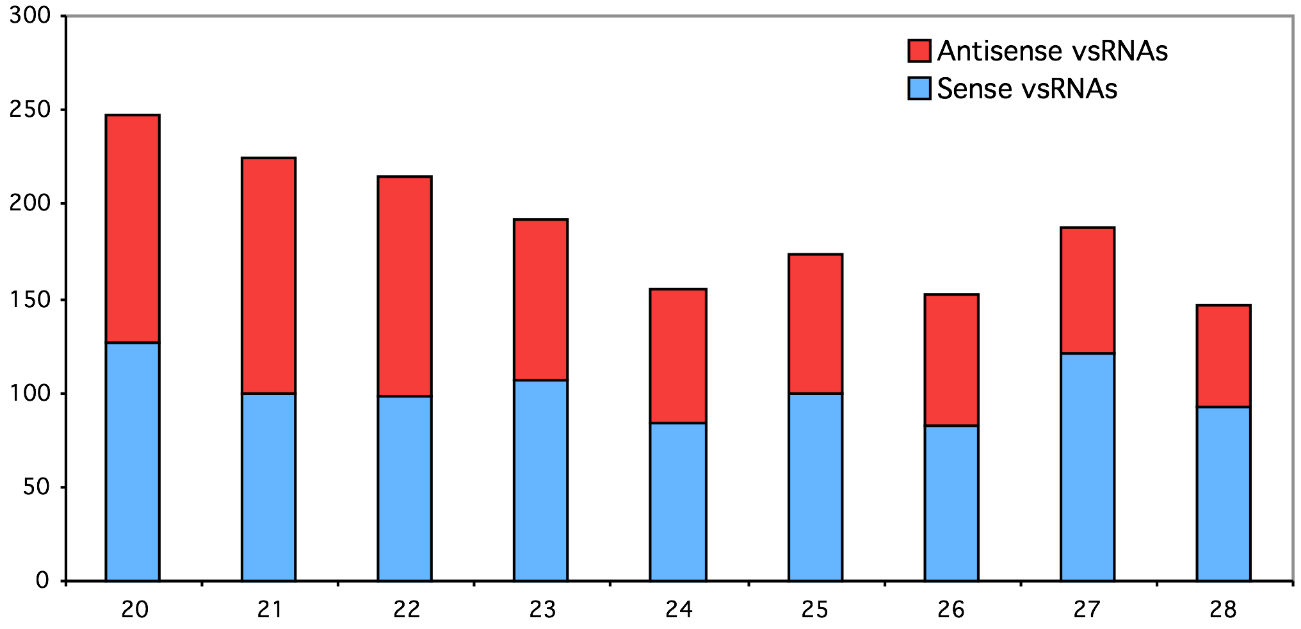

S2.i: Hepatitis C Replicon-derived vsRNAs from RP7; early passage: Solexa data
(5' P-dependent cloning; Sol-176)

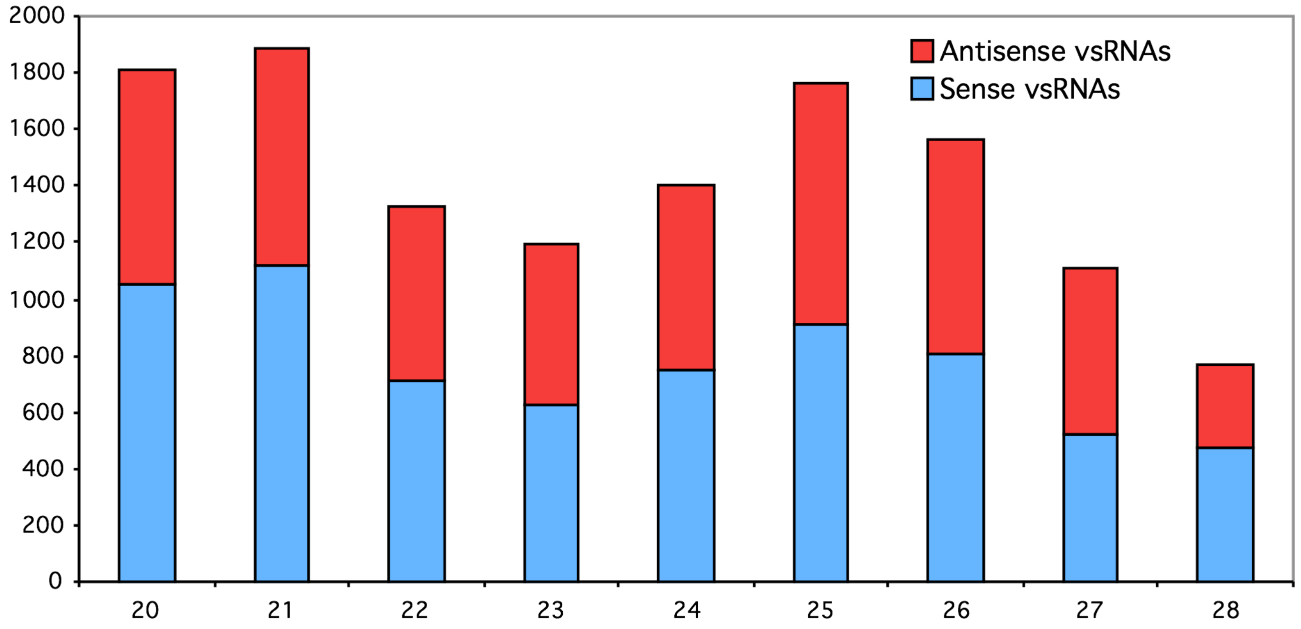

S2.j: Hepatitis C Replicon-derived vsRNAs from RP7; early passage: Solexa data
(5' P-INDependent cloning; Sol-179)

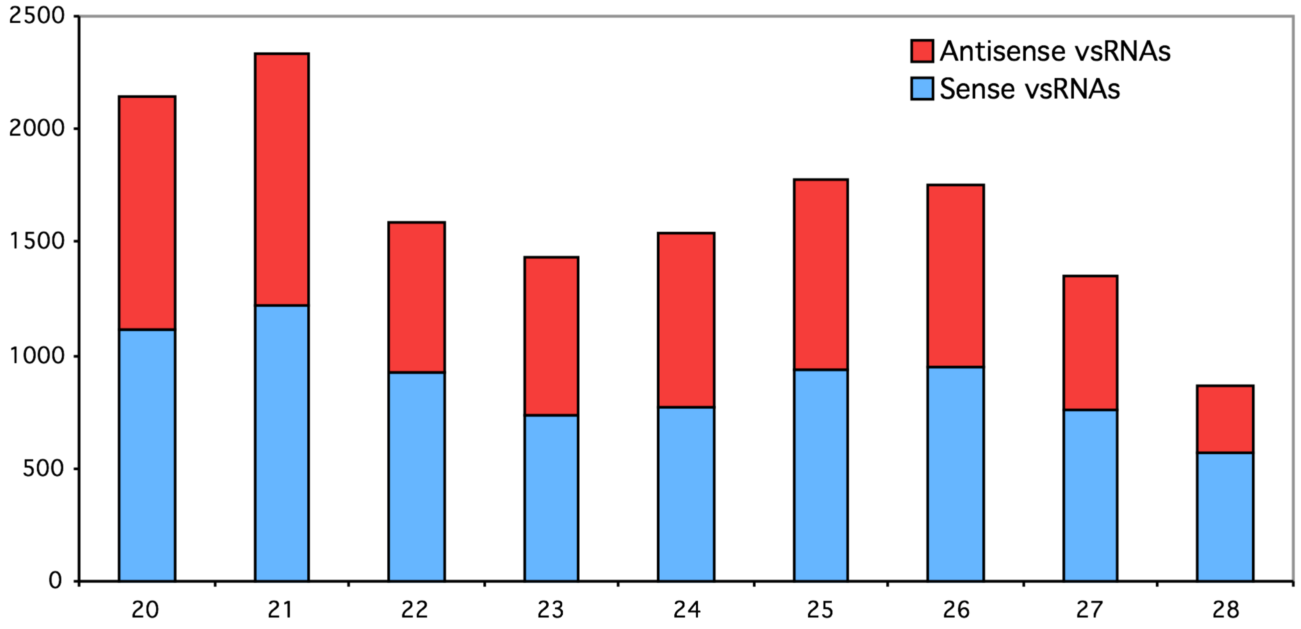

S2.k: Hepatitis C Replicon-derived vsRNAs from RP7; Ago-1 IP: Solexa data (5' P-dependent cloning; Sol-32)

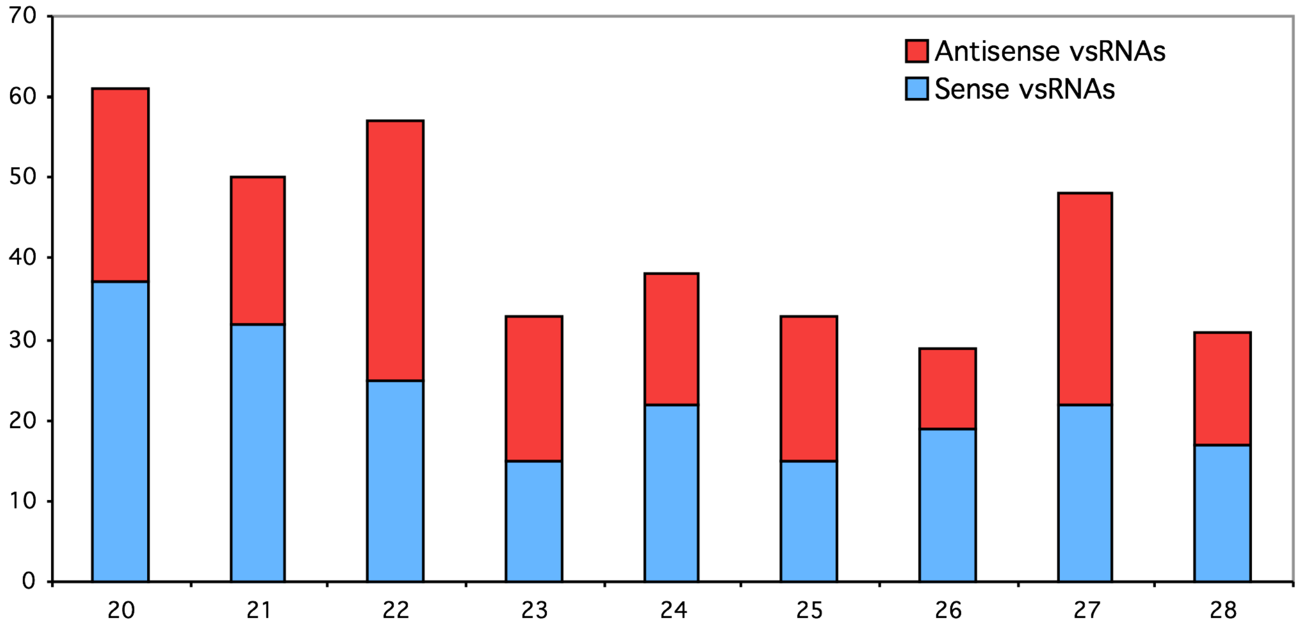

S2.l: Hepatitis C Replicon-derived vsRNAs from RP7; Ago-2 IP: Solexa data (5' P-dependent cloning; Sol-48)

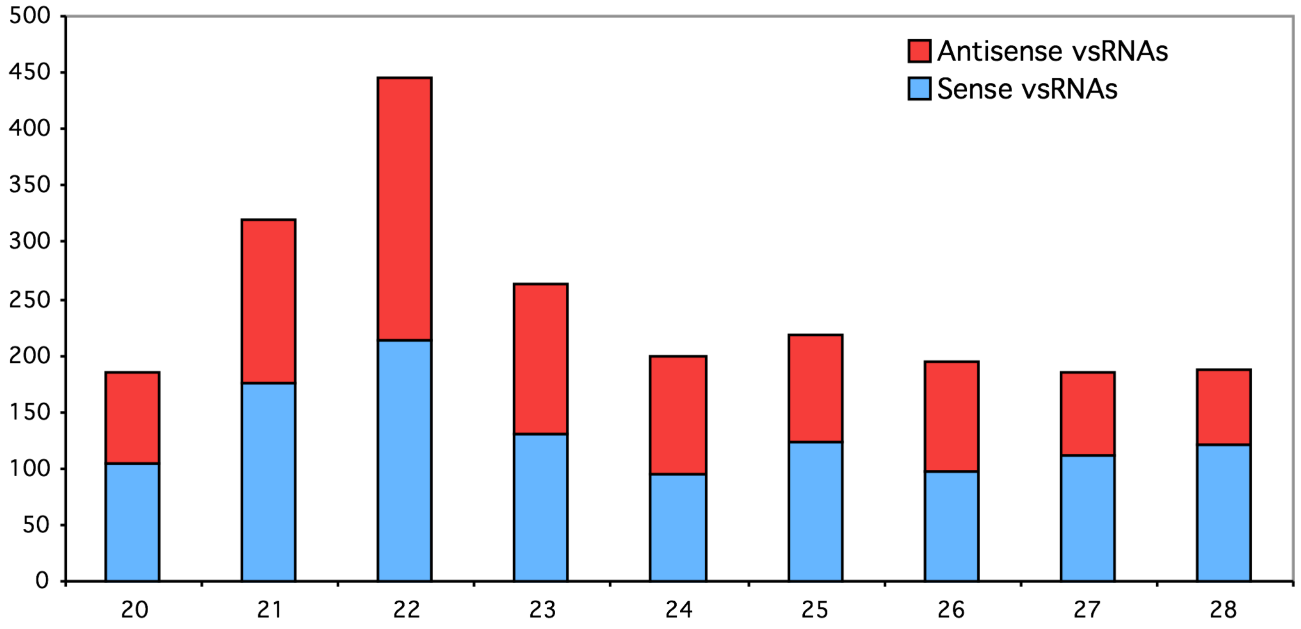

S2.m: Hepatitis C Replicon-derived vsRNAs from RP7; Ago-3 IP: Solexa data (5' P-dependent cloning; Sol-34)

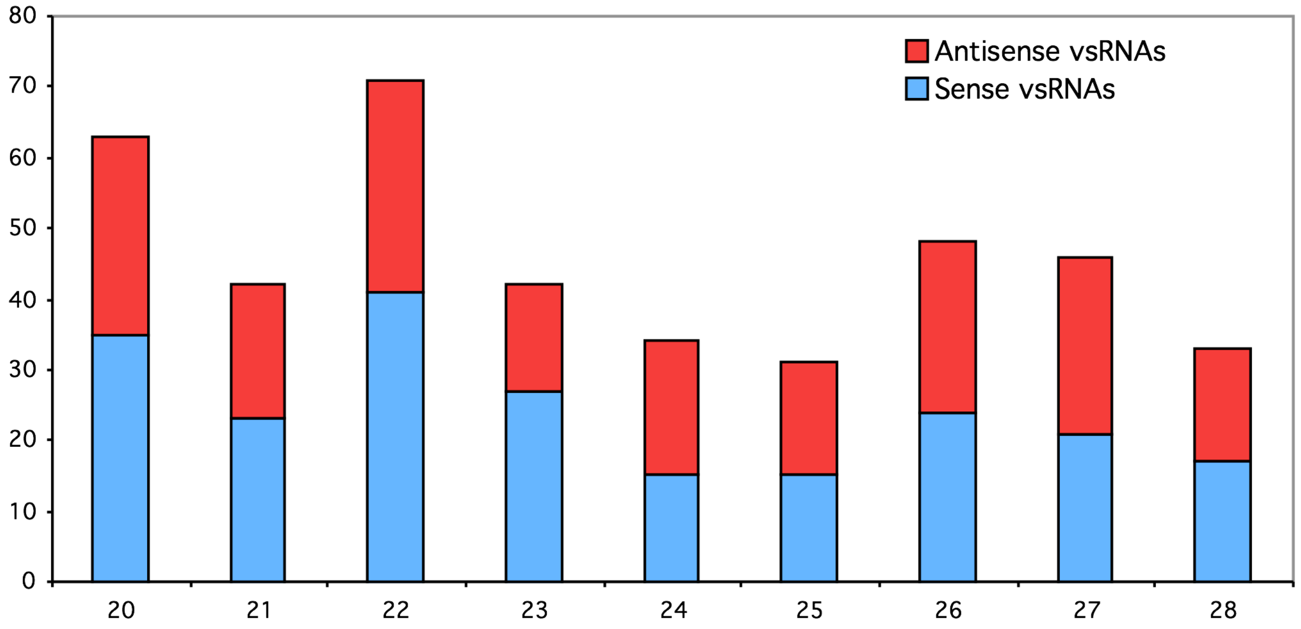

S2.n: Hepatitis C Replicon-derived vsRNAs from RP7; Ago-4 IP: Solexa data (5' P-dependent cloning; Sol-35)

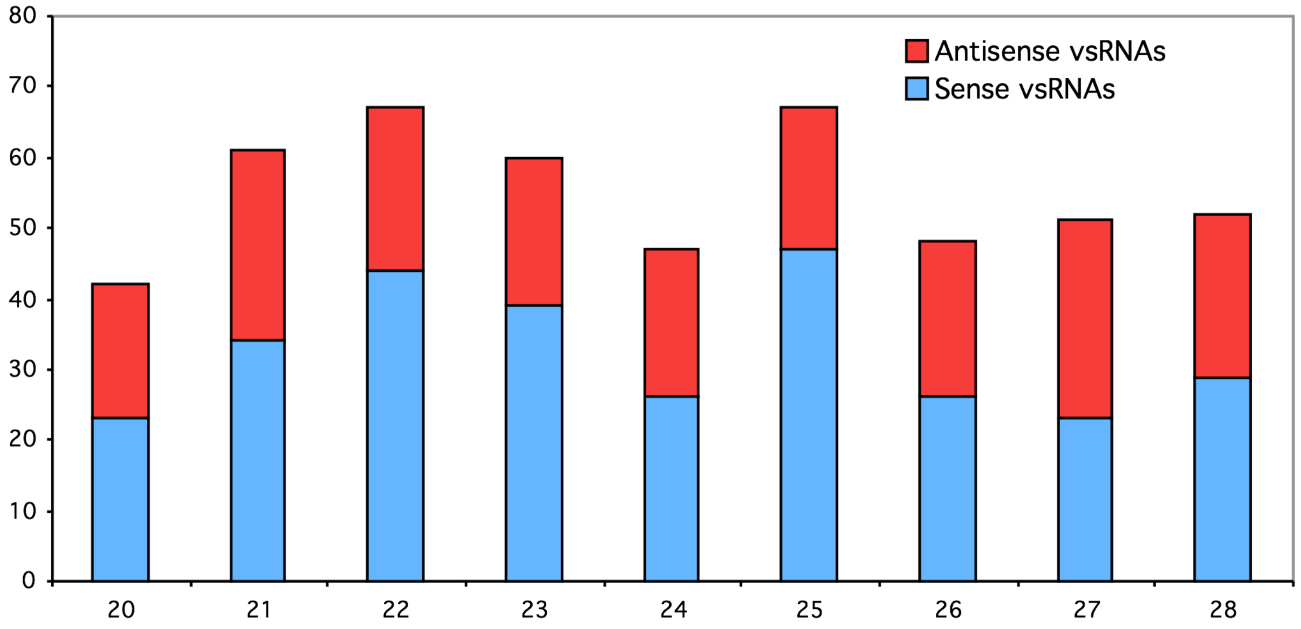

S2.o: Hepatitis C Replicon-derived vsRNAs from RP7; Mock IP: Solexa data (5' P-dependent cloning; Sol-36)

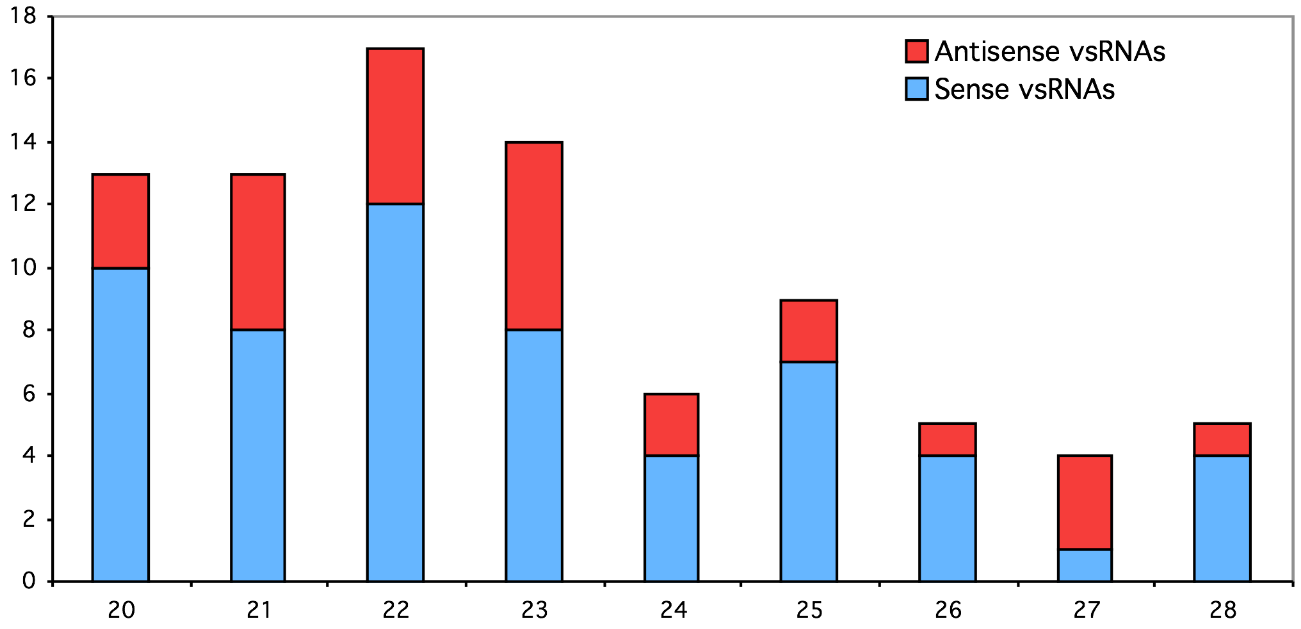

S2.p: Hepatitis C Replicon-derived vsRNAs from RP7; Ago-1 totRNA: Solexa data (5' P-dependent cloning; Sol-37)

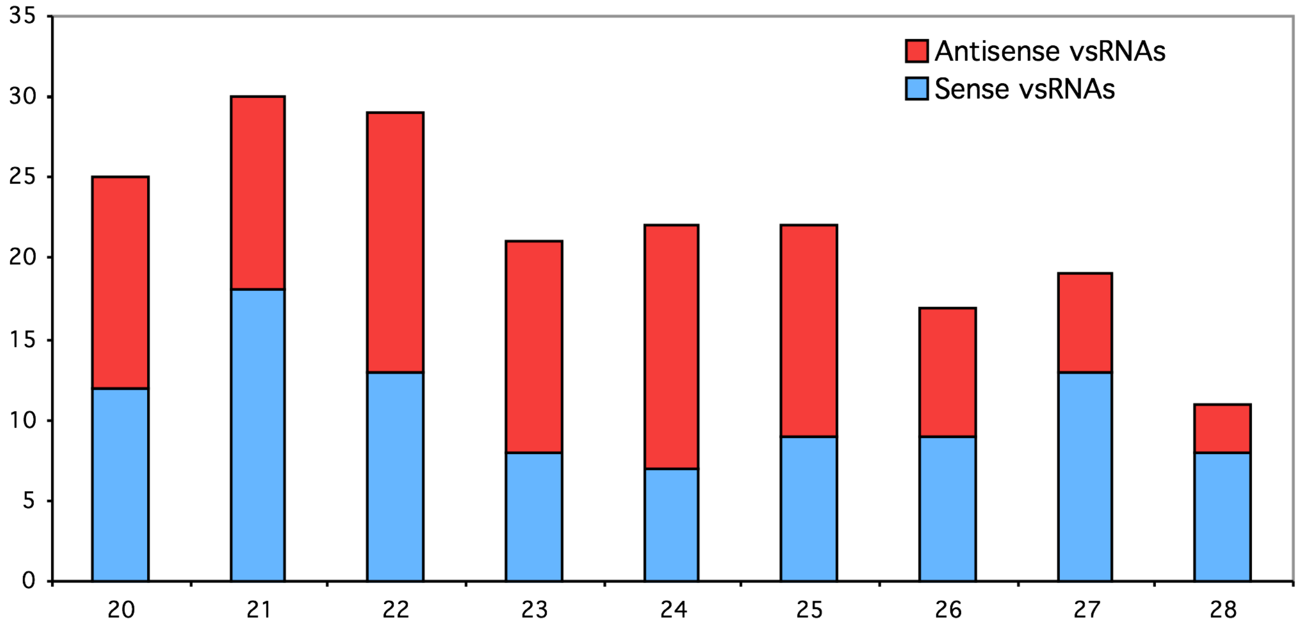

S2.q: Hepatitis C Replicon-derived vsRNAs from RP7; Ago-2 totRNA: Solexa data (5' P-dependent cloning; Sol-49)

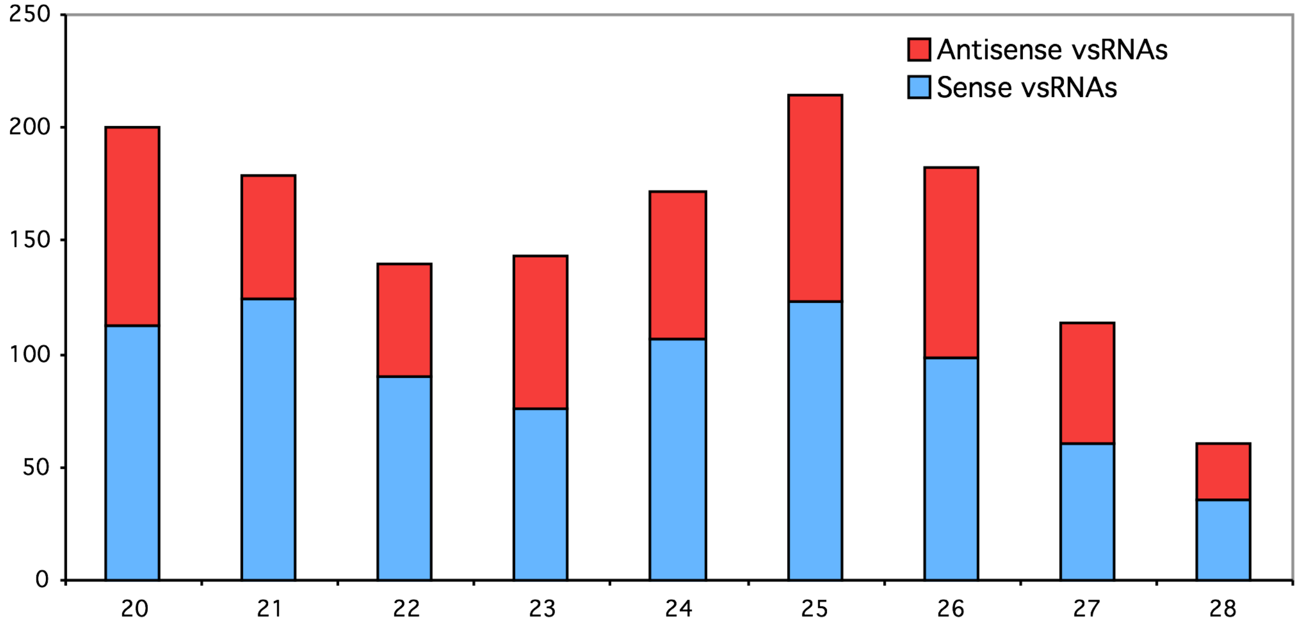

S2.r: Hepatitis C Replicon-derived vsRNAs from RP7; Ago-3 totRNA: Solexa data (5' P-dependent cloning; Sol-39)

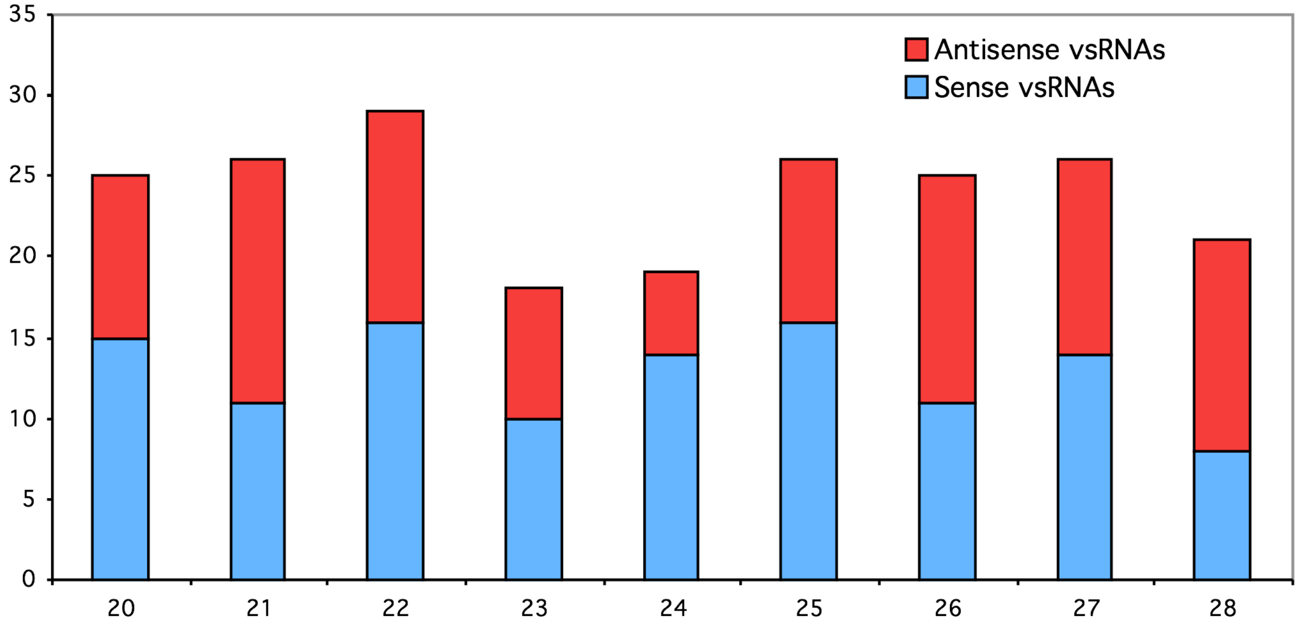

S2.s: Hepatitis C Replicon-derived vsRNAs from RP7; Ago-4 totRNA: Solexa data (5' P-dependent cloning; Sol-40)

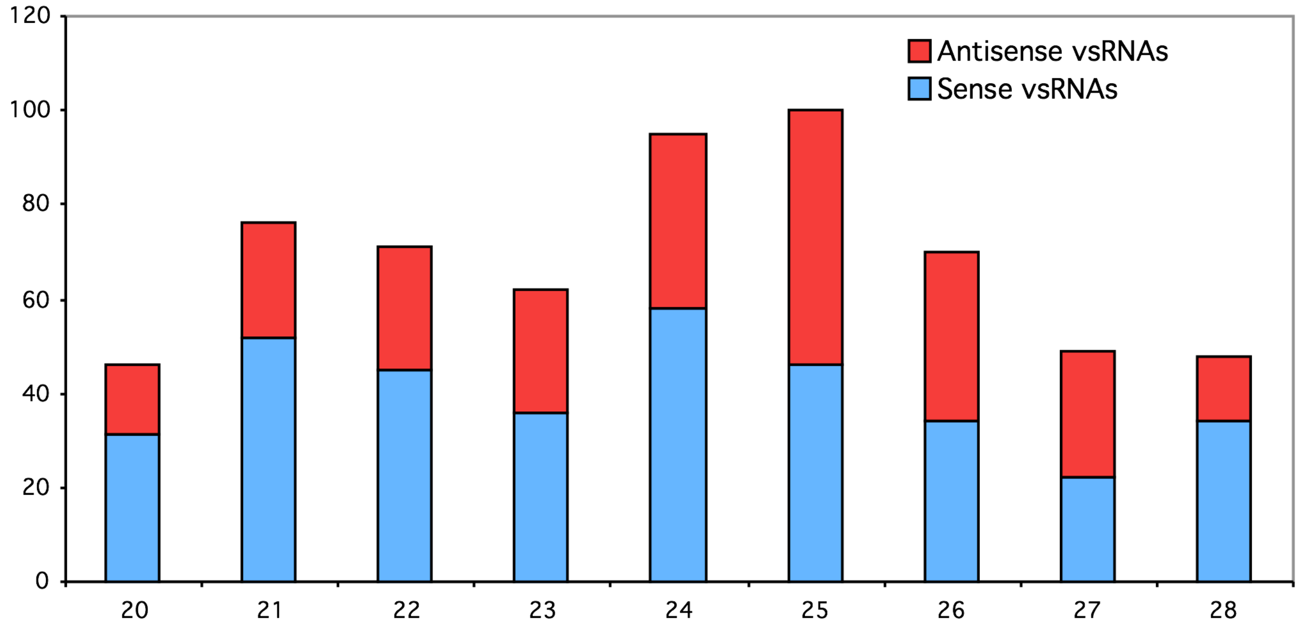

S2.t: Hepatitis C Virus (Infectious clone) vsRNAs: Cumulative 454 data (5'-P-dependent cloning)

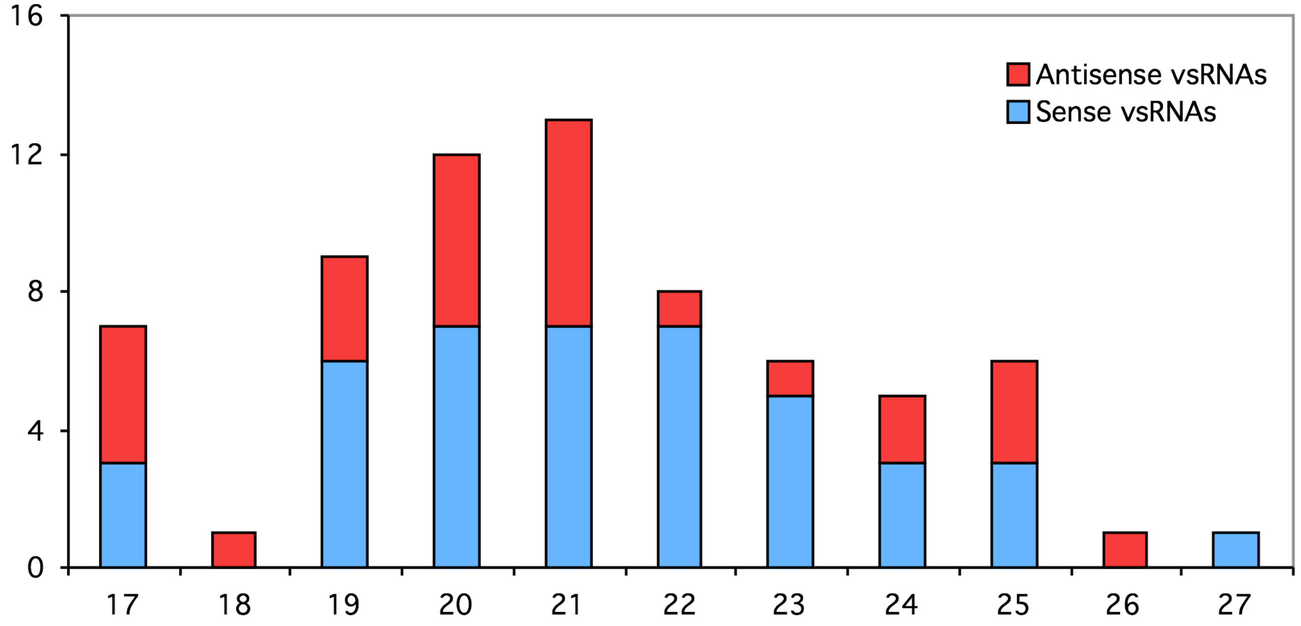

S2.u: Hepatitis C Virion-derived vsRNAs from Huh7.5; 5dpi: Solexa data (5’ P-dependent cloning; Sol-3)

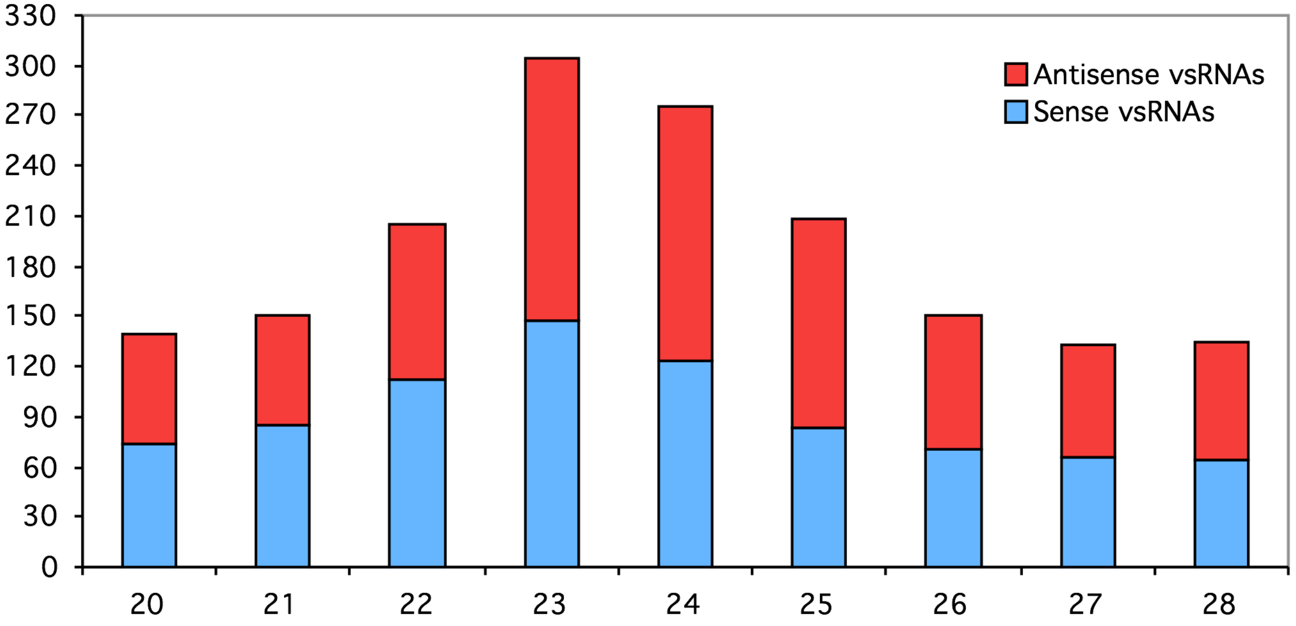

S2.v: Hepatitis C Virion-derived vsRNAs from Huh7.5; 5dpi: Solexa data (5’ P-INDependent cloning; Sol-76)

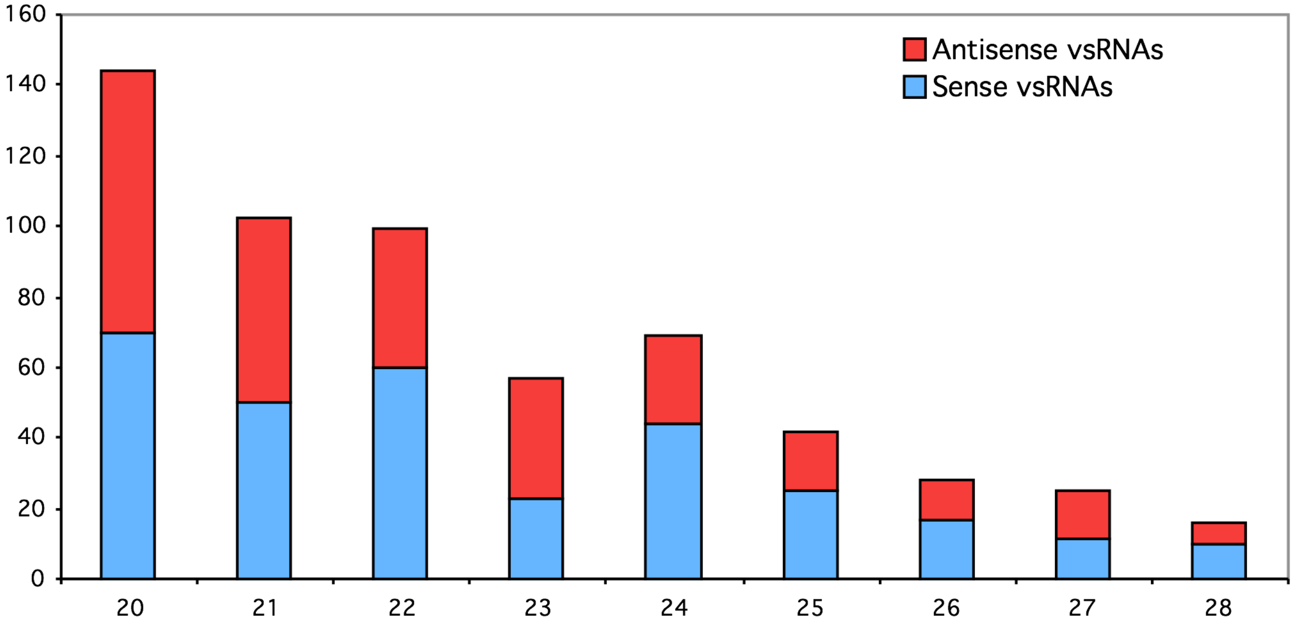

S2.w: Hepatitis C Virion-derived vsRNAs from Huh7.5 3dpi: Solexa data (5' P-dependent cloning; Sol-92)

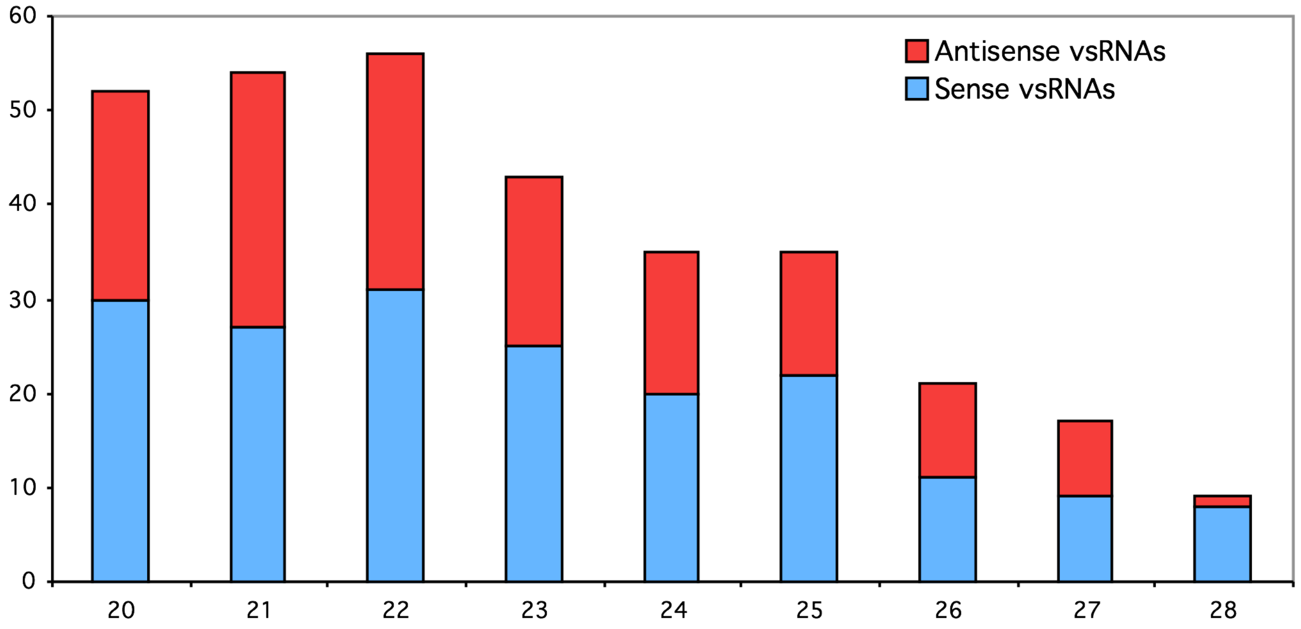

S2.x: Hepatitis C Virion-derived vsRNAs from Huh7.5 6dpi: Solexa data (5' P-dependent cloning; Sol-93)

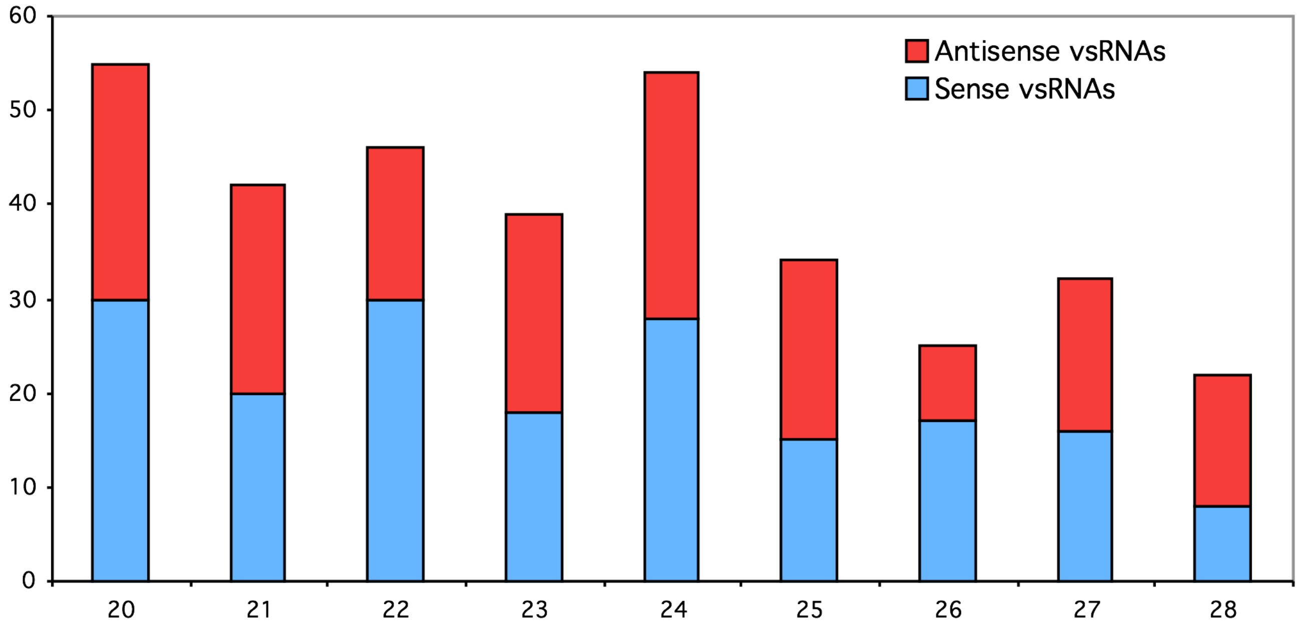

S2.y: Hepatitis C Virion-derived vsRNAs from Huh7.5 9dpi: Solexa data (5' P-dependent cloning; Sol-94)

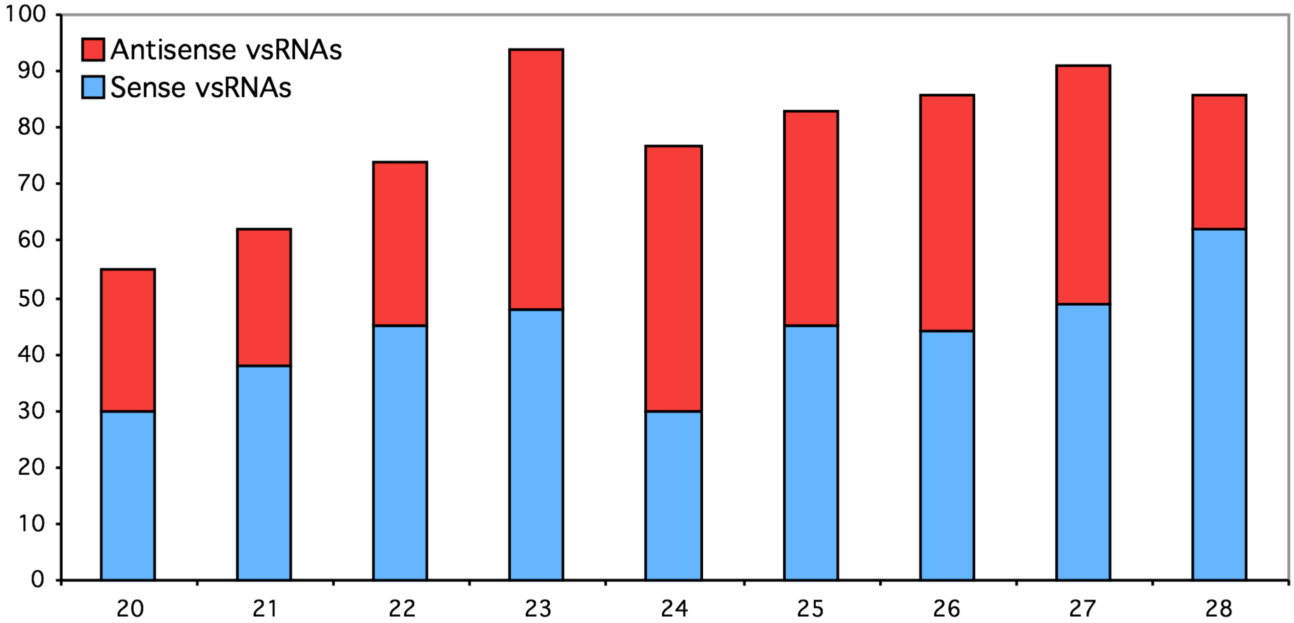

S2.z: Hepatitis C Virion-derived vsRNAs from Huh7.5 11dpi: Solexa data (5' P-dependent cloning; Sol-95)

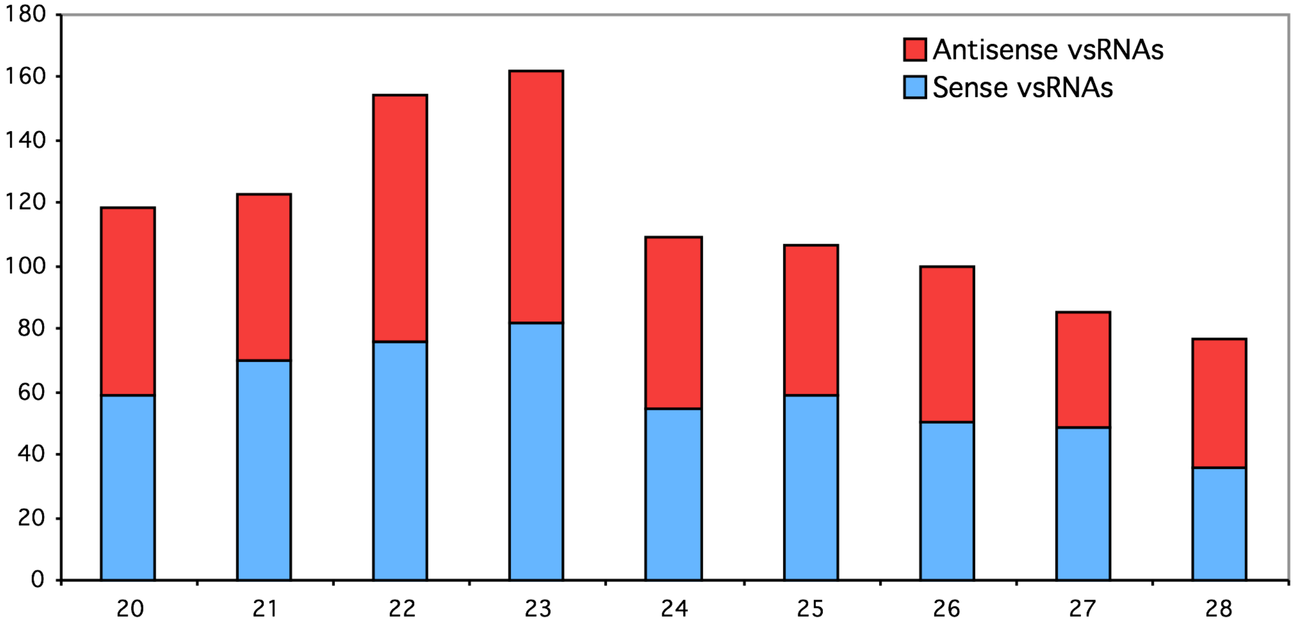

S2.aa: Hepatitis C Virion-derived vsRNAs from Huh7.5 15dpi: Solexa data (5' P-dependent cloning; Sol-96)

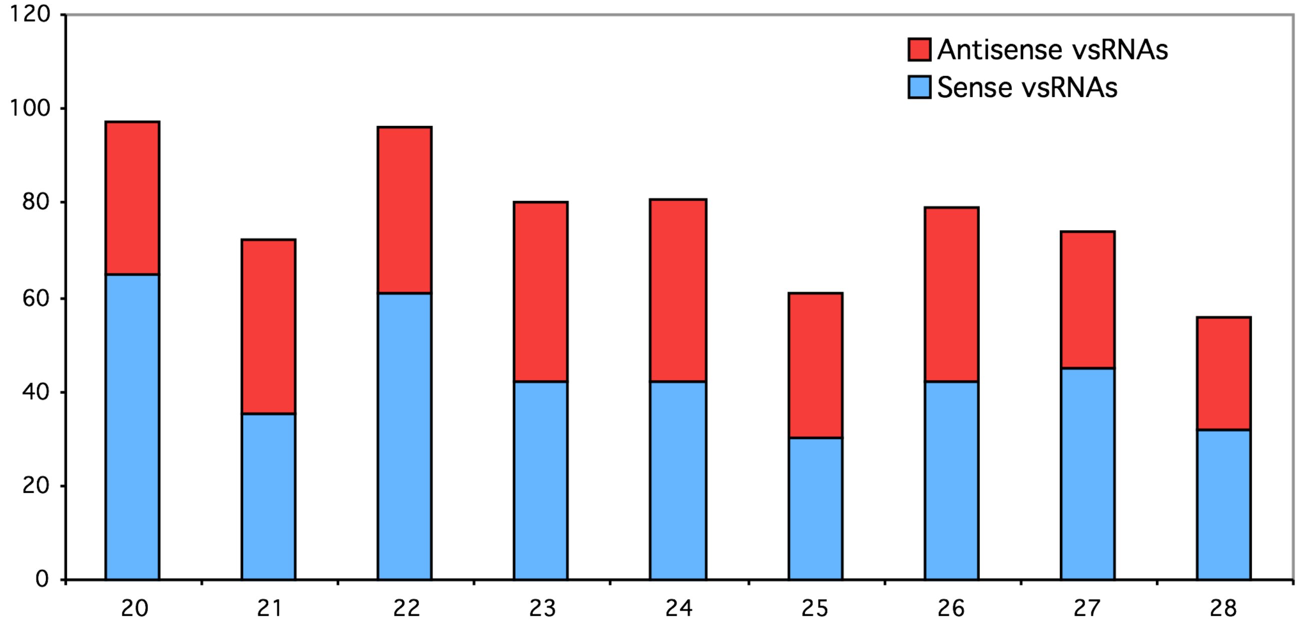

S2.bb: Poliovirus vsRNAs: Cumulative 454 data (5'-P-dependent cloning)

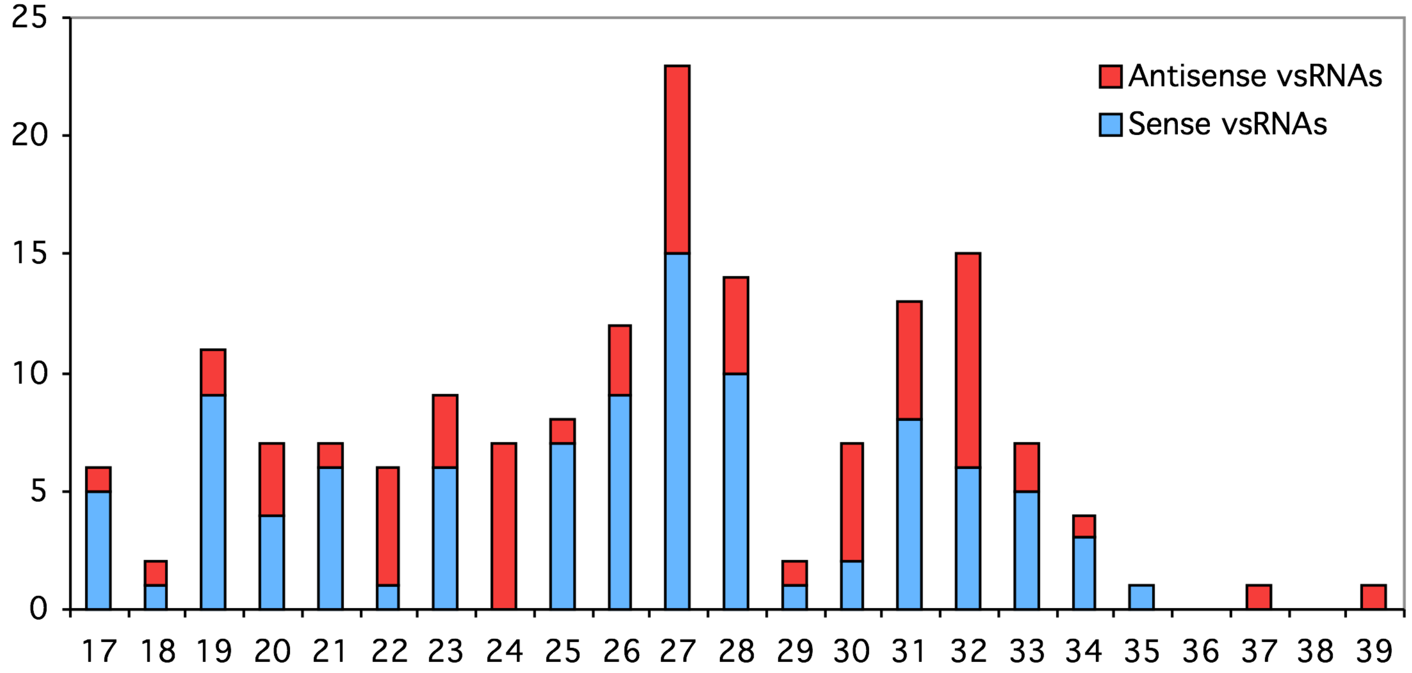

S2.cc: Poliovirus vsRNAs: Cumulative 454 data (5'-P-INDependent cloning)

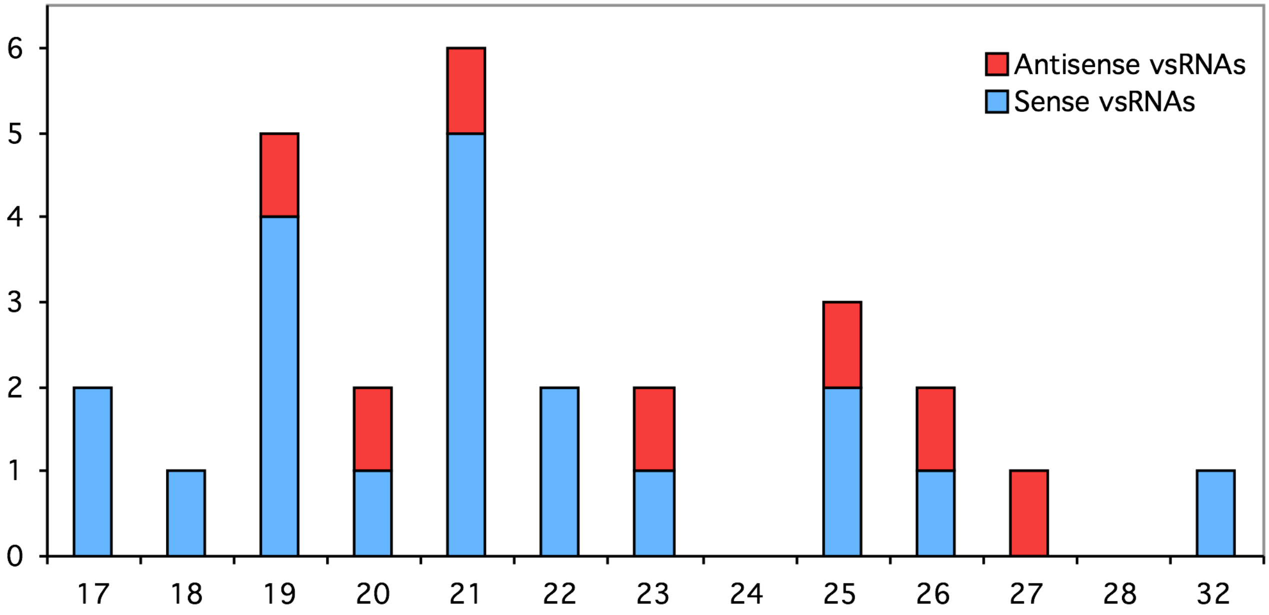

S2.dd: Poliovirus vsRNAs: Solexa data (5'-P-dependent cloning; Hela cells; 5.5hpi; Sol-2)

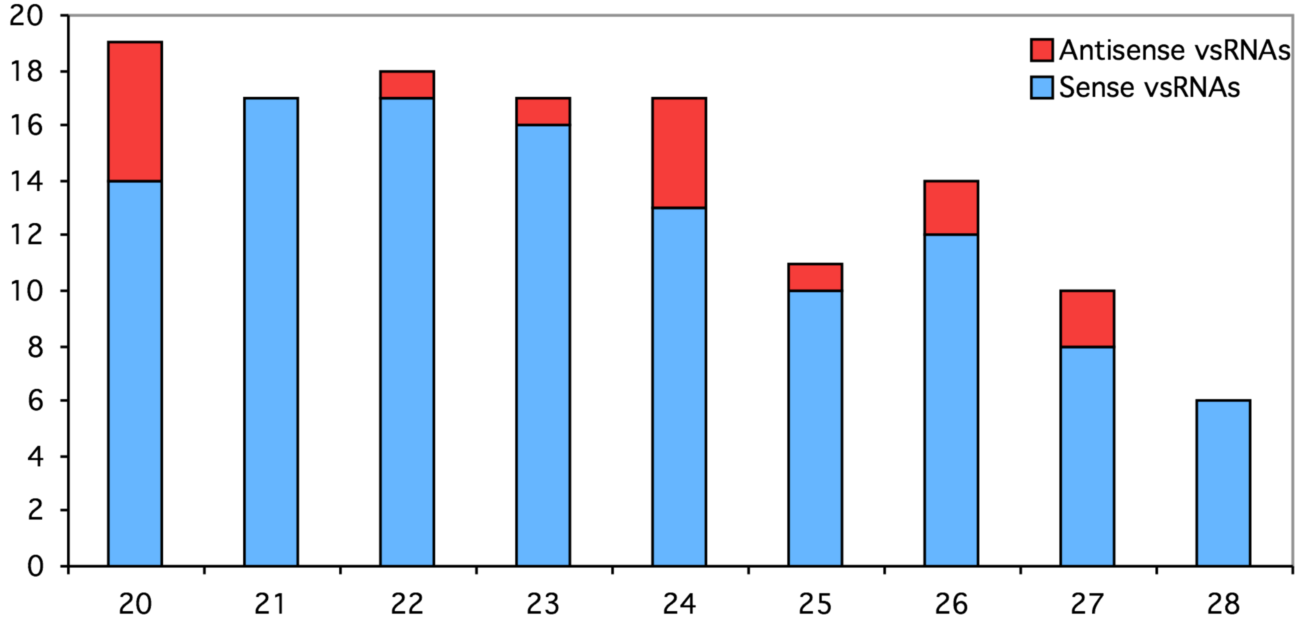

S2.ee: Polio-derived vsRNAs from *IFNabR*+/+ MEFs 6hpi: Solexa data (5’ P-dependent cloning; Sol-60)

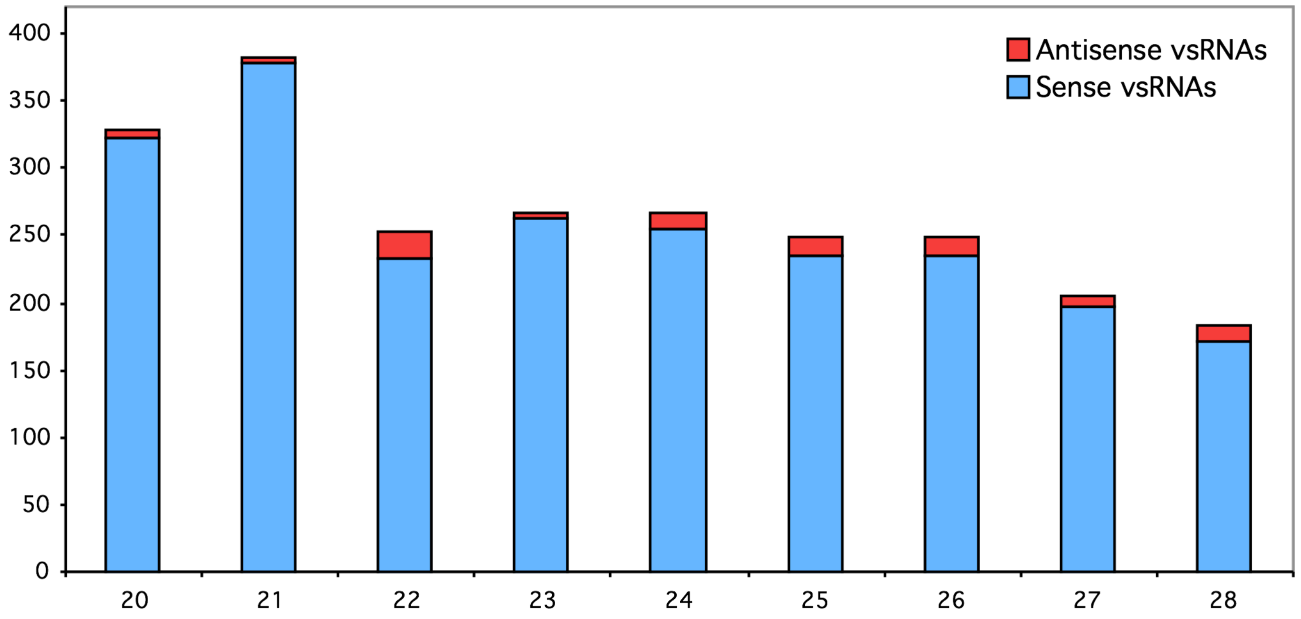

S2.ff: Polio-derived vsRNAs from *IFNabR*-/- MEFs 6hpi: Solexa data (5’ P-dependent cloning; Sol-62)

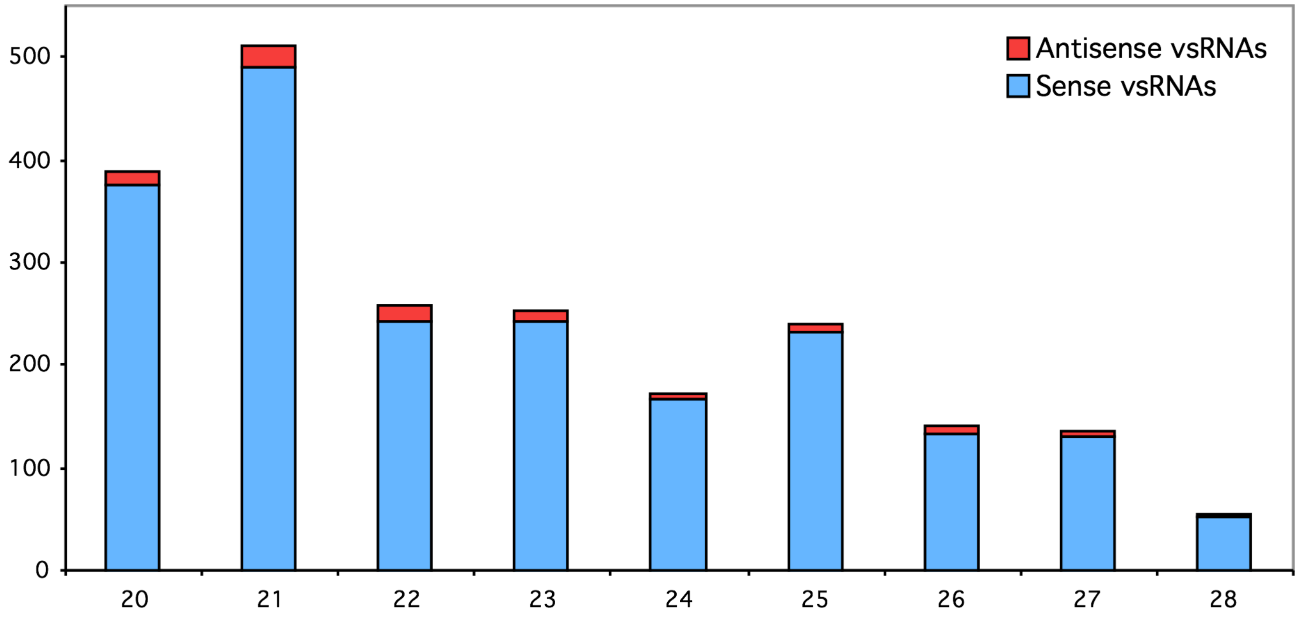

S2.gg: Polio-derived vsRNAs from *eri-1*+/+ MEFs 6hpi: Solexa data (5' P-dependent cloning; Sol-64)

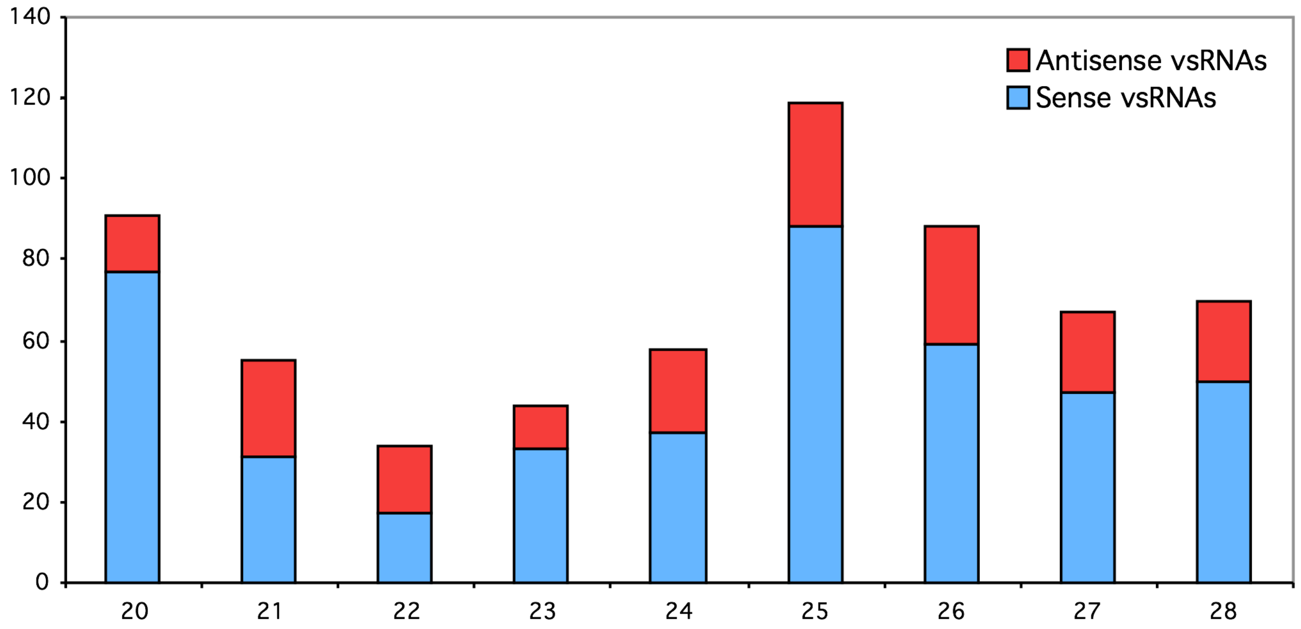

S2.hh: Polio-derived vsRNAs from *eri-1*-/- MEFs 6hpi: Solexa data (5' P-dependent cloning; Sol-66)

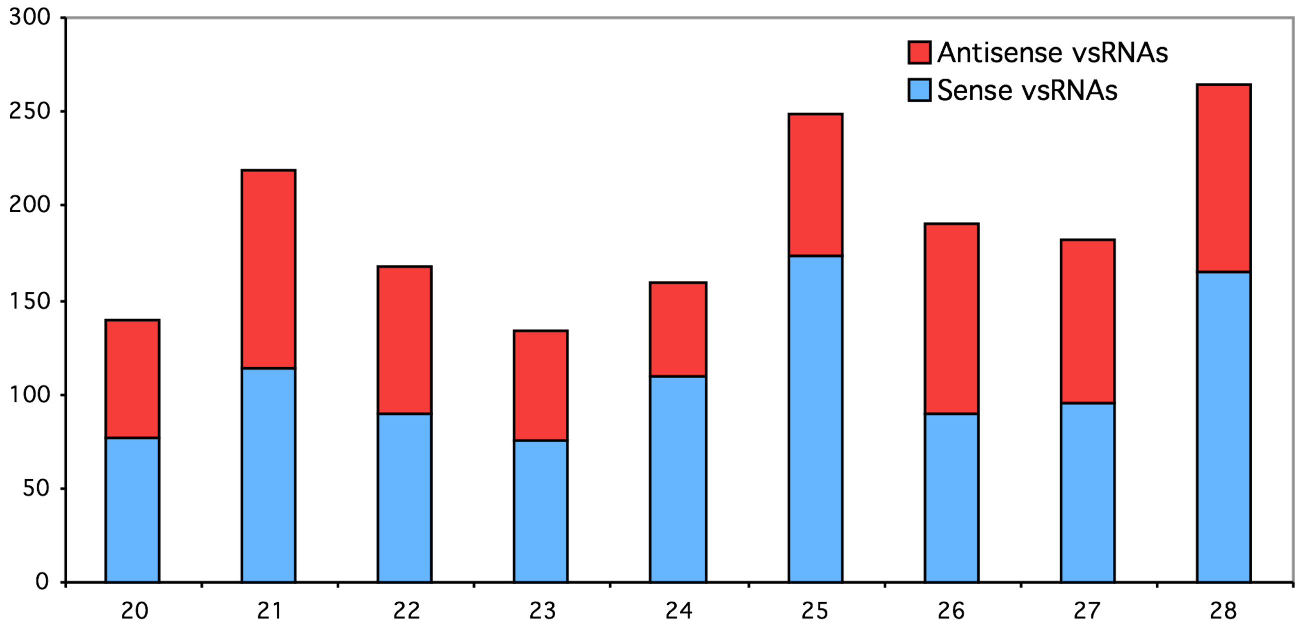

S2.ii: Polio-derived vsRNAs from *dcr-1*+/+ MEFs 6hpi: Solexa data (5' P-dependent cloning; Sol-68)

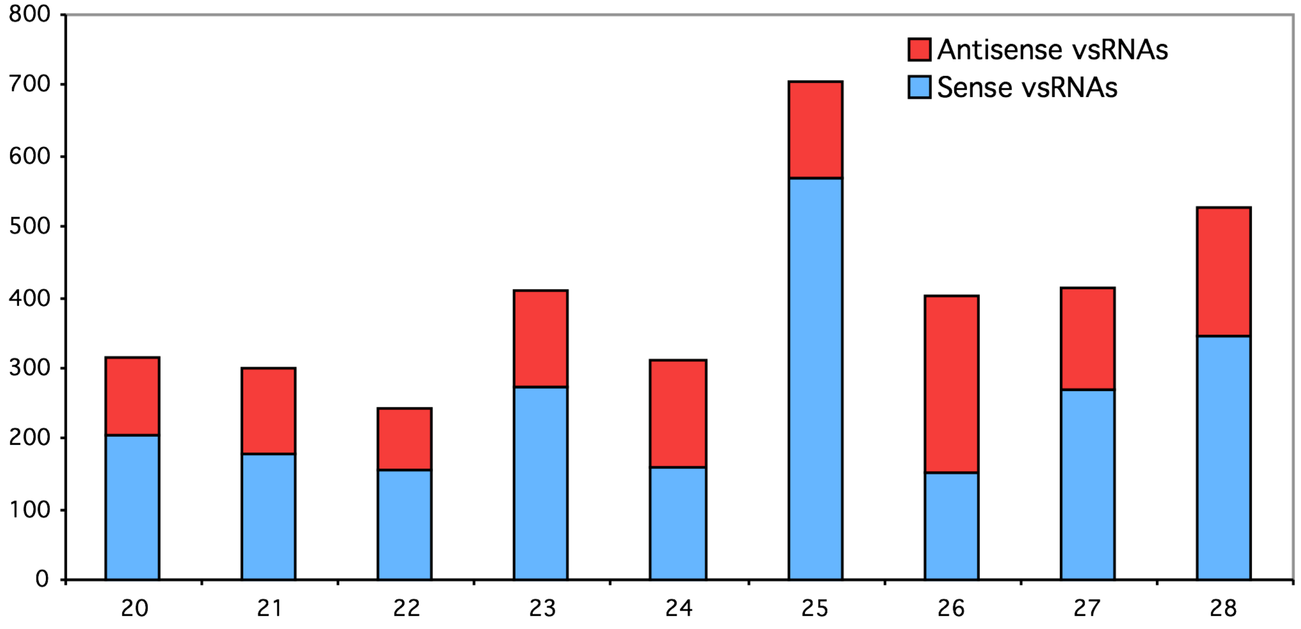

S2.jj: Polio-derived vsRNAs from *dcr-1*-/- MEFs 6hpi: Solexa data (5' P-dependent cloning; Sol-70)

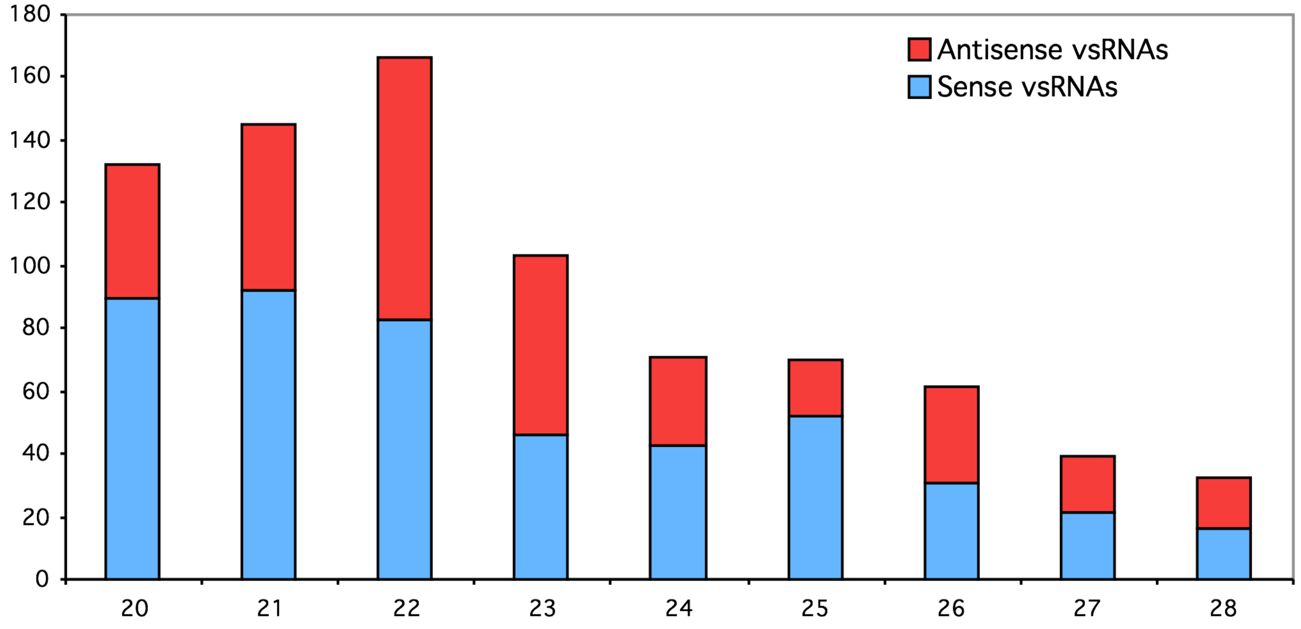

S2.kk: Polio-derived vsRNAs from *ago-2* +/+ MEFs 6hpi: Solexa data (5' P-INDependent cloning; Sol-79)

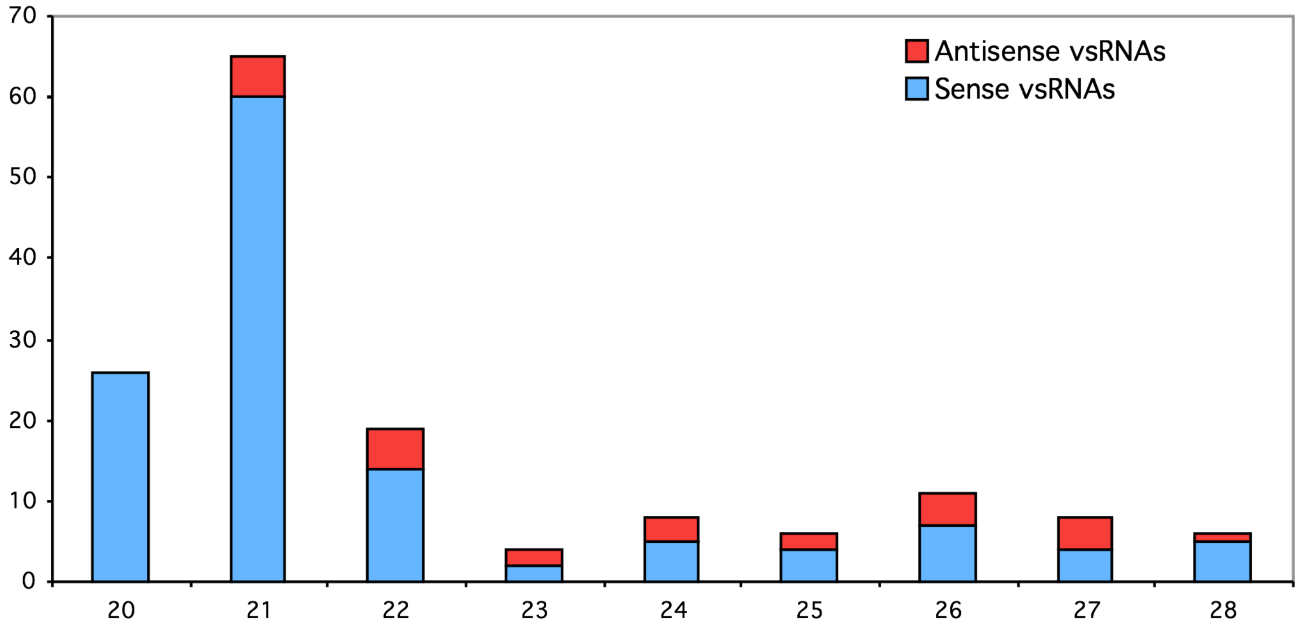

S2.ll: Polio-derived vsRNAs from *ago-2* -/- MEFs 6 hpi: Solexa data (5' P-INDependent cloning; Sol-80)

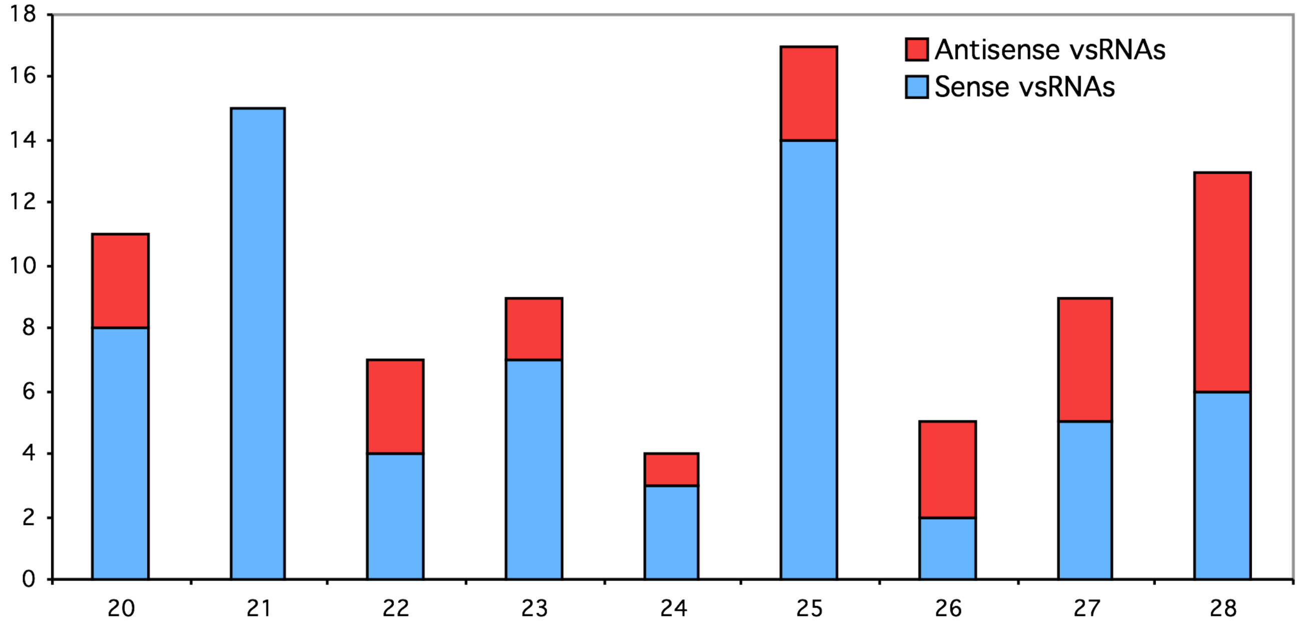

S2.mm: Polio-derived vsRNAs from *ago-2* +/+ MEFs 6 hpi: Solexa data (5' P-dependent cloning; Sol-82)

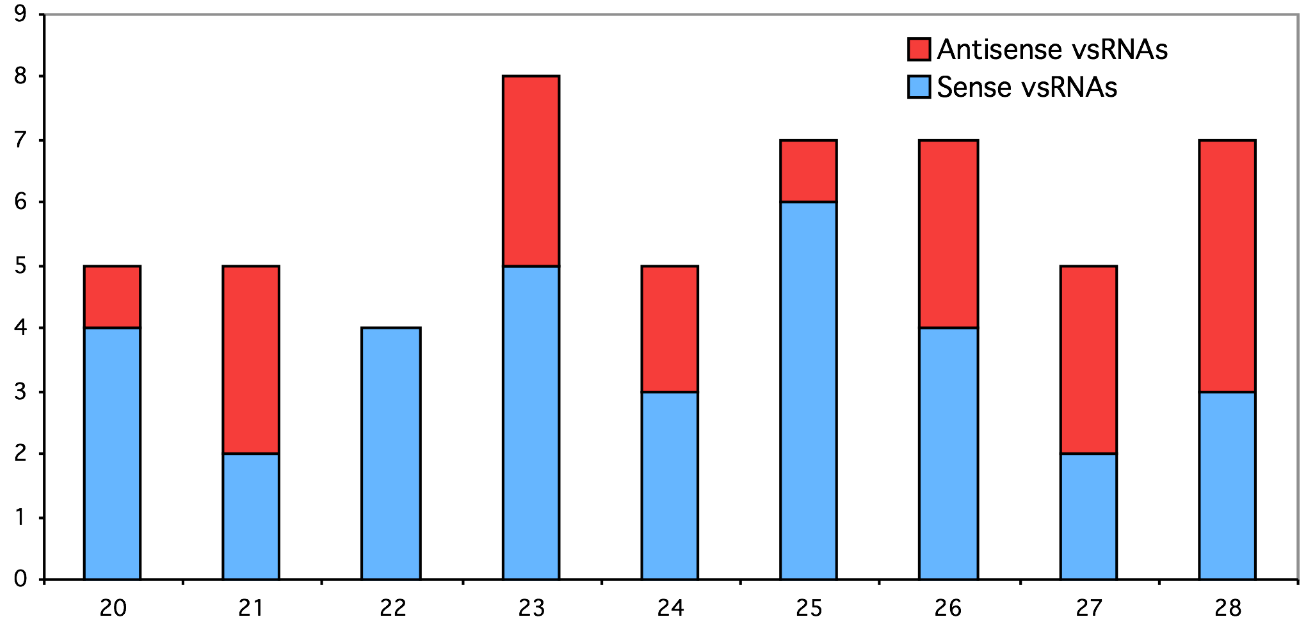

S2.nn: Polio-derived vsRNAs from *ago-2* -/- MEFs 6 hpi: Solexa data (5' P-dependent cloning; Sol-83)

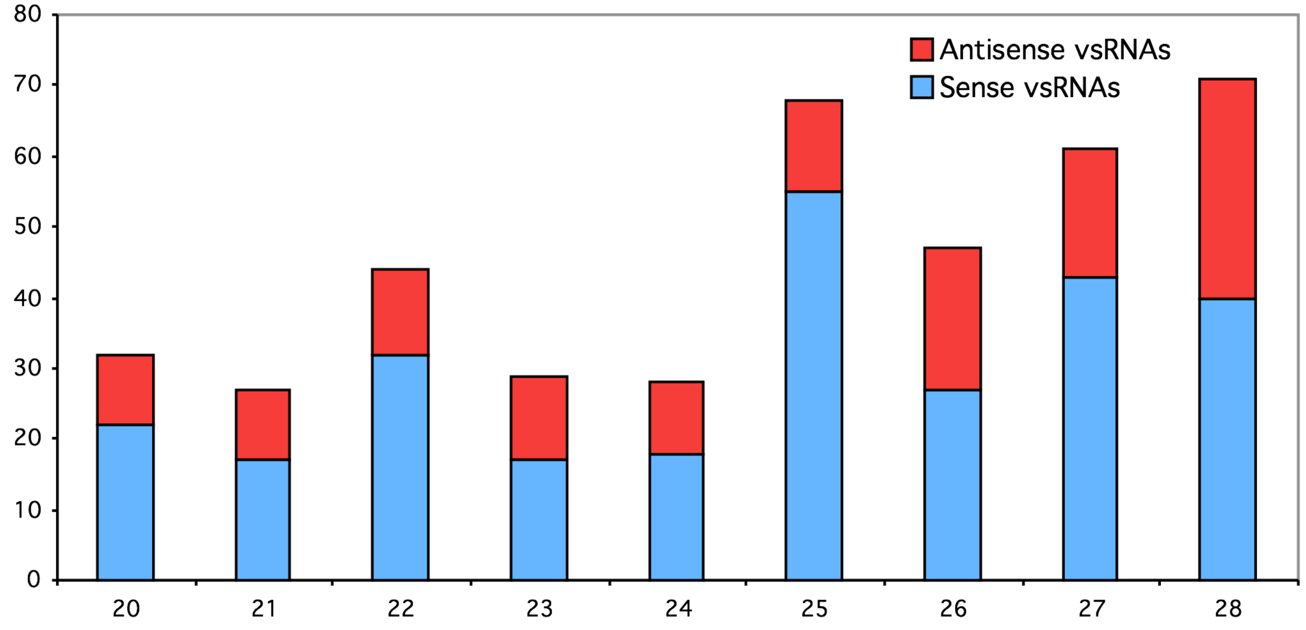

S2.oo: Poliovirus vsRNAs: Solexa data (5'-P-dependent cloning; IFNαβR-/- mice; muscle; Sol-1)

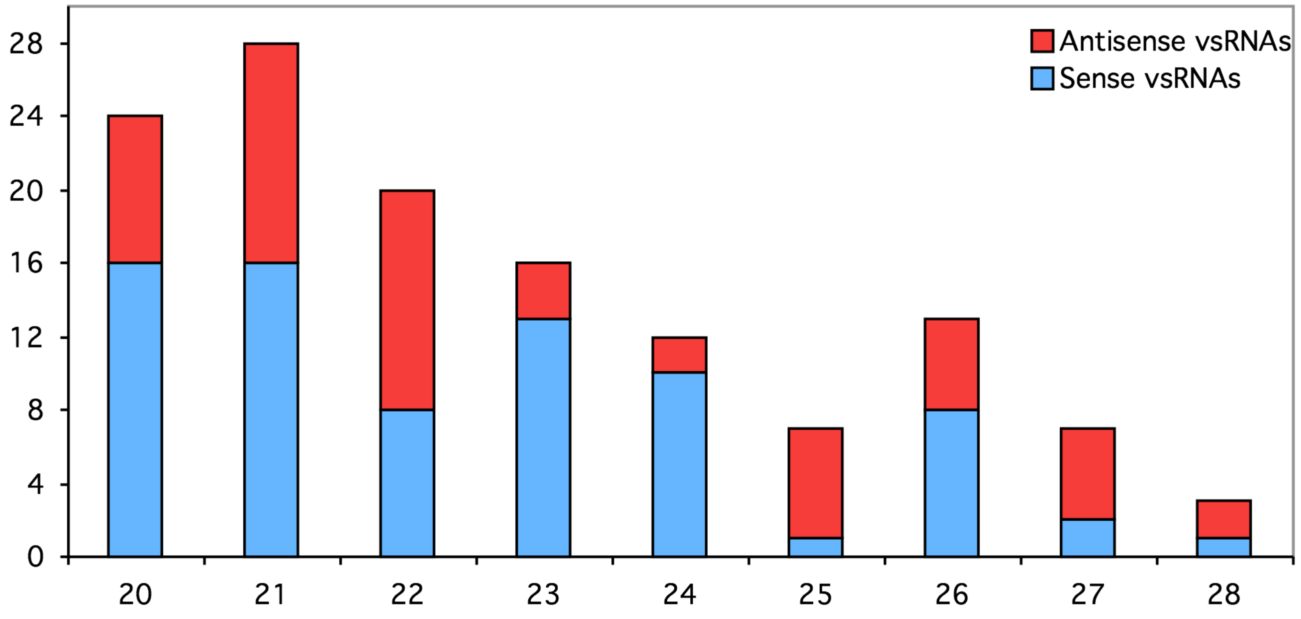

S2.pp: Polio-derived vsRNAs from *M. musculus* (*IFN*-/-; *PVR* +/+) Brain: Solexa data (5' P-INDependent cloning; Sol-77)

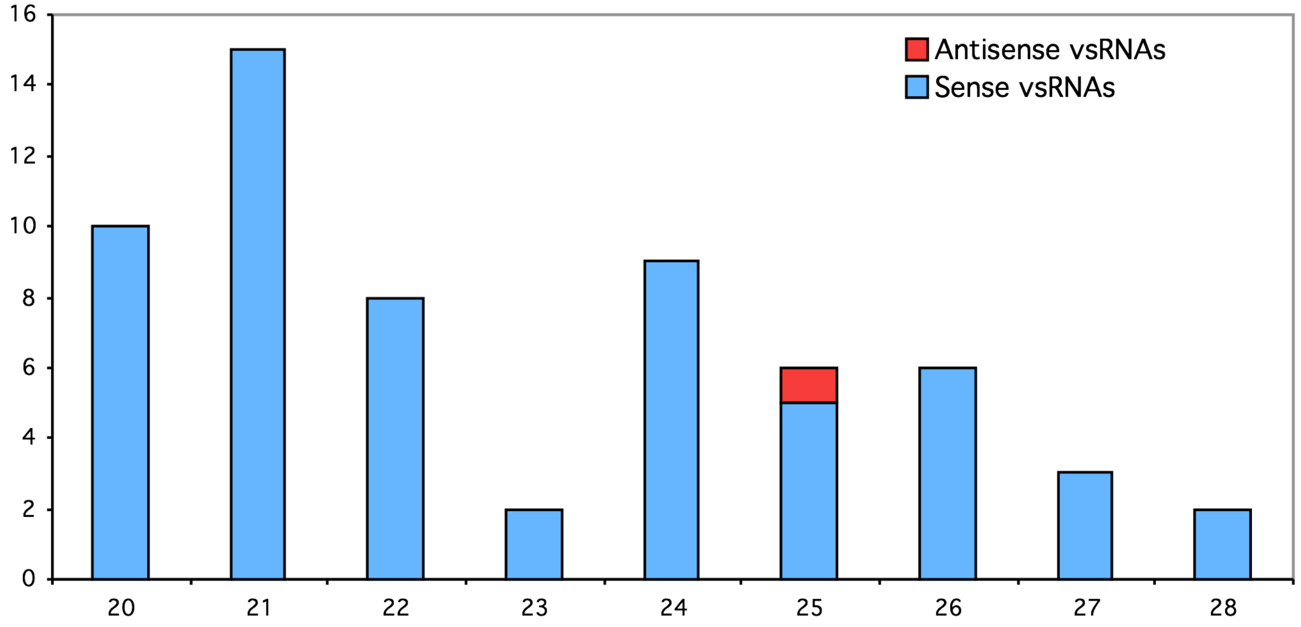

S2.qq: Polio-derived vsRNAs from *M. musculus* (*IFN*-/-; *PVR* +/+) Brain: Solexa data (5' P-dependent cloning; Sol-81)

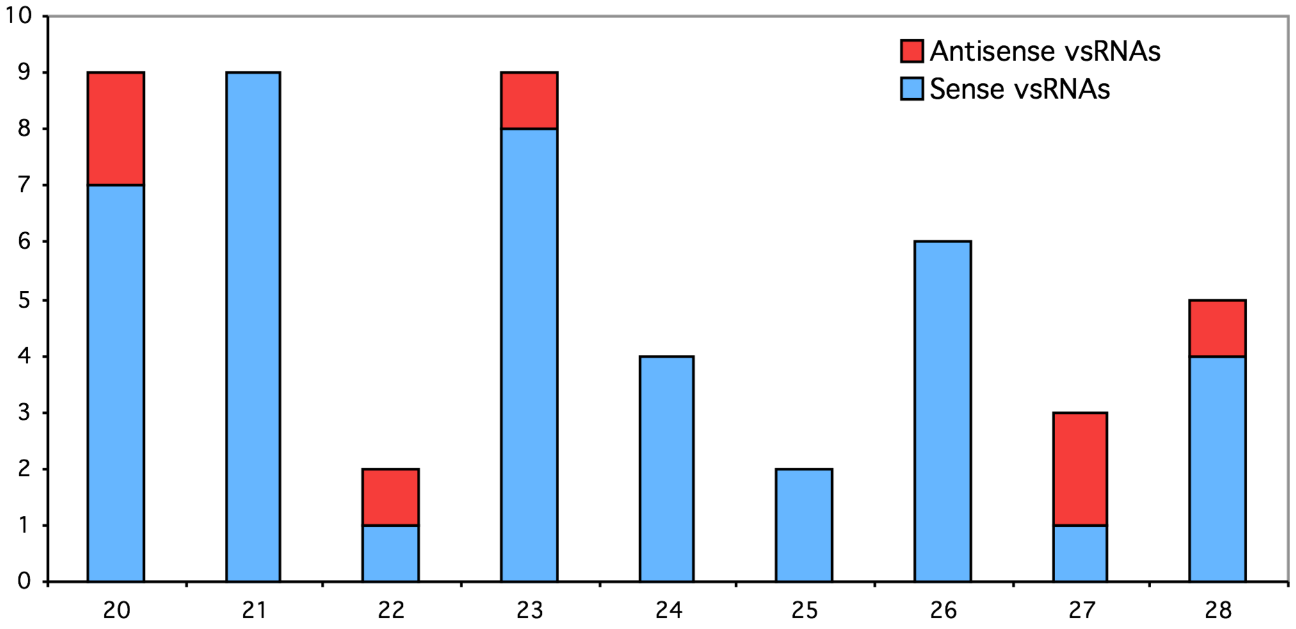

S2.rr: Polio-derived vsRNAs from *M. musculus* (*IFN*-/-; *PVR* +/+) Muscle: Solexa data (5' P-INDependent cloning; Sol-78)

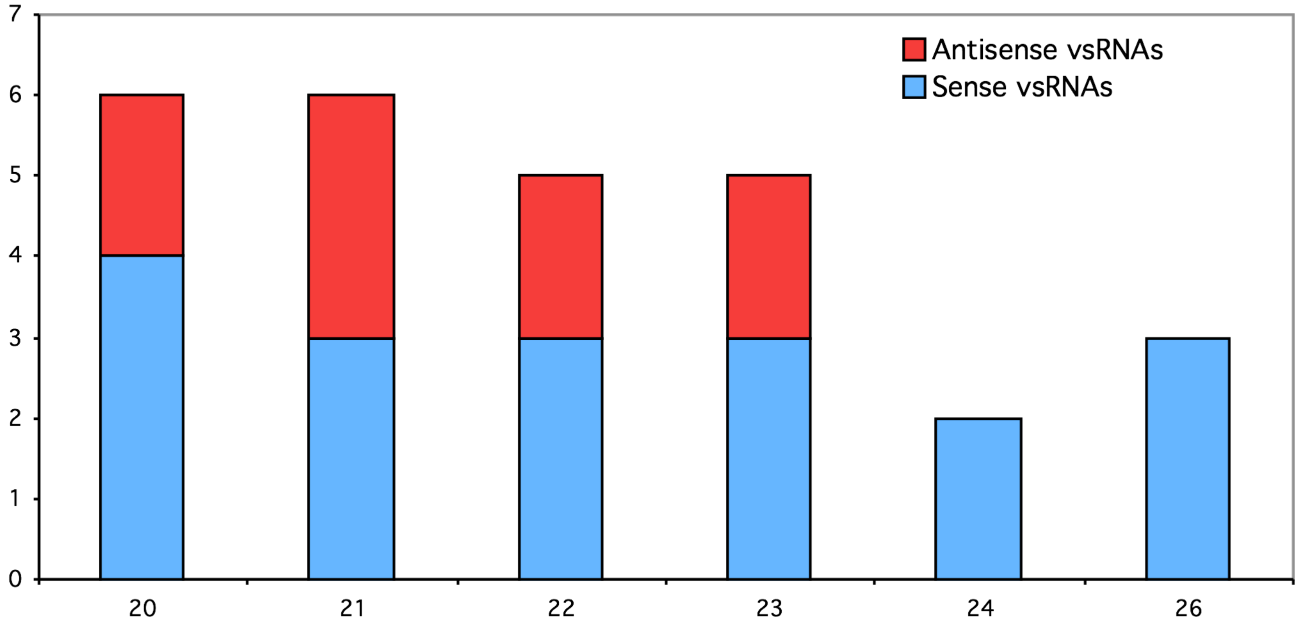

S2.ss: Vesicular Stomatitis Virus vsRNAs: Cumulative 454 data (5'-P-dependent cloning)

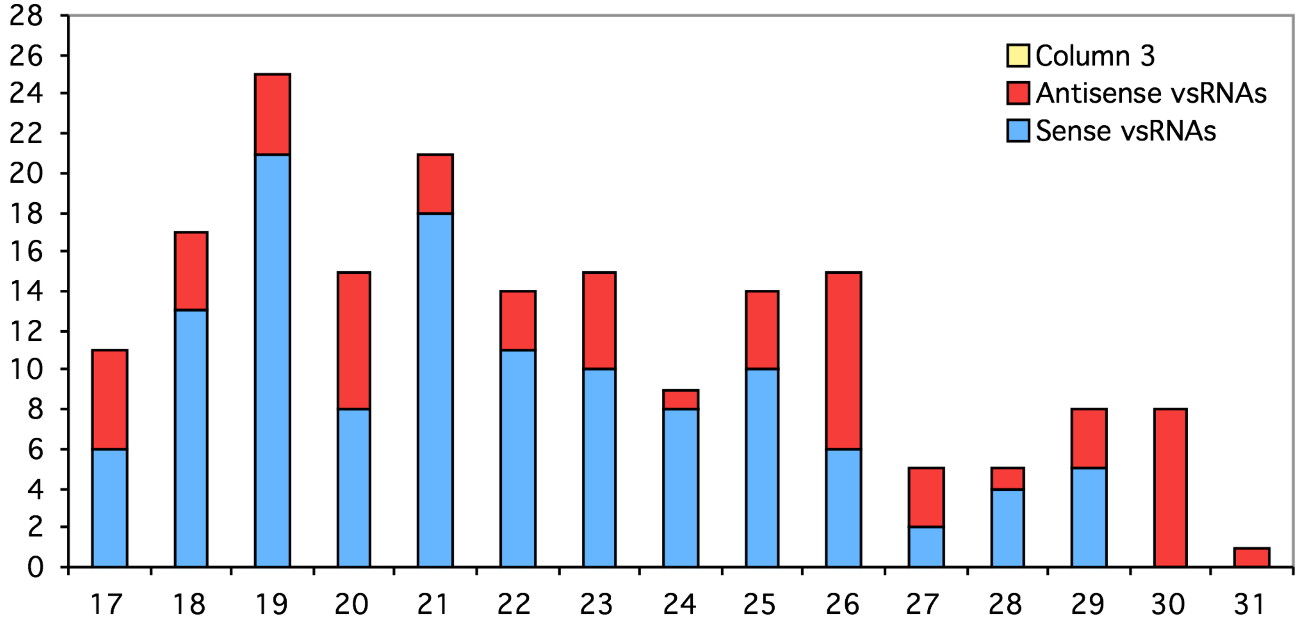

S2.tt: West Nile Virus vsRNAs: Cumulative 454 data (5'-P-dependent cloning)

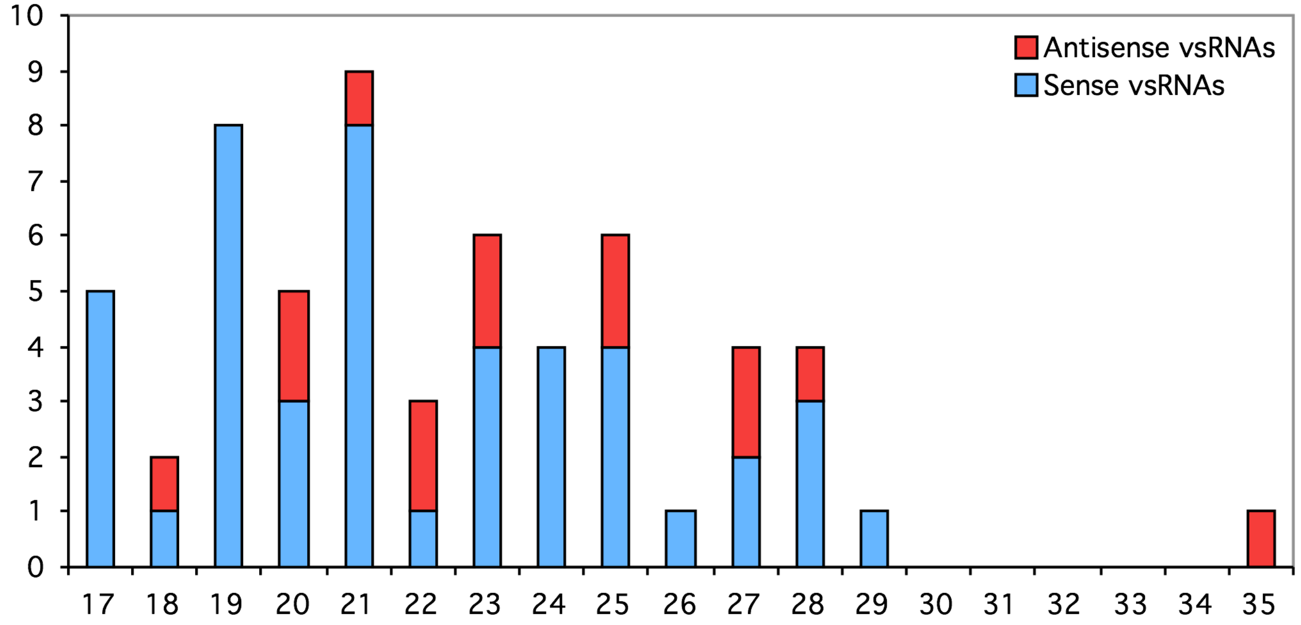

Supplement: Figure S2 — Frequency-Length profiles for vsRNAs. X-axis: vsRNA lengths; Y-axis: number of vsRNA instances. (2.09 MB PDF) [file ppat.1000764.s003.pdf]
