# Supplementary material for: Six RNA Viruses and Forty-One Hosts: Viral Small RNAs and Modulation of Small RNA Repertoires in Vertebrate and Invertebrate Systems
Source: PLoS Pathog. 2010 Feb 12;6(2):e1000764. doi: 10.1371/journal.ppat.1000764 (PMC2820531; doi:10.1371/journal.ppat.1000764)

S3A.

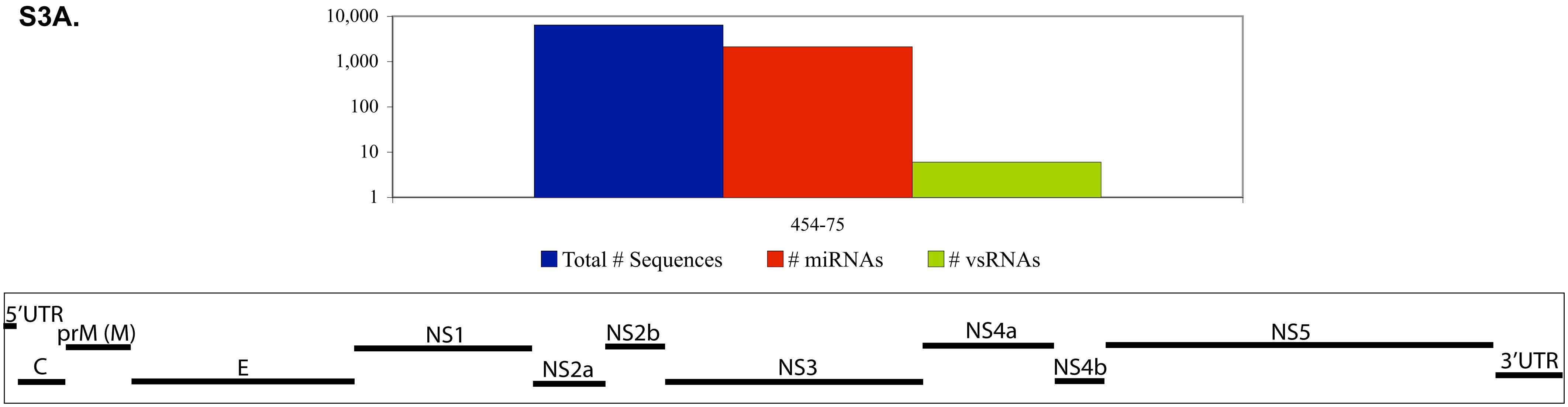

S3B.

454-75: Dengue Virus-derived vsRNAs from Huh7 cells. 5'-P-dep cloning. # of sequences: miRNAs (3569), (+) vsRNAs (6), (-) vsRNAs (0), Total (8387)

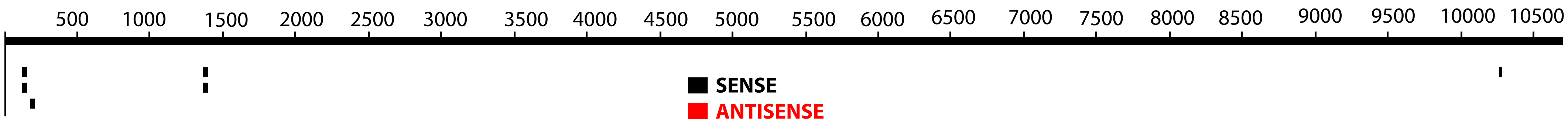

Supplement: Figure S3 — Rare Dengue-derived vsRNAs are detectable in certain host backgrounds. (S3A) Sequence count: all RNAs, miRNAs, vsRNAs (Y-axis: log scale). (S3B) vsRNAs with a 5′ monophosphate moiety from Huh7 cells infected with DENV-2, 25 h.p.i. (Sample: 454-75). (0.23 MB PDF) [file ppat.1000764.s004.pdf]
