# Supplementary material for: Six RNA Viruses and Forty-One Hosts: Viral Small RNAs and Modulation of Small RNA Repertoires in Vertebrate and Invertebrate Systems
Source: PLoS Pathog. 2010 Feb 12;6(2):e1000764. doi: 10.1371/journal.ppat.1000764 (PMC2820531; doi:10.1371/journal.ppat.1000764)

S5A.

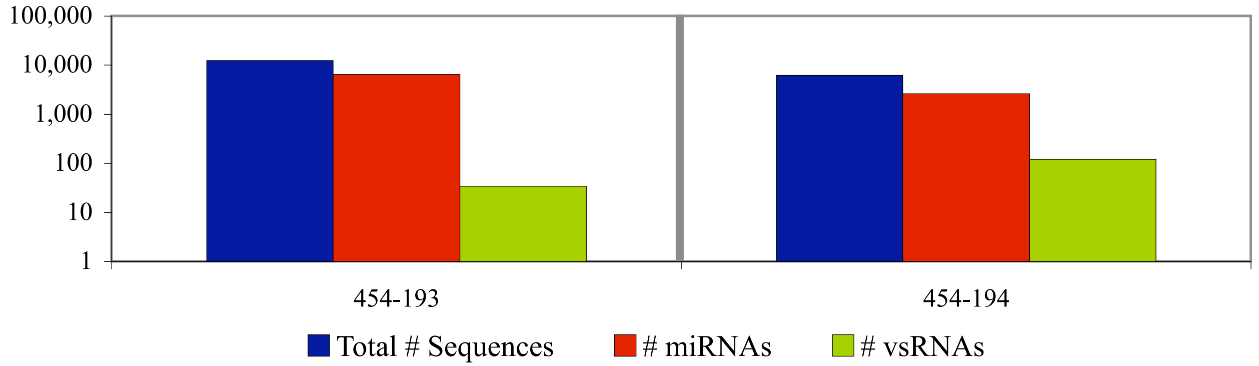

POLIOVIRUS

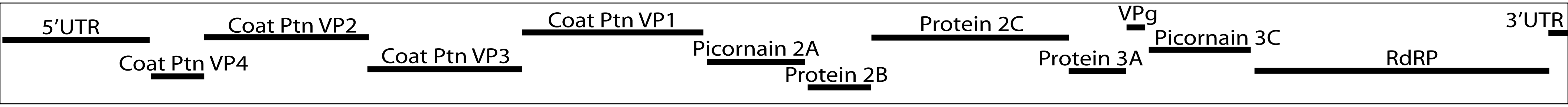

S5B.

454-193: Poliovirus vsRNAs from *ago-2*+/+ MEFs. 5′-P-dep cloning. # of sequences: miRNAs (7511), (+) vsRNAs (24), (-) vsRNAs (11), Total (13773)

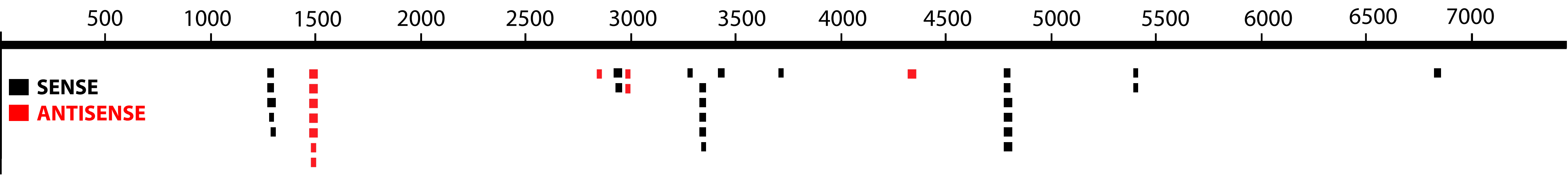

S5C.

454-194: Poliovirus vsRNAs from *ago-2*-/- MEFs. 5′-P-dep cloning. # of sequences: miRNAs (2880), (+) vsRNAs (65), (-) vsRNAs (48), Total (5871)

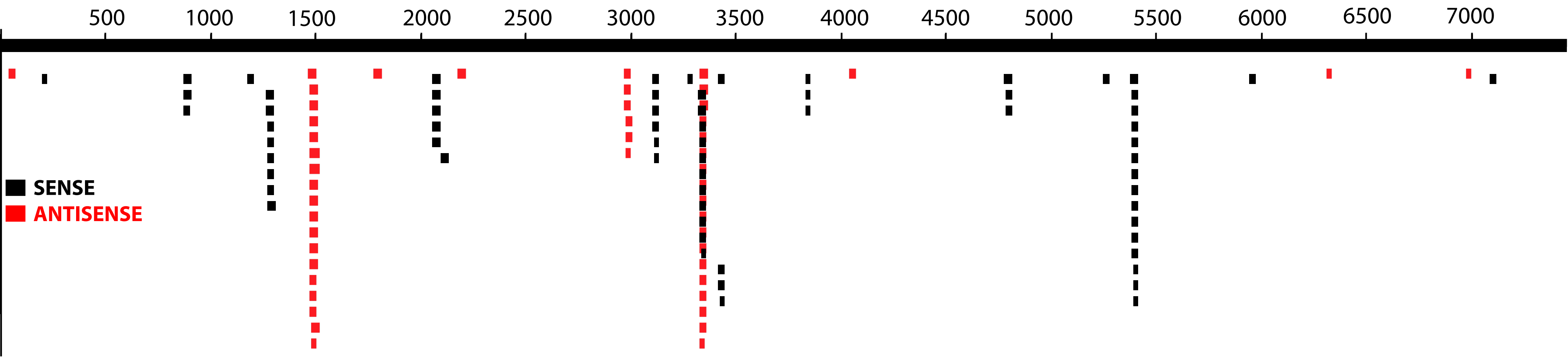

Supplement: Figure S5 — (Profiles from GS-20/FLX-sequenced libraries) Poliovirus vsRNAs are more abundant in MEFs deficient in Argonaute-2. (S5A) Sequence count: all RNAs, miRNAs, vsRNAs (Y-axis: log scale). vsRNAs with a 5′-monophosphate moiety from (S5B) ago-2+/+ MEFs (Sample: 454-193) and (S5C) ago-2−/− MEFs (Sample: 454-194) transfected with a plasmid encoding for self-replicating full-length Poliovirus RNA. (0.29 MB PDF) [file ppat.1000764.s006.pdf]
