# Supplementary material for: Six RNA Viruses and Forty-One Hosts: Viral Small RNAs and Modulation of Small RNA Repertoires in Vertebrate and Invertebrate Systems
Source: PLoS Pathog. 2010 Feb 12;6(2):e1000764. doi: 10.1371/journal.ppat.1000764 (PMC2820531; doi:10.1371/journal.ppat.1000764)

S8A.

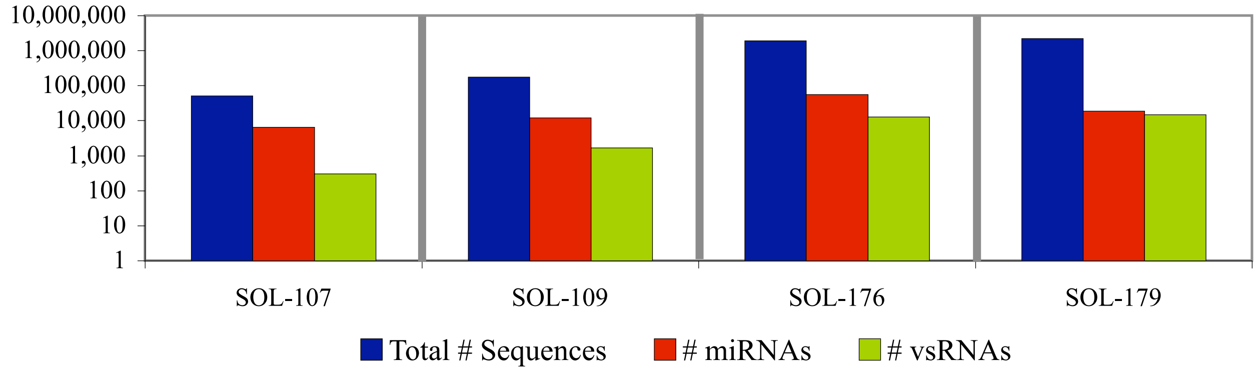

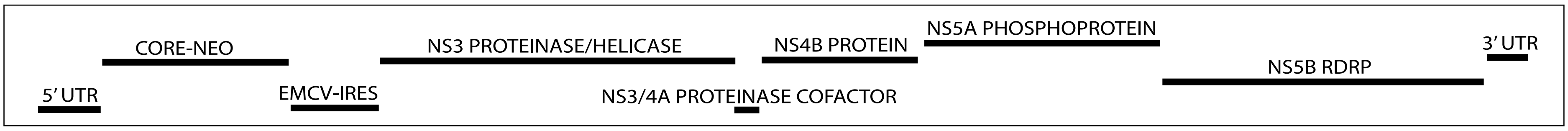

S8B.

HCVrep-derived sRNAs (Late passage) 5'-P-dep cloning (Sol-107). # of sequences: miRNAs (6461), (+) vsRNAs (164), (-) vsRNAs (138), Total (51,045)

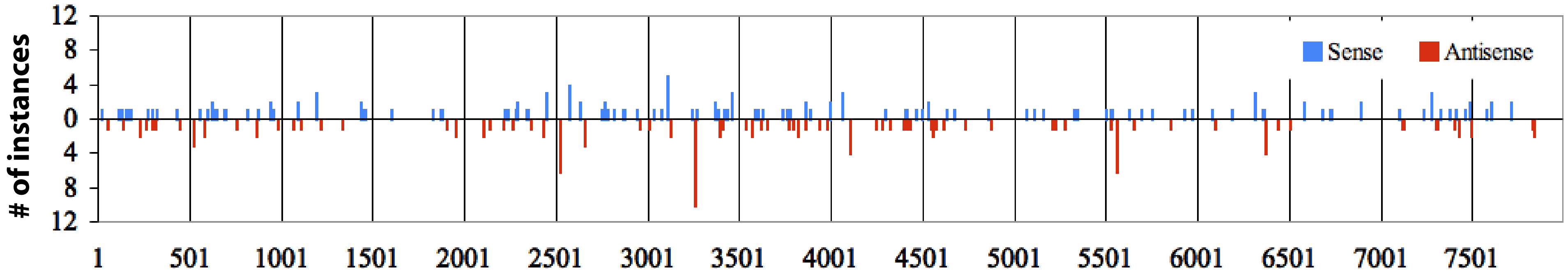

S8C.

HCVrep-derived sRNAs (Late passage) 5'-P-IND cloning (Sol-109). # of sequences: miRNAs (11,927), (+) vsRNAs (910), (-) vsRNAs (785), Total (178,464)

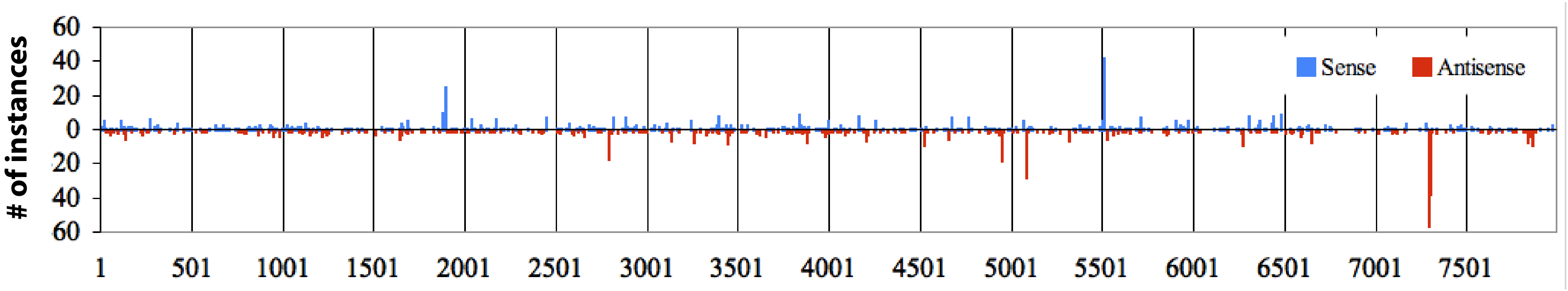

S8D.

HCVrep-derived sRNAs (Early passage) 5'-P-dep cloning (Sol-176). # of sequences: miRNAs (55,768), (+) vsRNAs (6959), (-) vsRNAs (5853), Total (1,946,923)

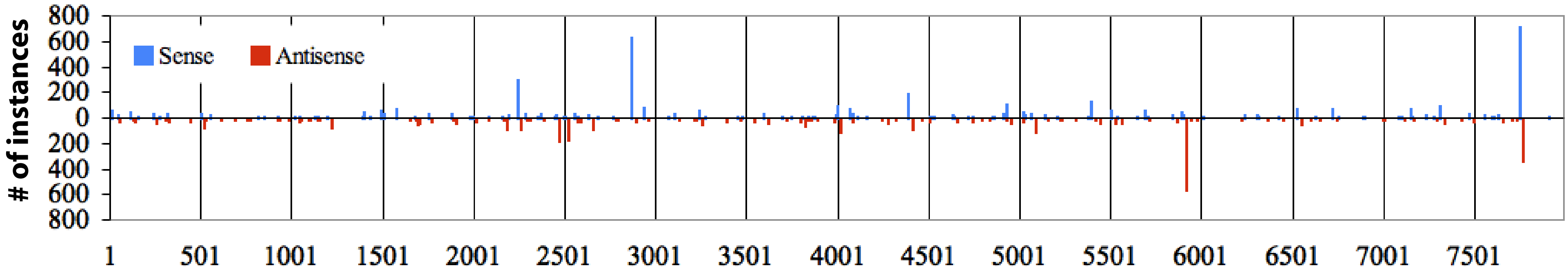

S8E.

HCVrep-derived sRNAs (Early passage) 5'-P-IND cloning (Sol-179). # of sequences: miRNAs (18,842), (+) vsRNAs (7964), (-) vsRNAs (6837), Total (2,211,220)

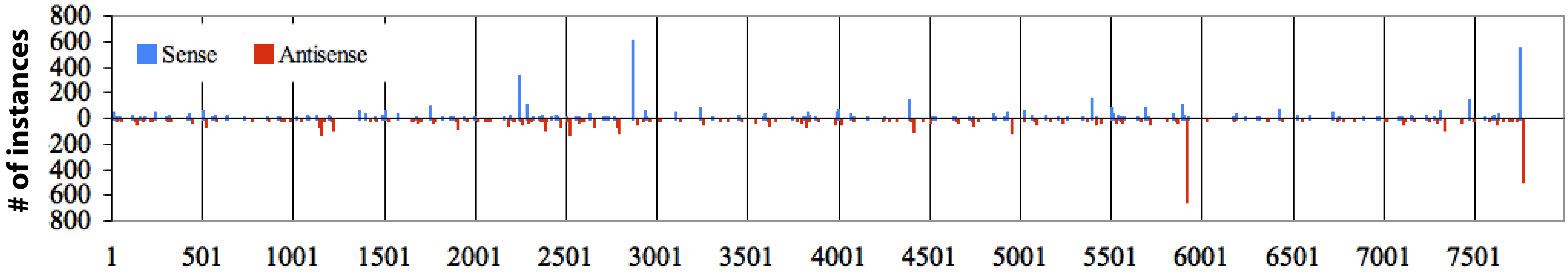

Supplement: Figure S8 — Abundance and distribution of HCVrep-derived 5′-P vsRNAs varies between early and late passage cells, but 5′-xP vsRNAs are enriched (relative to 5′-P) in both early and late passages. (S8A) Sequence count: all RNAs, miRNAs, vsRNAs (Y-axis: log scale). vsRNAs from a late passage (later than passage 15) of cells: (S8B) captured using the 5′-P-dependent protocol (Sol-107); (S8C) captured using the 5′-P-INDependent protocol (Sol-109). vsRNAs from an early passage (passage 3) of cells: (S8D) captured using the 5′-P-dependent protocol (Sol-176); (S8E) captured using the 5′-P-INDependent protocol (Sol-179). (0.34 MB PDF) [file ppat.1000764.s009.pdf]
