# Supplementary material for: Six RNA Viruses and Forty-One Hosts: Viral Small RNAs and Modulation of Small RNA Repertoires in Vertebrate and Invertebrate Systems
Source: PLoS Pathog. 2010 Feb 12;6(2):e1000764. doi: 10.1371/journal.ppat.1000764 (PMC2820531; doi:10.1371/journal.ppat.1000764)

S9A. Sequence count

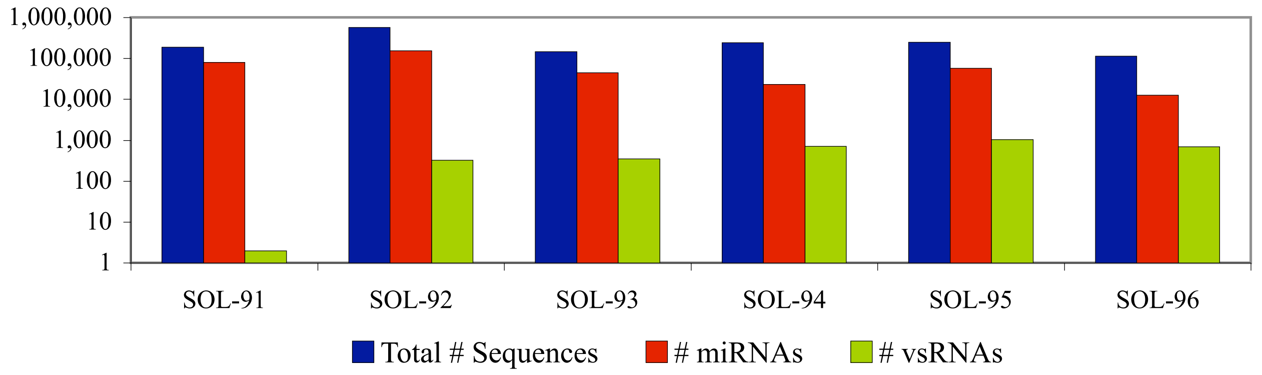

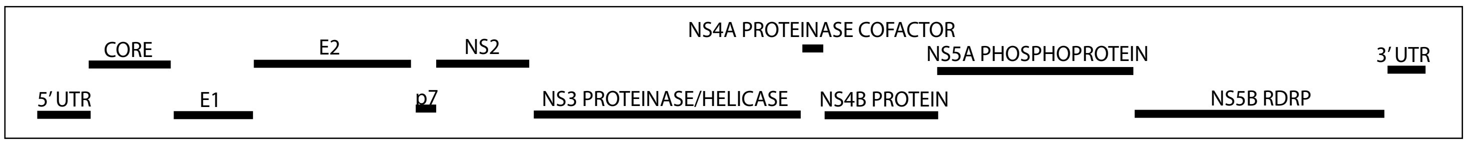

S9B. **Sol-91: Hepatitis C Virus vsRNAs (1dpi, Huh7.5). 5'-P-dep cloning. # of sequences: miRNAs (80,890), (+) vsRNAs (2), (-) vsRNAs (0), Total (187,137)**

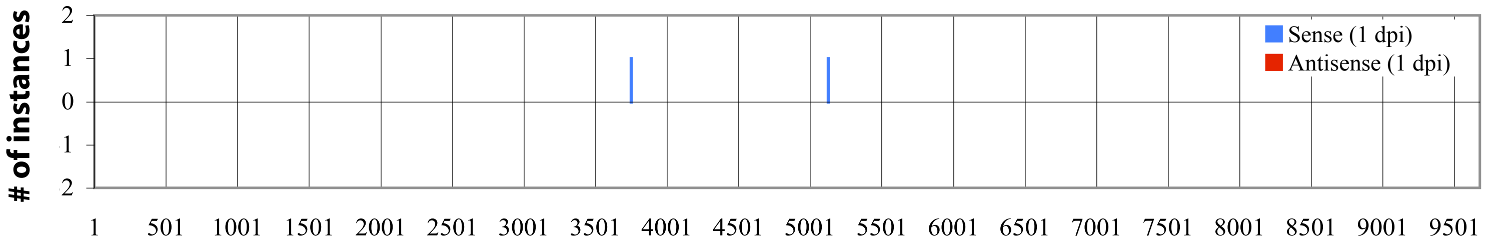

S9C. **Sol-92: Hepatitis C Virus vsRNAs (3dpi, Huh7.5). 5'-P-dep cloning. # of sequences: miRNAs (152,425), (+) vsRNAs (183), (-) vsRNAs (139), Total (568,886)**

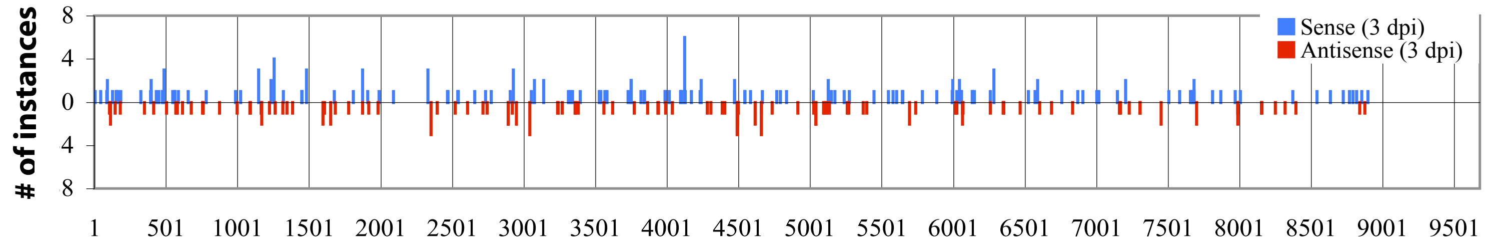

S9D. **Sol-93: Hepatitis C Virus vsRNAs (6dpi, Huh7.5). 5'-P-dep cloning. # of sequences: miRNAs (45,269), (+) vsRNAs (182), (-) vsRNAs (167), Total (146,972)**

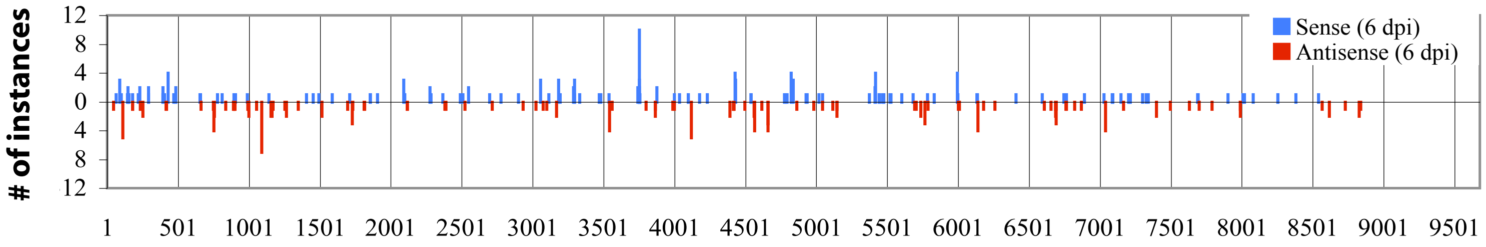

S9E. **Sol-94: Hepatitis C Virus vsRNAs (9dpi, Huh7.5). 5'-P-dep cloning. # of sequences: miRNAs (22,984), (+) vsRNAs (391), (-) vsRNAs (317), Total (244,248)**

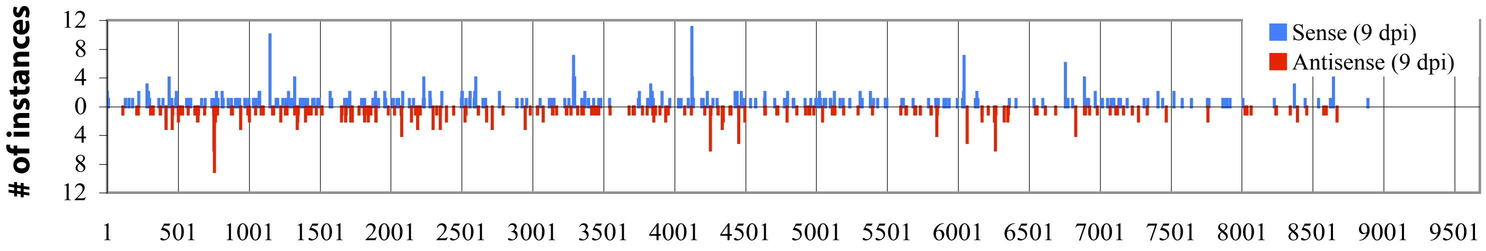

S9F. **Sol-95: Hepatitis C Virus vsRNAs (11dpi, Huh7.5). 5'-P-dep cloning. # of sequences: miRNAs (57,039), (+) vsRNAs (536), (-) vsRNAs (500), Total (250,510)**

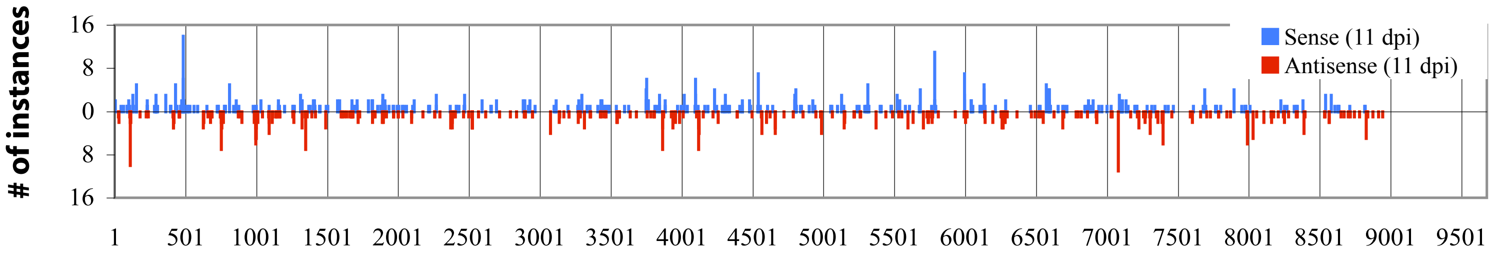

S9G. **Sol-96: Hepatitis C Virus vsRNAs (15dpi, Huh7.5). 5'-P-dep cloning. # of sequences: miRNAs (12,550), (+) vsRNAs (394), (-) vsRNAs (302), Total (114,109)**

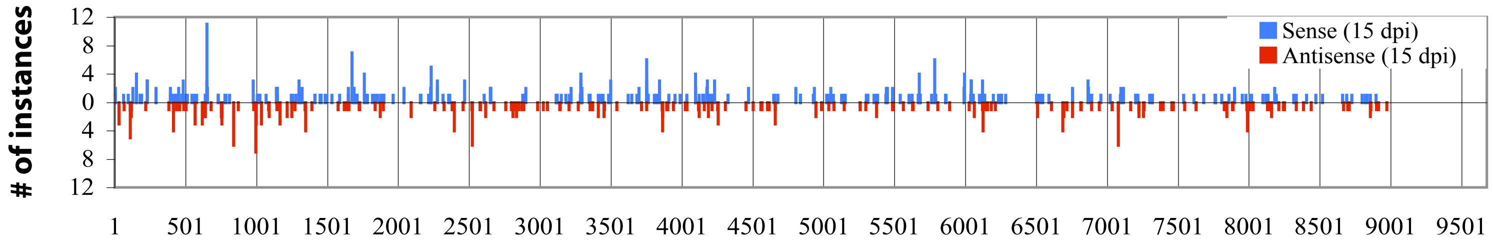

Supplement: Figure S9 — Increase in the abundance of HCVvir-derived vsRNAs (relative to miRNAs) at later time-points in infection (Solexa-sequenced libraries). Positive strand vsRNAs are shown as blue bars, and negative strand vsRNAs as red bars. (S9A) Sequence count: all RNAs, miRNAs, vsRNAs (Y-axis: log scale). vsRNAs with 5′ monophosphates from Huh7.5 cells infected with HCV virions: (S9B) 1 d.p.i (Sample: Sol-91); (S9C) 3 d.p.i (Sample Sol-92); (S9D) 6 d.p.i (Sample Sol-93); (S9E) 9 d.p.i (Sample Sol-94); (S9F) 11 d.p.i (Sample Sol-95); (S9G) 15 d.p.i (Sample Sol-96). (0.37 MB PDF) [file ppat.1000764.s010.pdf]
