# Supplementary material for: Six RNA Viruses and Forty-One Hosts: Viral Small RNAs and Modulation of Small RNA Repertoires in Vertebrate and Invertebrate Systems
Source: PLoS Pathog. 2010 Feb 12;6(2):e1000764. doi: 10.1371/journal.ppat.1000764 (PMC2820531; doi:10.1371/journal.ppat.1000764)

S10A.

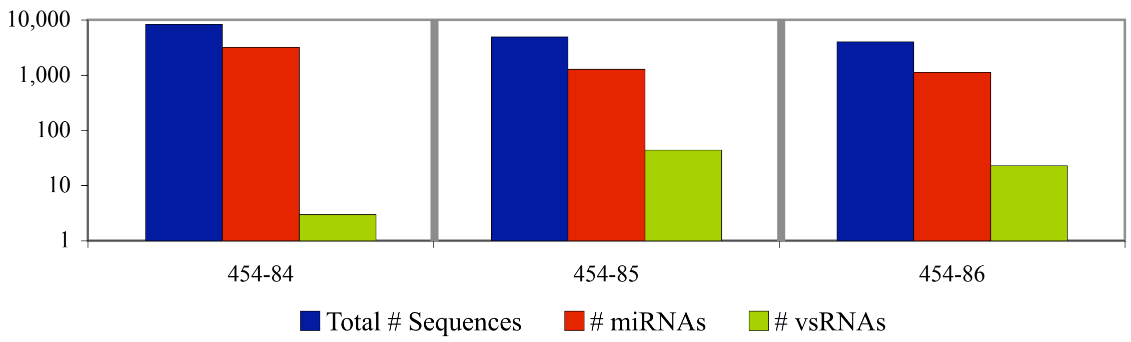

HEPATITIS C VIRUS (VIRION)

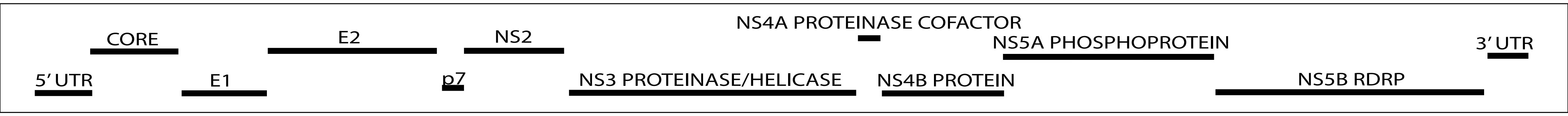

S10B.

454-84: Hepatitis C Virus vsRNAs (3dpi, Huh7.5). 5′-P-dep cloning. # of sequences: miRNAs (3183), (+) vsRNAs (3), (-) vsRNAs (0), Total (8296)

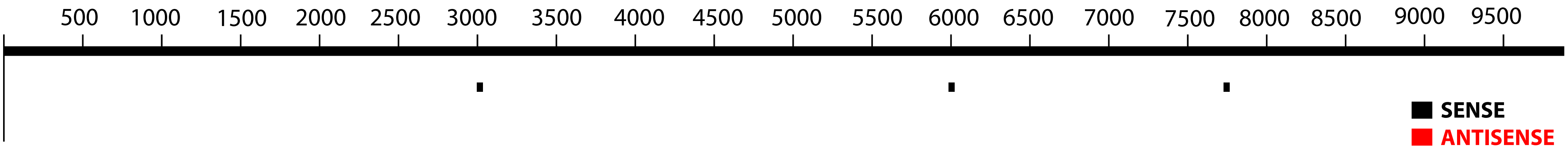

S10C.

454-85: Hepatitis C Virus vsRNAs (5dpi, Huh7.5). 5′-P-dep cloning. # of sequences: miRNAs (1289), (+) vsRNAs (27), (-) vsRNAs (17), Total (4911)

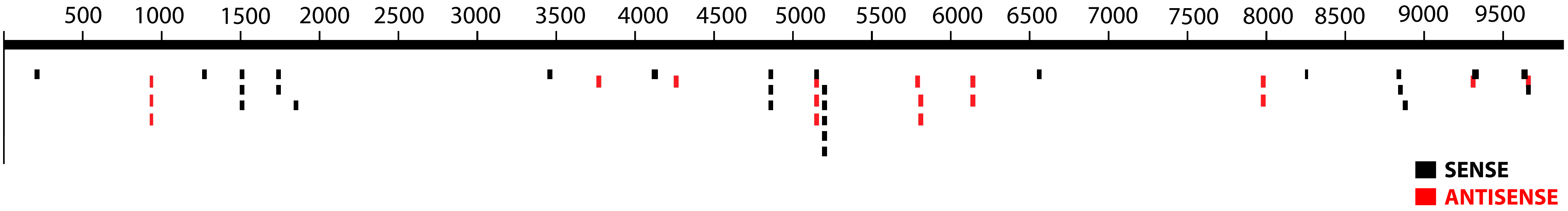

S10D.

454-86: Hepatitis C Virus vsRNAs (11dpi, Huh7.5). 5′-P-dep cloning. # of sequences: miRNAs (1127), (+) vsRNAs (14), (-) vsRNAs (9), Total (4062)

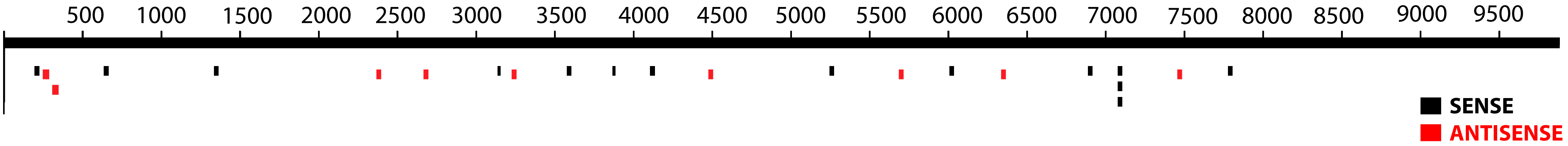

Supplement: Figure S10 — Temporal changes in the abundance of HCVvir-derived vsRNAs (GS-20/FLX-sequenced libraries). (S10A) Sequence count: all RNAs, miRNAs, vsRNAs (Y-axis: log scale). vsRNAs with 5′ monophosphates from Huh7.5 cells infected with HCV virions: (S10B) 3 d.p.i (Sample: 454-84); (S10C) 5 d.p.i (Sample: 454-85); (S10D) 11 d.p.i (Sample 454-86). (0.27 MB PDF) [file ppat.1000764.s011.pdf]
