# Supplementary material for: Six RNA Viruses and Forty-One Hosts: Viral Small RNAs and Modulation of Small RNA Repertoires in Vertebrate and Invertebrate Systems
Source: PLoS Pathog. 2010 Feb 12;6(2):e1000764. doi: 10.1371/journal.ppat.1000764 (PMC2820531; doi:10.1371/journal.ppat.1000764)

S11A.

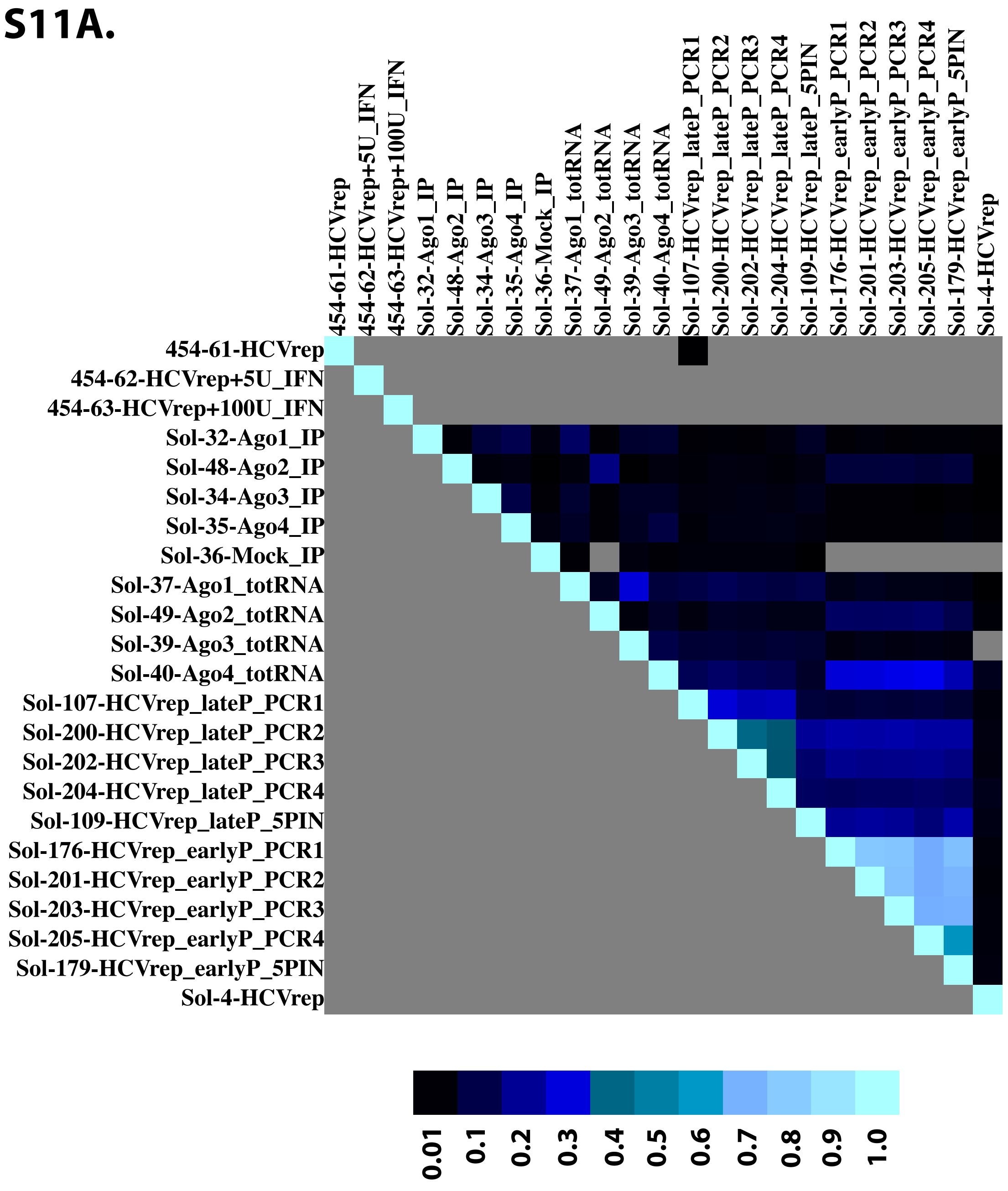

S11B.

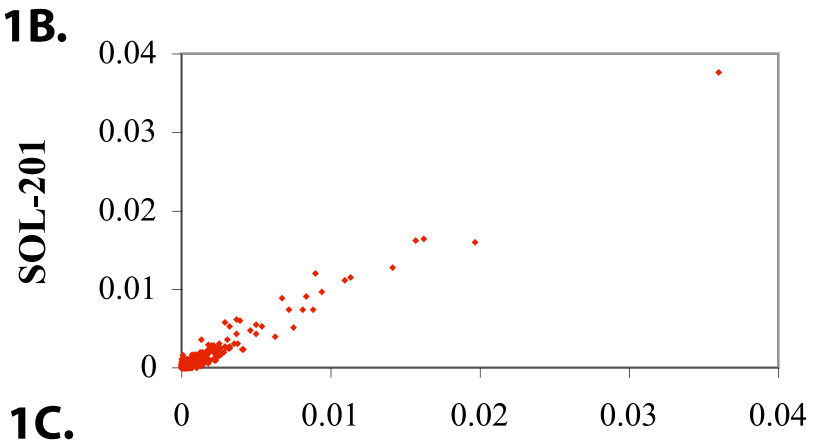

S11C.

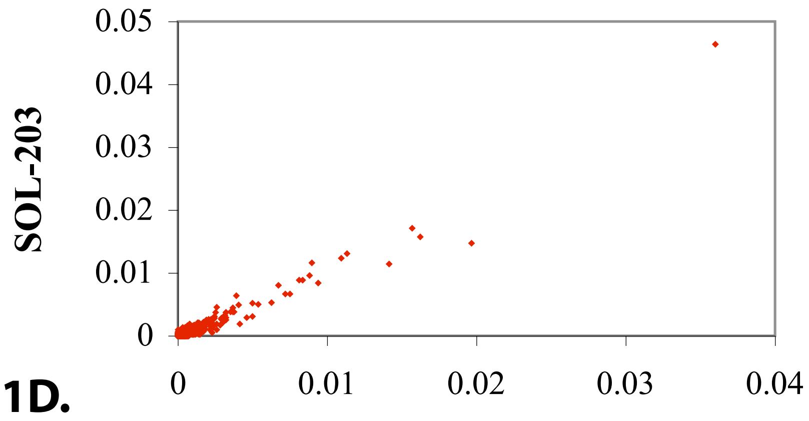

S11D.

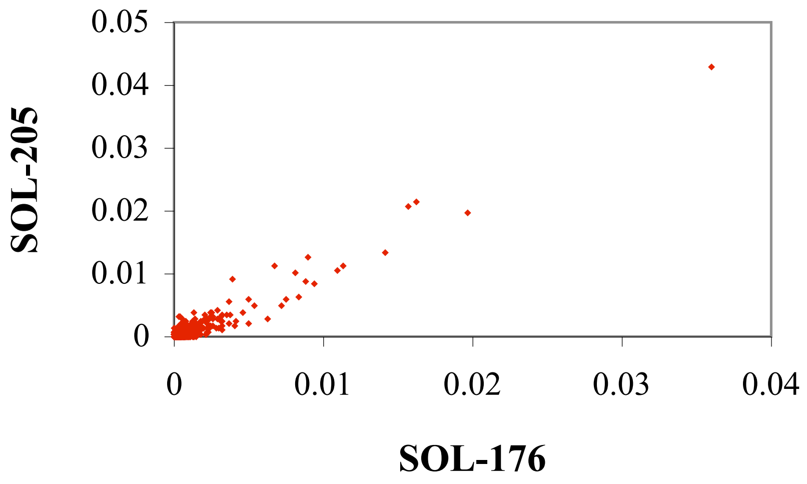

S11E.

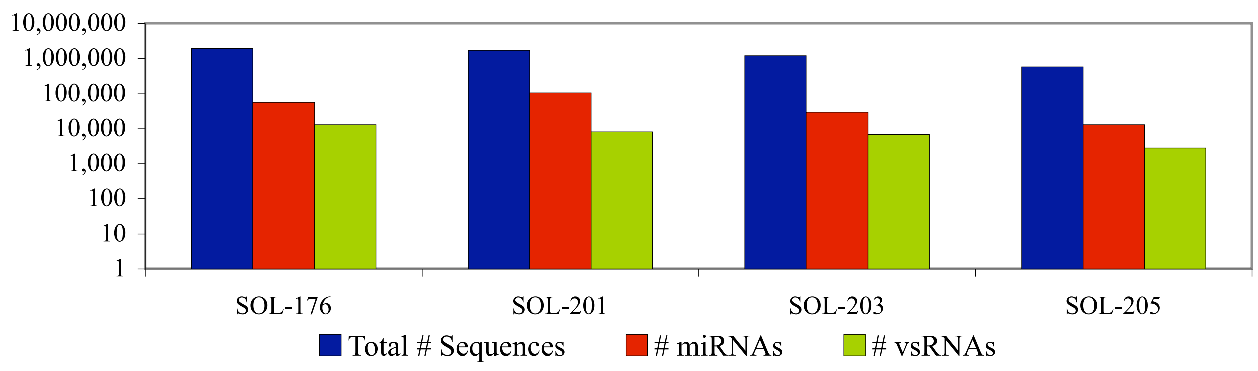

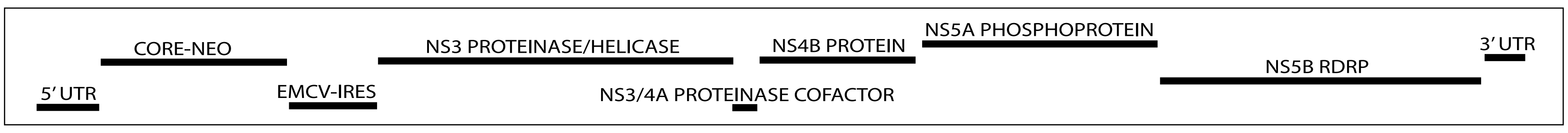

S11F.

Sol-176: Hepatitis C Virus Replicon-derived vsRNAs. 5'-P-dep cloning. # of sequences: miRNAs (55768), (+) vsRNAs (6959), (-) vsRNAs (5853), Total (1946923)

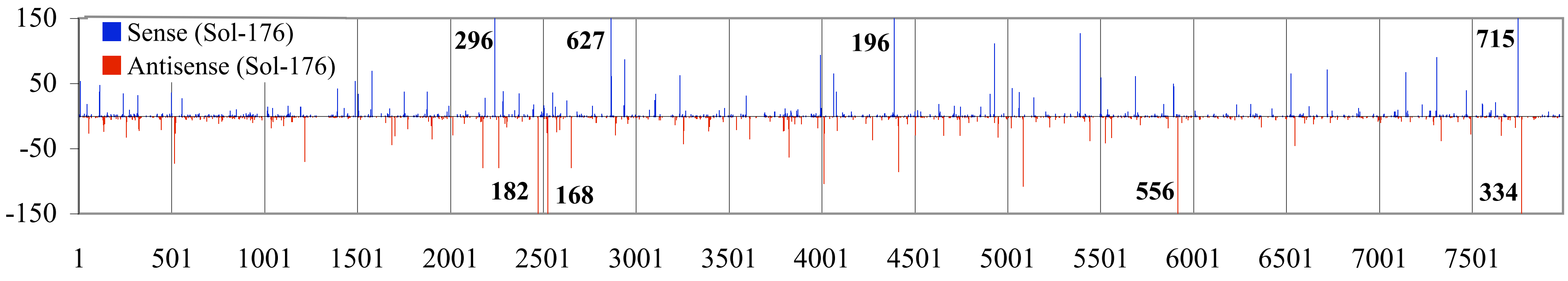

S11G.

Sol-201: Hepatitis C Virus Replicon-derived vsRNAs. 5'-P-dep cloning. # of sequences: miRNAs (103356), (+) vsRNAs (4438), (-) vsRNAs (3694), Total (1692531)

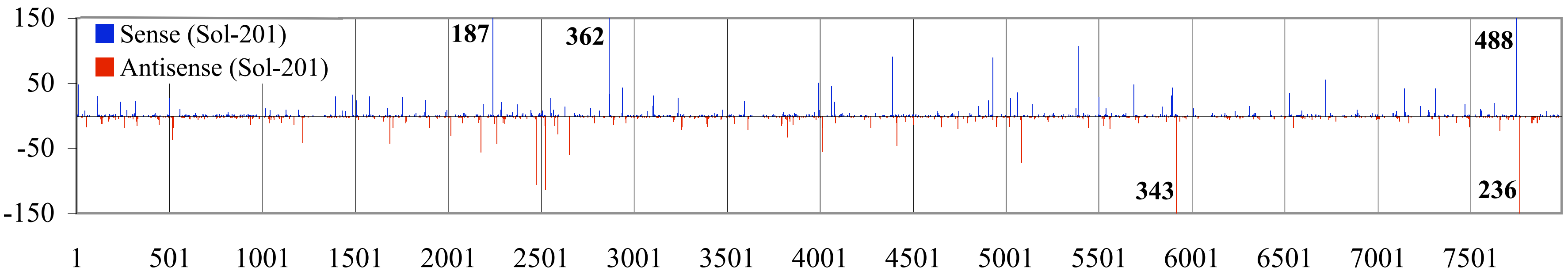

S11H.

Sol-203: Hepatitis C Virus Replicon-derived vsRNAs. 5'-P-dep cloning. # of sequences: miRNAs (29209), (+) vsRNAs (3690), (-) vsRNAs (3029), Total (1200175)

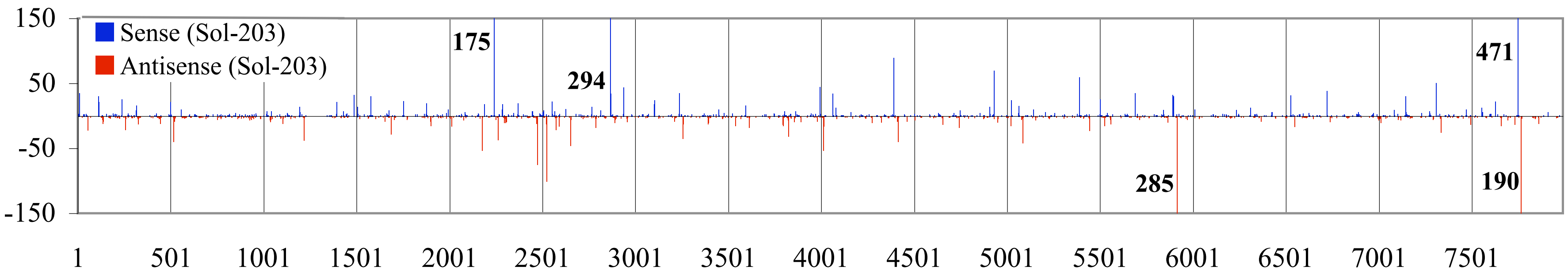

S11I.

Sol-205: Hepatitis C Virus Replicon-derived vsRNAs. 5'-P-dep cloning. # of sequences: miRNAs (13128), (+) vsRNAs (1569), (-) vsRNAs (1271), Total (581639)

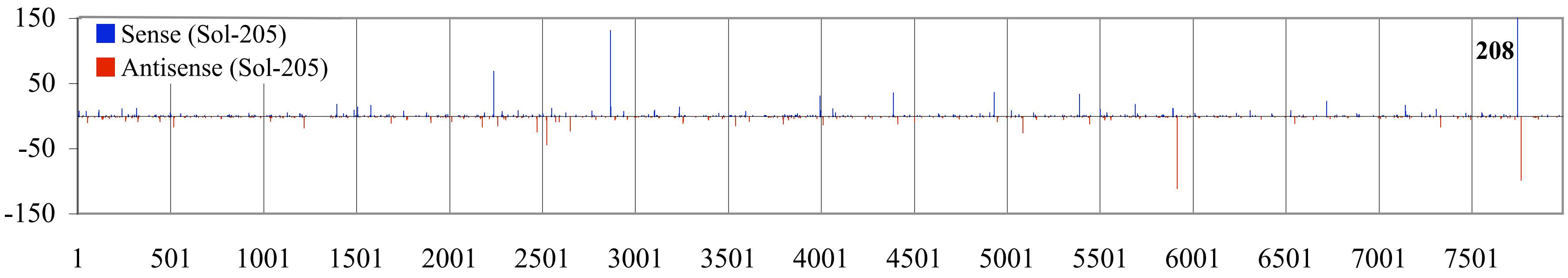

Supplement: Figure S11 — vsRNA coincidence plots show high degree of similarity between independent amplicon libraries from the same starting sample, and reproducible detection of hotspots. (S11A) Coincidences are defined by vsRNAs of the same orientation, with the same Start and End positions. Percent coincidence between ‘Sample-1’ and ‘Sample-2’ was calculated as follows: (# of coincidences between Sample-1 and Sample-2)/[(# of vsRNAs in Sample-1) + (# of vsRNAs in Sample-2)] * 100. These values are plotted as a heat map, with dark-to-light blue representing low-to-high values. (S11B-D) Pairwise comparison of the relative abundance of individual vsRNAs between independent amplicon libraries of early-passage HCV replicon cells. Each point represents a unique vsRNA, and the X- and Y-axes represent relative abundance (with respect to all vsRNAs) in the indicated amplicon libraries. (S11E) Sequence count: all RNAs, miRNAs, vsRNAs (Y-axis: log scale). vsRNAs with 5′ monophosphates from HCVrep cells: (S11F) Sample: Sol-176; (S11G) Sample Sol-201; (S11H) Sample Sol-203; (S11I) Sample Sol-205. Positive strand vsRNAs are shown as blue bars, and negative strand vsRNAs as red bars. (0.70 MB PDF) [file ppat.1000764.s012.pdf]
