# Supplementary material for: Six RNA Viruses and Forty-One Hosts: Viral Small RNAs and Modulation of Small RNA Repertoires in Vertebrate and Invertebrate Systems
Source: PLoS Pathog. 2010 Feb 12;6(2):e1000764. doi: 10.1371/journal.ppat.1000764 (PMC2820531; doi:10.1371/journal.ppat.1000764)

S14A. **5′ aligned; (+) HCV Replicon vsRNAs; Unique instances**

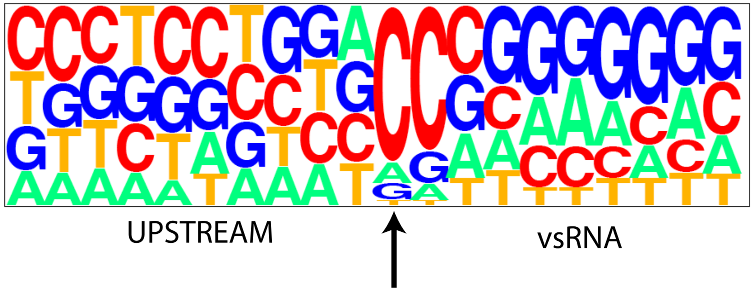

S14B. **5′ aligned; (-) HCV Replicon vsRNAs; Unique instances**

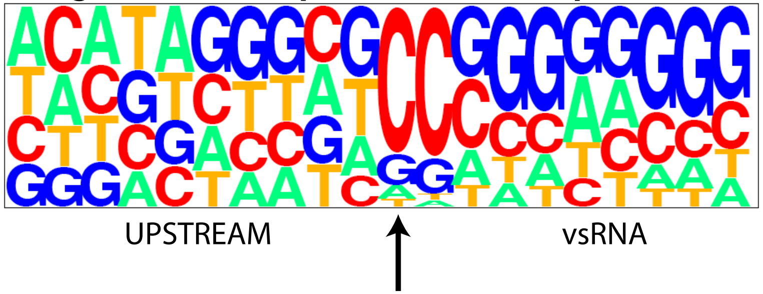

S14C. **5′ aligned; (+) HCV Replicon vsRNAs; All instances**

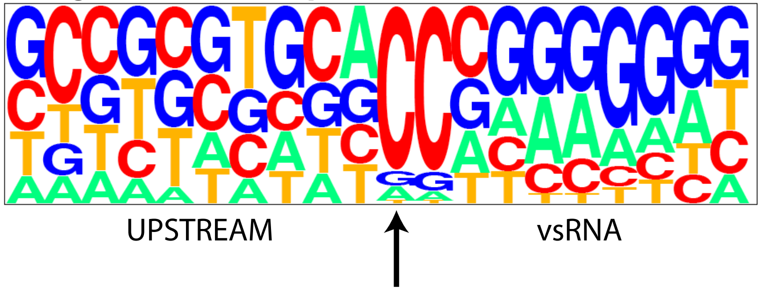

S14D. **5′ aligned; (-) HCV Replicon vsRNAs; All instances**

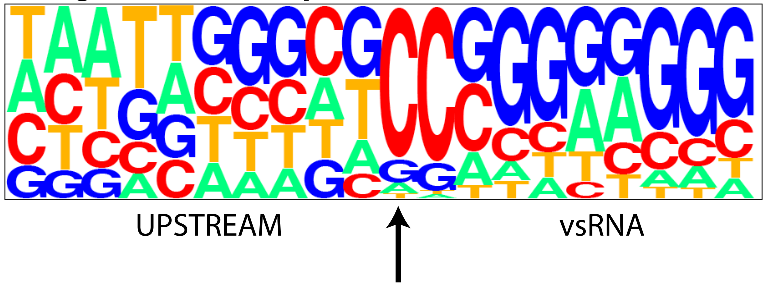

S14E. **3′ aligned; (+) HCV Replicon vsRNAs; Unique instances**

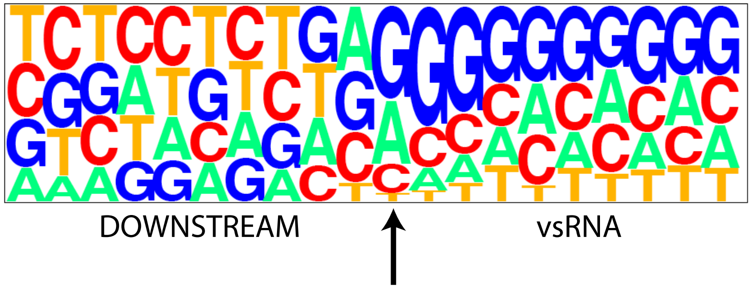

S14F. **3′ aligned; (-) HCV Replicon vsRNAs; Unique instances**

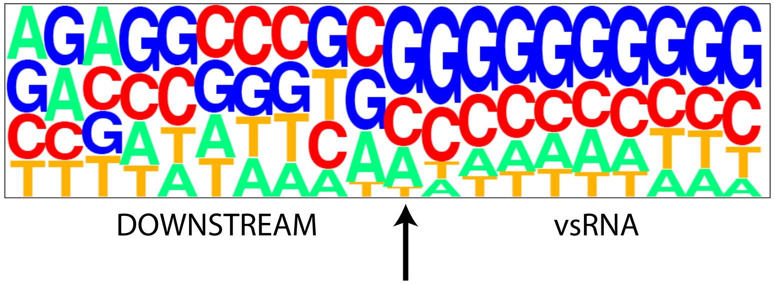

S14G. **3′ aligned; (+) HCV Replicon vsRNAs; All instances**

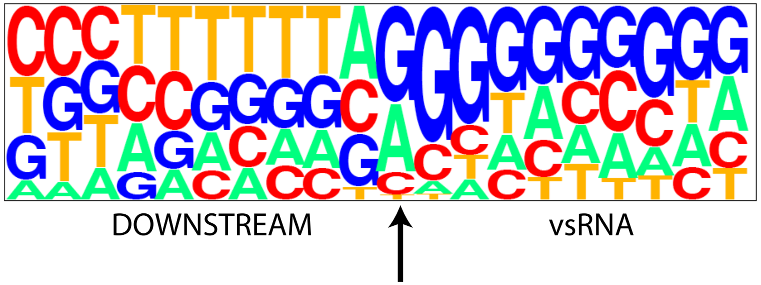

S14H. **3′ aligned; (-) HCV Replicon vsRNAs; All instances**

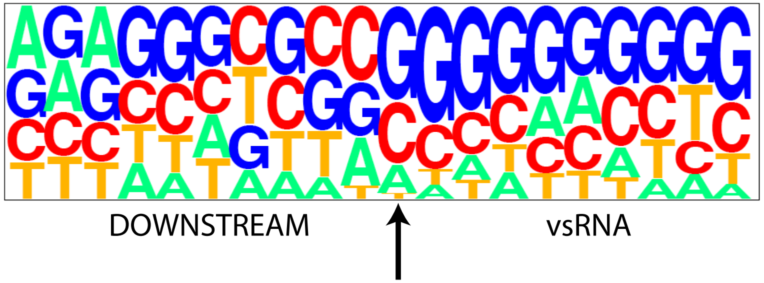

Supplement: Figure S14 — vsRNAs from cells with the HCV Replicon (Sol-4) exhibit a bias for Cytidines at their 5′ termini, and a bias for Guanines at their 3′ termini. vsRNAs were first aligned at their 5′ ends (or at their 3′ ends), and the nucleotide frequencies for 10 upstream (or 10 downstream) positions, plus for the first 10 (or last 10) vsRNA base positions were determined separately for positive strand and negative strand vsRNAs. This analysis was performed on all vsRNA instances, or post-collapse of the vsRNA dataset (‘unique instances’), such that multiple instances of vsRNAs were represented only once. Arrows indicate Start/End positions of vsRNAs. Nucleotide frequencies have been normalized to the overall nucleotide composition within each dataset. (S14A–S14D) Pictogram of 10 bases upstream+10 bases (from 5′ termini) of vsRNAs derived from (S14A) Positive strand: unique instances; (S14B) Negative strand: unique instances; (S14C) Positive strand: all instances; (S14D) Negative strand: all instances. (S14E–S14H) Pictogram of 10 bases downstream+10 bases (from 3′ termini) of vsRNAs derived from (S14E) Positive strand: unique instances; (S14F) Negative strand: unique instances; (S14G) Positive strand: all instances; (S14H) Negative strand: all instances. (0.67 MB PDF) [file ppat.1000764.s015.pdf]
