# Supplementary material for: Six RNA Viruses and Forty-One Hosts: Viral Small RNAs and Modulation of Small RNA Repertoires in Vertebrate and Invertebrate Systems
Source: PLoS Pathog. 2010 Feb 12;6(2):e1000764. doi: 10.1371/journal.ppat.1000764 (PMC2820531; doi:10.1371/journal.ppat.1000764)

S17A.

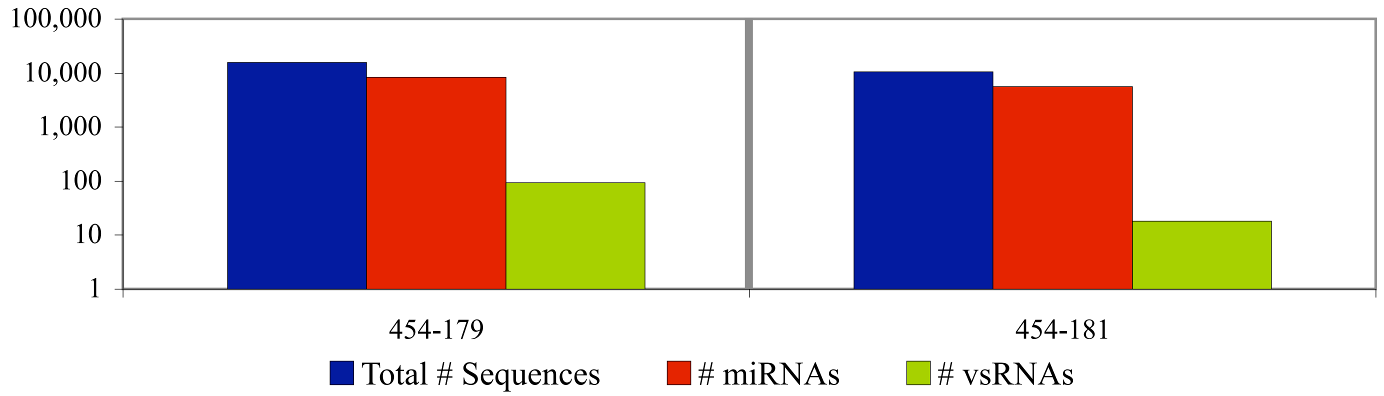

VESICULAR STOMATITIS VIRUS

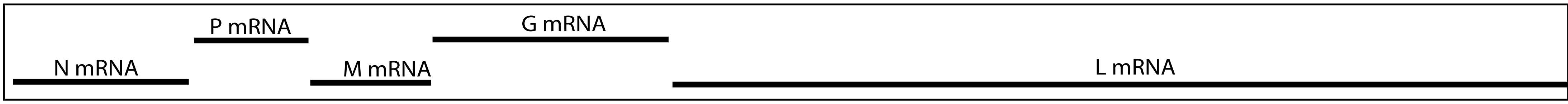

S17B.

454-179: Vesicular Stomatitis Virus vsRNAs in BHK cells. 5'-P-dep cloning (Pyroseq). # of sequences: miRNAs (9277), (+) vsRNAs (56), (-) vsRNAs (52), Total (15876)

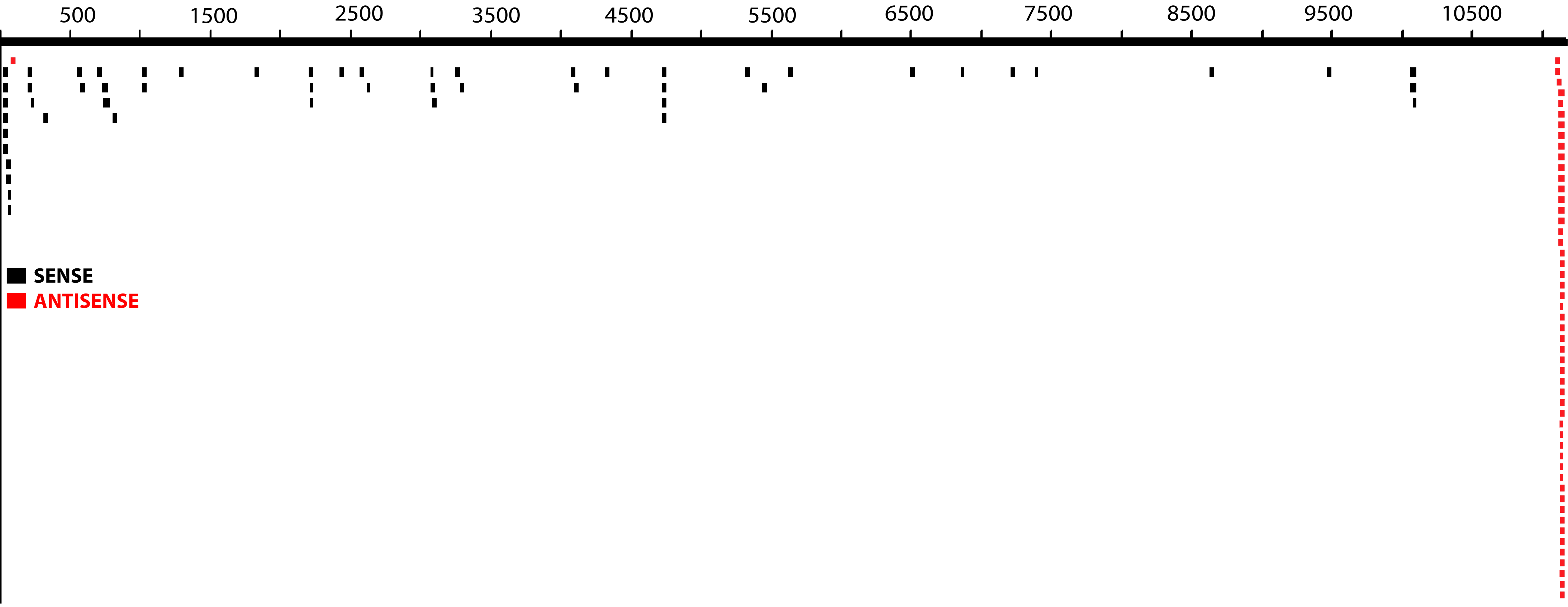

S17C.

454-181: Vesicular Stomatitis Virus vsRNAs in Hela cells. 5'-P-dep cloning (Pyroseq). # of sequences: miRNAs (6796), (+) vsRNAs (19), (-) vsRNAs (0), Total (10424)

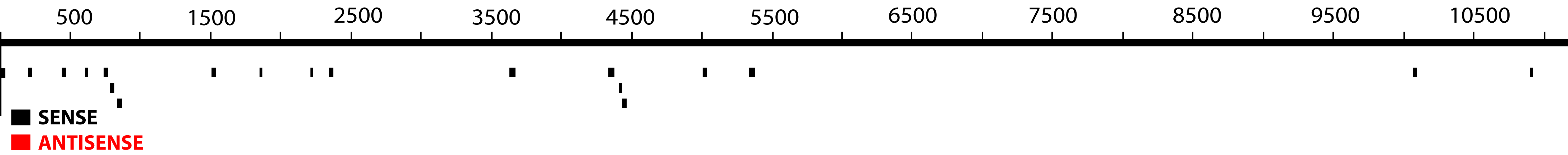

Supplement: Figure S17 — Abundance, distribution and orientation biases of VSV-derived vsRNAs are a function of cell-type. (S17A) Sequence count: all RNAs, miRNAs, vsRNAs (Y-axis: log scale). vsRNAs with 5′ monophosphates from Vesicular Stomatitis virus infections in: (S17B) BHK-21 cells, harvested 4 h.p.i (Sample: 454-179); (S17C) HeLa cells, harvested 4 h.p.i (Sample: 454-181). (0.28 MB PDF) [file ppat.1000764.s018.pdf]
