# Supplementary material for: Six RNA Viruses and Forty-One Hosts: Viral Small RNAs and Modulation of Small RNA Repertoires in Vertebrate and Invertebrate Systems
Source: PLoS Pathog. 2010 Feb 12;6(2):e1000764. doi: 10.1371/journal.ppat.1000764 (PMC2820531; doi:10.1371/journal.ppat.1000764)

S20A.

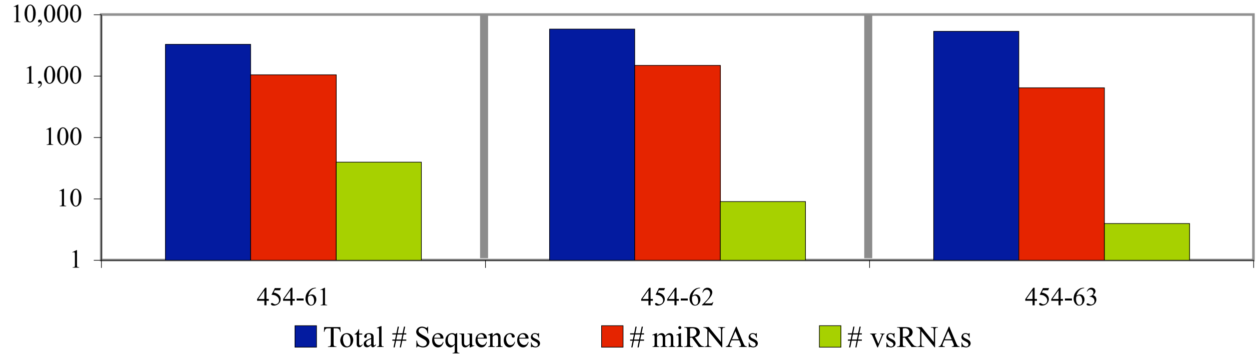

HEPATITIS C REPLICON

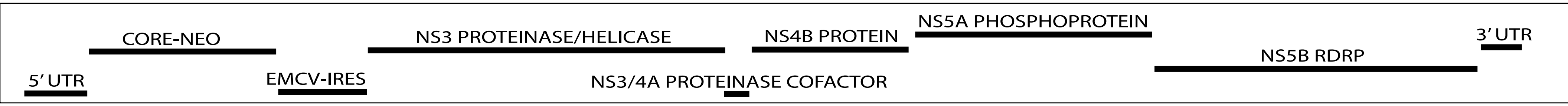

S20B.

454-61: Hepatitis C Replicon-derived vsRNAs. 5'-P-dep cloning. # of sequences: miRNAs (1373), (+) vsRNAs (26), (-) vsRNAs (18), Total (3987)

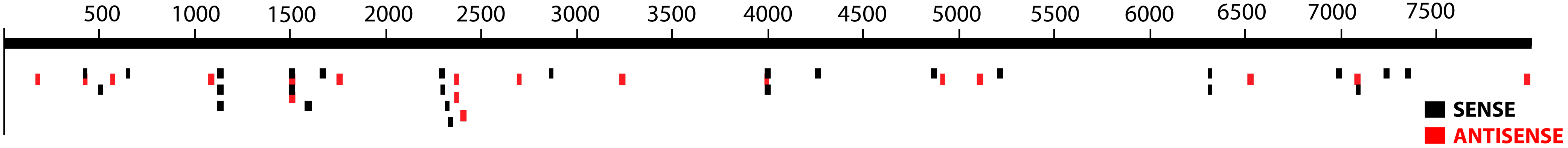

S20C.

454-62: Hepatitis C Replicon vsRNAs post-tr. with 5U/mL IFN. 5'-P-dep cloning. # of sequences: miRNAs (2039), (+) vsRNAs (6), (-) vsRNAs (4), Total (5840)

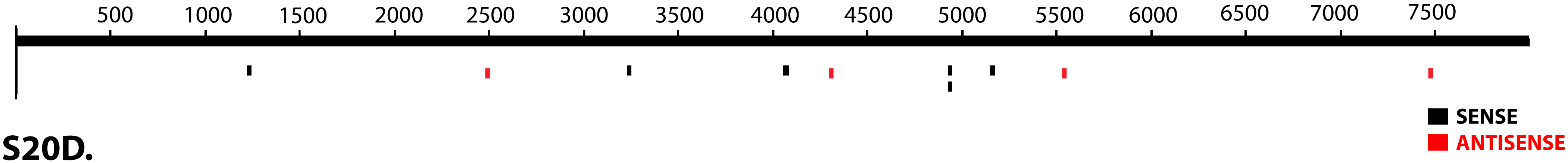

S20D.

454-63: Hepatitis C Replicon vsRNAs post-tr. with 100U/mL IFN. 5'-P-dep cloning. # of sequences: miRNAs (877), (+) vsRNAs (3), (-) vsRNAs (1), Total (5327)

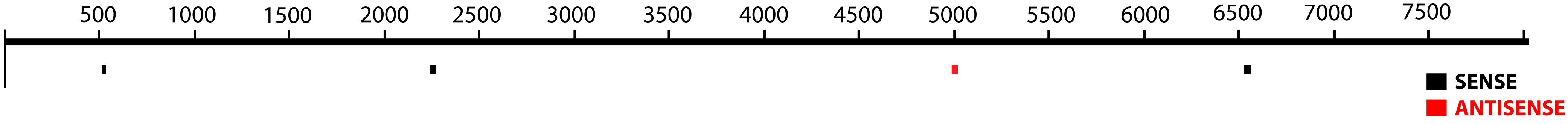

Supplement: Figure S20 — IFN responsiveness reduces the incidence of HCVrep-derived vsRNAs. (S20A) Sequence count: all RNAs, miRNAs, vsRNAs (Y-axis: log scale). vsRNA profiles from: (S20B) untreated HCVrep cells; (S20C) HCVrep cells treated with 5 U/mL IFN, and harvested 72 hours post-treatment; (S20D) HCVrep cells treated with 100 U/mL IFN, and harvested 72 hours post-treatment. (0.27 MB PDF) [file ppat.1000764.s021.pdf]
