# Supplementary material for: Six RNA Viruses and Forty-One Hosts: Viral Small RNAs and Modulation of Small RNA Repertoires in Vertebrate and Invertebrate Systems
Source: PLoS Pathog. 2010 Feb 12;6(2):e1000764. doi: 10.1371/journal.ppat.1000764 (PMC2820531; doi:10.1371/journal.ppat.1000764)
Supplement: Table S1 — Reference genomes of all viruses used. (0.04 MB PDF) [file ppat.1000764.s022.pdf]

Virus	Genome
**Poliovirus 1**	TTAAAACAGCTCTGGGGTTGTACCCACCCCAGAGGCCCACGTGGCGGCTAGTACTC<br>CGGTATTGCGGTACCCTTGTACGCCTGTTTTATACTCCCTTCCCGTAACTTAGACGCA<br>CAAAACCAAGTTCAATAGAAGGGGGTACAAACCAGTACCACCACGAACAAGCACTT<br>CTGTTTCCCCGGTGATGTCGTATAGACTGCTTGCGTGGTTGAAAGCGACGGATCCG<br>TTATCCGCTTATGTACTTCGAGAAGCCCAGTACCACCTCGGAATCTTCGATGCGTTG<br>CGCTCAGCACTCAACCCCAGAGTGTAGCTTAGGCTGATGAGTCTGGACATCCCTCA<br>CCGGTGACGGTGGTCCAGGCTGCGTTGGCGGCCTACCTATGGCTAACGCCATGGG<br>ACGCTAGTTGTGAACAAGGTGTGAAGAGCCTATTGAGCTACATAAGAATCCTCCGG<br>CCCCTGAATGCGGCTAATCCCAACCTCGGAGCAGGTGGTCACAAACCAGTGATTGG<br>CCTGTCGTAACGCGCAAGTCCGTGGCGGAACCGACTACTTTGGGTGTCCGTGTTTC<br>CTTTTATTTTATTGTGGCTGCTTATGGTGACAATCACAGATTGTTATCATAAAGCGAAT<br>TGGATTGGCCATCCGGTGAAAGTGAGACTCATTATCTATCTGTTTGCTGGATCCGCT<br>CCATTGAGTGTGTTTACTCTAAGTACAATTTCAACAGTTATTTCAATCAGACAATTGTA<br>TCATAATGGGTGCTCAGGTTTCATCACAGAAAGTGGGCGCACATGAAAACTCAAATA<br>GAGCGTATGGTGGTTCTACCATTAATTACACCACCATTAATTATTATAGAGATTCAGCT<br>AGTAACGCGGCTTCGAAACAGGACTTCTCTCAAGACCCTTCCAAGTTCACCGAGCC<br>CATCAAGGATGTCCTGATAAAAACAGCCCCAATGCTAAACTCGCCAAACATAGAGG<br>CTTGCGGGTATAGCGATAGAGTACTGCAATTAACACTGGGAAACTCCACTATAACCA<br>CACAGGAGGCGGCTAATTCAGTAGTCGCTTATGGGCGTTGGCCTGAATATCTGAGG<br>GACAGCGAAGCCAATCCAGTGGACCAGCCGACAGAACCAGACGTCGCTGCATGCAC
**Flock House Virus RNA1**	GTTTTCGAAACAAATAAAACAGAAAAGCGAACCTAAACAATGACTCTAAAAGTTATTC<br>TTGGAGAACACCAGATCACCCGAACTGAATTGTTAGTCGGGATTGCAACCGTATCTG<br>GGTGCGGTGCCGTAGTGTACTGCATATCCAAGTTCTGGGGCTATGGGGCAATTGCG<br>CCCTATCCTCAGAGTGGAGGGAACCGAGTTACACGCGCATTGCAACGGGCTGTCAT<br>TGACAAAACGAAGACCCCGATAGAGACACGTTTCTATCCGCTTGACAGCCTGCGTA<br>CCGTGACGCCTAAGCGTGTCGCAGACAACGGGCACGCCGTTTCAGGGGCCGTACG<br>TGATGCCGCACGTCGTTTGATCGACGAGTCCATCACGGCCGTTGGAGGATCCAAAT<br>TTGAGGTCAACCCCAACCCAAACTCAAGCACTGGACTGCGAAACCATTTCCACTTC<br>GCCGTCGGTGATTTGGCACAAGATTTCCGTAATGACACACCTGCGGATGATGCCTTC<br>ATCGTCGGTGTTGATGTTGATTATTATGTCACCGAGCCTGATGTGCTTTTAGAGCACA<br>TGCGTCCAGTAGTGTTACACACCTTTAACCCGAAGAAAGTGAGCGGTTTTGATGCTG<br>ACTCACCATTCACCATTAAGAACAACTTGGTTGAATATAAGGTTAGCGGTGGAGCAG<br>CATGGGTCCATCCAGTTTGGGATTGGTGCGAAGCTGGTGAGTTTATCGCTAGCAGA<br>GTCCGTACCAGCTGGAAGGAGTGGTTTTTACAACTACCACTGCGAATGATTGGTTTG<br>GAGAAGGTTGGCTATCATAAAATCCATCATTGTAGACCGTGGACTGATTGTCCAGAT<br>CGTGCACTTGTCTACACTATACCGCAATATGTCATTTGGCGATTTAATTGGATTGATAC<br>CGAACTACACGTGCGAAAACTGAAACGGATTGAATACCAGGACGAAACCAAACCTG<br>GTTGGAACAGATTGGAGTATGTGACCGACAAGAATGAACTGCTGGTTTCCATCGGT<br>CGAGAAGGGGAGCATGCTCAGATTACTATCGAGAAAGAAAAGTTGGATATGCTCTC<br>GGGATTATCCGCCACCCAATCTGTCAACGCTAGGCTTATCGGTATGGGACACAAGGA

**Hepatitis C subgenomic Replicon**	GCCAGCCCCCGATTGGGGGCGACACTCCACCATAGATCACTCCCCTGTGAGGAACT<br>ACTGTCTTCACGCAGAAAGCGTCTAGCCATGGCGTTAGTATGAGTGTCGTGCAGCC<br>TCCAGGACCCCCCCTCCCGGGAGAGCCATAGTGGTCTGCGGAACCGGTGAGTACA<br>CCGGAATTGCCAGGACGACCGGGTCCTTTCTTGGATCAACCCGCTCAATGCCTGGA<br>GATTTGGGCGTGCCCCCGCGAGACTGCTAGCCGAGTAGTGTTGGGTCGCGAAAGG<br>CCTTGTGGTACTGCCTGATAGGGTGCTTGCGAGTGCCCCGGGAGGTCTCGTAGACC<br>GTGCACCATGAGCACGAATCCTAAACCTCAAAGAAAAACCAAAGGGCGCGCCATGA<br>TTGAACAAGATGGATTGCACGCAGGTTCTCCGGCCGCTTGGGTGGAGAGGCTATTC<br>GGCTATGACTGGGCACAACAGACAATCGGCTGCTCTGATGCCGCCGTGTTCCGGCT<br>GTCAGCGCAGGGGCGCCCGGTTCTTTTTGTCAAGACCGACCTGTCCGGTGCCCTG<br>AATGAACTGCAGGACGAGGCAGCGCGGCTATCGTGGCTGGCCACGACGGGCGTTC<br>CTTGCGCAGCTGTGCTCGACGTTGTCACTGAAGCGGGAAGGGACTGGCTGCTATT<br>GGGCGAAGTGCCGGGGCAGGATCTCCTGTCATCTCACCTTGCTCCTGCCGAGAAA<br>GTATCCATCATGGCTGATGCAATGCGGCGGCTGCATACGCTTGATCCGGCTACCTGC<br>CCATTCGACCACCAAGCGAAACATCGCATCGAGCGAGCACGTACTCGGATGGAAG<br>CCGGTCTTGTCGATCAGGATGATCTGGACGAAGAGCATCAGGGGCTCGCGCCAGC<br>CGAACTGTTCGCCAGGCTCAAGGCGCGCATGCCCGACGGCGAGGATCTCGTCGTG<br>ACCCATGGCGATGCCTGCTTGCCGAATATCATGGTGGAAAATGGCCGCTTTTCTGGA<br>TTCATCGACTGTGGCCGGCTGGGTGTGGCGGACCGCTATCAGGACATAGCGTTGG<br>CTACCCGTGATATTGCTGAAGAGCTTGGCGGCGAATGGGCTGACCGCTTCCTCGTG
**Hepatitis C Virus**	ACCTGCCCCTAATAGGGGCGACACTCCGCCATGAATCACTCCCCTGTGAGGAACTA<br>CTGTCTTCACGCAGAAAGCGCCTAGCCATGGCGTTAGTATGAGTGTCGTACAGCCT<br>CCAGGCCCCCCCTCCCGGGAGAGCCATAGTGGTCTGCGGAACCGGTGAGTACAC<br>CGGAATTGCCGGGAAGACTGGGTCCTTTCTTGGATAAACCCACTCTATGCCCGGCC<br>ATTTGGGCGTGCCCCCGCAAGACTGCTAGCCGAGTAGCGTTGGGTTGCGAAAGGC<br>CTTGTGGTACTGCCTGATAGGGCGCTTGCGAGTGCCCCGGGAGGTCTCGTAGACC<br>GTGCACCATGAGCACAAATCCTAAACCTCAAAGAAAAACCAAAAGAAACACCAACC<br>GTCGCCCACAAGACGTTAAGTTTCCGGGCGGCGGCCAGATCGTTGGCGGAGTATA<br>CTTGTTGCCGCGCAGGGGCCCCAGGTTGGGTGTGCGCGCGACAAGGAAGACTTC<br>GGAGCGGTCCCAGCCACGTGGAAGGCGCCAGCCCATCCCTAAAGATCGGCGCTCC<br>ACTGGCAAATCCTGGGGAAAACCAGGATACCCCTGGCCCCTATACGGGAATGAGG<br>GACTCGGCTGGGCAGGATGGCTCCTGTCCCCCCGAGGTTCCCGTCCCTCTTGGGG<br>CCCCAATGACCCCCGGCATAGGTCGCGCAACGTGGGTAAGGTCATCGATACCCTAA<br>CGTGCGGCTTTGCCGACCTCATGGGGTACATCCCTGTCGTGGGCGCCCCGCTCGG<br>CGGCGTCGCCAGAGCTCTCGCGCATGGCGTGAGAGTCCTGGAGGACGGGGTTAAT<br>TTTGCAACAGGGAACTTACCCGGTTGCTCCTTTTCTATCTTCTTGCTGGCCCTGCTGT<br>CCTGCATCACCACCCCGGTCTCCGCTGCCGAAGTGAAGAACATCAGTACCGGCTAC<br>ATGGTGACTAACGACTGCACCAATGACAGCATTACCTGGCAGCTCCAGGCTGCTGT<br>CCTCCACGTCCCCGGGTGCGTCCCGTGCGAGAAAGTGGGGAATGCATCTCAGTGC<br>TGGATACCGGTCTCACCGAATGTGGCCGTGCAGCGGCCCGGCGCCCTCACGCAGG

**Dengue Virus 2**	TGGCCCGACAAAGACAGATTCTTTGAGGGAGCTGAGCTCAACGTAGTTCTGACTGT<br>TTTTTGATTAGAGAGCAGATCTCTGATGAATGACCAACGGAAAAAGGCGAGAAACA<br>CGCCTTTCAATATGCTGAAACGCGAGAGAAACCGCGTGTCAACTGTACAACAGTTG<br>ACAAAGAGATTCTCACTTGGAATGCTGCAGGGACGAGGACCACTAAAATTGTTCAT<br>GGCCCTGGTGGCATTCCTTCGTTTCCTAACAATCCCACCAACAGCAGGGATATTAAA<br>AAGATGGGGAACAATTAAAAAATCAAAGGCTATTAATGTTCTGAGAGGCTTCAGGAA<br>AGAGATTGGAAGGATGCTGAATATCTTAAACAGGAGACGTAGAACTGCAGGCATGA<br>TCATCATGCTGATTCCAACAGTGATGGCGTTTCATCTGACCACACGCAACGGAGAAC<br>CACACATGATCGTCAGTAGACAAGAAAAAGGGAAAAGCCTTCTGTTTAAGACAAAG<br>GACGGCACGAACATGTGTACCCTCATGGCCATGGACCTTGGTGAGTTGTGTGAAGA<br>CACAATCACGTATAAATGTCCCTTTCTCAAGCAGAACGAACCAGAAGACATAGATTG<br>TTGGTGCAACTCCACGTCCACATGGGTAACTTATGGGACATGTACCACCACAGGAG<br>AGCACAGAAGAGAAAAAAGATCAGTGGCGCTTGTTCCACACGTGGGAATGGGATT<br>GGAGACACGAACTGAAACATGGATGTCATCAGAAGGGGCCTGGAAACATGCCCAG<br>AGAATTGAAACTTGGATTCTGAGACATCCAGGCTTTACCATAATGGCCGCAATCCTG<br>GCATACACCATAGGAACGACGCATTTCCAAAGAGTCCTGATATTCATCCTACTGACA<br>GCCATCGCTCCTTCAATGACAATGCGCTGCATAGGAATATCAAATAGGGACTTTGTG<br>GAAGGAGTGTCAGGAGGGAGTTGGGTTGACATAGTTTTAGAACATGGAAGTTGTGT<br>GACGACGATGGCAAAAAATAAACCAACACTGGACTTTGAACTGATAAAAACAGAAG<br>CCAAACAACCCGCCACCTTAAGGAAGTACTGTATAGAGGCTAAACTGACCAACACGA
**West Nile Virus**	AGTAGTTCGCCTGTGTGAGCTGACAAACTTAGTAGTGTTTGTGAGGATTAACAACAA<br>TTAACACAGTGCGAGCTGTTTCTTAGCACGAAGATCTCGATGTCTAAGAAACCAGGA<br>GGGCCCGGCAAGAGCCGGGCTGTCAATATGCTAAAACGCGGAATGCCCCGCGTGT<br>TGTCCTTGATTGGACTGAAGAGGGCTATGTTGAGCCTGATCGACGGCAAGGGGCCA<br>ATACGATTTGTGTTGGCTCTCTTGGCGTTCTTCAGGTTCACAGCAATTGCTCCGACC<br>CGAGCAGTGCTGGATCGATGGAGAGGTGTGAACAAACAAACAGCGATGAAACACC<br>TTCTGAGTTTTAAGAAGGAACTAGGGACCTTGACCAGTGCTATCAATCGGCGGAGC<br>TCAAAACAAAAGAAAAGAGGAGGAAAGACCGGAATTGCAGTCATGATTGGCCTGAT<br>CGCCAGCGTAGGAGCAGTTACCCTCTCTAACTTCCAAGGGAAGGTGATGATGACGG<br>TAAATGCTACTGACGTCACAGATGTCATCACGATTCCAACAGCTGCTGGAAAGAACC<br>TATGCATTGTCAGAGCAATGGATGTGGGATACATGTGCGATGATACTATCACTTATGA<br>ATGCCCAGTGCTGTCGGCTGGTAATGATCCAGAAGACATCGACTGTTGGTGCACAA<br>AGTCAGCAGTCTACGTCAGGTATGGAAGATGCACCAAGACACGCCACTCAAGACGC<br>AGTCGGAGGTCACTGACAGTGCAGACACACGGAGAAAGCACTCTAGCGAACAAGA<br>AGGGGGCTTGGATGGACAGCACCAAGGCCACAAGGTATTTGGTAAAAACAGAATCA<br>TGGATCTTGAGGAACCCTGGATATGCCCTGGTGGCAGCCGTCATTGGTTGGATGCT<br>TGGGAGCAACACCATGCAGAGAGTTGTGTTTGTCGTGCTATTGCTTTTGGTGGCCC<br>CAGCTTACAGCTTCAACTGCCTTGGAATGAGCAACAGAGACTTCTTGGAAGGAGTG<br>TCTGGAGCAACATGGGTGGATTTGGTTCTCGAAGGCGACAGCTGCGTGACTATCAT<br>GTCTAAGGACAAGCCTACCATCGATGTGAAGATGATGAATATGGAGGCGGCCAACCT

**Vesicular Stomatitis Virus; Indiana Strain**	ACGAAGACAAACAAACCATTATTATCATTAAAAGGCTCAGGAGAAACTTTAACAGTA<br>ATCAAAATGTCTGTTACAGTCAAGAGAATCATTGACAACACAGTCATAGTTCCAAAA<br>CTTCCTGCAAATGAGGATCCAGTGGAATACCCGGCAGATTACTTCAGAAAATCAAAG<br>GAGATTCCTCTTTACATCAATACTACAAAAAGTTTGTCAGATCTAAGAGGATATGTCT<br>ACCAAGGCCTCAAATCCGGAAATGTATCAATCATACATGTCAACAGCTACTTGTATG<br>GAGCATTAAAGGACATCCGGGGTAAGTTGGATAAAGATTGGTCAAGTTTCGGAATAA<br>ACATCGGGAAAGCAGGGGATACAATCGGAATATTTGACCTTGTATCCTTGAAAGCCC<br>TGGACGGCGTACTTCCAGATGGAGTATCGGATGCTTCCAGAACCAGCGCAGATGAC<br>AAATGGTTGCCTTTGTATCTACTTGGCTTATACAGAGTGGGCAGAACACAAATGCCT<br>GAATACAGAAAAAAGCTCATGGATGGGCTGACAAATCAATGCAAAATGATCAATGAA<br>CAGTTTGAACCTCTTGTGCCAGAAGGTCGTGACATTTTTGATGTGTGGGGAAATGAC<br>AGTAATTACACAAAAATTGTCGCTGCAGTGGACATGTTCTTCCACATGTTCAAAAAAC<br>ATGAATGTGCCTCGTTCAGATACGGAACTATTGTTTCCAGATTCAAAGATTGTGCTGC<br>ATTGGCAACATTTGGACACCTCTGCAAAATAACCGGAATGTCTACAGAAGATGTAAC<br>GACCTGGATCTTGAACCGAGAAGTTGCAGATGAAATGGTCCAAATGATGCTTCCAG<br>GCCAAGAAATTGACAAGGCCGATTCATACATGCCTTATTTGATCGACTTTGGATTGTC<br>TTCTAAGTCTCCATATTCTTCCGTCAAAAACCCTGCCTTCCACTTCTGGGGGCAATTG<br>ACAGCTCTTCTGCTCAGATCCACCAGAGCAAGGAATGCCCGACAGCCTGATGACAT<br>TGAGTATACATCTCTTACTACAGCAGGTTTGTTGTACGCTTATGCAGTAGGATCCTCTG
