# Supplementary material for: Six RNA Viruses and Forty-One Hosts: Viral Small RNAs and Modulation of Small RNA Repertoires in Vertebrate and Invertebrate Systems
Source: PLoS Pathog. 2010 Feb 12;6(2):e1000764. doi: 10.1371/journal.ppat.1000764 (PMC2820531; doi:10.1371/journal.ppat.1000764)
Supplement: Table S2 — Most abundant vsRNAs. The five most abundant vsRNAs captured in infections with FHV, VSV, Polio, HCVrep and HCVvir are listed. Start position, length, orientation and sequence of these vsRNAs are indicated, as are the number of datasets in which they were identified, and their respective total and normalized counts. vsRNAs were first defined by start position, length and orientation. All defined vsRNAs were then ranked based on (a) number of samples in which they were identified, and (b) their total count across all samples, normalized to the total vsRNA count (or to the total miRNA count) for those samples. For West Nile and Dengue Viruses, because of poor coverage, only four and two vsRNAs (respectively) are shown. (0.07 MB PDF) [file ppat.1000764.s023.pdf]

Virus (# of datasets)	*Start pos.*	*Length*	*Orientation*	*Sequence (5' to 3')*	*# of datasets with vsRNA (incident datasets)*	*Incidence of specified vsRNA*	*vsRNA count norm. to all vsRNAs in incident datasets*	*vsRNA count norm. to all miRNAs in incident datasets*
Flock House (4)	372	22	Antisense	GATGGACTCGTCGATCAAACGA	3	7	0.003975014	2.37E-05
	2767	23	Antisense	TTCCTGGATTAGCGCGAGTTTGC	2	31	0.020	0.00011
	2793	23	Sense	TGGAAGCAGCCATGGGAATGAGC	2	23	0.013	0.00010
	2836	23	Antisense	GTTGTTCGGTGCGTCTTGGTAGC	2	14	0.0081	6.36E-05
	820	22	Antisense	GATGGATTTTATGATAGCCAAC	2	12	0.0081	5.63E-05
Vesicular Stomatitis (4)	11161	25	Antisense	ACGAAGACCACAAAACCAGATAAAA	2	3	0.02	0.00025
	11152	17	Antisense	ACAAAACCAGATAAAAA	2	3	0.024	0.00022
	11161	27	Antisense	ACGAAGACCACAAAACCAGATAAAAAA	2	3	0.02	0.00025
	11153	23	Antisense	CACAAAACCAGATAAAAAATAAA	2	2	0.013	0.00017
	2214	18	Sense	ATATCACGATCTAAGTGT	2	2	0.016	0.00012
	11153	19	Antisense	CACAAAACCAGATAAAAAA	2	2	0.013	0.00017
Polio (25)	4505	25	Sense	CATGGCAGCCCCGGAACAGGTAAAT	8	548	0.073	0.0041
	5790	28	Sense	CTGTGACTGAACAGGGATATCTAAATCT	8	194	0.026	0.0014
	4509	21	Sense	GCAGCCCCGGAACAGGTAAAT	8	176	0.023	0.0013
	1791	26	Antisense	CCAGGGGTGTTCATGACCGGCAGGCC	8	139	0.019	0.0010
	4508	22	Sense	GGCAGCCCCGGAACAGGTAAAT	8	84	0.0071	0.00046
West Nile (4)	10758	21	Sense	CACGCGGCCCTAGCCCCGGTA	1	3	0.054	0.0010
	3212	19	Sense	CGGGACCACGAAGCAATCA	1	2	0.036	0.00067
	3398	19	Sense	ATTGGTGCTGCAGGAGCTG	1	2	0.036	0.00067
	3473	28	Sense	TCAGACCACAGAGACATGATGAAAAGAC	1	2	0.036	0.00067
Dengue (1)	1369	22	Sense	GCAGTCGGAAATGACACAGGAA	1	2	0.33	0.00056
	130	17	Sense	AAACGCGAGAGAAACCG	1	2	0.33	0.00056
Hepatitis C Replicon (23)	4853	25	Sense	CCATGAGGATCGTGGGGCCTAGGAC	13	49	0.00095	0.00013
	3235	22	Sense	CCAGGAGAACGGCCCTCGGGCA	12	157	0.0030	0.00041
	3254	21	Antisense	CCCGAGGGCCGTTCTCCTGGA	12	134	0.0026	0.00035
	3102	25	Sense	CCAGACAGTCGACTTCAGCCTGGAC	12	98	0.0018	0.00018
	3255	23	Antisense	GCCCGAGGGCCGTTCTCCTGGAG	12	65	0.0013	0.00017
Hepatitis C Virion (11)	3750	22	Sense	TCACGCGGAACGCTGATGTCAT	7	21	0.0044	5.61E-05
	485	21	Sense	ACAAGGAAGACTTCGGAGCGG	5	17	0.0042	6.28E-05
	485	20	Sense	ACAAGGAAGACTTCGGAGCG	5	13	0.0032	4.80E-05
	3864	23	Antisense	CAGAGCACCGGCCCCCCCGAGGA	4	13	0.0035	0.00011
	1087	20	Antisense	CTGCACGGCCACATTCGGTG	4	9	0.0026	3.36E-05
	5806	26	Antisense	GGACGCTAACCAGCCTCCCATGATGT	4	4	0.00098	4.17E-05
