# Supplementary material for: Six RNA Viruses and Forty-One Hosts: Viral Small RNAs and Modulation of Small RNA Repertoires in Vertebrate and Invertebrate Systems
Source: PLoS Pathog. 2010 Feb 12;6(2):e1000764. doi: 10.1371/journal.ppat.1000764 (PMC2820531; doi:10.1371/journal.ppat.1000764)
Supplement: Table S3 — Comprehensive list of virus-host systems that were surveyed, with sample descriptions, bulk frequencies and ratios of various classes of small RNAs, and frequencies of ‘rogue’ viral and host genomic matches to vsRNAs. Samples are ordered first by sequencing platform, second by type of virus used in the infection, third by genotype, fourth by cell/tissue type, and fifth by time-point. (0.41 MB PDF) [file ppat.1000764.s024.pdf]

Sequencing Platform	*Virus System*	*Host System (Genotype)*	*Sample Name*	*Sample Description*	*Time-point*	*5P-IN or 5P*	*# with Barcode*	*# RNAs longer than 16 bp.*	*# Sense vsRNAs*	*Sense vsRNAs as a fraction of miRNAs*	*# Antisense vsRNAs*	*Antisense vsRNAs as a fraction of miRNAs*	*Total # vsRNAs*	*Total vsRNAs as a fraction of miRNAs*	*Total miRNAs*	*miRNAs as a fraction of RNAs longer than 16 bp.*	*Total vsRNAs as a fraction of RNAs longer than 16 bp*	*(+) vsRNA : (-) vsRNA*	*"False" hits to other viruses (# seq)*	*"False" hits to other viruses (virus names)*	*False hits as a % of # Parsed*	*Ratio of "False" : "True" hits*	*# vsRNAs that also map to Genomic Loci*
GS-20/GS-FLX	Dengue	In-vitro	454-91	Huh7	0 hpi	5P	8,476	4,859							1,489	0.3064							
GS-20/GS-FLX	Dengue	In-vitro	454-73	Huh7	2.5 hpi	5P	7,463	5,504							1,786	0.3245							
GS-20/GS-FLX	Dengue	In-vitro	454-74	Huh7	12 hpi	5P	14,264	10,667							2,952	0.2767							
GS-20/GS-FLX	Dengue	In-vitro	454-75	Huh7	24 hpi	5P	15,508	8,387	6	0.0017	0	0.0000	6	0.0017	3,569	0.4255	0.000715	6+ : 0-					
GS-20/GS-FLX	Dengue (uninfected)	In-vitro	454-95	U937; +ADE	"2 hpi"	5P	5,714	3,503							1,649	0.4707							
GS-20/GS-FLX	Dengue	In-vitro	454-92	U937; +ADE	2 hpi	5P	6,040	3,538							1,445	0.4084							
GS-20/GS-FLX	Dengue	In-vitro	454-93	U937; +ADE	15 hpi	5P	8,071	3,876							1,513	0.3904							
GS-20/GS-FLX	Dengue	In-vitro	454-94	U937; +ADE	35 hpi	5P	5,942	4,549							2,728	0.5997							
GS-20/GS-FLX	Dengue	In-vitro	454-157	Primary MDDC	4 hpi	5P	7,343	2,393							38	0.0159							
GS-20/GS-FLX	Dengue	In-vitro	454-158	Primary MDDC	24 hpi	5P	5,129	1,801							277	0.1538							
GS-20/GS-FLX	Hepatitis C (negative control)	In-vitro	454-96	Huh7	N/A	5P	12,320	9,153							4,268	0.4663							
GS-20/GS-FLX	Hepatitis C (Replicon; type 2A)	In-vitro	454-61	Huh7	N/A	5P	8,342	3,987	26	0.0189	18	0.0131	44	0.0320	1,373	0.3444	0.011036	1.44					
GS-20/GS-FLX	Hepatitis C (Replicon; type 2A)	In-vitro	454-62	Huh7; treated with 5 U/mL IFN	72 hr.post-treatment	5P	10,828	5,840	6	0.0029	4	0.0020	10	0.0049	2,039	0.3491	0.001712	1.50					
GS-20/GS-FLX	Hepatitis C (Replicon; type 2A)	In-vitro	454-63	Huh7; treated with 100 U/mL IFN	72 hr.post-treatment	5P	8,071	5,327	3	0.0034	1	0.0011	4	0.0046	877	0.1646	0.000751	3.00					
GS-20/GS-FLX	Hepatitis C (negative control)	In-vitro	454-97	Huh7.5	N/A	5P	5,685	4,032							1,900	0.4712							
GS-20/GS-FLX	Hepatitis C (Infectious clone)	In-vitro	454-83	Huh7.5	1 dpi	5P	12,501	8,080							1,498	0.1854							
GS-20/GS-FLX	Hepatitis C (Infectious clone)	In-vitro	454-84	Huh7.5	3 dpi	5P	14,545	8,296	3	0.0009	0	0.0000	3	0.0009	3,183	0.3837							
GS-20/GS-FLX	Hepatitis C (Infectious clone)	In-vitro	454-85	Huh7.5	5 dpi	5P	7,920	4,897	27	0.0157	17	0.0099	44	0.0257	1,715	0.3502	0.008985	1.59					
GS-20/GS-FLX	Hepatitis C (Infectious clone)	In-vitro	454-86	Huh7.5	11 dpi	5P	9,202	5,092	12	0.0062	10	0.0051	22	0.0113	1,950	0.3830	0.004321	1.20					

Sequencing Platform	Virus System	Host System (Genotype)	Sample Name	Sample Description	Time-point	5P-IN or 5P	# with Barcode	# RNAs longer than 16 bp.	# Sense vsRNAs	Sense vsRNAs as a fraction of miRNAs	# Antisense vsRNAs	Antisense vsRNAs as a fraction of miRNAs	Total # vsRNAs	Total vsRNAs as a fraction of miRNAs	Total miRNAs	miRNAs as a fraction of # with Barcode	Total vsRNAs as a fraction of # with Barcode	(+) vsRNA : (-) vsRNA	"False" hits to other viruses (# seq)	"False" hits to other viruses (virus names)	False hits as a % of # Parsed	Ratio of "False" : "True" hits	# vsRNAs that also map to Genomic Loci
GS-20/GS-FLX	Polio	In-vitro	454-45	Hela	2 hpi	5P	8,033	5,994							1,305	0.2177							
GS-20/GS-FLX	Polio	In-vitro	454-46	Hela	2 hpi	5P-IN	6,873	5,638							148	0.0263							
GS-20/GS-FLX	Polio	In-vitro	454-47	Hela	5.5 hpi	5P	7,523	5,482							1,133	0.2067							
GS-20/GS-FLX	Polio	In-vitro	454-48	Hela	5.5 hpi	5P-IN	7,741	7,175	2	0.0513	1	0.0256	3	0.0769	39	0.0054	0.000418	2.00					
GS-20/GS-FLX	Polio	In-vitro	454-49	K562	Persistent	5P	7,561	6,123	6	0.0017	2	0.0006	8	0.0023	3,502	0.5719	0.001307	3.00					
GS-20/GS-FLX	Polio	In-vitro	454-50	K562	Persistent	5P-IN	12,029	8,814	9	0.1364	0	0.0000	9	0.1364	66	0.0075	0.001021	9+ : 0-					
GS-20/GS-FLX	Polio	In-vitro (Ago-2 +/+)	454-193	MEF	5 hpi	5P	22,559	13,773	24	0.0032	11	0.0015	35	0.0047	7,511	0.5453	0.002541	2.18					
GS-20/GS-FLX	Polio	In-vitro (Ago-2 -/-)	454-194	MEF	5 hpi	5P	18,406	5,871	65	0.0226	48	0.0167	113	0.0392	2,880	0.4905	0.019247	1.35					
GS-20/GS-FLX	Polio (negative control)	M. musculus (IFN-/-; PVR +/+)	454-168	Brain	N/A	5P	11,580	8,702							6,932	0.7966							
GS-20/GS-FLX	Polio (negative control)	M. musculus (IFN-/-; PVR +/+)	454-167	Leg muscle	N/A	5P	5,240	3,594							2,719	0.7565							
GS-20/GS-FLX	Polio	M. musculus (IFN-/-; PVR +/+); paralyzed	454-41	Brain	4 dpi	5P	8,762	7,258							4,228	0.5825							
GS-20/GS-FLX	Polio	M. musculus (IFN-/-; PVR +/+); paralyzed	454-42	Brain	4 dpi	5P-IN	10,655	9,690							437	0.0451							
GS-20/GS-FLX	Polio	M. musculus (IFN-/-; PVR +/+); paralyzed	454-43	Leg muscle	4 dpi	5P	14,342	9,132	1	0.0004	7	0.0029	8	0.0033	2,392	0.2619	0.000876	0.14					
GS-20/GS-FLX	Polio	M. musculus (IFN-/-; PVR +/+); paralyzed	454-44	Leg muscle	4 dpi	5P-IN	10,443	9,933	7	0.1892	6	0.1622	13	0.3514	37	0.0037	0.001309	1.17					
GS-20/GS-FLX	Polio (negative control)	M. musculus (PVR +/+)	454-166	Brain	N/A	5P	9,021	5,236							1,964	0.3751							
GS-20/GS-FLX	Polio (negative control)	M. musculus (PVR +/+)	454-165	Leg muscle	N/A	5P	11,895	8,498							1,091	0.1284							
GS-20/GS-FLX	Polio	M. musculus (PVR +/+); non paralyzed	454-162	Brain	4 dpi	5P	11,265	9,358							8,075	0.8629							
GS-20/GS-FLX	Polio	M. musculus (PVR +/+); non paralyzed	454-170	Brain	4 dpi	5P-IN	12,518	10,882							5	0.0005							
GS-20/GS-FLX	Polio	M. musculus (PVR +/+); paralyzed	454-164	Brain	4 dpi	5P	11,427	8,351	2	0.0006	0	0.0000	2	0.0006	3,567	0.4271	0.000239	2+ : 0-					
GS-20/GS-FLX	Polio	M. musculus (PVR +/+); paralyzed	454-172	Brain	4 dpi	5P-IN	18,588	17,013							6	0.0004							
GS-20/GS-FLX	Polio	M. musculus (PVR +/+); non paralyzed	454-161	Leg Muscle	4 dpi	5P	7,863	4,871							1,074	0.2205							
GS-20/GS-FLX	Polio	M. musculus (PVR +/+); non paralyzed	454-169	Leg Muscle	4 dpi	5P-IN	25,649	23,306							17	0.0007							
GS-20/GS-FLX	Polio	M. musculus (PVR +/+); paralyzed	454-163	Leg muscle	4 dpi	5P	12,315	8,449	0	0.0000	1	0.0006	1	0.0006	1,627	0.1926	0.000118	0+ : 1-					
GS-20/GS-FLX	Polio	M. musculus (PVR +/+); paralyzed	454-171	Leg Muscle	4 dpi	5P-IN	15,535	13,352	2	0.1333	0	0.0000	2	0.1333	15	0.0011	0.000150	2+ : 0-					

Sequencing Platform	Virus System	Host System (Genotype)	Sample Name	Sample Description	Time-point	5P-IN or 5P	# with Barcode	# RNAs longer than 16 bp.	# Sense vsRNAs	Sense vsRNAs as a fraction of miRNAs	# Antisense vsRNAs	Antisense vsRNAs as a fraction of miRNAs	Total # vsRNAs	Total vsRNAs as a fraction of miRNAs	Total miRNAs	miRNAs as a fraction of # with Barcode	Total vsRNAs as a fraction of # with Barcode	(+) vsRNA : (-) vsRNA	"False" hits to other viruses (# seq)	"False" hits to other viruses (virus names)	False hits as a % of # Parsed	Ratio of "False" : "True" hits	# vsRNAs that also map to Genomic Loci
GS-20/GS-FLX	Vesicular Stomatitis	In-vitro (Ago-2 +/+)	454-87	MEF	4 hpi	5P	10,836	7,813	13	0.0031	2	0.0005	15	0.0036	4,196	0.5371	0.001920	6.50					2
GS-20/GS-FLX	Vesicular Stomatitis (negative control)	In-vitro (Ago-2 +/+)	454-177	MEF	N/A	5P	10,444	8,243							7,010	0.8504							
GS-20/GS-FLX	Vesicular Stomatitis	In-vitro (Ago-2 -/-)	454-88	MEF	4 hpi	5P	10,730	7,911	35	0.0133	7	0.0027	42	0.0160	2,633	0.3328	0.005309	5.00					
GS-20/GS-FLX	Vesicular Stomatitis (negative control)	In-vitro (Ago-2 -/-)	454-178	MEF	N/A	5P	15,061	11,257							7,297	0.6482							
GS-20/GS-FLX	Vesicular Stomatitis	In-vitro	454-179	BHK-21	4 hpi	5P	17,422	15,876	56	0.0060	52	0.0056	108	0.0116	9,277	0.5843	0.006803	1.08					
GS-20/GS-FLX	Vesicular Stomatitis (negative control)	In-vitro	454-180	BHK-21	N/A	5P	15,002	11,385							8,330	0.7317							
GS-20/GS-FLX	Vesicular Stomatitis	In-vitro	454-181	Hela	4 hpi	5P	11,548	10,424	19	0.0028	0	0.0000	19	0.0028	6,796	0.6520	0.001823	19+ : 0-					
GS-20/GS-FLX	Vesicular Stomatitis (negative control)	In-vitro	454-182	Hela	N/A	5P	14,699	9,537							9,228	0.9676							
GS-20/GS-FLX	West Nile (negative control)	In-vitro (B6)	454-152	Cortical Neurons	"4 hpi"	5P	21,490	10,585							2,318	0.2190							
GS-20/GS-FLX	West Nile	In-vitro (B6)	454-108	Cortical Neurons	4 hpi	5P	8,391	5,453							2,962	0.5432							
GS-20/GS-FLX	West Nile	In-vitro (B6)	454-109	Cortical Neurons	16 hpi	5P	6,679	4,928							2,750	0.5580							
GS-20/GS-FLX	West Nile (negative control)	In-vitro (B6)	454-146	Dendritic Cells	"4 hpi"	5P	7,526	4,875							956	0.1961							
GS-20/GS-FLX	West Nile	In-vitro (B6)	454-110	Dendritic Cells	4 hpi	5P	8,728	6,621							970	0.1465							
GS-20/GS-FLX	West Nile	In-vitro (B6)	454-111	Dendritic Cells	16 hpi	5P	6,511	3,253	1	0.0021	0	0.0000	1	0.0021	476	0.1463	0.000307	1+ : 0-					
GS-20/GS-FLX	West Nile (negative control)	In-vitro (B6)	454-149	Macrophages	"4 hpi"	5P	16,472	6,298							629	0.0999							
GS-20/GS-FLX	West Nile	In-vitro (B6)	454-102	Macrophages	4 hpi	5P	12,594	4,224							1,373	0.3250							
GS-20/GS-FLX	West Nile	In-vitro (B6)	454-103	Macrophages	16 hpi	5P	6,103	3,526							1,568	0.4447							
GS-20/GS-FLX	West Nile (negative control)	In-vitro (B6)	454-153	MEF	"4 hpi"	5P	8,091	5,164							2,169	0.4200							
GS-20/GS-FLX	West Nile	In-vitro (B6)	454-89	MEF	4 hpi	5P	8,422	6,247							3,653	0.5848							
GS-20/GS-FLX	West Nile	In-vitro (B6)	454-90	MEFs	16 hpi	5P	5,204	3,842							2,020	0.5258							
GS-20/GS-FLX	West Nile (negative control)	In-vitro (IFNab-/-)	454-151	Macrophages	"4 hpi"	5P	16,236	5,857							1,309	0.2235							
GS-20/GS-FLX	West Nile	In-vitro (IFNa/b -/-)	454-106	Macrophages	4 hpi	5P	3,696	2,461							367	0.1491							

Sequencing Platform	*Virus System*	*Host System (Genotype)*	*Sample Name*	*Sample Description*	*Time-point*	*5P-IN or 5P*	*# with Barcode*	*# RNAs longer than 16 bp.*	*# Sense vsRNAs*	*Sense vsRNAs as a fraction of miRNAs*	*# Antisense vsRNAs*	*Antisense vsRNAs as a fraction of miRNAs*	*Total # vsRNAs*	*Total vsRNAs as a fraction of miRNAs*	*Total miRNAs*	*miRNAs as a fraction of # with Barcode*	*Total vsRNAs as a fraction of # with Barcode*	*(+) vsRNA : (-) vsRNA*	*"False" hits to other viruses (# seq)*	*"False" hits to other viruses (virus names)*	*False hits as a % of # Parsed*	*Ratio of "False" : "True" hits*	*# vsRNAs that also map to Genomic Loci*
GS-20/GS-FLX	West Nile	In-vitro (IFNa/b -/-)	454-107	Macrophages	16 hpi	5P	11,186	7,458							4,109	0.5510							
GS-20/GS-FLX	West Nile (negative control)	In-vitro (IFNab-/-)	454-148	Dendritic Cells	"4 hpi"	5P	6,624	4,213							165	0.0392							
GS-20/GS-FLX	West Nile	In-vitro (IFNab-/-)	454-114	Dendritic Cells	4 hpi	5P	10,689	7,005							1,280	0.1827							
GS-20/GS-FLX	West Nile (negative control)	In-vitro (IFNab-/-)	454-115	Dendritic Cells	16 hpi	5P	16,033	6,891							203	0.0295							
GS-20/GS-FLX	West Nile (negative control)	In-vitro (PKR /-,RNaseL-/-)	454-147	Dendritic Cells	"4 hpi"	5P	28,042	6,467							327	0.0506							
GS-20/GS-FLX	West Nile	In-vitro (PKR /- RL-/-)	454-112	Dendritic Cells	4 hpi	5P	8,501	4,849							845	0.1743							
GS-20/GS-FLX	West Nile	In-vitro (PKR /- RL-/-)	454-113	Dendritic Cells	16 hpi	5P	5,107	2,426							345	0.1422							
GS-20/GS-FLX	West Nile (negative control)	In-vitro (PKR /-,RNaseL-/-)	454-150	Macrophages	"4 hpi"	5P	13,029	4,134							347	0.0839							
GS-20/GS-FLX	West Nile	In-vitro (PKR /- RL-/-)	454-105	Macrophages	16 hpi	5P	6,620	4,496							1,616	0.3594							
GS-20/GS-FLX	West Nile	M. musculus (B6)	454-129	Brain	1 dpi	5P	32,254	27,409							11,743	0.4284							
GS-20/GS-FLX	West Nile	M. musculus (B6)	454-132	Brain	3 dpi	5P	11,565	9,192							7,949	0.8648							
GS-20/GS-FLX	West Nile	M. musculus (B6)	454-130	LN	1 dpi	5P	15,292	9,851							5,500	0.5583							
GS-20/GS-FLX	West Nile	M. musculus (B6)	454-133	LN	3 dpi	5P	25,458	22,236							15,893	0.7147							
GS-20/GS-FLX	West Nile	M. musculus (B6)	454-116	Spleen	1 dpi	5P	13,263	11,072							4,056	0.3663							
GS-20/GS-FLX	West Nile	M. musculus (B6)	454-131	Spleen	3 dpi	5P	13,767	11,390							6,202	0.5445							
GS-20/GS-FLX	West Nile	M. musculus (IFNab-/-)	454-141	Brain	1 dpi	5P	16,762	11,516							8,051	0.6991							
GS-20/GS-FLX	West Nile	M. musculus (IFNab-/-)	454-144	Brain	3 dpi	5P	6,176	4,396							2,995	0.6813							
GS-20/GS-FLX	West Nile	M. musculus (IFNab-/-)	454-142	LN	1 dpi	5P	30,007	25,938	1	0.0001	0	0.0000	1	0.0001	19,209	0.7406	0.000039	1+ : 0-					
GS-20/GS-FLX	West Nile	M. musculus (IFNab-/-)	454-145	LN	3 dpi	5P	7,584	4,687							31	0.0066							
GS-20/GS-FLX	West Nile	M. musculus (IFNab-/-)	454-140	Spleen	1 dpi	5P	23,363	19,536	1	0.0001	0	0.0000	1	0.0001	8,416	0.4308	0.000051	1+ : 0-					
GS-20/GS-FLX	West Nile	M. musculus (IFNab-/-)	454-143	Spleen	3 dpi	5P	10,195	8,263	42	0.0140	14	0.0047	56	0.0187	2,998	0.3628	0.006777	3.00					
GS-20/GS-FLX	West Nile	M. musculus (PKR-/- RNaseL-/-)	454-135	Brain	1 dpi	5P	18,943	16,890							12,535	0.7422							
GS-20/GS-FLX	West Nile	M. musculus (PKR-/- RNaseL-/-)	454-138	Brain	3 dpi	5P	32,427	28,015							18,984	0.6776							
GS-20/GS-FLX	West Nile	M. musculus (PKR-/- RNaseL-/-)	454-136	LN	1 dpi	5P	15,686	11,689							6,174	0.5282							
GS-20/GS-FLX	West Nile	M. musculus (PKR-/- RNaseL-/-)	454-139	LN	3 dpi	5P	21,181	16,274							8,745	0.5374							
GS-20/GS-FLX	West Nile	M. musculus (PKR-/- RNaseL-/-)	454-134	Spleen	1 dpi	5P	21,810	17,890							10,597	0.5923							
GS-20/GS-FLX	West Nile	M. musculus (PKR-/- RNaseL-/-)	454-137	Spleen	3 dpi	5P	18,559	11,577							175	0.0151							

Sequencing Platform	*Virus System*	*Host System (Genotype)*	*Sample Name*	*Sample Description*	*Time-point*	*5P-IN or 5P*	*# with Barcode*	*# RNAs longer than 16 bp.*	*# Sense vsRNAs*	*Sense vsRNAs as a fraction of miRNAs*	*# Antisense vsRNAs*	*Antisense vsRNAs as a fraction of miRNAs*	*Total # vsRNAs*	*Total vsRNAs as a fraction of miRNAs*	*Total miRNAs*	*miRNAs as a fraction of # with Barcode*	*Total vsRNAs as a fraction of # with Barcode*	*(+) vsRNA : (-) vsRNA*	*"False" hits to other viruses (# seq)*	*"False" hits to other viruses (virus names)*	*False hits as a % of # Parsed*	*Ratio of "False" : "True" hits*	*# vsRNAs that also map to Genomic Loci*
Solexa	Poliovirus	M. musculus (IFN-/-; PVR +/+); paralyzed	SOL-1	Muscle	4 dpi	5P	67,155	67,155	75	0.0072	55	0.0053	130	0.0125	10,430	0.1553	0.001936	1.36					3
Solexa	Poliovirus	M. musculus (IFN-/-; PVR +/+); paralyzed	SOL-81	Brain	4 dpi	5P	356,393	356,393	42	0.0002	7	0.0000	49	0.0003	176,382	0.4949	0.000137	6.00	2	HCVrep, HCVvir	0.000561178	0.040816327	
Solexa	Poliovirus	M. musculus (IFN-/-; PVR +/+); paralyzed	SOL-77	Brain	4 dpi	5P-IN	220,755	220,755	60	0.0010	1	0.0000	61	0.0010	59,307	0.2687	0.000276	60.00	2	HCVrep	0.000905982	0.032786885	
Solexa	Poliovirus	M. musculus (IFN-/-; PVR +/+); paralyzed	SOL-78	Muscle	4 dpi	5P-IN	55,943	55,943	18	0.0460	9	0.0230	27	0.0691	391	0.0070	0.000483	2.00					
Solexa	Poliovirus	In-vitro	SOL-2	Hela	5.5 hpi	5P	538,618	538,618	114	0.0051	16	0.0007	130	0.0058	22,461	0.0417	0.000241	7.13					
Solexa	Poliovirus (negative control)	In-vitro (IFNabR+/+)	SOL-59	MEF	6 hpi	5P	90,429	90,429							11,674	0.1291							
Solexa	Poliovirus	In-vitro (IFNabR+/+)	SOL-60	MEF	6 hpi	5P	194,038	194,038	2291	0.0437	91	0.0017	2382	0.0454	52,481	0.2705	0.012276	25.18					
Solexa	Poliovirus (negative control)	In-vitro (IFNabR-/-)	SOL-61	MEF	6 hpi	5P	112,652	112,652							19,702	0.1749							
Solexa	Poliovirus	In-vitro (IFNabR-/-)	SOL-62	MEF	6 hpi	5P	319,911	319,911	2071	0.0749	88	0.0032	2159	0.0781	27,658	0.0865	0.006749	23.53	1	HCVrep	0.000312587	0.000463177	2
Solexa	Poliovirus (negative control)	In-vitro (eri-1+/+)	SOL-63	MEF	6 hpi	5P	185,782	185,782							52,222	0.2811							
Solexa	Poliovirus	In-vitro (eri-1+/+)	SOL-64	MEF	6 hpi	5P	267,768	267,768	440	0.0156	188	0.0067	628	0.0223	28,195	0.1053	0.002345	2.34					
Solexa	Poliovirus (negative control)	In-vitro (eri-1-/-)	SOL-65	MEF	6 hpi	5P	232,587	232,587							75,087	0.3228							
Solexa	Poliovirus	In-vitro (eri-1-/-)	SOL-66	MEF	6 hpi	5P	89,337	89,337	990	0.0584	718	0.0424	1708	0.1008	16,938	0.1896	0.019119	1.38					
Solexa	Poliovirus (negative control)	In-vitro (dcr-1+/+)	SOL-67	MEF	6 hpi	5P	257,714	257,714							18,160	0.0705							
Solexa	Poliovirus	In-vitro (dcr-1+/+)	SOL-68	MEF	6 hpi	5P	233,800	233,800	2319	0.0468	1316	0.0266	3635	0.0734	49,505	0.2117	0.015547	1.76	1	HCVrep	0.000427716	0.000275103	1
Solexa	Poliovirus (negative control)	In-vitro (dcr-1-/-)	SOL-69	MEF	6 hpi	5P	13,984	13,984							11	0.0008							
Solexa	Poliovirus	In-vitro (dcr-1-/-)	SOL-70	MEF	6 hpi	5P	114,504	114,504	476	7.4375	345	5.3906	821	12.8281	64	0.0006	0.007170	1.38					1
Solexa	Poliovirus	In-vitro (ago2+/+)	SOL-82	MEF	5 hpi	5P	69,089	69,089	33	0.0012	20	0.0007	53	0.0019	28,022	0.4056	0.000767	1.65	1	HCVrep	0.001447408	0.018867925	
Solexa	Poliovirus	In-vitro (ago2-/-)	SOL-83	MEF	5 hpi	5P	56,402	56,402	273	0.0374	136	0.0187	409	0.0561	7291	0.1293	0.007252	2.01					
Solexa	Poliovirus	In-vitro (ago2+/+)	SOL-79	MEF	5 hpi	5P-IN	61,844	61,844	127	0.0524	26	0.0107	153	0.0631	2423	0.0392	0.002474	4.88					
Solexa	Poliovirus	In-vitro (ago2-/-)	SOL-80	MEF	5 hpi	5P-IN	64,747	64,747	65	0.0412	26	0.0165	91	0.0577	1576	0.0243	0.001405	2.50	1	HCVrep	0.001544473	0.010989011	

Sequencing Platform	*Virus System*	*Host System (Genotype)*	*Sample Name*	*Sample Description*	*Time-point*	*5P-IN or 5P*	*# with Barcode*	*# RNAs longer than 16 bp.*	*# Sense vsRNAs*	*Sense vsRNAs as a fraction of miRNAs*	*# Antisense vsRNAs*	*Antisense vsRNAs as a fraction of miRNAs*	*Total # vsRNAs*	*Total vsRNAs as a fraction of miRNAs*	*Total miRNAs*	*miRNAs as a fraction of # with Barcode*	*Total vsRNAs as a fraction of # with Barcode*	*(+) vsRNA : (-) vsRNA*	*"False" hits to other viruses (# seq)*	*"False" hits to other viruses (virus names)*	*False hits as a % of # Parsed*	*Ratio of "False" : "True" hits*	*# vsRNAs that also map to Genomic Loci*
Solexa	Hepatitis C (Infectious clone; negative control)	In-vitro	SOL-89	Huh7.5	0 dpi (uninf-A)	5P	253,176	253,176							47,233	0.1866							
Solexa	Hepatitis C (Infectious clone)	In-vitro	SOL-90	Huh7.5	0 dpi (+HCV-A)	5P	151,828	151,828							21,040	0.1386							
Solexa	Hepatitis C (Infectious clone)	In-vitro	SOL-91	Huh7.5	1 dpi (+HCV-A)	5P	187,137	187,137	2	0.0000	0	0.0000	2	0.0000	80,890	0.4323	0.000011	2+ : 0-					
Solexa	Hepatitis C (Infectious clone)	In-vitro	SOL-92	Huh7.5	3 dpi (+HCV-A)	5P	568,886	568,886	183	0.0012	139	0.0009	322	0.0021	152,425	0.2679	0.000566	1.32					
Solexa	Hepatitis C (Infectious clone)	In-vitro	SOL-93	Huh7.5	6 dpi (+HCV-A)	5P	146,972	146,972	182	0.0040	167	0.0037	349	0.0077	45,269	0.3080	0.002375	1.09					
Solexa	Hepatitis C (Infectious clone)	In-vitro	SOL-94	Huh7.5	9 dpi (+HCV-A)	5P	244,248	244,248	391	0.0170	317	0.0138	708	0.0308	22,984	0.0941	0.002899	1.23					
Solexa	Hepatitis C (Infectious clone)	In-vitro	SOL-95	Huh7.5	11 dpi (+HCV A)	5P	250,510	250,510	536	0.0094	500	0.0088	1036	0.0182	57,039	0.2277	0.004136	1.07	1	FHV	0.000399186	0.000965251	1
Solexa	Hepatitis C (Infectious clone)	In-vitro	SOL-96	Huh7.5	15 dpi (+HCV A)	5P	114,109	114,109	394	0.0314	302	0.0241	696	0.0555	12,550	0.1100	0.006099	1.30					
Solexa	Hepatitis C (Infectious clone)	In-vitro	SOL-3	Huh7.5	5 dpi	5P	255,819	255,819	848	0.0654	888	0.0685	1,736	0.1338	12,970	0.0507	0.006786	0.95					
Solexa	Hepatitis C (Infectious clone)	In-vitro	SOL-76	Huh7.5	5 dpi	5P-IN	116,255	116,255	312	0.1073	273	0.0938	585	0.2011	2909	0.0250	0.005032	1.14					9
Solexa	Hepatitis C (Replicon; type 2A)	In-vitro	SOL-4	Huh7	Late passage	5P	39,845	39,845	65	0.0331	56	0.0285	121	0.0616	1,963	0.0493	0.003037	1.16					
Solexa	Hepatitis C (Replicon; type 2A)	In-vitro	SOL-107	Huh7	Late passage; Amplicon-1	5P	51,045	51,045	164	0.0254	138	0.0214	302	0.0467	6461	0.1266	0.005916	1.19	4	FHV	0.007836223	0.013245033	1
Solexa	Hepatitis C (Replicon; type 2A)	In-vitro	SOL-200	Huh7	Late passage; Amplicon-2	5P	359,794	359,794	1103	0.0142	883	0.0114	1986	0.0255	77,785	0.2162	0.005520	1.25					3
Solexa	Hepatitis C (Replicon; type 2A)	In-vitro	SOL-202	Huh7	Late passage; Amplicon-3	5P	131,104	131,104	559	0.0283	501	0.0253	1060	0.0536	19,771	0.1508	0.008085	1.12					
Solexa	Hepatitis C (Replicon; type 2A)	In-vitro	SOL-204	Huh7	Late passage; Amplicon-4	5P	83,714	83,714	373	0.0307	304	0.0250	677	0.0558	12,137	0.1450	0.008087	1.23					
Solexa	Hepatitis C (Replicon; type 2A)	In-vitro	SOL-109	Huh7	Late passage	5P-IN	178,464	178,464	910	0.0763	785	0.0658	1695	0.1421	11,927	0.0668	0.009498	1.16					1
Solexa	Hepatitis C (Replicon; type 2A)	In-vitro	SOL-176	Huh7	Early passage; Amplicon-1	5P	1,946,923	1,946,923	6959	0.1248	5853	0.1050	12812	0.2297	55,768	0.0286	0.006581	1.19					
Solexa	Hepatitis C (Replicon; type 2A)	In-vitro	SOL-201	Huh7	Early passage; Amplicon-2	5P	1,692,531	1,692,531	4438	0.0429	3694	0.0357	8132	0.0787	103,356	0.0611	0.004805	1.20					
Solexa	Hepatitis C (Replicon; type 2A)	In-vitro	SOL-203	Huh7	Early passage; Amplicon-3	5P	1,200,175	1,200,175	3690	0.1263	3029	0.1037	6719	0.2300	29,209	0.0243	0.005598	1.22					1
Solexa	Hepatitis C (Replicon; type 2A)	In-vitro	SOL-205	Huh7	Early passage; Amplicon-4	5P	581,639	581,639	1569	0.1195	1271	0.0968	2840	0.2163	13,128	0.0226	0.004883	1.23					
Solexa	Hepatitis C (Replicon; type 2A)	In-vitro	SOL-179	Huh7	Early passage	5P-IN	2,211,220	2,211,220	7964	0.4227	6837	0.3629	14801	0.7855	18,842	0.0085	0.006694	1.16					

Sequencing Platform	Virus System	Host System (Genotype)	Sample Name	Sample Description	Time-point	5P-IN or 5P	# with Barcode	# RNAs longer than 16 bp.	# Sense vsRNAs	Sense vsRNAs as a fraction of miRNAs	# Antisense vsRNAs	Antisense vsRNAs as a fraction of miRNAs	Total # vsRNAs	Total vsRNAs as a fraction of miRNAs	Total miRNAs	miRNAs as a fraction of # with Barcode	Total vsRNAs as a fraction of # with Barcode	(+) vsRNA : (-) vsRNA	"False" hits to other viruses (# seq)	"False" hits to other viruses (virus names)	False hits as a % of # Parsed	Ratio of "False" : "True" hits	# vsRNAs that also map to Genomic Loci
Solexa	Hepatitis C (Replicon; type 2A)	IP: Ago-1	PP-Sol-32	Huh7	N/A	5P	282,911	282,911	203	0.0080	188	0.0074	391	0.0153	25494	0.0901	0.001382	1.08					
Solexa	Hepatitis C (Replicon; type 2A)	IP: Ago-2	PP-Sol-48	Huh7	N/A	5P	2,147,467	2,147,467	1,174	0.0067	1029	0.0059	2,203	0.0126	175,489	0.0817	0.001026	1.14					
Solexa	Hepatitis C (Replicon; type 2A)	IP: Ago-3	PP-Sol-34	Huh7	N/A	5P	244,965	244,965	219	0.0085	198	0.0077	417	0.0162	25788	0.1053	0.001702	1.11					
Solexa	Hepatitis C (Replicon; type 2A)	IP: Ago-4	PP-Sol-35	Huh7	N/A	5P	300,266	300,266	294	0.0156	207	0.0110	501	0.0265	18884	0.0629	0.001669	1.42					
Solexa	Hepatitis C (Replicon; type 2A)	IP: Mock	PP-Sol-36	Huh7	N/A	5P	126,022	126,022	58	0.0213	28	0.0103	86	0.0315	2729	0.0217	0.000682	2.07					
Solexa	Hepatitis C (Replicon; type 2A)	Total RNA: Ago-1	PP-Sol-37	Huh7	N/A	5P	190,114	190,114	97	0.0045	247	0.0113	344	0.0158	21782	0.1146	0.001809	0.39					
Solexa	Hepatitis C (Replicon; type 2A)	Total RNA: Ago-2	PP-Sol-49	Huh7	N/A	5P	891,858	891,858	829	0.0181	585	0.0128	1,414	0.0309	45827	0.0514	0.001585	1.42					
Solexa	Hepatitis C (Replicon; type 2A)	Total RNA: Ago-3	PP-Sol-39	Huh7	N/A	5P	181,260	181,260	115	0.0116	155	0.0157	270	0.0273	9889	0.0546	0.001490	0.74					
Solexa	Hepatitis C (Replicon; type 2A)	Total RNA: Ago-4	PP-Sol-40	Huh7	N/A	5P	210,262	210,262	360	0.0169	268	0.0126	628	0.0295	21272	0.1012	0.002987	1.34					
Solexa	Flock House Virus RNA1deltaB2	C. elegans (PD3243)	SOL-50*	rde-4 ex[rol-6D, pha-1, FHVdeltaRNA1]	24 hours post-heat-shock	5P-IN	199,744	199,744	5	0.0008	4	0.0006	9	0.0014	6,247	0.0313	0.000045	1.25					1
Solexa	Flock House Virus RNA1deltaB2	C. elegans (PD3246)	SOL-51*	rde-1 ex[rol-6D, pha-1, FHVdeltaRNA1]	24 hours post-heat-shock	5P-IN	235,457	235,457							20,604	0.0875							
Solexa	Flock House Virus RNA1deltaB2	C. elegans (PD3244)	SOL-52*	N2 ex[rol-6D, pha-1, FHVdeltaRNA1]	24 hours post-heat-shock	5P-IN	238,477	238,477	55	0.0042	190	0.0144	245	0.0186	13,171	0.0552	0.001027	0.29					
Solexa	Flock House Virus RNA1deltaB2	C. elegans (PD3241)	SOL-53*	rrf-1 ex[rol-6D, pha-1, FHVdeltaRNA1]	24 hours post-heat-shock	5P-IN	268,448	268,448							51,760	0.1928							
Solexa	Flock House Virus RNA1deltaB2	C. elegans (PD3243)	SOL-71	rde-4 ex[rol-6D, pha-1, FHVdeltaRNA1]	24 hours post-heat-shock	5P	937,016	937,016							333,976	0.3564							
Solexa	Flock House Virus RNA1deltaB2	C. elegans (PD3246)	SOL-72	rde-1 ex[rol-6D, pha-1, FHVdeltaRNA1]	24 hours post-heat-shock	5P	894,120	894,120	22	0.0003	20	0.0003	42	0.0006	75,625	0.0846	0.000047	1.10					
Solexa	Flock House Virus RNA1deltaB2	C. elegans (PD3244)	SOL-73	N2 ex[rol-6D, pha-1, FHVdeltaRNA1]	24 hours post-heat-shock	5P	1,052,286	1,052,286	433	0.0021	1041	0.0050	1474	0.0071	207,084	0.1968	0.001401	0.42					
Solexa	Flock House Virus RNA1deltaB2	C. elegans (PD3241)	SOL-74	rrf-1 ex[rol-6D, pha-1, FHVdeltaRNA1]	24 hours post-heat-shock	5P	1,844,150	1,844,150							214,040	0.1161							
