# Supplementary material for: Six RNA Viruses and Forty-One Hosts: Viral Small RNAs and Modulation of Small RNA Repertoires in Vertebrate and Invertebrate Systems
Source: PLoS Pathog. 2010 Feb 12;6(2):e1000764. doi: 10.1371/journal.ppat.1000764 (PMC2820531; doi:10.1371/journal.ppat.1000764)
Supplement: Table S4 — List of vsRNAs that map perfectly to both host and viral genomes. Due to the complete nature of sRNA homology to both viral and host genomes, the origin of the listed vsRNAs cannot be determined. These numbers are also indicated in Table S3. (0.06 MB PDF) [file ppat.1000764.s025.pdf]

Infecting virus	siRNA ID (Sample_vsRNA=Start,Length,Orientation_SeqCount)	Sequence	Length	Start on viral genome	Genomic/RefSeq Hit	Multiple hits?
FHV	Sol-50FHV_vsRNA=1348,20,1_SeqCount=1	GAATCAATTGAACGT AGGGT	20	1348	>emb|Z79759.1| Caenorhabditis elegans Cosmid ZK858, complete sequence	Yes
HCVrep	Sol-76HCVrep_vsRNA=9628,23,1_SeqCount=1	CGTCTGCGACCTGAG CAACAACA	23	9628	>ref|NW_001030694.1|Mm2_11168276_37 Mus musculus chromosome 2 genomic contig, alternate assembly	No
HCVrep	Sol-76HCVrep_vsRNA=9633,24,1_SeqCount=4	GCGACCTGAGCAACA ACATGAATG	24	9633	>ref|NW_001030694.1|Mm2_11168276_37 Mus musculus chromosome 2 genomic contig, alternate assembly	No
HCVrep	Sol-76HCVrep_vsRNA=9633,23,1_SeqCount=2	GCGACCTGAGCAACA ACATGAAT	23	9633	>ref|NW_001030694.1|Mm2_11168276_37 Mus musculus chromosome 2 genomic contig, alternate assembly	No
HCVrep	Sol-76HCVrep_vsRNA=10528,28,-1_SeqCount=1	TTCCTGCGTTATCCCC TGATTCTGTGGA	28	10528	>ref|NW_001073945.1|MmUn_11170298_37 Mus musculus chromosome Un genomic contig, alternate assembly	Yes
HCVrep	Sol-76HCVrep_vsRNA=9611,22,1_SeqCount=1	CGACTGCTGCTGCAA AACGTCT	22	9611	>ref|NW_001030694.1|Mm2_11168276_37 Mus musculus chromosome 2 genomic contig, alternate assembly	No
HCVrep	Sol-203HCVrep_vsRNA=7143,19,1_SeqCount=1	CCCCACCACCCCCCT TGCG	19	7143	>ref|NM_197940.1| Mus musculus WAS/WASL interacting protein family, member 2 (Wipf2),	No
HCVrep	Sol-200HCVrep_vsRNA=10775,22,1_SeqCount=1	TGGCGCTTTCTCATA GCTCACG	22	10775	>ref|NW_001073945.1|MmUn_11170298_37 Mus musculus chromosome Un genomic contig, alternate assembly	Yes
HCVrep	Sol-200HCVrep_vsRNA=10773,24,1_SeqCount=2	CGTGGCGCTTTCTCAT AGCTCACG	24	10773	>ref|NW_001073945.1|MmUn_11170298_37 Mus musculus chromosome Un genomic contig, alternate assembly	Yes
HCVrep	Sol-109HCVrep_vsRNA=10661,28,-1_SeqCount=1	GGGTTTCGCCACCTC TGACTTGAGCGTC	28	10661	>ref|NW_001073945.1|MmUn_11170298_37 Mus musculus chromosome Un genomic contig, alternate assembly	Yes
HCVrep	Sol-107HCVrep_vsRNA=10819,23,1_SeqCount=1	TAGGTCGTTCGCTCC AAGCTGGG	23	10819	>ref|NW_001073945.1|MmUn_11170298_37 Mus musculus chromosome Un genomic contig, alternate assembly	No
HCVvir	Sol-95HCVvir_vsRNA=7609,21,1_SeqCount=1	TTCGGGCTCGGGGTC TTGGTC	21	7609	>ref|NM_177167.4| Mus musculus protein phosphatase 1E (PP2C domain containing)	No
Polio	Sol-70Polio_vsRNA=6702,22,1_SeqCount=1	CATCTCTCAGCCCTG CTTGGTT	22	6702	>ref|NT_039625.7|Mm16_39665_37 Mus musculus chromosome 16 genomic contig, strain C57BL/6J	Yes
Polio	Sol-62Polio_vsRNA=2187,20,-1_SeqCount=2	GGGTCGGCTCCAGGA GGCGC	20	2187	>ref|XM_002345485.1| PREDICTED: Homo sapiens hypothetical protein LOC100291817 (LOC100291817),	Yes
Polio	Sol-1Polio_vsRNA=44,20,-1_SeqCount=3	CCACGTGGGCCTCTG GGGTG	20	44	>ref|NM_021116.2| Homo sapiens adenylate cyclase 1 (brain) (ADCY1), mRNA	No
Polio	Sol-68Polio_vsRNA=2187,19,-1_SeqCount=1	GGGTCGGCTCCAGGA GGCG	19	2187	>ref|XM_002345485.1| PREDICTED: Homo sapiens hypothetical protein LOC100291817 (LOC100291817),	Yes
VSV	454-87VSV_vsRNA=48,18,1_SeqCount=1	TTTAACAGTAATCAA AAT	18	48	>ref|NT_039207.7|Mm2_39247_37 Mus musculus chromosome 2 genomic contig, strain C57BL/6J	Yes
VSV	454-87VSV_vsRNA=11152,17,-1_SeqCount=1	ACAAAACCAGATAAA AA	17	11152	>ref|NT_039706.7|MmX_39746_37 Mus musculus chromosome X genomic contig, strain C57BL/6J	Yes
